# Predicting PD-L1 expression status in patients with non-small cell lung cancer using [^18^F]FDG PET/CT radiomics

**DOI:** 10.1186/s13550-023-00956-9

**Published:** 2023-01-22

**Authors:** Xiaoqian Zhao, Yan Zhao, Jingmian Zhang, Zhaoqi Zhang, Lihua Liu, Xinming Zhao

**Affiliations:** 1 Department of Nuclear Medicine, The Fourth Hospital of Hebei Medical University, 12 Jiankang Road, Shijiazhuang, 050011 Hebei China; 2 Hebei Provincial Key Laboratory of Tumor Microenvironment and Drug Resistance, Shijiazhuang, Hebei China; 3 Department of Oncology, The Fourth Hospital of Hebei Medical University and Hebei Provincial Tumor Hospital, Shijiazhuang, Hebei China; 4 Department of Tumor Immunotherapy, The Fourth Hospital of Hebei Medical University, Shijiazhuang, 050011 Hebei China

**Keywords:** Non-small cell lung cancer, [^18^F]FDG PET/CT, Radiomics, PD-L1

## Abstract

**Background:**

In recent years, immune checkpoint inhibitor (ICI) therapy has greatly changed the treatment prospects of patients with non-small cell lung cancer (NSCLC). Among the available ICI therapy strategies, programmed death-1 (PD-1)/programmed death ligand-1 (PD-L1) inhibitors are the most widely used worldwide. At present, immunohistochemistry (IHC) is the main method to detect PD-L1 expression levels in clinical practice. However, given that IHC is invasive and cannot reflect the expression of PD-L1 dynamically and in real time, it is of great clinical significance to develop a new noninvasive, accurate radiomics method to evaluate PD-L1 expression levels and predict and filter patients who will benefit from immunotherapy. Therefore, the aim of our study was to assess the predictive power of pretherapy [^18^F]-fluorodeoxyglucose ([^18^F]FDG) positron emission tomography/computed tomography (PET/CT)-based radiomics features for PD-L1 expression status in patients with NSCLC.

**Methods:**

A total of 334 patients with NSCLC who underwent [^18^F]FDG PET/CT imaging prior to treatment were analyzed retrospectively from September 2016 to July 2021. The LIFEx7.0.0 package was applied to extract 63 PET and 61 CT radiomics features. In the training group, the least absolute shrinkage and selection operator (LASSO) regression model was employed to select the most predictive radiomics features. We constructed and validated a radiomics model, clinical model and combined model. Receiver operating characteristic (ROC) curves and the area under the ROC curve (AUC) were used to evaluate the predictive performance of the three models in the training group and validation group. In addition, a radiomics nomogram to predict PD-L1 expression status was established based on the optimal predictive model.

**Results:**

Patients were randomly assigned to a training group (*n* = 233) and a validation group (*n* = 101). Two radiomics features were selected to construct the radiomics signature model. Multivariate analysis showed that the clinical stage (odds ratio [OR] 1.579, 95% confidence interval [CI] 0.220–0.703, *P* < 0.001) was a significant predictor of different PD-L1 expression statuses. The AUC of the radiomics model was higher than that of the clinical model in the training group (0.706 vs. 0.638) and the validation group (0.761 vs. 0.640). The AUCs in the training group and validation group of the combined model were 0.718 and 0.769, respectively.

**Conclusion:**

PET/CT-based radiomics features demonstrated strong potential in predicting PD-L1 expression status and thus could be used to preselect patients who may benefit from PD-1/PD-L1-based immunotherapy.

**Supplementary Information:**

The online version contains supplementary material available at 10.1186/s13550-023-00956-9.

## Background

Lung cancer is the leading cause of cancer-related death worldwide, and non-small cell lung cancer (NSCLC) accounts for approximately 85% of all cases [1]. In recent years, the advent of immune checkpoint inhibitor (ICI) therapy has radically shifted the paradigm of treatment of advanced NSCLC and transformed the outlook of NSCLC in the early stages [2]. Among the ICI therapy strategies, PD-1/PD-L1 inhibitors are the most widely used worldwide [3]; these inhibitors reactivate T cells that otherwise would remain suppressed. Studies have shown that PD-1/PD-L1 inhibitors prolong progression-free survival (PFS) and overall survival (OS) in patients with advanced NSCLC [4–6]. Especially for PD-L1-positive patients with NSCLC, the clinical benefits are greater. PD-1/PD-L1 inhibitors can improve the OS of patients compared with first-line chemotherapy [7–9]. At present, PD-L1 detection methods mainly include immunohistochemistry (IHC), enzyme-linked immunosorbent assay, quantitative immunofluorescence and flow cytometry, among which IHC is the most widely applied [10]. However, IHC requires tumor tissue for detection, which is invasive and cannot dynamically reflect the expression status of PD-L1 [11]. Moreover, the application of IHC is greatly limited for tumors that are not easily accessible with biopsies. Therefore, it is essential to develop a new noninvasive, rapid and accurate imaging method to evaluate PD-L1 expression levels in clinical practice.

With the continuous development of artificial intelligence, radiomics, as a noninvasive imaging tool, plays a nonnegligible role in the diagnosis and clinical management of cancer [12]. Radiomics can extract numerous quantitative features from medical images with high throughput and apply automatic or semiautomatic analysis methods to transform imaging data into mineable data. In recent years, researchers have demonstrated that fluorodeoxyglucose ([^18^F]FDG) positron emission tomography/computed tomography (PET/CT) radiomics can macroscopically predict the PFS or OS of NSCLC patients. With the development of radiomics in the field of molecular precision medicine, epidermal growth factor receptor (EGFR) gene mutation status and lymph node metastasis in NSCLC patients can also be well predicted [13–15]. In addition, some studies have demonstrated that several PET/CT-derived textural features can provide relevant supplementary information for determining the expression status of PD-L1 in patients with head and neck squamous cell carcinoma [16]. In this study, to evaluate the predictive power of radiomics based on [^18^F]FDG PET/CT imaging for PD-L1 expression in patients with NSCLC, we constructed and validated a radiomics model, clinical model and combined model and further developed a nomogram based on the optimal predictive model to predict PD-L1 expression in patients with NSCLC by using [^18^F]FDG PET/CT imaging radiomics.

## Materials and methods

### Patient selection

This study retrospectively analyzed 334 patients with NSCLC who underwent [^18^F]FDG PET/CT examination before treatment at the Fourth Hospital of Hebei Medical University from September 2016 to July 2021. The study population included 203 men and 131 women, aged from 15 to 87 years old with an average age of 62.1 ± 8.9 years. The enrolled patients met the following criteria: (1) non-small cell lung cancer confirmed by operation or puncture biopsy pathology, (2) [^18^F]FDG PET/CT examination was performed before operation or biopsy, (3) PD-L1 expression levels detected by IHC, and (4) no history of other malignant tumors. The exclusion criteria were (1) received antitumor treatment before PET/CT scan and IHC test, (2) the diameter of the target lesion was less than 1 cm and (3) no [^18^F]FDG uptake was found in the lesions on PET/CT images. The clinical data and imaging data of each patient were collected. Clinical data mainly included sex, age, smoking history, clinical stage and pathological type. These patients were randomly divided into a training group (*n* = 233) and a validation group (*n* = 101) at a ratio of 7:3. This retrospective analysis was approved by the Institutional Review Board of the Fourth Hospital of Hebei Medical University (No. 2019MEC031).

### Detection of PD-L1 expression status

In this study, IHC was used to detect PD-L1 expression levels. Histological samples for PD-L1 detection were obtained by surgical resection or puncture biopsy. Studies have shown no significant difference in the feasibility of PD-L1 immunohistochemistry on small biopsy specimens compared with specimens obtained by surgical resection [17]. The PD-L1 detection kit was 22c3 pharmDx (Dako Company). The positive standard was defined as ≥ 1% tumor cell staining [18]. The specimens were fixed with 10% formalin solution, in which the biopsy specimens were fixed for 5 h and the surgical specimens were fixed for at least 24–72 h. The PD-L1 score was read in a double-blinded manner by two different pathologists. When the results were inconsistent with the previous research results, further research and analysis were performed. The latest version of the National Comprehensive Cancer Network (NCCN) has confirmed that immune checkpoint inhibitor therapy is effective in the second-line treatment of NSCLC patients with PD-L1 expression levels ≥ 1%. Moreover, the Food and Drug Administration (FDA) has approved the PD-1 inhibitor pembrolizumab alone as front-line single-agent therapy instead of chemotherapy in patients with PD-L1 expression ≥ 1% [19]. Therefore, in this study, the PD-L1 expression level of the enrolled patients was divided into two groups: ≥ 1% and < 1%. A PD-L1 expression level ≥ 1% was defined as the positive group, and a PD-L1 expression level < 1% was defined as the negative group.

### PET/CT image acquisition

All patients underwent [^18^F]FDG PET/CT before surgery or biopsy. A PHILIPS GEMINI GXL16 PET/CT scanner and PHILIPS VEREOS PET/CT scanner were used. Patients fasted for at least 6 h before injection with 3.70–5.55 MBq/kg [^18^F]FDG, and PET/CT acquisition was performed 60 ± 5 min after the injection. The fasting blood glucose concentration was controlled below 11.1 mmol/L. The body scanning range was from the skull base to the upper femur. The CT data were used to correct the attenuation of the PET image, and the corrected PET image was fused with the CT image.

### Image segmentation and radiomics feature extraction

LIFEx 7.0.0 software was used to extract the radiomics features of PET/CT images in the region of interest (ROI) of lung lesions. The PET/CT image of the patient in the Digital Imaging and Communications in Medicine (DICOM) format was imported into the software. Two experienced nuclear medicine doctors used three-dimensional (3D) drawing tools to draw the ROI layer by layer on the cross section of the image and took 40% of the maximum standardized uptake value (SUV_max_) as the optimization threshold. The spatial resampling interval of all patients with PET and CT images was 1 mm in the X, Y and Z axes. Intensity discretization for CT data was processed by decreasing the continuous scale to 400 bins with absolute scale bounds between − 1000 and 3000 Hounsfield units (HU), whereas that of PET data was performed with 64 bins between 0 and 25. Based on the above, LIFEx software automatically extracted and calculated 124 radiomics features, including 63 PET features and 61 CT features, which are provided in Additional file 1: Table S1. The features extracted in this study can be divided into three types: shape features, first-order statistical features and second-order statistical features.

### Radiomics feature screening and model building

In our study, feature selection was performed in the training group. We first selected the features with significant differences between the PD-L1-positive group and the PD-L1-negative group using the Mann‒Whitney U test and obtained a total of 75 features with a *P* value < 0.05. Then, to avoid overfitting phenomena, the least absolute shrinkage and selection operator (LASSO) algorithm and tenfold cross-validation were used to further screen the optimal radiomics features from the 75 radiomics features and construct the radiomics model by logistic regression. The radiomics score (Rad-score) of each patient was calculated using a logistic regression formula. For the LASSO algorithm, tenfold cross-validation was used to select the best *λ*. The optimal clinical variables with significance based on different expression statuses of PD-L1 were selected to construct the clinical model. The predictive performance of the model was tested in the validation group. Receiver operating characteristic (ROC) curves and the area under the ROC curve (AUC) were used to evaluate the predictive performance of the three models in the training group and the validation group. The Rad-score and the optimal clinical variables were combined to establish a multivariate logistic regression model (the combined model) and to develop a nomogram. Moreover, the predictive probability of the three models for different expression statuses of PD-L1 in each patient was analyzed, and the calibration curve was drawn by comparing the predictive probability with the actual probability. The calibration curve was used to evaluate the performance of the nomogram, which was verified using the Hosmer‒Lemeshow test.

### Statistical method

Statistical analyses were performed with SPSS statistics for Windows (version 26.0, IBM) and R software (version 4.1.2).

Clinical variables included continuous variables (age) and categorical variables (sex, smoking history, pathological type and clinical stage). Quantitative data conforming to a normal distribution are expressed in $\overline{x} \pm s$, and quantitative data that do not conform to a normal distribution are expressed as M (P25, P75). Independent sample t tests, Mann‒Whitney U tests and Chi-square tests were used to identify meaningful clinical variables based on PD-L1 expression status, and then these screened variables were used to establish a clinical model based on multivariate logistic regression analysis. The significant difference between the best radiomics features and their radiomics scores between two different devices was analyzed using the Mann‒Whitney U test. The above analysis was completed using IBM SPSS statistics 26.0 statistical software.

In R software package, "glmnet" package was used to execute LASSO algorithm, "rms" package was used to make nomogram and calibration curve, "waterfalls" package and "ggplot2" package were used to draw waterfall diagram showing Rad-score of each patient in the training group and validation group, and "pRoc" package was used to draw ROC curves and calculate AUC. The above analysis was completed using R 4.1.2 statistical software.

All *P* values < 0.05 were considered statistically significant.

## Results

### Clinical characteristics of patients

Among 334 NSCLC patients, no significant differences in age (*P* = 0.540), sex (*χ*^2^ = 2.604, *P* = 0.107), smoking history (*χ*^2^ = 2.248, *P* = 0.134), pathological type (*χ*^2^ = 1.914, *P* = 0.588) or clinical stage (*χ*^2^ = 2.425, *P*=0.489) were noted between the training group and the validation group. Sex, smoking history and clinical stage were significantly different between the PD-L1-positive and PD-L1-negative groups in both the training and validation groups. PD-L1-positive expression was more commonly observed in male patients with a smoking history and clinical stages II, III and IV, whereas PD-L1-negative expression was more common in nonsmokers and patients in stage I. Age and pathological type were not significantly different between the PD-L1-positive and PD-L1-negative groups in either the training or the validation group. Multivariate logistic regression analysis showed that the clinical stage (odds ratio [OR] 1.579, 95% confidence interval [CI] 0.220–0.703, *P*<0.001) was a significant predictor of different expression statuses of PD-L1 in patients with NSCLC. Table 1 summarizes the basic clinical characteristics of NSCLC patients in the training group and the validation group.

**Table 1:** Basic distribution of clinical characteristics of NSCLC patients in the training and validation groups

Characteristic	Training group (*n* = 233)		*P*	Validation group (*n* = 101)		*P*
	PD-L1 negative (*n* = 125)	PD-L1 positive (*n* = 108)		PD-L1 negative (*n* = 47)	PD-L1 positive (*n* = 54)	
Age (mean ± SD) (years)	62.24 ± 9.10	61.89 ± 9.18	0.504	62.49 ± 8.17	61.54 ± 8.43	0.506
Sex			0.048			0.016
Male	65 (52.00%)	70 (64.81%)		26 (55.32%)	42 (77.78%)	
Female	60 (48.00%)	38 (35.19%)		21 (44.68%)	12 (22.22%)	
Smoking history			0.023			0.044
Never	73 (58.40%)	47 (43.52%)		25 (53.19)	18 (33.33%)	
Current or ever	52 (41.60%)	61 (56.48%)		22 (46.81%)	36 (66.67%)	
Pathological type			0.051			0.165
Adenocarcinoma	10 (84.00%)	77 (71.30%)		39 (82.98%)	37 (68.52%)	
Squamous carcinoma	16 (12.80%)	24 (22.22%)		6 (12.76%)	13 (24.07%)	
Adenosquamous carcinoma	1 (0.80%)	5 (4.63%)		1 (2.13%)	4 (7.41%)	
Large cell carcinoma	3 (2.40%)	2 (1.85%)		1 (2.13%)	0 (0%)	
Pathological stage			0.001			0.006
I	78 (62.40%)	42 (38.89%)		30 (63.83%)	17 (31.48)	
II	16 (12.80%)	17 (15.74%)		6 (12.77%)	10 (18.52%)	
III	22 (17.60%)	25 (23.15%)		6 (12.77%)	21 (38.89%)	
IV	9 (7.20%)	24 (22.22%)		5 (10.64%)	6 (11.11%)	

### Feature extraction and selection

In our study, the features described met the definition described by the Imaging Biomarker Standardization Initiative (IBSI) [20]. The IBSI reporting guidelines are provided in Additional file 2: File 1. There were 50 first-order statistical features, 10 shape features and 64 s-order statistical features, including 14 features of the gray level cooccurrence matrix (GLCM), 22 features of the gray level run length matrix (GLRLM), 22 features of the gray level zone length matrix (GLZLM), and six features of the neighborhood gray level difference matrix (NGLDM).

**Fig. 1:**
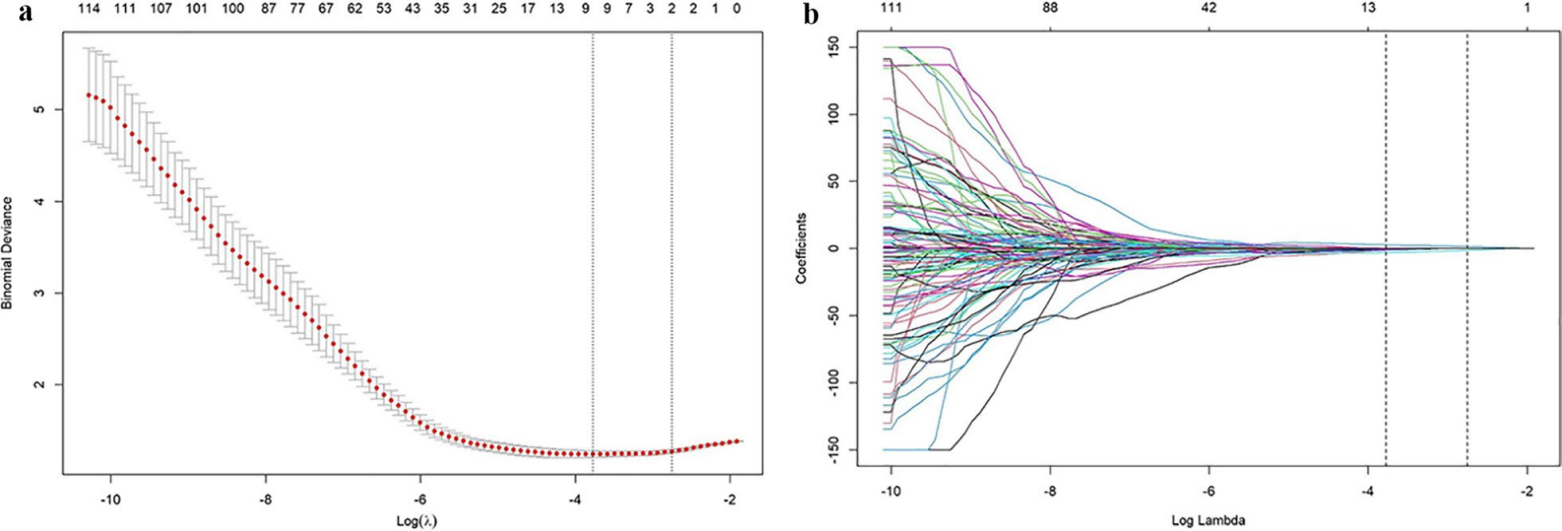
The LASSO algorithm and tenfold cross-validation were used to extract the optimal subgroup of radiomic features. **a** The left vertical line indicates the value of the characteristic parameters corresponding to the minimum of *λ* in cross-validation, and the vertical line on the right represents the parameter value of the more simplified model *λ* within a standard error. When the value of *λ* increased to -2.752, it corresponded to the optimal number of radiomic features. **b** The penalty diagram of the characteristic coefficient of radiomics

The LASSO algorithm and tenfold cross-validation were used to extract the optimal subset of radiomics features. Eventually, two optimal features were selected, including a PET feature and a CT feature: Gray Level Run Length Matrix (GLRLM)_Run Percentage (RP) and SHAPE_Sphericity, respectively. The process of LASSO algorithm filtering features is shown in Fig. 1.

### Model construction

Based on the two optimal features selected above, the Rad-score of each patient was calculated using the following formula. Thus, a radiomics model for predicting different expression statuses of PD-L1 in patients with NSCLC was constructed:

$${\text{Rad{-}score}} = - 1.199 + 3.514*{\text{GLRLM}}\_{\text{RP}} - 1.954*{\text{SHAPE}}\_{\text{Sphericity}}.$$

**Fig. 2:**
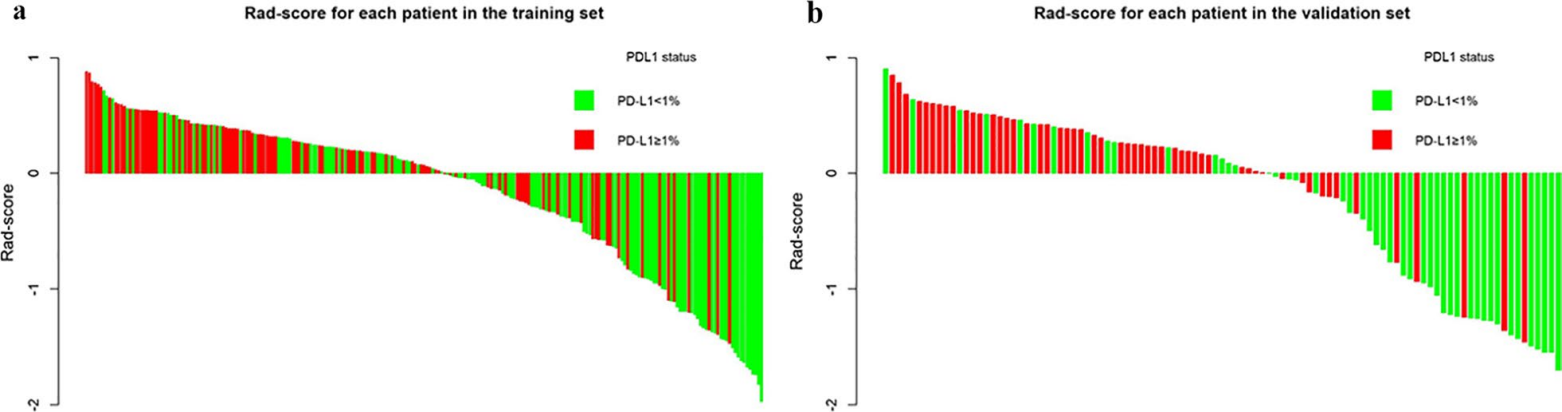
Rad-score of patients in both the training and validation groups

The median and the interquartile range for the 2 selected radiomics features and the calculated Rad-scores are shown in Table 2. In the training group and validation group, the Rad-score was significantly different between the PD-L1-negative group and the PD-L1-positive group (*P* < 0.001). In the training group, the median Rad-score was 0.239 in the PD-L1-positive group and − 0.192 in the PD-L1-negative group. In the validation group, the median Rad-score was 0.254 in the PD-L1-positive group and − 0.493 in the PD-L1-negative group. PD-L1-positive patients had a higher Rad-score than PD-L1-negative patients. The Rad-score for each patient in the two groups is displayed in the form of a bar graph in Fig. 2.

**Table 2:** Comparison of 2 radiomic features and Rad-score between PD-L1 positive and PD-L1 negative group

Features	Training group (*n* = 233)		*P*	Validation group (*n* = 101)		*P*
	PD-L1 negative (*n* = 125)	PD-L1 positive (*n* = 108)		PD-L1 negative (*n* = 47)	PD-L1 positive (*n* = 54)	
Rad-score	− 0.192 (− 0.922, 0.218)	0.239 (− 0.133, 0.424)	< 0.001	− 0.493 (− 1.241, 0.140)	0.254 (0.024, 0.488)	< 0.001
PET feature						
GLRLM_RP	0.660 (0.424, 0.792)	0.786 (0.678, 0.837)	< 0.001	0.615 (0.352, 0.784)	0.791 (0.728, 0.851)	< 0.001
CT feature						
SHAPE_Sphericity	0.686 (0.645, 0.735)	0.682 (0.607, 0.720)	< 0.001	0.675 (0.609, 0.721)	0.677 (0.630, 0.713)	< 0.001

In our study, we found that clinical stage (OR 1.579, 95% CI 0.220–0.703, *P* < 0.001) was a significant predictor of different PD-L1 expression statuses. Therefore, the clinical model was constructed with this factor, and the following formula was obtained:

$${\text{Clinical\;score}} = - 1.157 + 0.457*{\text{clinical\;stage}}$$

We combined the radiomics model and clinical model, and the following formula was obtained:

$${\text{Complex\;score}} = 0.1518 + 0.719*{\text{clinical\;stage}} + 1.093*{\text{Rad\;score}}$$

### Predictive performance of the radiomics model, clinical model and combined model

**Fig. 3:**
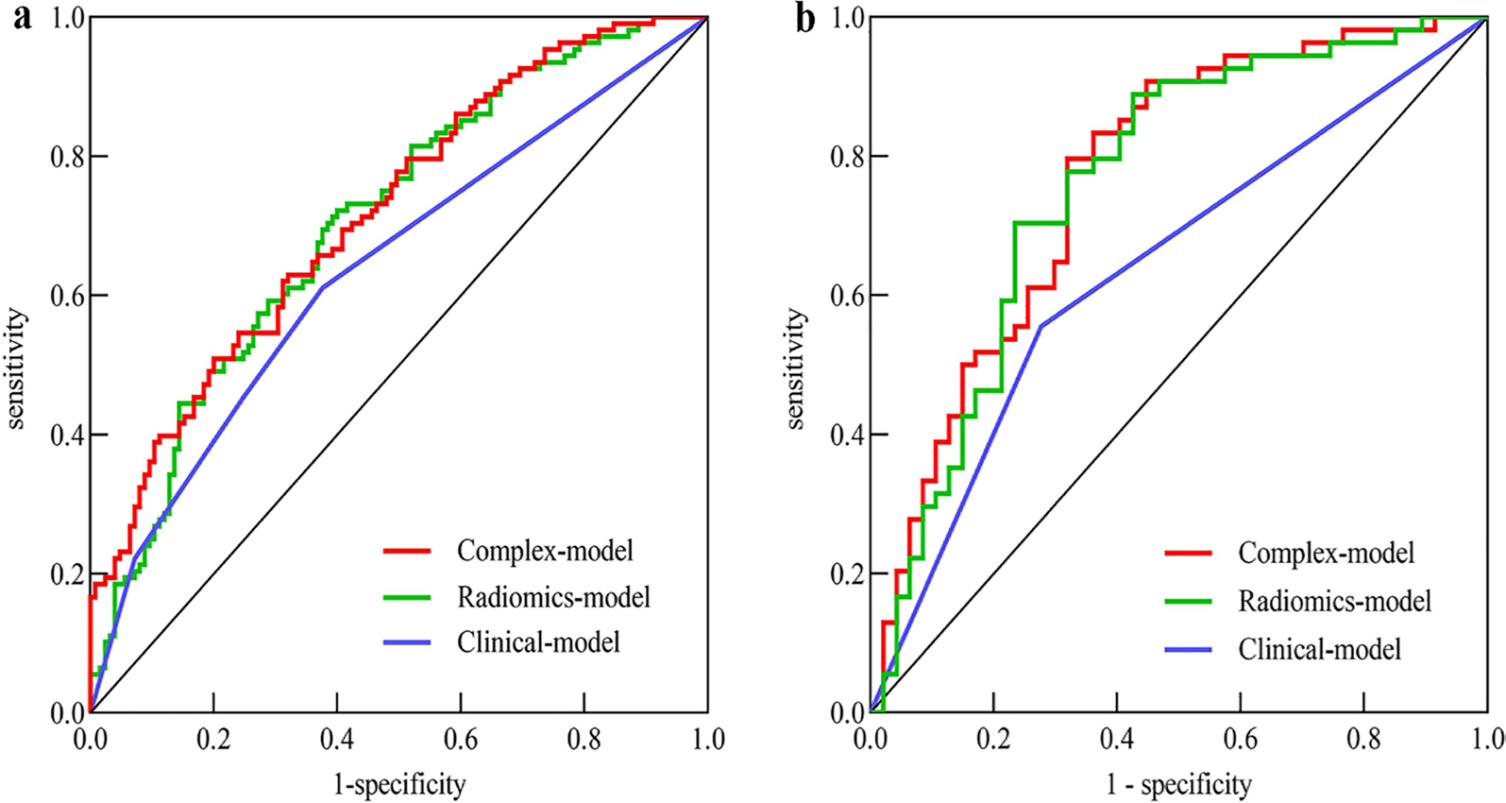
ROC curves for the radiomics model, the clinical model, and the combined model in predicting different expression statuses of PD-L1. **a** The ROC curve of the training group. **b** The ROC curve of the validation group

To evaluate the performance of radiomics in predicting PD-L1 expression status in patients with NSCLC, we constructed and validated a radiomics model, clinical model and their combination (Fig. 3). In both the training and validation groups, the AUC values of the clinical model used to predict the different expression statuses of PD-L1 in NSCLC patients were 0.638 (95% CI 0.572–0.705) and 0.640 (95% CI 0.547–0.733), respectively, and the AUC values of the radiomics model were 0.706 (95% CI 0.640–0.772) and 0.761 (95% CI 0.664–0.860), respectively. In addition, the AUC values of the combined model based on the radiomics signature score (Rad-score) and clinical variables in both the training and validation groups were 0.718 (95% CI 0.653–0.783) and 0.769 (95% CI 0.675–0.863), respectively.

The DeLong test showed that the AUC values between the clinical model and the combined model were significantly different in both the training and validation groups (*P* = 0.004 and 0.007, respectively), and the AUC value between the radiomics model and the combined model exhibited no significant differences in either the training or the validation group (*P* = 0.505 and 0.629, respectively). The predictive abilities of the three models, including sensitivity, specificity, and accuracy, are shown in Table 3.

**Table 3:** Predictive performance of the three models in the training and validation groups

Models	Training group				Validation group			
	AUC (95% CI)	Sensitivity (%)	Specificity (%)	Accuracy (%)	AUC (95% CI)	Sensitivity (%)	Specificity (%)	Accuracy (%)
Clinical model	0.638 (0.572–0.705)	56.48	67.20	62.23	0.640 (0.547–0.733)	64.81	65.96	65.35
Radiomics model	0.706 (0.640–0.772)	62.96	71.20	67.38	0.761 (0.664–0.860)	68.52	72.34	70.30
Complex model	0.718 (0.653–0.783)	61.11	68.80	65.24	0.769 (0.675–0.863)	68.52	68.09	68.32

### Individualized nomogram construction and validation

**Fig. 4:**
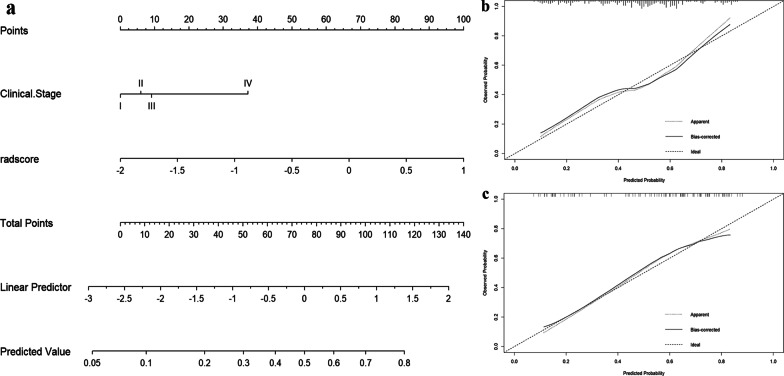
Nomogram development and performance. **a** Nomogram based on the combined model. **b** Calibration curve of the nomogram in the training group. **c** Calibration curve of the nomogram in the validation group

The combined model based on the Rad-score and clinical variables (clinical stage) exhibited relatively good predictive performance; therefore, an individualized nomogram based on the combined model was developed and constructed in the training group, which can more intuitively show the proportion of prediction results and each influencing factor (Fig. 4a). Figures 4b and 4b show the calibration curves of the nomogram. The results revealed good consistency between the predicted probability and the actual predicted probability in the training and validation groups. The Hosmer‒Lemeshow test confirmed that there was good consistency between the predicted probability and the actual predicted probability in the training group (*χ*^2^ = 1.463, *P* = 0.481) and the validation group (*χ*^2^ = 1.563, *P* = 0.458).

### Comparison of equipment differences

In the training group and the validation group, the significant differences in the two radiomics feature parameters and Rad-score of the cases examined by two different devices (PHILIPS GEMINI GXL16 PET/CT scanner and PHILIPS VEREOS PET/CT scanner) were compared. No significant differences were noted (*P* > 0.05).

## Discussion

In recent years, immunotherapy with PD-1/PD-L1 checkpoint inhibitors has greatly changed the prognosis of NSCLC patients. Several studies have shown that patients with elevated PD-L1 expression will likely benefit from PD-1/PD-L1 inhibitors [21]. At present, some scholars have concluded that the expression of PD-L1 is one of the most widely used biomarkers [22]. Therefore, the PD-L1 expression level plays an important role in guiding immunotherapy in NSCLC patients. To predict the response of NSCLC patients to immunotherapy, it is of great significance to select the most meaningful biomarkers to predict the effect of NSCLC immunotherapy.

In this study, we established and validated a predictive model of PD-L1 expression status in NSCLC patients before treatment based on [^18^F]FDG PET/CT radiomics features. From the above results, it can be seen that the radiomics model exhibits good performance in predicting the different expression statuses of PD-L1 in NSCLC patients, and its predictive performance is similar to that of the combined model. This study shows that the radiomics model based on the two best features obtained from [^18^F]FDG PET/CT images and the combined model can better predict the expression status of PD-L1 in NSCLC patients, and its predictive performance in the validation group is better than that in the training group.

PET can provide tumor metabolic information, glucose metabolism and hypoxia, angiogenesis and other related information. Therefore, PET/CT imaging not only accurately locates the focus but also provides metabolic information on the tumor. Moreover, radiomics analysis can reflect the potential spatial variation of tumors, the heterogeneity of internal voxel intensity and tracer uptake and can better characterize tumors. Unlike the clinical model, the radiomics model used to predict the expression status of PD-L1 in NSCLC patients is noninvasive and can reflect the changes in PD-L1 in patient tumors in real time. Moreover, radiomics can perform functional imaging of tumors at the molecular and cellular levels, reflecting the overall expression of PD-L1 in tumor lesions. This feature also represents an advantage of the radiomics model compared with the clinical model.

Among the two optimal features screened in our study, SHAPE_Sphericity is a basic CT feature. Shape-based features describe the 2D or 3D size and size of the region of interest and can quantitatively describe the geometric features of the region of interest, which is independent of the gray level intensity distribution of the region of interest [23]. Among them, SHAPE_Sphericity can describe the roundness of the shape of tumor areas related to sphericity. Some studies have found that the sphericity value has the ability to predict the proportion of micropapilla in lung adenocarcinoma, and the prognosis of patients with lung adenocarcinoma will deteriorate with an increase in the proportion of micropapilla in tumors [24]. Bracci et al. [25] studied its ability to predict PD-L1 expression in NSCLC patients by extracting texture features from CT images of 72 NSCLC patients. Their results showed that in the training group, patients with PD-L1 < 1% had higher sphericity than patients with PD-L1 ≥ 1% [sphericity: 0.944 (< 1%) vs. 0.923 (≥ 1%)], which was consistent with the results of our study [in the training group, sphericity: 0.686 (< 1%) vs. 0.682 (≥ 1%)].

Another feature is GLRLM_RP. The gray level run length matrix (GLRLM) displays the length of continuous voxels with the same intensity in the pregroup direction in the image. Among them, the run percentage (RP) measures the roughness of texture features by taking the ratio of run number to voxel number in the ROI. This feature belongs to the second-order feature (first proposed by Haralick). The second-order feature is based on the joint probability distribution of paired voxels and describes the spatial arrangement of patterns; that is, it is related to the uniformity and nonuniformity of images. In our study, the images of the PD-L1-positive group showed more heterogeneity than those of the PD-L1-negative group.

The PET/CT image data in our study were obtained from two scanners with different parameters. In clinical practice, the image data of patients are usually obtained from a scanner with different scanning parameters [26]. In our study, no significant difference was noted between the two best radiomics features extracted from two different devices and their Rad-scores, which is consistent with the results obtained by Hu et al. [27] There was no significant difference between the nine radiomics features extracted from two different devices and the Rad-scores.

In recent years, the application of radiomics in NSCLC has become increasingly extensive, and the research used to predict the expression of PD-L1 is also increasing. In the research of Sun et al. [28], by combining the radiomics model based on CT image features with clinicopathological features, the best model for predicting the expression status of PD-L1 in NSCLC patients was obtained. The AUC values in the training group and the validation group were 0.829 and 0.848, respectively. However, the AUC values of the combined model of [^18^F]FDG PET/CT radiomics to predict PD-L1 expression in NSCLC patients obtained in our study were 0.718 and 0.769 in the training group and the validation group, respectively. The reason may lie in the different stratification of PD-L1 expression levels. In our study, PD-L1 was divided into ≥ 1% and < 1%, whereas Sun et al. divided PD-L1 into ≥ 50% and < 50%. In future studies, we will perform a more detailed stratification of PD-L1 expression and strive to provide more accurate and detailed services for clinical practice. Furthermore, considering that the AUCs of 0.7–0.8 are generally considered acceptable and values from 0.8 to 0.9 are considered excellent [29], these results demonstrate that the model we have established is clinically useful. Similarly, the research of Yoon et al. [30] also confirmed this view. Specifically, the combination of clinical variables and radiomics models based on CT images would be conducive to the noninvasive evaluation of PD-L1 expression in NSCLC patients. However, PET radiomics features were not included in either study. In recent years, research on [^18^F]FDG PET/CT radiomics in predicting PD-L1 expression in tumor patients has gradually increased. Li et al. [31] segmented the [^18^F]FDG PET/CT imaging of 255 NSCLC patients and extracted the radiomics features, revealing that PD-L1 expression was related to the histopathological type of NSCLC patients but not to the clinical stage. This finding is contrary to the results obtained in our study. Two possible explanations are proposed. First, Li's study only included patients with squamous cell carcinoma and adenocarcinoma, whereas our study also included patients with adenosquamous carcinoma and large cell carcinoma. Second, Li's study compared the difference in PD-L1 expression between patients with clinical stages I-II and III-IV, whereas our study compared the difference in PD-L1 expression between patients with stages I-IV in more detail.

Jiang et al. [32] established and validated the predictive model of PD-L1 expression in patients with NSCLC based on PET, CT and PET/CT images by segmenting and extracting features from PET/CT images of 399 patients with stage I-IV NSCLC. The researchers concluded that the radiomics features derived from CT images can better predict the expression status of specific types of PD-L1 in NSCLC patients than those derived from PET images. However, the study did not combine risk factors related to the patient's clinical characteristics. Moreover, in this study, the researchers obtained the image ROI by sketching the ROI on the CT image first and then corresponding it to the PET image, which may lead to inaccurate image matching and affect image segmentation. Compared with this study, our study not only combined the clinical risk factors for patients but also directly outlined the ROI on the fused PET/CT images.

In addition, in a recent study, Mu et al. [33] developed a deep learning score predictive model (PDL1-DLS) based on [^18^F]FDG PET/CT to evaluate the expression status of PD-L1 in patients with NSCLC and a deep learning score predictive model (EGFR-DLS) to evaluate the mutation status of epidermal growth factor receptor (EGFR) in patients with NSCLC, which can help clinicians choose more suitable treatment modes of NSCLC patients in the treatment of ICI and targeted treatment represented by EGFR tyrosine kinase inhibitors (TKIs).

Some limitations in our study should be noted. First, this study was a single-center retrospective study. In the future, a multicenter prospective study will be encouraged. This design can reduce selection bias and improve the stability and repeatability of the predictive model. Second, we can try a variety of machine learning methods to establish predictive models to find the best modeling method.

In conclusion, this radiomics study based on [^18^F]FDG PET/CT radiomics features before treatment showed that the prediction model could predict PD-L1 expression status and provide a convenient, noninvasive and relatively accurate method for clinicians to identify patients who can benefit from anti-PD-L1 immunotherapy in NSCLC to guide the clinical immunotherapy of patients with NSCLC.

## Supplementary Information

**Additional file 1: Table S1.** Title of data: PET and CT radiomics features extracted from LIFEx software. Description of data: This Table S1 describes the extracted radiomics feature parameters.

**Additional file 2: File 1.** Title of data: Image biomarker standardization initiative. Description of data: This file describes the standardization of image segmentation and feature extraction.

## Data Availability

Not applicable.

## Abbreviations

ICI: Immune checkpoint inhibitor
[^18^F]FDG: [^18^F]-Fluorodeoxyglucose
PET/CT: Positron emission tomography/computed tomography
NSCLC: Non-small cell lung cancer
ROI: Region of interest
NCCN: National Comprehensive Cancer Network
LASSO: Least Absolute Shrinkage and Selection Operator
SUV_max_: Maximum of standardized uptake value
PD-1: Programmed death-1
PD-L1: Programmed death ligand-1
IHC: Immunohistochemistry
GLCM: Gray level co-occurrence matrix
GLRLM: Gray level run length matrix
GLZLM: Gray level zone length matrix
NGLDM: Neighborhood gray level difference matrix
PFS: Progression-free survival
OS: Overall survival
ROC: Receiver operating characteristic
AUC: Area under ROC curve

## References

[1] Sung H, Ferlay J, Siegel RL, Laversanne M, Soerjomataram I, Jemal A (2021). Global cancer statistics 2020: GLOBOCAN estimates of incidence and mortality worldwide for 36 cancers in 185 countries. CA Cancer J Clin.

[2] Deslypere G, Gullentops D, Wauters E, Vansteenkiste J (2018). Immunotherapy in non-metastatic non-small cell lung cancer: Can the benefits of stage IV therapy be translated into earlier stages?. Ther Adv Med Oncol.

[3] Hayashi H, Nakagawa K (2020). Combination therapy with PD-1 or PD-L1 inhibitors for cancer. Int J Clin Oncol.

[4] Rizvi NA, Mazières J, Planchard D, Stinchcombe TE, Dy GK, Antonia SJ (2015). Activity and safety of nivolumab, an anti-PD-1 immune checkpoint inhibitor, for patients with advanced, refractory squamous non-small-cell lung cancer (CheckMate 063): a phase 2, single-arm trial. Lancet Oncol.

[5] Reck M, Rodriguez-Abreu D, Robinson AG, Hui R, Csoszi T, Fulop A (2016). Pembrolizumab versus chemotherapy for PD-L1-positive non-small-cell lung cancer. N Engl J Med.

[6] Herbst RS, Baas P, Kim DW, Felip E, Pérez-Gracia JL, Han JY (2016). Pembrolizumab versus docetaxel for previously treated, PD-L1-positive, advanced non-small-cell lung cancer (KEYNOTE-010): a randomised controlled trial. Lancet.

[7] Rittmeyer A, Barlesi F, Waterkamp D, Park K, Ciardiello F, von Pawel J (2017). Atezolizumab versus docetaxel in patients with previously treated non-small-cell lung cancer (OAK): a phase 3, open-label, multicentre randomised controlled trial. Lancet.

[8] Doroshow DB, Sanmamed MF, Hastings K, Politi K, Rimm DL, Chen L (2019). Immunotherapy in non-small cell lung cancer: facts and hopes. Clin Cancer Res.

[9] Brahmer J, Reckamp KL, Baas P, Crino L, Eberhardt WE, Poddubskaya E (2015). Nivolumab versus docetaxel in advanced squamous-cell non-small-cell lung cancer. N Engl J Med.

[10] Tejerina E, Garca Tobar L, Echeveste JI, de Andrea CE, Vigliar E, Lozano MD (2021). PD-L1 in cytological samples: a review and a practical approach. Front Med.

[11] Wu X, Huang Y, Zhao Q, Wang L, Song X, Li Y (2020). PD-L1 expression correlation with metabolic parameters of FDG PET/CT and clinicopathological characteristics in non-small cell lung cancer. EJNMMI Res.

[12] De la Pinta C, Barrios-Campo N, Sevillano D (2020). Radiomics in lung cancer for oncologists. J Clin Transl Res..

[13] Wang H, Zhou Z, Li Y, Chen Z, Lu P, Wang W (2017). Comparison of machine learning methods for classifying mediastinal lymph node metastasis of non-small cell lung cancer from (18)F-FDG PET/CT images. EJNMMI Res.

[14] Yang L, Yang J, Zhou X, Huang L, Zhao W, Wang T (2019). Development of a radiomics nomogram based on the 2D and 3D CT features to predict the survival of non-small cell lung cancer patients. Eur Radiol.

[15] Zhang J, Zhao X, Zhao Y, Zhang J, Zhang Z, Wang J (2020). Value of pre-therapy (18)F-FDG PET/CT radiomics in predicting EGFR mutation status in patients with non-small cell lung cancer. Eur J Nucl Med Mol Imaging.

[16] Chen RY, Lin YC, Shen WC, Hsieh TC, Yen KY, Chen SW (2018). Associations of tumor PD-1 ligands, immunohistochemical studies, and textural features in (18)F-FDG PET in squamous cell carcinoma of the head and neck. Sci Rep.

[17] Heymann JJ, Bulman WA, Swinarski D, Pagan CA, Crapanzano JP, Haghighi M (2017). PD-L1 expression in non-small cell lung carcinoma: comparison among cytology, small biopsy, and surgical resection specimens. Cancer Cytopathol.

[18] Teixido C, Vilarino N, Reyes R, Reguart N (2018). PD-L1 expression testing in non-small cell lung cancer. Ther Adv Med Oncol.

[19] Ullah A, Pulliam S, Karki NR, Khan J, Jogezai S, Sultan S (2022). PD-L1 over-expression varies in different subtypes of lung cancer: Will this affect future therapies?. Clin Pract.

[20] Zwanenburg A, Vallieres M, Abdalah MA, Aerts H, Andrearczyk V, Apte A (2020). The image biomarker standardization initiative: standardized quantitative radiomics for high-throughput image-based phenotyping. Radiology.

[21] Sacher AG, Gandhi L (2016). Biomarkers for the clinical use of PD-1/PD-L1 inhibitors in non-small-cell lung cancer: a review. JAMA Oncol.

[22] Passiglia F, Bronte G, Bazan V, Natoli C, Rizzo S, Galvano A (2016). PD-L1 expression as predictive biomarker in patients with NSCLC: a pooled analysis. Oncotarget.

[23] Scapicchio C, Gabelloni M, Barucci A, Cioni D, Saba L, Neri E (2021). A deep look into radiomics. Radiol Med.

[24] Song SH, Park H, Lee G, Lee HY, Sohn I, Kim HS (2017). Imaging phenotyping using radiomics to predict micropapillary pattern within lung adenocarcinoma. J Thorac Oncol.

[25] Bracci S, Dolciami M, Trobiani C, Izzo A, Pernazza A, D'Amati G (2021). Quantitative CT texture analysis in predicting PD-L1 expression in locally advanced or metastatic NSCLC patients. Radiol Med.

[26] Kim YJ, Lee HJ, Kim KG, Lee SH (2019). The effect of CT scan parameters on the measurement of CT radiomic features: a lung nodule phantom study. Comput Math Methods Med.

[27] Hu Y, Zhao X, Zhang J, Han J, Dai M (2021). Value of (18)F-FDG PET/CT radiomic features to distinguish solitary lung adenocarcinoma from tuberculosis. Eur J Nucl Med Mol Imaging.

[28] Sun Z, Hu S, Ge Y, Wang J, Duan S, Song J (2020). Radiomics study for predicting the expression of PD-L1 in non-small cell lung cancer based on CT images and clinicopathologic features. J Xray Sci Technol.

[29] Mandrekar JN (2010). Receiver operating characteristic curve in diagnostic test assessment. J Thorac Oncol.

[30] Yoon J, Suh YJ, Han K, Cho H, Lee HJ, Hur J (2020). Utility of CT radiomics for prediction of PD-L1 expression in advanced lung adenocarcinomas. Thorac Cancer.

[31] Li J, Ge S, Sang S, Hu C, Deng S (2021). Evaluation of PD-L1 expression level in patients with non-small cell lung cancer by (18)F-FDG PET/CT radiomics and clinicopathological characteristics. Front Oncol.

[32] Jiang M, Sun D, Guo Y, Guo Y, Xiao J, Wang L (2020). Assessing PD-L1 expression level by radiomic features from PET/CT in nonsmall cell lung cancer patients: an initial result. Acad Radiol.

[33] Mu W, Jiang L, Zhang J, Shi Y, Gray JE, Tunali I (2020). Non-invasive decision support for NSCLC treatment using PET/CT radiomics. Nat Commun.

