# Supplementary material for: Predicting PD-L1 expression status in patients with non-small cell lung cancer using [18F]FDG PET/CT radiomics
Source: EJNMMI Res. 2023 Jan 22;13:4. doi: 10.1186/s13550-023-00956-9 (PMC9868196; doi:10.1186/s13550-023-00956-9)
Supplement: Supplementary file 1 — Additional file 1: Table S1. Title of data: PET and CT radiomics features extracted from LIFEx software. Description of data: This Table S1 describes the extracted radiomics feature parameters. [file 13550_2023_956_MOESM1_ESM.docx]

**Tabel 1**  PET and CT radiomics features extracted from LIFEx software

| Feature set | \| **Basic features** \| \| \| --- \| --- \| \| **Basic features PET** \| **Basic features CT** \| \| CONVENTIONAL/DISCRETIZED  SUVminimum  SUV_mean_  SUV_std_  SUVmaximum  SUV_Q1/Q2/Q3_  SUV_Skewness_  SUV_Kurtosis_  SUV_ExcessKurtosis_  TLG  DISCRETIZED_AUC_CSH \| CONVENTIONAL/DISCRETIZED  HU_Minimum_  HU_Maximum_  HU_Mean_  HU_std_  HU_Q1/Q2/Q3_  HU_Skewness_  HU_Kurtosis_  HU_ExcessKurtosis_  DISCRETIZED_AUC_CSH \| \| **Indices from Histogram** \| \| \| HISTO_Entropy_log10  HISTO_Entropy_log2  HISTO_Energy \| \| \| **Indices from Shape** \| \| \| SHAPE_Sphericity  SHAPE_Compacity  SHAPE_Volume (mL)  SHAPE_Volume (voxels)  SHAPE_SurfaceArea \| \| \| **Texture Features** \| \| \| **Grey Level Co-occurrence Matrix (GLCM)** \| \| \| GLCM_Homogeneity  GLCM_Energy  GLCM_Contrast  GLCM_Correlation  GLCM_Entropy_log10  GLCM_Entropy_log2  GLCM_Dissimilarity \| \| \| **Grey-Level Run Length Matrix (GLRLM)** \| \| \| GLRLM_SRE (Short-Run Emphasis)  GLRLM_LRE (Long-Run Emphasis)  GLRLM_LGRE (Low Gray-level Run Emphasis )  GLRLM_HGRE (High Gray-level Run Emphasis)  GLRLM_SRLGE (Short-Run Low Gray-level Emphasis)  GLRLM_SRHGE (Short-Run High Gray-level Emphasis)  GLRLM_LRLGE (Long-Run Low Gray-level Emphasis)  GLRLM_LRHGE (Long-Run High Gray-level Emphasis)  GLRLM_GLNUr (Gray-Level Non-Uniformity for run)  GLRLM_RLNU (Run Length Non-Uniformity)  GLRLM_RP (Run Percentage) \| \| \| **Neighborhood Grey-Level Different Matrix (NGLDM)** \| \| \| NGLDM_Coarseness  NGLDM_Contrast  NGLDM_Busyness \| \| \| **Grey-Level Zone Length Matrix (GLZLM)** \| \| \| GLZLM_SZE (Short-Zone Emphasis)  GLZLM_LZE (Long-Zone Emphasis)  GLZLM_LGZE (Low Gray-level Zone Emphasis)  GLZLM_HGZE (High Gray-level Zone Emphasis)  GLZLM_SZLGE (Short-Zone Low Gray-level Emphasis)  GLZLM_SZHGE (Short-Zone High Gray-level Emphasis)  GLZLM_LZLGE (Long-Zone Low Gray-level Emphasis)  GLZLM_LZHGE (Long-Zone High Gray-level Emphasis)  GLZLM_GLNUz (Gray-Level Non-Uniformity for zone)  GLZLM_ZLNU (Zone Length Non-Uniformity)  GLZLM_ZP (Zone Percentage) \| \| |
| --- | --- | --- | --- | --- | --- | --- | --- | --- | --- | --- | --- | --- | --- | --- | --- | --- | --- | --- | --- | --- | --- | --- | --- | --- | --- | --- | --- | --- | --- | --- | --- | --- | --- |

This supplementary table1 describes the extracted radiomics feature parameters.
