# Supplementary material for: Predicting PD-L1 expression status in patients with non-small cell lung cancer using [18F]FDG PET/CT radiomics
Source: EJNMMI Res. 2023 Jan 22;13:4. doi: 10.1186/s13550-023-00956-9 (PMC9868196; doi:10.1186/s13550-023-00956-9)
Supplement: Supplementary file 2 — Additional file 2: File 1. Title of data: Image biomarker standardization initiative. Description of data: This file describes the standardization of image segmentation and feature extraction. [file 13550_2023_956_MOESM2_ESM.pdf]

Image biomarker standardisation initiative

Reference manual

version 1.0 (December 2019)

The image biomarker standardisation initiative

The image biomarker standardisation initiative (IBSI) is an independent international collaboration which works towards standardising the extraction of image biomarkers from acquired imaging for the purpose of high-throughput quantitative image analysis (radiomics). Lack of reproducibility and validation of radiomic studies is considered to be a major challenge for the field. Part of this challenge lies in the scantiness of consensus-based guidelines and definitions for the process of translating acquired imaging into high-throughput image biomarkers. The IBSI therefore seeks to provide standardised image biomarker nomenclature and definitions, a standardised general image processing workflow, tools for verifying radiomics software implementations and reporting guidelines for radiomic studies.

Permanent identifiers

The IBSI uses permanent identifiers for image biomarker definitions and important related concepts such as image processing. These consist of four-character codes and may be used for reference. Please do not use page numbers or section numbers as references, as these are subject to change.

Copyright

This work is a copy-edited version of the final (v10) pre-print version of the IBSI reference manual, which was licensed under the Creative Commons Attribution 4.0 International License (CC-BY). The original version is: Zwanenburg A, Leger S, Vallières M, Löck S, for the Image Biomarker Standardisation Initiative. Image Biomarker Standardisation Initiative. arXiv preprint (2016):1612.07003.

Copyright information regarding the reference data sets may be found on GitHub: https://github.com/theibsi/data_sets

Contents

1 Introduction **1**

2 Image processing **3**

2.1 Data conversion 3
2.2 Post-acquisition processing 5
2.3 Segmentation 5
2.4 Interpolation 6
2.5 Re-segmentation 11
2.6 ROI extraction 13
2.7 Intensity discretisation 13
2.8 Feature calculation 16

3 Image features **17**

3.1 Morphological features 20
3.2 Local intensity features 35
3.3 Intensity-based statistical features 37
3.4 Intensity histogram features 45
3.5 Intensity-volume histogram features 56
3.6 Grey level co-occurrence based features 60
3.7 Grey level run length based features 84
3.8 Grey level size zone based features 98
3.9 Grey level distance zone based features 108
3.10 Neighbourhood grey tone difference based features 118
3.11 Neighbouring grey level dependence based features 124

4 Radiomics reporting guidelines and nomenclature **136**

4.1 Reporting guidelines 136
4.2 Feature nomenclature 142

5 Reference data sets **148**

5.1 Digital phantom . . . 148
5.2 Lung cancer CT image . . . 149

A Digital phantom texture matrices **155**

A.1 Grey level co-occurrence matrix (2D) . . . 155
A.2 Grey level co-occurrence matrix (2D, merged) . . . 156
A.3 Grey level co-occurrence matrix (3D) . . . 157
A.4 Grey level co-occurrence matrix (3D, merged) . . . 159
A.5 Grey level run length matrix (2D) . . . 159
A.6 Grey level run length matrix (2D, merged) . . . 161
A.7 Grey level run length matrix (3D) . . . 161
A.8 Grey level run length matrix (3D, merged) . . . 163
A.9 Grey level size zone matrix (2D) . . . 163
A.10 Grey level size zone matrix (3D) . . . 163
A.11 Grey level distance zone matrix (2D) . . . 164
A.12 Grey level distance zone matrix (3D) . . . 164
A.13 Neighbourhood grey tone difference matrix (2D) . . . 164
A.14 Neighbourhood grey tone difference matrix (3D) . . . 164
A.15 Neighbouring grey level dependence matrix (2D) . . . 165
A.16 Neighbouring grey level dependence matrix (3D) . . . 165

Chapter 1

Introduction

A biomarker is *"a characteristic that is objectively measured and evaluated as an indicator of normal biological processes, pathogenic processes, or pharmacologic responses to a therapeutic intervention"*[7]. Biomarkers may be measured from a wide variety of sources, such as tissue samples, cell plating, and imaging. The latter are often referred to as imaging biomarkers[55]. Imaging biomarkers consist of both qualitative biomarkers, which require expert interpretation, and quantitative biomarkers which are based on mathematical definitions. Calculation of quantitative imaging biomarkers can be automated, which enables high-throughput analyses. We refer to such (high-throughput) quantitative biomarkers as image biomarkers to differentiate them from qualitative imaging biomarkers. Image biomarkers characterise the contents of (regions of) an image, such as *volume* or *mean intensity*. Because of the historically close relationship with the computer vision field, image biomarkers are also referred to as image features. The term *features*, instead of biomarkers, will be used throughout the remainder of the reference manual, as the contents are generally applicable and not limited to life sciences and medicine only.

This work focuses specifically on the (high-throughput) extraction of image biomarkers from acquired, reconstructed and stored imaging. High-throughput quantitative image analysis (radiomics) has shown considerable growth in e.g. cancer research[41], but the scarceness of consensus guidelines and definitions has led to it being described as a "wild frontier"[13]. This reference manual therefore presents an effort to chart a course through part of this frontier by presenting consensus-based recommendations, guidelines, definitions and reference values for image biomarkers and defining a general radiomics image processing scheme. We hope use of this manual will improve reproducibility of radiomic studies.

We opted for a specific focus on the computation of image biomarkers from acquired imaging. Thus, validation of imaging biomarkers, either viewed in a broader framework such as the one presented by O'Connor et al.[55], or within smaller-scope settings such as those presented by Caicedo et al.[13] and by Lambin et al.[41], falls beyond the scope of this work. Notably, the issue of harmonising and standardising (medical) image acquisition and reconstruction is being addressed in a more comprehensive manner by groups such as the Quantitative Imaging Biomarker Alliance[53,68], the Quantitative Imaging Network[17,54], and task groups and committees of the American Association of Physicists in Medicine, the European Association for Nuclear Medicine[11], the European Society of Radiology (ESR)[28], and the European Organisation for Research and Treatment of Cancer (EORTC)[55,86], among others. Where overlap does exists, the reference manual refers to existing recommendations and guidelines.

This reference manual is divided into several chapters that describe processing of acquired and reconstructed (medical) imaging for high-throughput computation of image biomarkers (**Chapter 2**); that define a diverse set of image biomarkers (**Chapter 3**); that describe guidelines for reporting on radiomic studies and provide nomenclature for image biomarkers (**Chapter 4**); and that describe the data sets and image processing configurations used to find reference values for image biomarkers (**Chapter 5**).

Chapter 2

Image processing

Image processing is the sequence of operations required to derive image biomarkers (features) from acquired images. In the context of this work an image is defined as a three-dimensional (3D) stack of two-dimensional (2D) digital image slices. Image slices are stacked along the z-axis. This stack is furthermore assumed to possess the same coordinate system, i.e. image slices are not rotated or translated (in the xy-plane) with regard to each other. Moreover, digital images typically possess a finite resolution. Intensities in an image are thus located at regular intervals, or spacing. In 2D such regular positions are called *pixels*, whereas in 3D the term *voxels* is used. Pixels and voxels are thus represented as the intersections on a regularly spaced grid. Alternatively, pixels and voxels may be represented as rectangles and rectangular cuboids. The centers of the pixels and voxels then coincide with the intersections of the regularly spaced grid. Both representations are used in the document.

Pixels and voxels contain an intensity value for each channel of the image. The number of channels depends on the imaging modality. Most medical imaging generates single-channel images, whereas the number of channels in microscopy may be greater, e.g. due to different stainings. In such multi-channel cases, features may be extracted for each separate channel, a subset of channels, or alternatively, channels may be combined and converted to a single-channel representation. In the remainder of the document we consider an image as if it only possesses a single channel.

The intensity of a pixel or voxel is also called a *grey level* or *grey tone*, particularly in single-channel images. Though practically there is no difference, the terms *grey level* or *grey tone* are more commonly used to refer to discrete intensities, including discretised intensities.

Image processing may be conducted using a wide variety of schemes. We therefore designed a general image processing scheme for image feature calculation based on schemes used within scientific literature [38]. The image processing scheme is shown in figure 2.1. The processing steps referenced in the figure are described in detail within this chapter.

2.1 Data conversion 23XZ

Some imaging modalities require conversion of raw image data into a more meaningful presentation, e.g. standardised uptake values (SUV) [11]. This is performed during the data conversion step. Assessment of data conversion methods falls outside the scope of the

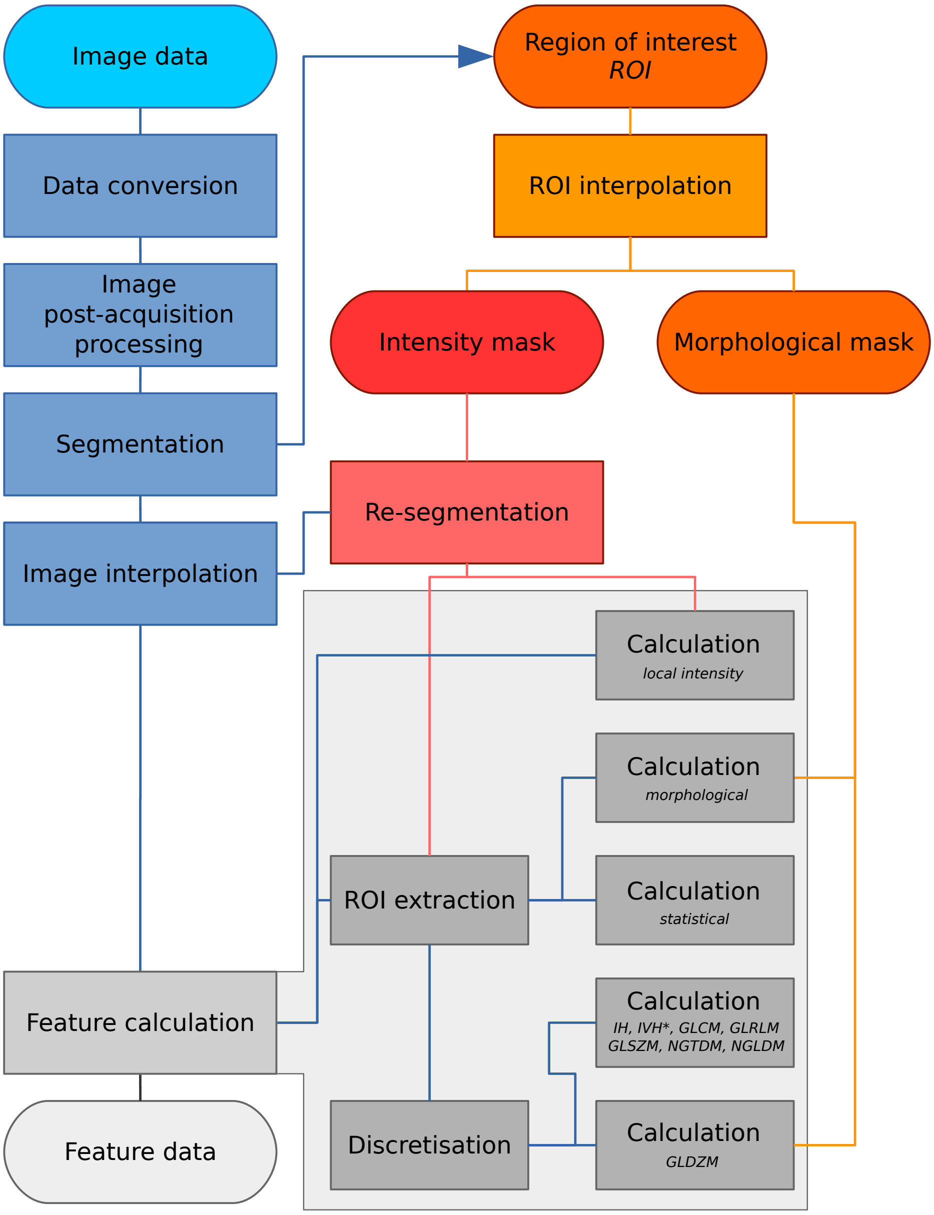

Figure 2.1 | Image processing scheme for image feature calculation. Depending on the specific imaging modality and purpose, some steps may be omitted. The region of interest (ROI) is explicitly split into two masks, namely an intensity and morphological mask, after interpolation to the same grid as the interpolated image. Feature calculation is expanded to show the different feature families with specific pre-processing. IH: intensity histogram; IVH: intensity-volume histogram; GLCM: grey level cooccurrence matrix; GLRLM: grey level run length matrix; GLSZM: grey level size zone matrix; NGTDM: neighbourhood grey tone difference matrix; NGLDM: Neighbouring grey level dependence matrix; GLDZM: grey level distance zone matrix; *Discretisation of IVH differs from IH and texture features, see section 3.5.

current work.

2.2 Image post-acquisition processing PCDE

Images are post-processed to enhance image quality. For instance, magnetic resonance imaging (MRI) contains both Gaussian and Rician noise [33] and may benefit from denoising. As another example, intensities measured using MR may be non-uniform across an image and could require correction [9,61,84]. FDG-PET-based may furthermore be corrected for partial volume effects [12,66] and noise [26,43]. In CT imaging, metal objects, e.g. pacemakers and tooth implants, introduce artifacts and may require artifinterpact suppression [32]. Microscopy images generally benefit from field-of-view illumination correction as illumination is usually inhomogeneous due to the light-source or the optical path [13,62].

Evaluation and standardisation of various image post-acquisition processing methods falls outside the scope of the current work. Note that vendors may provide or implement software to perform noise reduction and other post-processing during image reconstruction. In such cases, additional post-acquisition processing may not be required.

2.3 Segmentation OQYT

High-throughput image analysis, within the feature-based paradigm, relies on the definition of regions of interest (ROI). ROIs are used to define the region in which features are calculated. What constitutes an ROI depends on the imaging and the study objective. For example, in 3D microscopy of cell plates, cells are natural ROIs. In medical imaging of cancer patients, the tumour volume is a common ROI. ROIs can be defined manually by experts or (semi-)automatically using algorithms.

From a process point-of-view, segmentation leads to the creation of an ROI mask $\mathbf{R}$, for which every voxel $j \in \mathbf{R}$ (R_j) is defined as:

$$R_j = \begin{cases} 1 & j \text{ in ROI} \\ 0 & \text{otherwise} \end{cases}$$

ROIs are typically stored with the accompanying image. Some image formats directly store ROI masks as voxels (e.g. NIfTI, NRRD and DICOM Segmentation), and generating the ROI mask is conducted by loading the corresponding image. In other cases the ROI is saved as a set of (x, y, z) points that define closed loops of (planar) polygons, for example within DICOM RTSTRUCT or DICOM SR files. In such cases, we should determine which voxel centers lie within the space enclosed by the contour polygon in each slice to generate the ROI mask.

A common method to determine whether a point in an image slice lies inside a 2D polygon is the *crossing number* algorithm, for which several implementations exist [58]. The main concept behind this algorithm is that for any point inside the polygon, any line originating outside the polygon will cross the polygon an uneven number of times. A simple example is shown in figure 2.2. The implementation in the example makes use of the fact that the ROI mask is a regular grid to scan entire rows at a time. The example implementation consists of the following steps:

1. (optional) A ray is cast horizontally from outside the polygon for each of the n image

rows. As we iterate over the rows, it is computationally beneficial to exclude polygon edges that will not be crossed by the ray for the current row j. If the current row has y-coordinate y_j, and edge k has two vertices with y-coordinates y_{k1} and y_{k2}, the ray will not cross the edge if both vertices lie either above or below y_j, i.e. $y_j < y_{k1}, y_{k2}$ or $y_j > y_{k1}, y_{k2}$. For each row j, find those polygon edges whose y-component of the vertices do not both lie on the same side of the row coordinate y_j. This step is used to limit calculation of intersection points to only those that cross a ray cast from outside the polygon – e.g. ray with origin $(-1, y_j)$ and direction $(1, 0)$. This an optional step.

2. Determine intersection points x_i of the (remaining) polygon edges with the ray.

3. Iterate over intersection points and add 1 to the count of each pixel center with $x \geq x_i$.

4. Apply the *even-odd* rule. Pixels with an odd count are inside the polygon, whereas pixels with an even count are outside.

Note that the example represents a relatively naive implementation that will not consistently assign voxel centers positioned on the polygon itself to the interior.

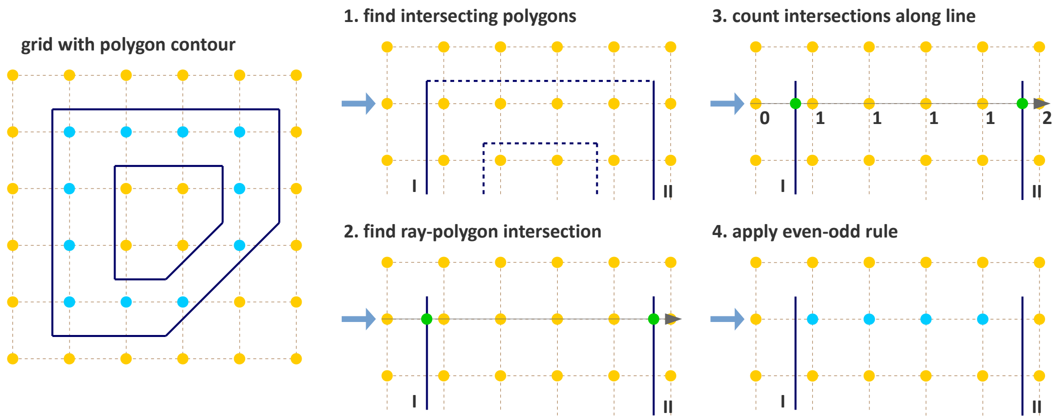

Figure 2.2 | Simple algorithm to determine which pixels are inside a 2D polygon. The suggested implementation consists of four steps: (1) Omit edges that will not intersect with the current row of voxel centers. (2) Calculate intersection points of edges I and II with the ray for the current row. (3) Determine the number of intersections crossed from ray origin to the row voxel centers. (4) Apply *even-odd* rule to determine whether voxel centers are inside the polygon.

2.4 Interpolation

VTM2

Texture feature sets require interpolation to isotropic voxel spacing to be rotationally invariant, and to allow comparison between image data from different samples, cohorts or batches. Voxel interpolation affects image feature values as many image features are sensitive to changes in voxel size[4,8,59,60,87]. Maintaining consistent isotropic voxel spacing across different measurements and devices is therefore important for reproducibility. At the moment there are no clear indications whether upsampling or downsampling schemes are preferable. Consider, for example, an image stack of slices with $1.0 \times 1.0 \times 3.0$ mm^3 voxel spacing. Upsampling to $1.0 \times 1.0 \times 1.0$ mm^3 requires inference and introduces artificial information, while conversely downsampling to the largest dimension ($3.0 \times 3.0 \times 3.0$ mm^3) incurs information loss. Multiple-scaling strategies potentially offer a good trade-off[78]. Note

that downsampling may introduce image aliasing artifacts. Downsampling may therefore require anti-aliasing filters prior to filtering[49,90].

While in general 3D interpolation algorithms are used to interpolate 3D images, 2D interpolation within the image slice plane may be recommended in some situations. In 2D interpolation voxels are not interpolated between slices. This may be beneficial if, for example, the spacing between slices is large compared to the desired voxel size, and/or compared to the in-plane spacing. Applying 3D interpolation would either require inferencing a large number of voxels between slices (upsampling), or the loss of a large fraction of in-plane information (downsampling). The disadvantage of 2D interpolation is that voxel spacing is no longer isotropic, and as a consequence texture features can only be calculated in-plane.

Interpolation algorithms

Interpolation algorithms translate image intensities from the original image grid to an interpolation grid. In such grids, voxels are spatially represented by their center. Several algorithms are commonly used for interpolation, such as *nearest neighbour*, *trilinear*, *tricubic convolution* and *tricubic spline interpolation*. In short, *nearest neighbour interpolation* assigns the intensity of the most nearby voxel in the original grid to each voxel in the interpolation grid. *Trilinear interpolation* uses the intensities of the eight most nearby voxels in the original grid to calculate a new interpolated intensity using linear interpolation. *Tricubic convolution* and *tricubic spline interpolation* draw upon a larger neighbourhood to evaluate a smooth, continuous third-order polynomial at the voxel centers in the interpolation grid. The difference between *tricubic convolution* and *tricubic spline interpolation* lies in the implementation. Whereas *tricubic spline interpolation* evaluates the smooth and continuous third-order polynomial at every voxel center, *tricubic convolution* approximates the solution using a convolution filter. Though *tricubic convolution* is faster, with modern hardware and common image sizes, the difference in execution speed is practically meaningless. Both interpolation algorithms produce similar results, and both are often referred to as *tricubic interpolation*.

While no consensus exists concerning the optimal choice of interpolation algorithm, *trilinear interpolation* is usually seen as a conservative choice. It does not lead to the blockiness produced by *nearest neighbour interpolation* that introduces bias in local textures[38]. Nor does it lead to out-of-range intensities which may occur due to overshoot with *tricubic* and higher order interpolations. The latter problem can occur in acute intensity transitions, where the local neighbourhood itself is not sufficiently smooth to evaluate the polynomial within the allowed range. *Tricubic* methods, however, may retain tissue contrast differences better. Particularly when upsampling, *trilinear* interpolation may act as a low-pass filter which suppresses higher spatial frequencies and cause artefacts in high-pass spatial filters. Interpolation algorithms and their advantages and disadvantages are treated in more detail elsewhere, e.g. Thévenaz et al.[70].

In a phantom study, Larue et al.[42] compared *nearest neighbour*, *trilinear* and *tricubic* interpolation and indicated that feature reproducibility is dependent on the selected interpolation algorithm, i.e. some features were more reproducible using one particular algorithm.

Rounding image intensities after interpolation 68QD

Image intensities may require rounding after interpolation, or the application of cut-off values. For example, in CT images intensities represent Hounsfield Units, and these do not take non-integer values. Following voxel interpolation, interpolated CT intensities are thus rounded to the nearest integer.

Partial volume effects in the ROI mask E8H9

If the image on which the ROI mask was defined, is interpolated after the ROI was segmented, the ROI mask $\mathbf{R}$ should likewise be interpolated to the same dimensions. Interpolation of the ROI mask is best conducted using either the *nearest neighbour* or *trilinear interpolation* methods, as these are guaranteed to produce meaningful masks. *Trilinear interpolation* of the ROI mask leads to partial volume effects, with some voxels containing fractions of the original voxels. Since a ROI mask is a binary mask, such fractions need to be binarised by setting a partial volume threshold δ:

$$R_j = \begin{cases} 1 & R_{interp,j} \geq \delta \\ 0 & R_{interp,j} < \delta \end{cases}$$

A common choice for the partial volume threshold is $\delta = 0.5$. For *nearest neighbour interpolation* the ROI mask does not contain partial volume fractions, and may be used directly.

Interpolation results depend on the floating point representation used for the image and ROI masks. Floating point representations should at least be full precision (32-bit) to avoid rounding errors.

Interpolation grid UMPJ

Interpolated voxel centers lie on the intersections of a regularly spaced grid. Grid intersections are represented by two coordinate systems. The first coordinate system is the grid coordinate system, with origin at $(0.0, 0.0, 0.0)$ and distance between directly neighbouring voxel centers (spacing) of 1.0. The grid coordinate system is the coordinate system typically used by computers, and consequentially, by interpolation algorithms. The second coordinate system is the world coordinate system, which is typically found in (medical) imaging and provides an image scale. As the desired isotropic spacing is commonly defined in world coordinate dimensions, conversions between world coordinates and grid coordinates are necessary, and are treated in more detail after assessing grid alignment methods.

Grid alignment affects feature values and is non-trivial. Three common grid alignments may be identified, and are shown in figure 2.3:

1. **Fit to original grid** (58MB). In this case the interpolation grid is deformed so that the voxel centers at the grid intersections overlap with the original grid vertices. For an original 4×4 voxel grid with spacing $(3.00, 3.00)$ mm and a desired interpolation spacing of $(2.00, 2.00)$ mm we first calculate the extent of the original voxel grid in world coordinates leading to an extent of $((4-1)\,3.00, ((4-1)\,3.00) = (9.00, 9.00)$ mm. In this case the interpolated grid will not exactly fit the original grid. Therefore we try to find the closest fitting grid, which leads to a $6{\times}6$ grid by rounding up $(9.00/2.00, 9.00/2.00)$. The resulting grid has a grid spacing of $(1.80, 1.80)$ mm in world coordinates, which differs from the desired grid spacing of $(2.00, 2.00)$ mm.

2. **Align grid origins** (SBKJ). A simple approach which conserves the desired grid spacing is the alignment of the origins of the interpolation and original grids. Keeping with the same example, the interpolation grid is (6×6). The resulting voxel grid has a grid spacing of $(2.00, 2.00)$ mm in world coordinates. By definition both grids are aligned at the origin, $(0.00, 0.00)$.

3. **Align grid centers** (3WE3). The position of the origin may depend on image meta-data defining image orientation. Not all software implementations may process this meta-data the same way. An implementation-independent solution is to align both grids

on the grid center. Again, keeping with the same example, the interpolation grid is (6×6). Thus, the resulting voxel grid has a grid spacing of $(2.00, 2.00)$ mm in world coordinates.

Align grid centers is recommended as it is implementation-independent and achieves the desired voxel spacing. Technical details of implementing *align grid centers* are described below.

Interpolation grid dimensions 026Q

The dimensions of the interpolation grid are determined as follows. Let n_a be the number of points along one axis of the original grid and $s_{a,w}$ their spacing in world coordinates. Then, let $s_{b,w}$ be the desired spacing after interpolation. The axial dimension of the interpolated mesh grid is then:

$$n_b = \left\lceil \frac{n_a s_a}{s_b} \right\rceil$$

Rounding towards infinity guarantees that the interpolation grid exists even when the original grid contains few voxels. However, it also means that the interpolation mesh grid is partially located outside of the original grid. Extrapolation is thus required. Padding the original grid with the intensities at the boundary is recommended. Some implementations of interpolation algorithms may perform this padding internally.

Interpolation grid position QCY4

For the *align grid centers* method, the positions of the interpolation grid points are determined as follows. As before, let n_a and n_b be the dimensions of one axis in the original and interpolation grid, respectively. Moreover, let $s_{a,w}$ be the original spacing and $s_{b,w}$ the desired spacing for the same axis in world coordinates. Then, with $x_{a,w}$ the origin of the original grid in world coordinates, the origin of the interpolation grid is located at:

$$x_{b,w} = x_{a,w} + \frac{s_a(n_a - 1) - s_b(n_b - 1)}{2}$$

In the grid coordinate system, the original grid origin is located at $x_{a,g} = 0$. The origin of the interpolation grid is then located at:

$$x_{b,g} = \frac{1}{2}\left(n_a - 1 - \frac{s_{b,w}}{s_{a,w}}\,(n_b - 1)\right)$$

Here the fraction $s_{b,w}/s_{a,w} = s_{b,g}$ is the desired spacing in grid coordinates. Thus, the interpolation grid points along the considered axis are located at grid coordinates:

$$x_{b,g},\, x_{b,g} + s_{b,g},\, x_{b,g} + 2s_{b,g},\, \ldots,\, x_{b,g} + (n_b - 1)s_{b,g}$$

Naturally, the above description applies to each grid axis.

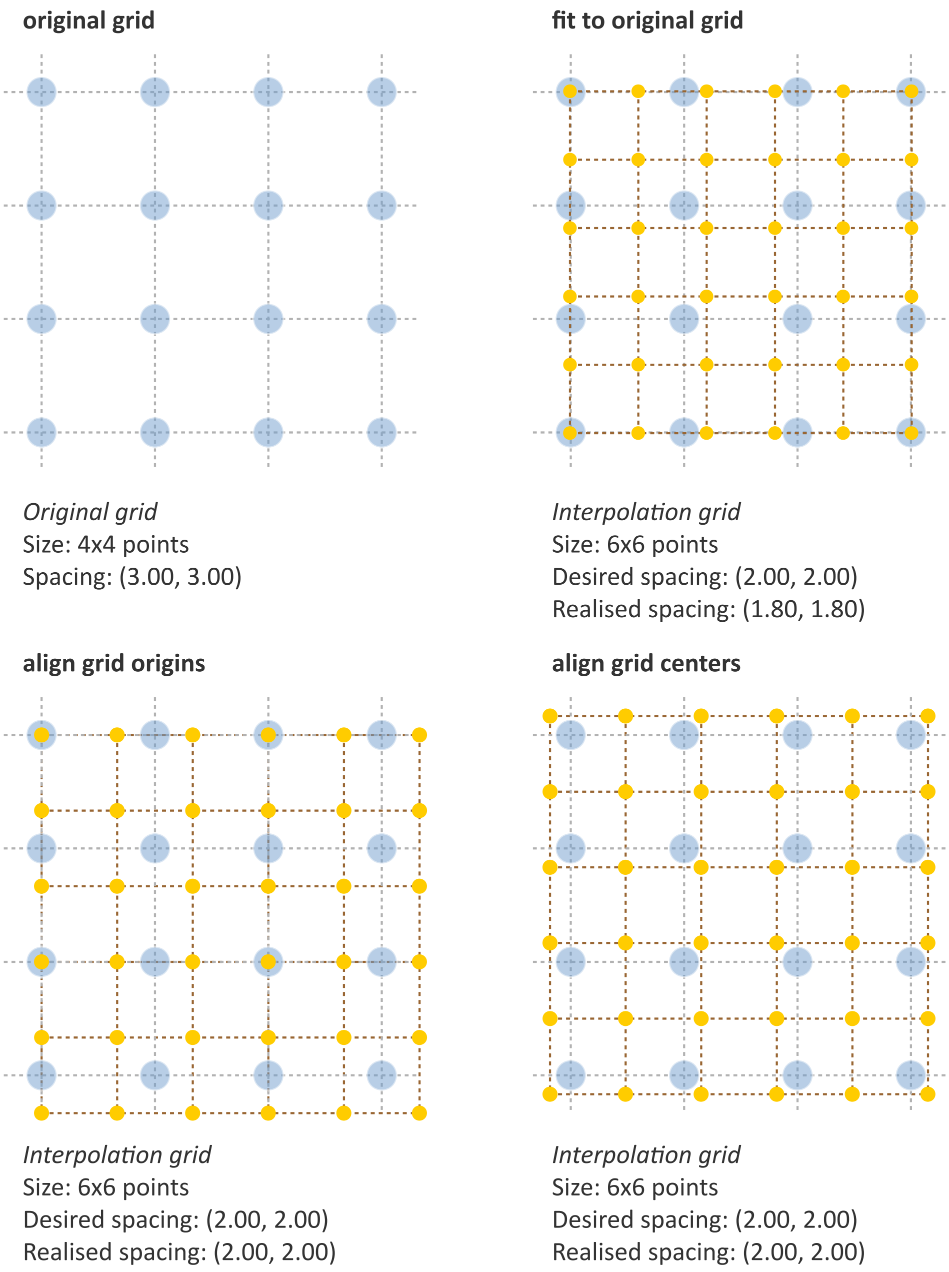

Figure 2.3 | Different interpolation mesh grids based on an original 4×4 grid with $(3.00, 3.00)$ mm spacing. The desired interpolation spacing is $(2.00, 2.00)$ mm. *Fit to original grid* creates an interpolation mesh grid that overlaps with the corners of the original grid. *Align grid origins* creates an interpolation mesh grid that is positioned at the origin of the original grid. *Align grid centers* creates an interpolation grid that is centered on the center of original and interpolation grids.

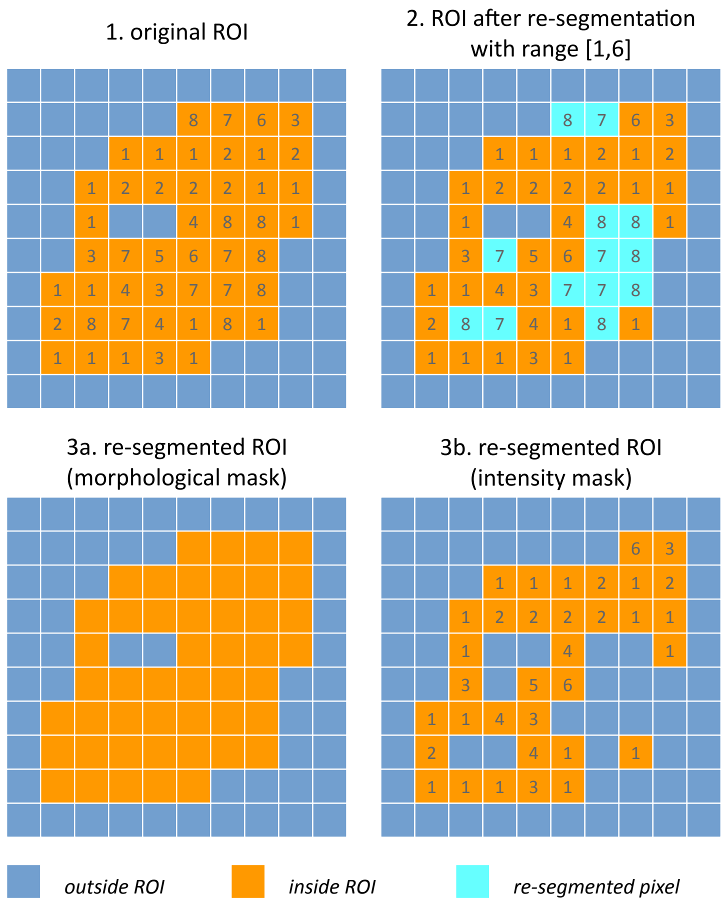

Figure 2.4 | Example showing how intensity and morphological masks may differ due to re-segmentation. (1) The original region of interest (ROI) is shown with pixel intensities. (2) Subsequently, the ROI is re-segmented to only contain values in the range $[1,6]$. Pixels outside this range are marked for removal from the intensity mask. (3a) Resulting morphological mask, which is identical to the original ROI. (3b) Re-segmented intensity mask. Note that due to re-segmentation, intensity and morphological masks are different.

2.5 Re-segmentation IF9H

Re-segmentation entails updating the ROI mask $\mathbf{R}$ based on corresponding voxel intensities $\mathbf{X}_{gl}$. Re-segmentation may be performed to exclude voxels from a previously segmented ROI, and is performed after interpolation. An example use would be the exclusion of air or bone voxels from an ROI defined on CT imaging. Two common re-segmentation methods are described in this section. Combining multiple re-segmentation methods is possible. In this case, the intersection of the intensity ranges defined by the re-segmentation methods is used.

Intensity and morphological masks of an ROI ECJF

Conventionally, an ROI consists of a single mask. However, re-segmentation may lead to exclusion of internal voxels, or divide the ROI into sub-volumes. To avoid undue complexity by again updating the re-segmented ROI for a more plausible morphology, we define two separate ROI masks.

The morphological mask (G5KJ) is not re-segmented and maintains the original mor-

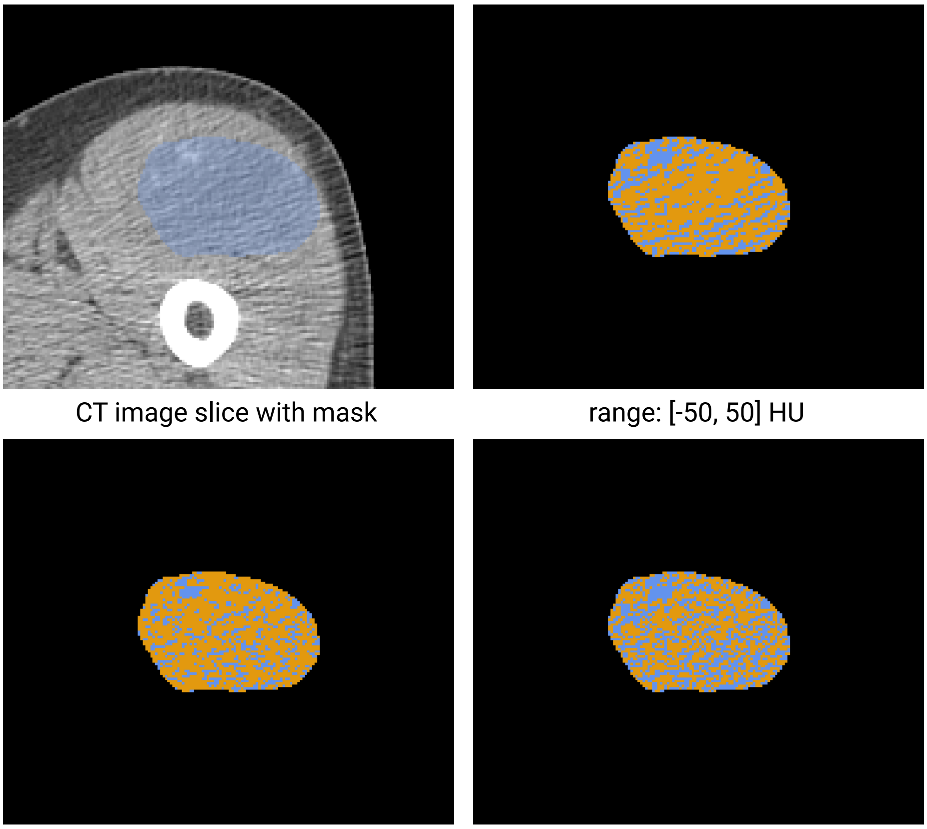

Figure 2.5 | Re-segmentation example based on a CT-image. The masked region (blue) is re-segmented to create an intensity mask (orange). Examples using three different re-segmentation parameter sets are shown. The bottom right combines the range and outlier re-segmentation, and the resulting mask is the intersection of the masks in the other two examples. Image data from Vallières et al.[16,76,77].

phology as defined by an expert and/or (semi-)automatic segmentation algorithms.

The intensity mask (`SEFI`) can be re-segmented and will contain only the selected voxels. For many feature families, only this is important. However, for morphological and grey level distance zone matrix (GLDZM) feature families, both intensity and morphological masks are used. A two-dimensional schematic example is shown in figure 2.4, and a real example is shown in figure 2.5.

Range re-segmentation USB3

Re-segmentation may be performed to remove voxels from the intensity mask that fall outside of a specified range. An example is the exclusion of voxels with Hounsfield Units indicating air and bone tissue in the tumour ROI within CT images, or low activity areas in PET images. Such ranges of intensities of included voxels are usually presented as a closed interval $[a, b]$ or half-open interval $[a, \infty)$, respectively. For arbitrary intensity units (found in e.g. raw MRI data, uncalibrated microscopy images, and many spatial filters), no re-segmentation range can be provided.

When a re-segmentation range is defined by the user, it needs to be propagated and used for the calculation of features that require a specified intensity range (e.g. intensity-volume histogram features) and/or that employs *fixed bin size* discretisation. Recommendations for the possible combinations of different imaging intensity definitions, re-segmentation ranges and discretisation algorithms are provided in Table 2.1.

Intensity outlier filtering 7ACA

ROI voxels with outlier intensities may be removed from the intensity mask. One method for defining outliers was suggested by Vallières et al.[76] after Collewet et al.[19]. The mean μ and standard deviation σ of grey levels of voxels assigned to the ROI are calculated. Voxels outside the range $[\mu - 3\sigma, \mu + 3\sigma]$ are subsequently excluded from the intensity mask.

2.6 ROI extraction 10BP

Many feature families require that the ROI is isolated from the surrounding voxels. The ROI intensity mask is used to extract the image volume to be studied. Excluded voxels are commonly replaced by a placeholder value, often *NaN*. This placeholder value may then used to exclude these voxels from calculations. Voxels included in the ROI mask retain their original intensity. An example is shown in figure 2.6.

2.7 Intensity discretisation 4R0B

Discretisation or quantisation of image intensities inside the ROI is often required to make calculation of texture features tractable[88], and possesses noise-suppressing properties as well. An example of discretisation is shown in figure 2.7.

Two approaches to discretisation are commonly used. One involves the discretisation to a fixed number of bins, and the other discretisation with a fixed bin width. As we will observe, there is no inherent preference for one or the other method. However, both methods have particular characteristics (as described below) that may make them better suited for specific purposes. Note that the lowest bin always has value 1, and not 0. This ensures consistency for calculations of texture features, where for some features grey level 0 is not allowed .

Fixed bin number K15C

In the *fixed bin number* method, intensities X_{gl} are discretised to a fixed number of N_g bins. It is defined as follows:

$$X_{d,k} = \begin{cases} \left\lfloor N_g \frac{X_{gl,k} - X_{gl,min}}{X_{gl,max} - X_{gl,min}} \right\rfloor + 1 & X_{gl,k} < X_{gl,max} \\ N_g & X_{gl,k} = X_{gl,max} \end{cases}$$

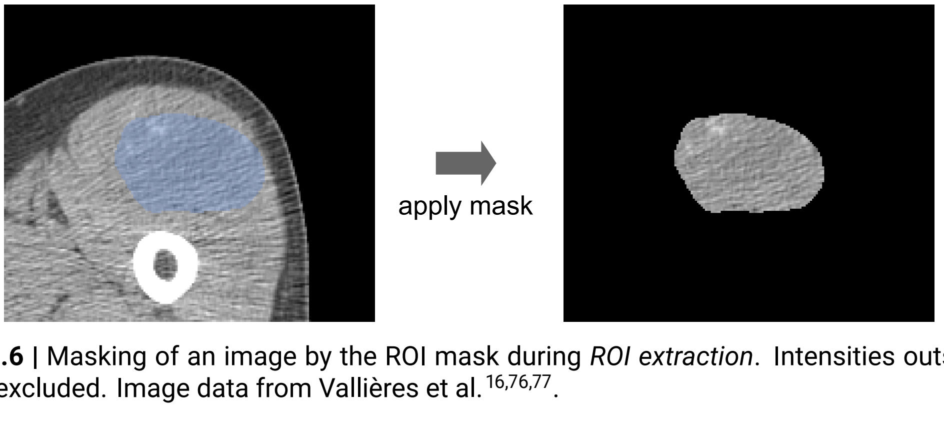

Figure 2.6 | Masking of an image by the ROI mask during *ROI extraction*. Intensities outside the ROI are excluded. Image data from Vallières et al.[16,76,77].

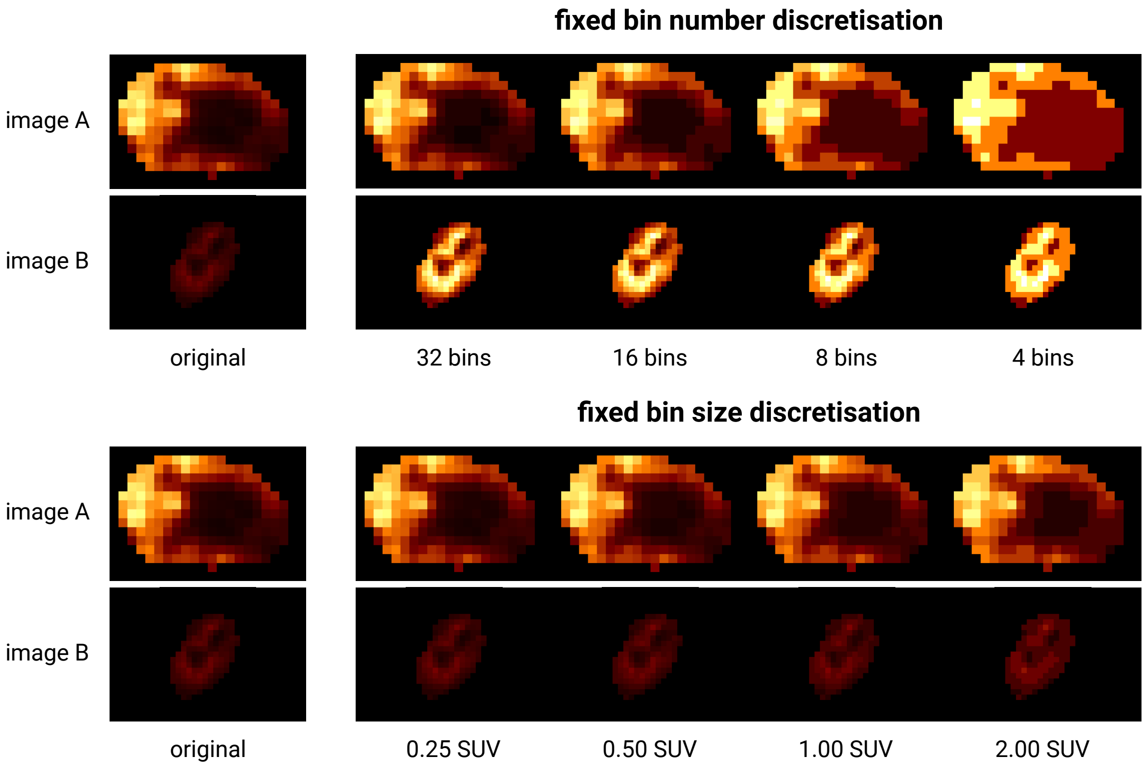

Figure 2.7 | Discretisation of two different 18F-FDG-PET images with SUV_{max} of 27.8 (A) and 6.6 (B). *Fixed bin number* discretisation adjust the contrast between the two images, with the number of bins determining the coarseness of the discretised image. *Fixed bin size* discretisation leaves the contrast differences between image A and B intact. Increasing the bin size increases the coarseness of the discretised image. Image data from Vallières et al.[16,76,77].

In short, the intensity $X_{gl,k}$ of voxel k is corrected by the lowest occurring intensity $X_{gl,min}$ in the ROI, divided by the bin width $\left(X_{gl,max} - X_{gl,min}\right) / N_g$, and subsequently rounded down to the nearest integer (floor function).

The *fixed bin number* method breaks the relationship between image intensity and physiological meaning (if any). However, it introduces a normalising effect which may be beneficial when intensity units are arbitrary (e.g. raw MRI data and many spatial filters), and where contrast is considered important. Furthermore, as values of many features depend on the number of grey levels found within a given ROI, the use of a *fixed bin number* discretisation algorithm allows for a direct comparison of feature values across multiple analysed ROIs (e.g. across different samples).

Fixed bin size Q3RU

Fixed bin size discretisation is conceptually simple. A new bin is assigned for every intensity interval with width w_b; i.e. w_b is the bin width, starting at a minimum $X_{gl,min}$. The minimum intensity may be a user-set value as defined by the lower bound of the re-segmentation range, or data-driven as defined by the minimum intensity in the ROI $X_{gl,min} = \min\left(X_{gl}\right)$. In all cases, the method used and/or set minimum value must be clearly reported. However, to maintain consistency between samples, we strongly recommend to always set the same minimum value for all samples as defined by the lower bound of the re-segmentation range (e.g. HU of -500 for CT, SUV of 0 for PET, etc.). In the case that no re-segmentation range may be defined due to arbitrary intensity units (e.g. raw MRI data and many spatial filters), the use of the *fixed bin size* discretisation algorithm is not recommended.

**Imaging intensity units**$^{(1)}$	**Re-segmentation range**	**FBN**$^{(2)}$	**FBS**$^{(3)}$
	$[a,b]$	✔	✔
calibrated	$[a,\infty)$	✔	✔
	none	✔	×
arbitrary	none	✔	×

Table 2.1 | Recommendations for the possible combinations of different imaging intensity definitions, re-segmentation ranges and discretisation algorithms. Checkmarks (✔) represent recommended combinations of re-segmentation range and discretisation algorithm, whereas crossmarks (×) represent non-recommended combinations.
$^{(1)}$ PET and CT are examples of imaging modalities with *calibrated* intensity units (e.g. SUV and HU, respectively), and raw MRI data of arbitrary intensity units.
$^{(2)}$ *Fixed bin number* (FBN) discretisation uses the actual range of intensities in the analysed ROI (re-segmented or not), and not the re-segmentation range itself (when defined).
$^{(3)}$ *Fixed bin size* (FBS) discretisation uses the lower bound of the re-segmentation range as the minimum set value. When the re-segmentation range is not or cannot be defined (e.g. arbitrary intensity units), the use of the FBS algorithm is not recommended.

The *fixed bin size* method has the advantage of maintaining a direct relationship with the original intensity scale, which could be useful for functional imaging modalities such as PET.

Discretised intensities are computed as follows:

$$X_{d,k} = \left\lfloor \frac{X_{gl,k} - X_{gl,min}}{w_b} \right\rfloor + 1$$

In short, the minimum intensity $X_{gl,min}$ is subtracted from intensity $X_{gl,k}$ in voxel k, and then divided by the bin width w_b. The resulting value is subsequently rounded down to the nearest integer (floor function), and 1 is added to arrive at the discretised intensity.

Other methods

Many other methods and variations for discretisation exist, but are not described in detail here. Vallières et al.[76] described the use of *intensity histogram equalisation* and *Lloyd-Max* algorithms for discretisation. *Intensity histogram equalisation* involves redistributing intensities so that the resulting bins contain a similar number of voxels, i.e. contrast is increased by flattening the histogram as much as possible[34]. Histogram equalisation of the ROI imaging intensities can be performed before any other discretisation algorithm (e.g. FBN, FSB, etc.), and it also requires the definition of a given number of bins in the histogram to be equalised. The *Lloyd-Max* algorithm is an iterative clustering method that seeks to minimise mean squared discretisation errors[47,50].

Recommendations

The discretisation method that leads to optimal feature inter- and intra-sample reproducibility is modality-dependent. Usage recommendations for the possible combinations of different imaging intensity definitions, re-segmentation ranges and discretisation algorithms are provided in Table 2.1. Overall, the discretisation choice has a substantial impact on intensity distributions, feature values and reproducibility[4,25,37,38,44,59,83].

2.8 Feature calculation

Feature calculation is the final processing step where feature descriptors are used to quantify characteristics of the ROI. After calculation such features may be used as image biomarkers by relating them to physiological and medical outcomes of interest. Feature calculation is handled in full details in the next chapter.

Let us recall that the image processing steps leading to image biomarker calculations can be performed in many different ways, notably in terms of spatial filtering, segmentation, interpolation and discretisation parameters. Furthermore, it is plausible that different texture features will better quantify the characteristics of the ROI when computed using different image processing parameters. For example, a lower number of grey levels in the discretisation process (e.g. 8 or 16) may allow to better characterize the sub-regions of the ROI using *grey level size zone matrix* (GLSZM) features, whereas *grey level co-occurence matrix* (GLCM) features may be better modeled with a higher number of grey levels (e.g. 32 or 64). Overall, these possible differences opens the door to the optimization of image processing parameters for each different feature in terms of a specific objective. For the specific case of the optimization of image interpolation and discretisation prior to texture analysis, Vallières *et al.*[76] have named this process *texture optimization*. The authors notably suggested that the *texture optimization* process could have significant influence of the prognostic capability of subsequent features. In another study[78], the authors constructed predictive models using textures calculated from all possible combinations of PET and CT images interpolated at four isotropic resolutions and discretised with two different algorithms and four numbers of grey levels.

Chapter 3

Image features

In this chapter we will describe a set of quantitative image features together with the reference values established by the IBSI. This feature set builds upon the feature sets proposed by Aerts et al.[1] and Hatt et al.[38], which are themselves largely derived from earlier works. References to earlier work are provided whenever they could be identified.

Reference values were derived for each feature. A table of reference values contains the values that could be reliably reproduced, within a tolerance margin, for the reference data sets (see Chapter 5). Consensus on the validity of each reference value is also noted. Consensus can have four levels, depending on the number of teams that were able to produce the same value during the standardization process: weak (<3 matches), moderate (3 to 5 matches), strong (6 to 9 matches), and very strong (≥ 10 matches). If consensus on a reference value was weak or if it could not be reproduced by an absolute majority of teams, it was not considered standardized. Such features do currently not have reference values, and should not be used.

The set of features can be divided into a number of families, of which intensity-based statistical, intensity histogram-based, intensity-volume histogram-based, morphological features, local intensity, and texture matrix-based features are treated here. All texture matrices are rotationally and translationally invariant. Illumination invariance of texture matrices may be achieved by particular image post-acquisition schemes, e.g. *histogram matching*. None of the texture matrices are scale invariant, a property which can be useful in many (biomedical) applications. What the presented texture matrices lack, however, is directionality in combination with rotation invariance. These may be achieved by local binary patterns and steerable filters, which however fall beyond the scope of the current work. For these and other texture features, see Depeursinge et al.[24].

Features are calculated on the base image, as well as images transformed using wavelet or Gabor filters). To calculate features, it is assumed that an image segmentation mask exists, which identifies the voxels located within a region of interest (ROI). The ROI itself consists of two masks, an intensity mask and a morphological mask. These masks may be identical, but not necessarily so, as described in Section 2.5.

Several feature families require additional image processing steps before feature calculation. Notably intensity histogram and texture feature families require prior discretisation of intensities into grey level bins. Other feature families do not require discretisation before calculations. For more details on image processing, see figure 2.1 in the previous chapter.

Below is an overview table that summarises image processing requirements for the different feature families.

Feature family	count	ROI mask morph.	ROI mask int.	discr.
morphology	29	✔	✔	×
local intensity	2	×	✔[a]	×
intensity-based statistics	18	×	✔	×
intensity histogram	23	×	✔	✔
intensity-volume histogram	5	×	✔	✔[b]
grey level co-occurrence matrix	25	×	✔	✔
grey level run length matrix	16	×	✔	✔
grey level size zone matrix	16	×	✔	✔
grey level distance zone matrix	16	✔	✔	✔
neighbourhood grey tone difference matrix	5	×	✔	✔
neighbouring grey level dependence matrix	17	×	✔	✔

Table 3.1 | Feature families and required image processing. For each feature family, the number of features in the document, the required input of a morphological (morph.) and/or intensity (int.) ROI mask, as well as the requirement of image discretisation (discr.) is provided.
[a] The entire image volume should be available when computing local intensity features.
[b] Image discretisation for the intensity-volume histogram is performed with finer discretisation than required for e.g. textural features.

Though image processing parameters affect feature values, three other concepts influence feature values for many features: distance, feature aggregation and distance weighting. These are described below.

Grid distances MPUJ

Grid distance is an important concept that is used by several feature families, particularly texture features. Grid distances can be measured in several ways. Let $\mathbf{m} = (m_x, m_y, m_z)$ be the vector from a center voxel at $\mathbf{k} = (k_x, k_y, k_z)$ to a neighbour voxel at $\mathbf{k} + \mathbf{m}$. The following norms (distances) are used:

- ℓ_1 norm or *Manhattan* norm (LIFZ):

$$\|\mathbf{m}\|_1 = |m_x| + |m_y| + |m_z|$$

- ℓ_2 norm or *Euclidean* norm (G9EV):

$$\|\mathbf{m}\|_2 = \sqrt{m_x^2 + m_y^2 + m_z^2}$$

- ℓ_∞ norm or *Chebyshev* norm (PVMT):

$$\|\mathbf{m}\|_\infty = \max(|m_x|, |m_y|, |m_z|)$$

An example of how the above norms differ in practice is shown in figure 3.1.

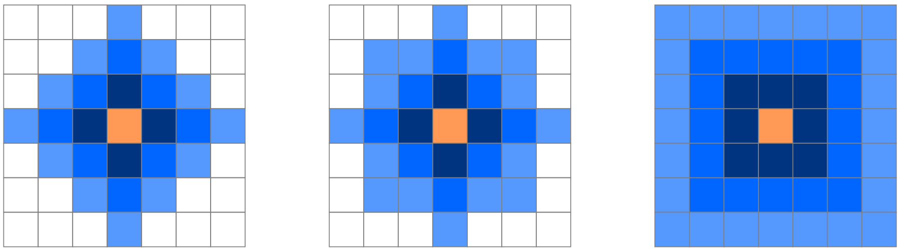

(a) Manhattan norm **(b)** Euclidean norm **(c)** Chebyshev norm

Figure 3.1 | Grid neighbourhoods for distances up to 3 according to Manhattan, Euclidean and Chebyshev norms. The orange pixel is considered the center pixel. Dark blue pixels have distance $\delta = 1$, blue pixels $\delta \leq 2$ and light blue pixels $\delta \leq 3$ for the corresponding norm.

Feature aggregation 5QB6

Features from some families may be calculated from, e.g. slices. As a consequence, multip le values for the same feature may be computed. These different values should be combined into a single value for many common purposes. This process is referred to as feature aggregation. Feature aggregation methods depend on the family, and are detailed in the family description.

Distance weighting 6CK8

Distance weighting is not a default operation for any of the texture families, but is implemented in software such as PyRadiomics[81]. It may for example be used to put more emphasis on local intensities.

3.1 Morphological features HCUG

Morphological features describe geometric aspects of a region of interest (ROI), such as area and volume. Morphological features are based on ROI voxel representations of the volume. Three voxel representations of the volume are conceivable:

1. The volume is represented by a collection of voxels with each voxel taking up a certain volume (LQD8).
2. The volume is represented by a voxel point set $\mathbf{X}_c$ that consists of coordinates of the voxel centers (4KW8).
3. The volume is represented by a surface mesh (WRJH).

We use the second representation when the inner structure of the volume is important, and the third representation when only the outer surface structure is important. The first representation is not used outside volume approximations because it does not handle partial volume effects at the ROI edge well, and also to avoid inconsistencies in feature values introduced by mixing representations in small voxel volumes.

Mesh-based representation WRJH

A mesh-based representation of the outer surface allows consistent evaluation of the surface volume and area independent of size. Voxel-based representations lead to partial volume effects and over-estimation of the surface area. The surface of the ROI volume is translated into a triangle mesh using a meshing algorithm. While multiple meshing algorithms exist, we suggest the use of the *Marching Cubes* algorithm[45,48] because of its widespread availability in different programming languages and reasonable approximation of the surface area and volume[67]. In practice, mesh-based feature values depend upon the meshing algorithm and small differences may occur between implementations[46].

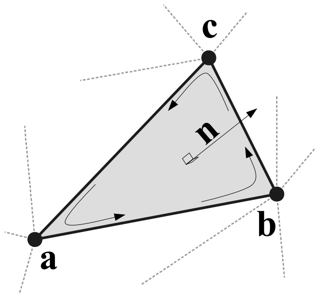

Figure 3.2 | Meshing algorithms draw faces and vertices to cover the ROI. One face, spanned by vertices $\mathbf{a}$, $\mathbf{b}$ and $\mathbf{c}$, is highlighted. Moreover, the vertices define the three edges $\mathbf{ab} = \mathbf{b} - \mathbf{a}$, $\mathbf{bc} = \mathbf{c} - \mathbf{b}$ and $\mathbf{ca} = \mathbf{a} - \mathbf{c}$. The face normal $\mathbf{n}$ is determined using the right-hand rule, and calculated as $\mathbf{n} = (\mathbf{ab} \times \mathbf{bc}) / \|\mathbf{ab} \times \mathbf{bc}\|$, i.e. the outer product of edge $\mathbf{ab}$ with edge $\mathbf{bc}$, normalised by its length.

Meshing algorithms use the ROI voxel point set $\mathbf{X}_c$ to create a closed mesh. Dependent on the algorithm, a parameter is required to specify where the mesh should be drawn. A default level of 0.5 times the voxel spacing is used for marching cube algorithms. Other algorithms require a so-called *isovalue*, for which a value of 0.5 can be used since the ROI mask consists of 0 and 1 values, and we want to roughly draw the mesh half-way between voxel centers. Depending on implementation, algorithms may also require padding of the

ROI mask with non-ROI (0) voxels to correctly estimate the mesh in places where ROI voxels would otherwise be located at the edge of the mask.

The closed mesh drawn by the meshing algorithm consists of N_{fc} triangle faces spanned by N_{vx} vertex points. An example triangle face is drawn in Figure 3.2. The set of vertex points is then $\mathbf{X}_{vx}$.

The calculation of the mesh volume requires that all faces have the same orientation of the face normal. Consistent orientation can be checked by the fact that in a regular, closed mesh, all edges are shared between exactly two faces. Given the edge spanned by vertices $\mathbf{a}$ and $\mathbf{b}$, the edge must be $\mathbf{ab} = \mathbf{b} - \mathbf{a}$ for one face and $\mathbf{ba} = \mathbf{a} - \mathbf{b}$ for the adjacent face. This ensures consistent application of the right-hand rule, and thus consistent orientation of the face normals. Algorithm implementations may return consistently orientated faces by default.

ROI morphological and intensity masks

The ROI consists of a morphological and an intensity mask. The morphological mask is used to calculate many of the morphological features and to generate the voxel point set $\mathbf{X}_c$. Any holes within the morphological mask are understood to be the result of segmentation decisions, and thus to be intentional. The intensity mask is used to generate the voxel intensity set $\mathbf{X}_{gl}$ with corresponding point set $\mathbf{X}_{c,gl}$.

Aggregating features

By definition, morphological features are calculated in 3D (DHQ4), and not per slice.

Units of measurement

By definition, morphological features are computed using the unit of length as defined in the DICOM standard, i.e. millimeter for most medical imaging modalities[1].

If the unit of length is not defined by a standard, but is explicitly defined as meta data, this definition should be used. In this case, care should be taken that this definition is consistent across all data in the cohort.

If a feature value should be expressed as a different unit of length, e.g. cm instead of mm, such conversions should take place after computing the value using the standard units.

3.1.1 Volume (mesh) RNU0

The mesh-based *volume* V is calculated from the ROI mesh as follows[89]. A tetrahedron is formed by each face k and the origin. By placing the origin vertex of each tetrahedron at $(0,0,0)$, the signed volume of the tetrahedron is:

$$V_k = \frac{\mathbf{a} \cdot (\mathbf{b} \times \mathbf{c})}{6}$$

Here $\mathbf{a}$, $\mathbf{b}$ and $\mathbf{c}$ are the vertex points of face k. Depending on the orientation of the normal, the signed volume may be positive or negative. Hence, the orientation of face normals should be consistent, e.g. all normals must be either pointing outward or inward. The *volume* V is then calculated by summing over the face volumes, and taking the absolute

[1]DICOM PS3.3 2019a - Information Object Definitions, Section 10.7.1.3

value:

$$F_{morph.vol} = V = \left|\sum_{k=1}^{N_{fc}} V_k\right|$$

In positron emission tomography, the *volume* of the ROI commonly receives a name related to the radioactive tracer, e.g. *metabolically active tumour volume* (MATV) for ^{18}F-FDG.

data	value	tol.	consensus
dig. phantom	556	4	very strong
config. A	3.58×10^5	5×10^3	very strong
config. B	3.58×10^5	5×10^3	strong
config. C	3.67×10^5	6×10^3	strong
config. D	3.67×10^5	6×10^3	strong
config. E	3.67×10^5	6×10^3	strong

Table 3.2 | Reference values for the *volume (mesh)* feature.

3.1.2 Volume (voxel counting) YEKZ

In clinical practice, volumes are commonly determined by counting voxels. For volumes consisting of a large number of voxels (1000s), the differences between *voxel counting* and *mesh-based* approaches are usually negligible. However for volumes with a low number of voxels (10s to 100s), *voxel counting* will overestimate volume compared to the *mesh-based* approach. It is therefore only used as a reference feature, and not in the calculation of other morphological features.

Voxel counting volume is defined as:

$$F_{morph.approx.vol} = \sum_{k=1}^{N_v} V_k$$

Here N_v is the number of voxels in the morphological mask of the ROI, and V_k the volume of voxel k.

data	value	tol.	consensus
dig. phantom	592	4	very strong
config. A	3.59×10^5	5×10^3	strong
config. B	3.58×10^5	5×10^3	strong
config. C	3.68×10^5	6×10^3	strong
config. D	3.68×10^5	6×10^3	strong
config. E	3.68×10^5	6×10^3	strong

Table 3.3 | Reference values for the *volume (voxel counting)* feature.

3.1.3 Surface area (mesh) C0JK

The *surface area* A is also calculated from the ROI mesh by summing over the triangular face surface areas[1]. By definition, the area of face k is:

$$A_k = \frac{|\mathbf{ab} \times \mathbf{ac}|}{2}$$

As in Figure 3.2, edge $\mathbf{ab} = \mathbf{b} - \mathbf{a}$ is the vector from vertex $\mathbf{a}$ to vertex $\mathbf{b}$, and edge $\mathbf{ac} = \mathbf{c} - \mathbf{a}$ the vector from vertex $\mathbf{a}$ to vertex $\mathbf{c}$. The total *surface area* A is then:

$$F_{morph.area} = A = \sum_{k=1}^{N_{fc}} A_k$$

data	value	tol.	consensus
dig. phantom	388	3	very strong
config. A	3.57×10^4	300	strong
config. B	3.37×10^4	300	strong
config. C	3.43×10^4	400	strong
config. D	3.43×10^4	400	strong
config. E	3.43×10^4	400	strong

Table 3.4 | Reference values for the *surface area (mesh)* feature.

3.1.4 Surface to volume ratio 2PR5

The *surface to volume ratio* is given as[1]:

$$F_{morph.av} = \frac{A}{V}$$

Note that this feature is not dimensionless.

data	value	tol.	consensus
dig. phantom	0.698	0.004	very strong
config. A	0.0996	0.0005	strong
config. B	0.0944	0.0005	strong
config. C	0.0934	0.0007	strong
config. D	0.0934	0.0007	strong
config. E	0.0934	0.0007	strong

Table 3.5 | Reference values for the *surface to volume ratio* feature.

3.1.5 Compactness 1 SKGS

Several features (*compactness 1* and *2*, *spherical disproportion*, *sphericity* and *asphericity*) quantify the deviation of the ROI volume from a representative spheroid. All these definitions can be derived from one another. As a results these features are are highly correlated and may thus

be redundant. *Compactness 1*[1] is a measure for how compact, or sphere-like the volume is. It is defined as:

$$F_{morph.comp.1} = \frac{V}{\pi^{1/2} A^{3/2}}$$

Compactness 1 is sometimes[1] defined using $A^{2/3}$ instead of $A^{3/2}$, but this does not lead to a dimensionless quantity.

data	value	tol.	consensus
dig. phantom	0.0411	0.0003	strong
config. A	0.03	0.0001	strong
config. B	0.0326	0.0001	strong
config. C	—	—	moderate
config. D	0.0326	0.0002	strong
config. E	0.0326	0.0002	strong

Table 3.6 | Reference values for the *compactness 1* feature. An unset value (—) indicates the lack of a reference value.

3.1.6 Compactness 2 BQWJ

Like *Compactness 1*, *Compactness 2*[1] quantifies how sphere-like the volume is:

$$F_{morph.comp.2} = 36\pi \frac{V^2}{A^3}$$

By definition $F_{morph.comp.1} = 1/6\pi \left(F_{morph.comp.2}\right)^{1/2}$.

data	value	tol.	consensus
dig. phantom	0.599	0.004	strong
config. A	0.319	0.001	strong
config. B	0.377	0.001	strong
config. C	0.378	0.004	strong
config. D	0.378	0.004	strong
config. E	0.378	0.004	strong

Table 3.7 | Reference values for the *compactness 2* feature.

3.1.7 Spherical disproportion KRCK

Spherical disproportion[1] likewise describes how sphere-like the volume is:

$$F_{morph.sph.dispr} = \frac{A}{4\pi R^2} = \frac{A}{\left(36\pi V^2\right)^{1/3}}$$

By definition $F_{morph.sph.dispr} = \left(F_{morph.comp.2}\right)^{-1/3}$.

data	value	tol.	consensus
dig. phantom	1.19	0.01	strong
config. A	1.46	0.01	strong
config. B	1.38	0.01	strong
config. C	1.38	0.01	strong
config. D	1.38	0.01	strong
config. E	1.38	0.01	strong

Table 3.8 | Reference values for the *spherical disproportion* feature.

3.1.8 Sphericity QCFX

Sphericity[1] is a further measure to describe how sphere-like the volume is:

$$F_{morph.sphericity} = \frac{\left(36\pi V^2\right)^{1/3}}{A}$$

By definition $F_{morph.sphericity} = \left(F_{morph.comp.2}\right)^{1/3}$.

data	value	tol.	consensus
dig. phantom	0.843	0.005	very strong
config. A	0.683	0.001	strong
config. B	0.722	0.001	strong
config. C	0.723	0.003	strong
config. D	0.723	0.003	strong
config. E	0.723	0.003	strong

Table 3.9 | Reference values for the *sphericity* feature.

3.1.9 Asphericity 25C7

Asphericity[6] also describes how much the ROI deviates from a perfect sphere, with perfectly spherical volumes having an asphericity of 0. Asphericity is defined as:

$$F_{morph.asphericity} = \left(\frac{1}{36\pi}\frac{A^3}{V^2}\right)^{1/3} - 1$$

By definition $F_{morph.asphericity} = \left(F_{morph.comp.2}\right)^{-1/3} - 1$

data	value	tol.	consensus
dig. phantom	0.186	0.001	strong
config. A	0.463	0.002	strong
config. B	0.385	0.001	moderate
config. C	0.383	0.004	strong
config. D	0.383	0.004	strong
config. E	0.383	0.004	strong

Table 3.10 | Reference values for the *asphericity* feature.

3.1.10 Centre of mass shift KLMA

The distance between the ROI volume centroid and the intensity-weighted ROI volume is an abstraction of the spatial distribution of low/high intensity regions within the ROI. Let $N_{v,m}$ be the number of voxels in the morphological mask. The ROI volume centre of mass is calculated from the morphological voxel point set $\mathbf{X}_c$ as follows:

$$\overrightarrow{CoM}_{geom} = \frac{1}{N_{v,m}} \sum_{k=1}^{N_{v,m}} \vec{X}_{c,k}$$

The intensity-weighted ROI volume is based on the intensity mask. The position of each voxel centre in the intensity mask voxel set $\mathbf{X}_{c,gl}$ is weighted by its corresponding intensity $\mathbf{X}_{gl}$. Therefore, with $N_{v,gl}$ the number of voxels in the intensity mask:

$$\overrightarrow{CoM}_{gl} = \frac{\sum_{k=1}^{N_{v,gl}} X_{gl,k}\vec{X}_{c,gl,k}}{\sum_{k=1}^{N_{v,gl}} X_{gl,k}}$$

The distance between the two centres of mass is then:

$$F_{morph.com} = ||\overrightarrow{CoM}_{geom} - \overrightarrow{CoM}_{gl}||_2$$

data	value	tol.	consensus
dig. phantom	0.672	0.004	very strong
config. A	52.9	28.7	strong
config. B	63.1	29.6	strong
config. C	45.6	2.8	strong
config. D	64.9	2.8	strong
config. E	68.5	2.1	moderate

Table 3.11 | Reference values for the *centre of mass shift* feature.

3.1.11 Maximum 3D diameter L0JK

The *maximum 3D diameter*[1] is the distance between the two most distant vertices in the ROI mesh vertex set $\mathbf{X}_{vx}$:

$$F_{morph.diam} = \max\left(||\vec{X}_{vx,k_1} - \vec{X}_{vx,k_2}||_2\right), \qquad k_1 = 1,\dots,N \qquad k_2 = 1,\dots,N$$

A practical way of determining the *maximum 3D diameter* is to first construct the convex hull of the ROI mesh. The convex hull vertex set $\mathbf{X}_{vx,convex}$ is guaranteed to contain the two most distant vertices of $\mathbf{X}_{vx}$. This significantly reduces the computational cost of calculating distances between all vertices. Despite the remaining $O(n^2)$ cost of calculating distances between different vertices, $\mathbf{X}_{vx,convex}$ is usually considerably smaller in size than $\mathbf{X}_{vx}$. Moreover, the convex hull is later used for the calculation of other morphological features (3.1.25-3.1.26).

data	value	tol.	consensus
dig. phantom	13.1	0.1	strong
config. A	125	1	strong
config. B	125	1	strong
config. C	125	1	strong
config. D	125	1	strong
config. E	125	1	strong

Table 3.12 | Reference values for the *maximum 3D diameter* feature.

3.1.12 Major axis length TDIC

Principal component analysis (PCA) can be used to determine the main orientation of the ROI[65]. On a three dimensional object, PCA yields three orthogonal eigenvectors $\{e_1, e_2, e_3\}$ and three eigenvalues $(\lambda_1, \lambda_2, \lambda_3)$. These eigenvalues and eigenvectors geometrically describe a triaxial ellipsoid. The three eigenvectors determine the orientation of the ellipsoid, whereas the eigenvalues provide a measure of how far the ellipsoid extends along each eigenvector. Several features make use of principal component analysis, namely *major*, *minor* and *least axis length*, *elongation*, *flatness*, and *approximate enclosing ellipsoid volume* and area density.

The eigenvalues can be ordered so that $\lambda_{major} \geq \lambda_{minor} \geq \lambda_{least}$ correspond to the major, minor and least axes of the ellipsoid respectively. The semi-axes lengths a, b and c for the major, minor and least axes are then $2\sqrt{\lambda_{major}}$, $2\sqrt{\lambda_{minor}}$ and $2\sqrt{\lambda_{least}}$ respectively. The *major axis length* is twice the semi-axis length a, determined using the largest eigenvalue obtained by PCA on the point set of voxel centers $\mathbf{X}_c$[39]:

$$F_{morph.pca.major} = 2a = 4\sqrt{\lambda_{major}}$$

data	value	tol.	consensus
dig. phantom	11.4	0.1	very strong
config. A	92.7	0.4	very strong
config. B	92.6	0.4	strong
config. C	93.3	0.5	strong
config. D	93.3	0.5	strong
config. E	93.3	0.5	strong

Table 3.13 | Reference values for the *major axis length* feature.

3.1.13 Minor axis length P9VJ

The *minor axis length* of the ROI provides a measure of how far the volume extends along the second largest axis. The *minor axis length* is twice the semi-axis length b, determined using the second largest eigenvalue obtained by PCA, as described in Section 3.1.12:

$$F_{morph.pca.minor} = 2b = 4\sqrt{\lambda_{minor}}$$

data	value	tol.	consensus
dig. phantom	9.31	0.06	very strong
config. A	81.5	0.4	very strong
config. B	81.3	0.4	strong
config. C	82	0.5	strong
config. D	82	0.5	strong
config. E	82	0.5	strong

Table 3.14 | Reference values for the *minor axis length* feature.

3.1.14 Least axis length 7J51

The least axis is the axis along which the object is least extended. The *least axis length* is twice the semi-axis length c, determined using the smallest eigenvalue obtained by PCA, as described in Section 3.1.12:

$$F_{morph.pca.least} = 2c = 4\sqrt{\lambda_{least}}$$

data	value	tol.	consensus
dig. phantom	8.54	0.05	very strong
config. A	70.1	0.3	strong
config. B	70.2	0.3	strong
config. C	70.9	0.4	strong
config. D	70.9	0.4	strong
config. E	70.9	0.4	strong

Table 3.15 | Reference values for the *least axis length* feature.

3.1.15 Elongation Q3CK

The ratio of the major and minor principal axis lengths could be viewed as the extent to which a volume is longer than it is wide, i.e. is eccentric. For computational reasons, we express *elongation* as an inverse ratio. 1 is thus completely non-elongated, e.g. a sphere, and smaller values express greater elongation of the ROI volume.

$$F_{morph.pca.elongation} = \sqrt{\frac{\lambda_{minor}}{\lambda_{major}}}$$

data	value	tol.	consensus
dig. phantom	0.816	0.005	very strong
config. A	0.879	0.001	strong
config. B	0.878	0.001	strong
config. C	0.879	0.001	strong
config. D	0.879	0.001	strong
config. E	0.879	0.001	strong

Table 3.16 | Reference values for the *elongation* feature.

3.1.16 Flatness N17B

The ratio of the major and least axis lengths could be viewed as the extent to which a volume is flat relative to its length. For computational reasons, we express *flatness* as an inverse ratio. 1 is thus completely non-flat, e.g. a sphere, and smaller values express objects which are increasingly flatter.

$$F_{morph.pca.flatness} = \sqrt{\frac{\lambda_{least}}{\lambda_{major}}}$$

data	value	tol.	consensus
dig. phantom	0.749	0.005	very strong
config. A	0.756	0.001	strong
config. B	0.758	0.001	strong
config. C	0.76	0.001	strong
config. D	0.76	0.001	strong
config. E	0.76	0.001	strong

Table 3.17 | Reference values for the *flatness* feature.

3.1.17 Volume density (axis-aligned bounding box) PBX1

Volume density is the fraction of the ROI volume and a comparison volume. Here the comparison volume is that of the axis-aligned bounding box (AABB) of the ROI mesh vertex set $\mathbf{X}_{vx}$ or the ROI mesh convex hull vertex set $\mathbf{X}_{vx,convex}$. Both vertex sets generate an identical bounding box, which is the smallest box enclosing the vertex set, and aligned with the axes of the reference frame.

$$F_{morph.v.dens.aabb} = \frac{V}{V_{aabb}}$$

This feature is also called *extent*[27,65].

data	value	tol.	consensus
dig. phantom	0.869	0.005	strong
config. A	0.486	0.003	strong
config. B	0.477	0.003	strong
config. C	0.478	0.003	strong
config. D	0.478	0.003	strong
config. E	0.478	0.003	strong

Table 3.18 | Reference values for the *volume density (AABB)* feature.

3.1.18 Area density (axis-aligned bounding box) R59B

Conceptually similar to the *volume density (AABB)* feature, *area density* considers the ratio of the ROI surface area and the surface area A_{aabb} of the axis-aligned bounding box enclosing

the ROI mesh vertex set $\mathbf{X}_{vx}$[80]. The bounding box is identical to the one used for computing the *volume density (AABB)* feature. Thus:

$$F_{morph.a.dens.aabb} = \frac{A}{A_{aabb}}$$

data	value	tol.	consensus
dig. phantom	0.866	0.005	strong
config. A	0.725	0.003	strong
config. B	0.678	0.003	strong
config. C	0.678	0.003	strong
config. D	0.678	0.003	strong
config. E	0.678	0.003	strong

Table 3.19 | Reference values for the *area density (AABB)* feature.

3.1.19 Volume density (oriented minimum bounding box) ZH1A

Note: This feature currently has no reference values and should not be used.

The volume of an axis-aligned bounding box is generally not the smallest obtainable volume enclosing the ROI. By orienting the box along a different set of axes, a smaller enclosing volume may be attainable. The oriented minimum bounding box (OMBB) of the ROI mesh vertex set $\mathbf{X}_{vx}$ or $\mathbf{X}_{vx,convex}$ encloses the vertex set and has the smallest possible volume. A 3D rotating callipers technique was devised by O'Rourke[56] to derive the oriented minimum bounding box. Due to computational complexity of this technique, the oriented minimum bounding box is commonly approximated at lower complexity, see e.g. Barequet and Har-Peled[10] and Chan and Tan[14]. Thus:

$$F_{morph.v.dens.ombb} = \frac{V}{V_{ombb}}$$

Here V_{ombb} is the volume of the oriented minimum bounding box.

3.1.20 Area density (oriented minimum bounding box) IQYR

Note: This feature currently has no reference values and should not be used.

The *area density (OMBB)* is estimated as:

$$F_{morph.a.dens.ombb} = \frac{A}{A_{ombb}}$$

Here A_{ombb} is the surface area of the same bounding box as calculated for the *volume density (OMBB)* feature.

3.1.21 Volume density (approximate enclosing ellipsoid) 6BDE

The eigenvectors and eigenvalues from PCA of the ROI voxel center point set $\mathbf{X}_c$ can be used to describe an ellipsoid approximating the point cloud[51], i.e. the approximate enclosing

ellipsoid (AEE). The volume of this ellipsoid is $V_{aee} = 4\pi\, a\, b\, c/3$, with a, b, and c being the lengths of the ellipsoid's semi-principal axes, see Section 3.1.12. The *volume density (AEE)* is then:

$$F_{morph.v.dens.aee} = \frac{3V}{4\pi abc}$$

data	value	tol.	consensus
dig. phantom	1.17	0.01	moderate
config. A	1.29	0.01	strong
config. B	1.29	0.01	strong
config. C	1.29	0.01	moderate
config. D	1.29	0.01	moderate
config. E	1.29	0.01	strong

Table 3.20 | Reference values for the *volume density (AEE)* feature.

3.1.22 Area density (approximate enclosing ellipsoid) RDD2

The surface area of an ellipsoid can generally not be evaluated in an elementary form. However, it is possible to approximate the surface using an infinite series. We use the same semi-principal axes as for the *volume density (AEE)* feature and define:

$$A_{aee}\left(a,b,c\right) = 4\pi\, a\, b \sum_{\nu=0}^{\infty} \frac{\left(\alpha\, \beta\right)^{\nu}}{1-4\nu^2} P_{\nu}\left(\frac{\alpha^2+\beta^2}{2\alpha\beta}\right)$$

Here $\alpha = \sqrt{1-b^2/a^2}$ and $\beta = \sqrt{1-c^2/a^2}$ are eccentricities of the ellipsoid and P_{ν} is the Legendre polynomial function for degree ν. The Legendre polynomial series, though infinite, converges, and approximation may be stopped early when the incremental gains in precision become limited. By default, we stop the series after $\nu = 20$.

The *area density (AEE)* is then approximated as:

$$F_{morph.a.dens.aee} = \frac{A}{A_{aee}}$$

data	value	tol.	consensus
dig. phantom	1.36	0.01	moderate
config. A	1.71	0.01	moderate
config. B	1.62	0.01	moderate
config. C	1.62	0.01	moderate
config. D	1.62	0.01	moderate
config. E	1.62	0.01	strong

Table 3.21 | Reference values for the *area density (AEE)* feature.

3.1.23 Volume density (minimum volume enclosing ellipsoid) SWZ1

Note: This feature currently has no reference values and should not be used.

The minimum volume enclosing ellipsoid (MVEE), unlike the approximate enclosing ellipsoid, is the smallest ellipsoid that encloses the ROI. Direct computation of the MVEE is usually unfeasible, and is therefore approximated. Various approximation algorithms have been described, e.g.[2,72], which are usually elaborations on Khachiyan's barycentric coordinate descent method[40].

The MVEE encloses the ROI mesh vertex set $\mathbf{X}_{vx}$, and by definition $\mathbf{X}_{vx,convex}$ as well. Use of the convex mesh set $\mathbf{X}_{vx,convex}$ is recommended due to its sparsity compared to the full vertex set. The volume of the MVEE is defined by its semi-axes lengths $V_{mvee} = 4\pi\, a\, b\, c/3$. Then:

$$F_{morph.v.dens.mvee} = \frac{V}{V_{mvee}}$$

For Khachiyan's barycentric coordinate descent-based methods we use a default tolerance $\tau = 0.001$ as stopping criterion.

3.1.24 Area density (minimum volume enclosing ellipsoid) BRI8

Note: This feature currently has no reference values and should not be used.

The surface area of an ellipsoid does not have a general elementary form, but should be approximated as noted in Section 3.1.22. Let the approximated surface area of the MVEE be A_{mvee}. Then:

$$F_{morph.a.dens.mvee} = \frac{A}{A_{mvee}}$$

3.1.25 Volume density (convex hull) R3ER

The convex hull encloses ROI mesh vertex set $\mathbf{X}_{vx}$ and consists of the vertex set $\mathbf{X}_{vx,convex}$ and corresponding faces, see section 3.1.11. The volume of the ROI mesh convex hull set V_{convex} is computed in the same way as that of the *volume (mesh)* feature (3.1.1). The *volume density* can then be calculated as follows:

$$F_{morph.v.dens.conv.hull} = \frac{V}{V_{convex}}$$

This feature is also called *solidity*[27,65].

data	value	tol.	consensus
dig. phantom	0.961	0.006	strong
config. A	0.827	0.001	moderate
config. B	0.829	0.001	moderate
config. C	0.834	0.002	moderate
config. D	0.834	0.002	moderate
config. E	0.834	0.002	moderate

Table 3.22 | Reference values for the *volume density (convex hull)* feature.

3.1.26 Area density (convex hull) 7T7F

The area of the convex hull A_{convex} is the sum of the areas of the faces of the convex hull, and is computed in the same way as the *surface area (mesh)* feature (section 3.1.3). The convex hull is identical to the one used in the *volume density (convex hull)* feature. Then:

$$F_{morph.a.dens.conv.hull} = \frac{A}{A_{convex}}$$

data	value	tol.	consensus
dig. phantom	1.03	0.01	strong
config. A	1.18	0.01	moderate
config. B	1.12	0.01	moderate
config. C	1.13	0.01	moderate
config. D	1.13	0.01	moderate
config. E	1.13	0.01	moderate

Table 3.23 | Reference values for the *area density (convex hull)* feature.

3.1.27 Integrated intensity 99N0

Integrated intensity is the average intensity in the ROI, multiplied by the volume. In the context of ^{18}F-FDG-PET, this feature is often called *total lesion glycolysis*[75]. Thus:

$$F_{morph.integ.int} = V \frac{1}{N_{v,gl}} \sum_{k=1}^{N_{v,gl}} X_{gl,k}$$

$N_{v,gl}$ is the number of voxels in the ROI intensity mask.

data	value	tol.	consensus
dig. phantom	1.2×10^3	10	moderate
config. A	4.81×10^6	3.2×10^5	strong
config. B	4.12×10^6	3.2×10^5	strong
config. C	-1.8×10^7	1.4×10^6	strong
config. D	-8.64×10^6	1.56×10^6	strong
config. E	-8.31×10^6	1.6×10^6	strong

Table 3.24 | Reference values for the *integrated intensity* feature.

3.1.28 Moran's I index N365

Moran's *I* index is an indicator of spatial autocorrelation[21,52]. It is defined as:

$$F_{morph.moran.i} = \frac{N_{v,gl}}{\sum_{k_1=1}^{N_{v,gl}} \sum_{k_2=1}^{N_{v,gl}} w_{k_1k_2}} \frac{\sum_{k_1=1}^{N_{v,gl}} \sum_{k_2=1}^{N_{v,gl}} w_{k_1k_2} \left(X_{gl,k_1} - \mu\right) \left(X_{gl,k_2} - \mu\right)}{\sum_{k=1}^{N_{v,gl}} \left(X_{gl,k} - \mu\right)^2}, \qquad k_1 \neq k_2$$

As before $N_{v,gl}$ is the number of voxels in the ROI intensity mask, μ is the mean of $\mathbf{X}_{gl}$ and $w_{k_1k_2}$ is a weight factor, equal to the inverse Euclidean distance between voxels k_1 and k_2

of the point set $\mathbf{X}_{c,gl}$ of the ROI intensity mask[20]. Values of Moran's *I* close to 1.0, 0.0 and -1.0 indicate high spatial autocorrelation, no spatial autocorrelation and high spatial anti-autocorrelation, respectively.

Note that for an ROI containing many voxels, calculating Moran's *I* index may be computationally expensive due to $O(n^2)$ behaviour. Approximation by repeated subsampling of the ROI may be required to make the calculation tractable, at the cost of accuracy.

data	value	tol.	consensus
dig. phantom	0.0397	0.0003	strong
config. A	0.0322	0.0002	moderate
config. B	0.0329	0.0001	moderate
config. C	0.0824	0.0003	moderate
config. D	0.0622	0.0013	moderate
config. E	0.0596	0.0014	moderate

Table 3.25 | Reference values for the *Moran's I index* feature.

3.1.29 Geary's C measure NPT7

Geary's *C* measure assesses spatial autocorrelation, similar to Moran's *I* index[21,31]. In contrast to Moran's *I* index, Geary's *C* measure directly assesses intensity differences between voxels and is more sensitive to local spatial autocorrelation. This measure is defined as:

$$F_{morph.geary.c} = \frac{N_{v,gl}-1}{2\sum_{k_1=1}^{N_{v,gl}}\sum_{k_2=1}^{N_{v,gl}} w_{k_1k_2}} \frac{\sum_{k_1=1}^{N_{v,gl}}\sum_{k_2=1}^{N_{v,gl}} w_{k_1k_2}\left(X_{gl,k_1}-X_{gl,k_2}\right)^2}{\sum_{k=1}^{N_{v,gl}}\left(X_{gl,k}-\mu\right)^2}, \qquad k_1 \neq k_2$$

As with Moran's *I*, $N_{v,gl}$ is the number of voxels in the ROI intensity mask, μ is the mean of $\mathbf{X}_{gl}$ and $w_{k_1k_2}$ is a weight factor, equal to the inverse Euclidean distance between voxels k_1 and k_2 of the ROI voxel point set $\mathbf{X}_{c,gl}$[20].

Just as Moran's *I*, Geary's *C* measure exhibits $O(n^2)$ behaviour and an approximation scheme may be required to make calculation feasible for large ROIs.

data	value	tol.	consensus
dig. phantom	0.974	0.006	strong
config. A	0.863	0.001	moderate
config. B	0.862	0.001	moderate
config. C	0.846	0.001	moderate
config. D	0.851	0.001	moderate
config. E	0.853	0.001	moderate

Table 3.26 | Reference values for the *Geary's C measure* feature.

3.2 Local intensity features 9ST6

Voxel intensities within a defined neighbourhood around a center voxel are used to compute local intensity features. Unlike many other feature sets, local features do not draw solely on intensities within the ROI. While only voxels within the ROI intensity map can be used as a center voxel, the local neighbourhood draws upon all voxels regardless of being in an ROI.

Aggregating features

By definition, local intensity features are calculated in 3D (DHQ4), and not per slice.

3.2.1 Local intensity peak VJGA

The *local intensity peak* was originally devised for reducing variance in determining standardised uptake values[85]. It is defined as the mean intensity in a 1 cm^3 spherical volume (in world coordinates), which is centered on the voxel with the maximum intensity level in the ROI intensity mask[29].

To calculate $F_{loc.peak.local}$, we first select all the voxels with centers within a radius $r = \left(\frac{3}{4\pi}\right)^{1/3} \approx 0.62$ cm of the center of the maximum intensity voxel. Subsequently, the mean intensity of the selected voxels, including the center voxel, are calculated.

In case the maximum intensity is found in multiple voxels within the ROI, *local intensity peak* is calculated for each of these voxels, and the highest *local intensity peak* is chosen.

data	value	tol.	consensus
dig. phantom	2.6	–	strong
config. A	−277	10	moderate
config. B	178	10	moderate
config. C	169	10	moderate
config. D	201	10	strong
config. E	181	13	moderate

Table 3.27 | Reference values for the *local intensity peak* feature.

3.2.2 Global intensity peak 0F91

The *global intensity peak* feature $F_{loc.peak.global}$ is similar to the *local intensity peak*[29]. However, instead of calculating the mean intensity for the voxel(s) with the maximum intensity, the mean intensity is calculated within a 1 cm^3 neighbourhood for every voxel in the ROI intensity mask. The highest intensity peak value is then selected.

Calculation of the *global intensity peak* feature may be accelerated by construction and application of an appropriate spatial spherical mean convolution filter, due to the convolution theorem. In this case one would first construct an empty 3D filter that will fit a 1 cm^3 sphere. Within this context, the filter voxels may be represented by a point set, akin to $\mathbf{X}_c$ in section 3.1. Euclidean distances in world spacing between the central voxel of the filter and every remaining voxel are computed. If this distance lies within radius $r = \left(\frac{3}{4\pi}\right)^{1/3} \approx 0.62$ the corresponding voxel receives a label 1, and 0 otherwise. Subsequent summation of the

voxel labels yields N_s, the number of voxels within the 1 cm^3 sphere. The filter then becomes a spherical mean filter by dividing the labels by N_s.

data	value	tol.	consensus
dig. phantom	3.1	–	strong
config. A	189	5	moderate
config. B	178	5	moderate
config. C	180	5	moderate
config. D	201	5	moderate
config. E	181	5	moderate

Table 3.28 | Reference values for the *global intensity peak* feature.

3.3 Intensity-based statistical features UHIW

The intensity-based statistical features describe how intensities within the region of interest (ROI) are distributed. The features in this set do not require discretisation, and may be used to describe a continuous intensity distribution. Intensity-based statistical features are not meaningful if the intensity scale is arbitrary.

The set of intensities of the N_v voxels included in the ROI intensity mask is denoted as $\mathbf{X}_{gl} = \left\{X_{gl,1}, X_{gl,2}, \ldots, X_{gl,N_v}\right\}$.

Aggregating features

We recommend calculating intensity-based statistical features using the 3D volume (DHQ4). An approach that computes intensity-based statistical features per slice and subsequently averages them (3IDG) is not recommended.

3.3.1 Mean intensity Q4LE

The *mean intensity* of $\mathbf{X}_{gl}$ is calculated as:

$$F_{stat.mean} = \frac{1}{N_v}\sum_{k=1}^{N_v} X_{gl,k}$$

data	value	tol.	consensus
dig. phantom	2.15	–	very strong
config. A	13.4	1.1	very strong
config. B	11.5	1.1	strong
config. C	−49	2.9	very strong
config. D	−23.5	3.9	strong
config. E	−22.6	4.1	strong

Table 3.29 | Reference values for the *mean* feature.

3.3.2 Intensity variance ECT3

The *intensity variance* of $\mathbf{X}_{gl}$ is defined as:

$$F_{stat.var} = \frac{1}{N_v}\sum_{k=1}^{N_v} \left(X_{gl,k} - \mu\right)^2$$

Note that we do not apply a bias correction when computing the variance.

data	value	tol.	consensus
dig. phantom	3.05	–	very strong
config. A	1.42×10^4	400	very strong
config. B	1.44×10^4	400	strong
config. C	5.06×10^4	1.4×10^3	very strong
config. D	3.28×10^4	2.1×10^3	strong
config. E	3.51×10^4	2.2×10^3	strong

Table 3.30 | Reference values for the *variance* feature.

3.3.3 Intensity skewness

KE2A

The *skewness* of the intensity distribution of $\mathbf{X}_{gl}$ is defined as:

$$F_{stat.skew} = \frac{\frac{1}{N_v}\sum_{k=1}^{N_v}\left(X_{gl,k}-\mu\right)^3}{\left(\frac{1}{N_v}\sum_{k=1}^{N_v}\left(X_{gl,k}-\mu\right)^2\right)^{3/2}}$$

Here $\mu = F_{stat.mean}$. If the *intensity variance* $F_{stat.var} = 0$, $F_{stat.skew} = 0$.

data	value	tol.	consensus
dig. phantom	1.08	–	very strong
config. A	−2.47	0.05	very strong
config. B	−2.49	0.05	strong
config. C	−2.14	0.05	very strong
config. D	−2.28	0.06	strong
config. E	−2.3	0.07	strong

Table 3.31 | Reference values for the *skewness* feature.

3.3.4 (Excess) intensity kurtosis

IPH6

Kurtosis, or technically excess kurtosis, is a measure of peakedness in the intensity distribution $\mathbf{X}_{gl}$:

$$F_{stat.kurt} = \frac{\frac{1}{N_v}\sum_{k=1}^{N_v}\left(X_{gl,k}-\mu\right)^4}{\left(\frac{1}{N_v}\sum_{k=1}^{N_v}\left(X_{gl,k}-\mu\right)^2\right)^{2}} - 3$$

Here $\mu = F_{stat.mean}$. Note that kurtosis is corrected by a Fisher correction of -3 to center it on 0 for normal distributions. If the *intensity variance* $F_{stat.var} = 0$, $F_{stat.kurt} = 0$.

data	value	tol.	consensus
dig. phantom	−0.355	—	very strong
config. A	5.96	0.24	very strong
config. B	5.93	0.24	strong
config. C	3.53	0.23	very strong
config. D	4.35	0.32	strong
config. E	4.44	0.33	strong

Table 3.32 | Reference values for the *(excess) kurtosis* feature.

3.3.5 Median intensity Y12H

The *median intensity* $F_{stat.median}$ is the sample median of $\mathbf{X}_{gl}$.

data	value	tol.	consensus
dig. phantom	1	—	very strong
config. A	46	0.3	very strong
config. B	45	0.3	strong
config. C	40	0.4	very strong
config. D	42	0.4	strong
config. E	43	0.5	strong

Table 3.33 | Reference values for the *median* feature.

3.3.6 Minimum intensity 1GSF

The *minimum intensity* is equal to the lowest intensity present in $\mathbf{X}_{gl}$, i.e:

$$F_{stat.min} = \min(\mathbf{X}_{gl})$$

data	value	tol.	consensus
dig. phantom	1	—	very strong
config. A	−500	—	very strong
config. B	−500	—	strong
config. C	−939	4	very strong
config. D	−724	12	strong
config. E	−743	13	strong

Table 3.34 | Reference values for the *minimum* feature.

3.3.7 10th intensity percentile QG58

P_{10} is the 10th percentile of $\mathbf{X}_{gl}$. P_{10} is a more robust alternative to the *minimum intensity*.

data	value	tol.	consensus
dig. phantom	1	—	very strong
config. A	−129	8	very strong
config. B	−136	8	strong
config. C	−424	14	very strong
config. D	−304	20	strong
config. E	−310	21	strong

Table 3.35 | Reference values for the *10th percentile* feature.

3.3.8 90th intensity percentile 8DWT

P_{90} is the 90th percentile of $\mathbf{X}_{gl}$. P_{90} is a more robust alternative to the *maximum intensity*.

data	value	tol.	consensus
dig. phantom	4	—	very strong
config. A	95	—	strong
config. B	91	—	strong
config. C	86	0.1	strong
config. D	86	0.1	strong
config. E	93	0.2	strong

Table 3.36 | Reference values for the *90th percentile* feature.

Note that the *90th intensity percentile* obtained for the digital phantom may differ from the above reference value depending on the software implementation used to compute it. For example, some implementations were found to produce a value of 4.2 instead of 4.

3.3.9 Maximum intensity 84IY

The *maximum intensity* is equal to the highest intensity present in $\mathbf{X}_{gl}$, i.e:

$$F_{stat.max} = \max(\mathbf{X}_{gl})$$

data	value	tol.	consensus
dig. phantom	6	—	very strong
config. A	377	9	very strong
config. B	391	9	strong
config. C	393	10	very strong
config. D	521	22	strong
config. E	345	9	strong

Table 3.37 | Reference values for the *maximum* feature.

3.3.10 Intensity interquartile range SAL0

The *interquartile range* (IQR) of $\mathbf{X}_{gl}$ is defined as:

$$F_{stat.iqr} = P_{75} - P_{25}$$

P_{25} and P_{75} are the 25th and 75th percentiles of $\mathbf{X}_{gl}$, respectively.

data	value	tol.	consensus
dig. phantom	3	–	very strong
config. A	56	0.5	very strong
config. B	52	0.5	strong
config. C	67	4.9	very strong
config. D	57	4.1	strong
config. E	62	3.5	strong

Table 3.38 | Reference values for the *interquartile range* feature.

3.3.11 Intensity range 2OJQ

The *intensity range* is defined as:

$$F_{stat.range} = \max(\mathbf{X}_{gl}) - \min(\mathbf{X}_{gl})$$

data	value	tol.	consensus
dig. phantom	5	–	very strong
config. A	877	9	very strong
config. B	891	9	strong
config. C	1.33×10^3	20	very strong
config. D	1.24×10^3	40	strong
config. E	1.09×10^3	30	strong

Table 3.39 | Reference values for the *range* feature.

3.3.12 Intensity-based mean absolute deviation 4FUA

Mean absolute deviation is a measure of dispersion from the mean of $\mathbf{X}_{gl}$:

$$F_{stat.mad} = \frac{1}{N_v} \sum_{k=1}^{N_v} \left| X_{gl,k} - \mu \right|$$

Here $\mu = F_{stat.mean}$.

data	value	tol.	consensus
dig. phantom	1.55	—	very strong
config. A	73.6	1.4	very strong
config. B	74.4	1.4	strong
config. C	158	4	very strong
config. D	123	6	strong
config. E	125	6	strong

Table 3.40 | Reference values for the *mean absolute deviation* feature.

3.3.13 Intensity-based robust mean absolute deviation 1128

The *intensity-based mean absolute deviation* feature may be influenced by outliers. To increase robustness, the set of intensities can be restricted to those which lie closer to the center of the distribution. Let

$$\mathbf{X}_{gl,10-90} = \left\{x \in \mathbf{X}_{gl} | P_{10}\left(\mathbf{X}_{gl}\right) \leq x \leq P_{90}\left(\mathbf{X}_{gl}\right)\right\}$$

Then $\mathbf{X}_{gl,10-90}$ is the set of $N_{v,10-90} \leq N_v$ voxels in $\mathbf{X}_{gl}$ whose intensities fall in the interval bounded by the 10th and 90th percentiles of $\mathbf{X}_{gl}$. The robust mean absolute deviation is then:

$$F_{stat.rmad} = \frac{1}{N_{v,10-90}} \sum_{k=1}^{N_{v,10-90}} \left|X_{gl,10-90,k} - \overline{X}_{gl,10-90}\right|$$

$\overline{X}_{gl,10-90}$ denotes the sample mean of $\mathbf{X_{gl,10-90}}$.

data	value	tol.	consensus
dig. phantom	1.11	—	very strong
config. A	27.7	0.8	very strong
config. B	27.3	0.8	strong
config. C	66.8	3.5	very strong
config. D	46.8	3.6	strong
config. E	46.5	3.7	strong

Table 3.41 | Reference values for the *robust mean absolute deviation* feature.

3.3.14 Intensity-based median absolute deviation N72L

Median absolute deviation is similar in concept to the *intensity-based mean absolute deviation*, but measures dispersion from the median intensity instead of the mean intensity. Thus:

$$F_{stat.medad} = \frac{1}{N_v} \sum_{k=1}^{N_v} \left|X_{gl,k} - M\right|$$

Here, median $M = F_{stat.median}$.

data	value	tol.	consensus
dig. phantom	1.15	−	very strong
config. A	64.3	1	strong
config. B	63.8	1	strong
config. C	119	4	strong
config. D	94.7	3.8	strong
config. E	97.9	3.9	strong

Table 3.42 | Reference values for the *median absolute deviation* feature.

3.3.15 Intensity-based coefficient of variation 7TET

The *coefficient of variation* measures the dispersion of $\mathbf{X}_{gl}$. It is defined as:

$$F_{stat.cov} = \frac{\sigma}{\mu}$$

Here $\sigma = {F_{stat.var}}^{1/2}$ and $\mu = F_{stat.mean}$ are the standard deviation and mean of the intensity distribution, respectively.

data	value	tol.	consensus
dig. phantom	0.812	−	very strong
config. A	8.9	4.98	strong
config. B	10.4	5.2	strong
config. C	−4.59	0.29	strong
config. D	−7.7	1.01	strong
config. E	−8.28	0.95	strong

Table 3.43 | Reference values for the *coefficient of variation* feature.

3.3.16 Intensity-based quartile coefficient of dispersion 9S40

The *quartile coefficient of dispersion* is a more robust alternative to the *intensity-based coefficient of variance*. It is defined as:

$$F_{stat.qcod} = \frac{P_{75} - P_{25}}{P_{75} + P_{25}}$$

P_{25} and P_{75} are the 25th and 75th percentile of $\mathbf{X}_{gl}$, respectively.

data	value	tol.	consensus
dig. phantom	0.6	−	very strong
config. A	0.636	0.008	strong
config. B	0.591	0.008	strong
config. C	1.03	0.4	strong
config. D	0.74	0.011	strong
config. E	0.795	0.337	strong

Table 3.44 | Reference values for the *quartile coefficient of dispersion* feature.

3.3.17 Intensity-based energy N8CA

The *energy*[1] of $\mathbf{X}_{gl}$ is defined as:

$$F_{stat.energy} = \sum_{k=1}^{N_v} X_{gl,k}^2$$

data	value	tol.	consensus
dig. phantom	567	–	very strong
config. A	1.65×10^9	2×10^7	very strong
config. B	3.98×10^8	1.1×10^7	strong
config. C	2.44×10^9	1.2×10^8	strong
config. D	1.48×10^9	1.4×10^8	strong
config. E	1.58×10^9	1.4×10^8	strong

Table 3.45 | Reference values for the *energy* feature.

3.3.18 Root mean square intensity 5ZWQ

The *root mean square intensity* feature[1], which is also called the *quadratic mean*, of $\mathbf{X}_{gl}$ is defined as:

$$F_{stat.rms} = \sqrt{\frac{\sum_{k=1}^{N_v} X_{gl,k}^2}{N_v}}$$

data	value	tol.	consensus
dig. phantom	2.77	–	very strong
config. A	120	2	very strong
config. B	121	2	strong
config. C	230	4	strong
config. D	183	7	strong
config. E	189	7	strong

Table 3.46 | Reference values for the *root mean square* feature.

3.4 Intensity histogram features ZVCW

An intensity histogram is generated by discretising the original intensity distribution $\mathbf{X}_{gl}$ into intensity bins. Approaches to discretisation are described in Section 2.7.

Let $\mathbf{X}_d = \{X_{d,1}, X_{d,2}, \dots, X_{d,N_v}\}$ be the set of N_g discretised intensities of the N_v voxels in the ROI intensity mask. Let $\mathbf{H} = \{n_1, n_2, \dots, n_{N_g}\}$ be the histogram with frequency count n_i of each discretised intensity i in $\mathbf{X}_d$. The occurrence probability p_i for each discretised intensity i is then approximated as $p_i = n_i/N_v$.

Aggregating features

We recommend calculating intensity histogram features using the 3D volume (DHQ4). An approach that computes features per slice and subsequently averages (3IDG) is not recommended.

3.4.1 Mean discretised intensity X6K6

The *mean*[1] of $\mathbf{X}_d$ is calculated as:

$$F_{ih.mean} = \frac{1}{N_v}\sum_{k=1}^{N_v} X_{d,k}$$

An equivalent definition is:

$$F_{ih.mean} = \sum_{i=1}^{N_g} i p_i$$

data	value	tol.	consensus
dig. phantom	2.15	—	very strong
config. A	21.1	0.1	strong
config. B	18.9	0.3	strong
config. C	38.6	0.2	strong
config. D	18.5	0.5	strong
config. E	21.7	0.3	strong

Table 3.47 | Reference values for the *mean* feature.

3.4.2 Discretised intensity variance CH89

The *variance*[1] of $\mathbf{X}_d$ is defined as:

$$F_{ih.var} = \frac{1}{N_v}\sum_{k=1}^{N_v} \left(X_{d,k} - \mu\right)^2$$

Here $\mu = F_{ih.mean}$. This definition is equivalent to:

$$F_{ih.var} = \sum_{i=1}^{N_g} (i - \mu)^2 p_i$$

Note that no bias-correction is applied when computing the variance.

data	value	tol.	consensus
dig. phantom	3.05	—	strong
config. A	22.8	0.6	strong
config. B	18.7	0.2	strong
config. C	81.1	2.1	strong
config. D	21.7	0.4	strong
config. E	30.4	0.8	strong

Table 3.48 | Reference values for the *variance* feature.

3.4.3 Discretised intensity skewness 88K1

The *skewness*[1] of $\mathbf{X}_d$ is defined as:

$$F_{ih.skew} = \frac{\frac{1}{N_v}\sum_{k=1}^{N_v}\left(X_{d,k}-\mu\right)^3}{\left(\frac{1}{N_v}\sum_{k=1}^{N_v}\left(X_{d,k}-\mu\right)^2\right)^{3/2}}$$

Here $\mu = F_{ih.mean}$. This definition is equivalent to:

$$F_{ih.skew} = \frac{\sum_{i=1}^{N_g}\left(i-\mu\right)^3 p_i}{\left(\sum_{i=1}^{N_g}\left(i-\mu\right)^2 p_i\right)^{3/2}}$$

If the *discretised intensity variance* $F_{ih.var} = 0$, $F_{ih.skew} = 0$.

data	value	tol.	consensus
dig. phantom	1.08	—	very strong
config. A	−2.46	0.05	strong
config. B	−2.47	0.05	strong
config. C	−2.14	0.05	strong
config. D	−2.27	0.06	strong
config. E	−2.29	0.07	strong

Table 3.49 | Reference values for the *skewness* feature.

3.4.4 (Excess) discretised intensity kurtosis C3I7

Kurtosis[1], or technically excess kurtosis, measures the peakedness of the $\mathbf{X}_d$ distribution:

$$F_{ih.kurt} = \frac{\frac{1}{N_v}\sum_{k=1}^{N_v}\left(X_{d,k}-\mu\right)^4}{\left(\frac{1}{N_v}\sum_{k=1}^{N_v}\left(X_{d,k}-\mu\right)^2\right)^{2}} - 3$$

Here $\mu = F_{ih.mean}$. An alternative, but equivalent, definition is:

$$F_{ih.kurt} = \frac{\sum_{i=1}^{N_g}\left(i-\mu\right)^4 p_i}{\left(\sum_{i=1}^{N_g}\left(i-\mu\right)^2 p_i\right)^{2}} - 3$$

Note that kurtosis is corrected by a Fisher correction of -3 to center kurtosis on 0 for normal distributions. If the *discretised intensity variance* $F_{ih.var} = 0$, $F_{ih.kurt} = 0$.

data	value	tol.	consensus
dig. phantom	−0.355	—	very strong
config. A	5.9	0.24	strong
config. B	5.84	0.24	strong
config. C	3.52	0.23	strong
config. D	4.31	0.32	strong
config. E	4.4	0.33	strong

Table 3.50 | Reference values for the *(excess) kurtosis* feature.

3.4.5 Median discretised intensity WIFQ

The *median* $F_{ih.median}$ is the sample median of $\mathbf{X}_d$[1].

data	value	tol.	consensus
dig. phantom	1	—	very strong
config. A	22	—	strong
config. B	20	0.3	strong
config. C	42	—	strong
config. D	20	0.5	strong
config. E	24	0.2	strong

Table 3.51 | Reference values for the *median* feature.

3.4.6 Minimum discretised intensity 1PR8

The *minimum discretised intensity*[1] is equal to the lowest discretised intensity present in $\mathbf{X}_d$, i.e.:

$$F_{ih.min} = \min(\mathbf{X}_d)$$

For *fixed bin number* discretisation $F_{ih.min} = 1$ by definition, but $F_{ih.min} > 1$ is possible for *fixed bin size* discretisation.

data	value	tol.	consensus
dig. phantom	1	—	very strong
config. A	1	—	strong
config. B	1	—	strong
config. C	3	0.16	strong
config. D	1	—	strong
config. E	1	—	strong

Table 3.52 | Reference values for the *minimum* feature.

3.4.7 10th discretised intensity percentile GPMT

P_{10} is the 10th percentile of $\mathbf{X}_d$.

data	value	tol.	consensus
dig. phantom	1	–	very strong
config. A	15	0.4	strong
config. B	14	0.5	strong
config. C	24	0.7	strong
config. D	11	0.7	strong
config. E	13	0.7	strong

Table 3.53 | Reference values for the *10th percentile* feature.

3.4.8 90th discretised intensity percentile OZ0C

P_{90} is the 90th percentile of $\mathbf{X}_d$ and is defined as $F_{ih.P90}$.

data	value	tol.	consensus
dig. phantom	4	–	strong
config. A	24	–	strong
config. B	22	0.3	strong
config. C	44	–	strong
config. D	21	0.5	strong
config. E	25	0.2	strong

Table 3.54 | Reference values for the *90th percentile* feature.

Note that the *90th discretised intensity percentile* obtained for the digital phantom may differ from the above reference value depending on the software implementation used to compute it. For example, some implementations were found to produce a value of 4.2 instead of 4 for this feature.

3.4.9 Maximum discretised intensity 3NCY

The *maximum discretised intensity*[1] is equal to the highest discretised intensity present in $\mathbf{X}_d$, i.e.:

$$F_{ih.max} = \max(\mathbf{X}_d)$$

By definition, $F_{ih.max} = N_g$.

data	value	tol.	consensus
dig. phantom	6	—	very strong
config. A	36	0.4	strong
config. B	32	—	strong
config. C	56	0.5	strong
config. D	32	—	strong
config. E	32	—	strong

Table 3.55 | Reference values for the *maximum* feature.

3.4.10 Intensity histogram mode AMMC

The *mode* of $\mathbf{X}_d$ $F_{ih.mode}$ is the most common discretised intensity present, i.e. the value i for with the highest count n_i. The mode may not be uniquely defined. When the highest count is found in multiple bins, the value i of the bin closest to the *mean discretised intensity* is chosen as *intensity histogram mode*. In pathological cases with two such bins equidistant to the mean, the bin to the left of the mean is selected.

data	value	tol.	consensus
dig. phantom	1	—	very strong
config. A	23	—	strong
config. B	20	0.3	strong
config. C	43	0.1	strong
config. D	20	0.4	strong
config. E	24	0.1	strong

Table 3.56 | Reference values for the *mode* feature.

3.4.11 Discretised intensity interquartile range WR0O

The *interquartile range* (IQR) of $\mathbf{X}_d$ is defined as:

$$F_{ih.iqr} = P_{75} - P_{25}$$

P_{25} and P_{75} are the 25th and 75th percentile of $\mathbf{X}_d$, respectively.

data	value	tol.	consensus
dig. phantom	3	—	very strong
config. A	2	—	strong
config. B	2	—	strong
config. C	3	0.21	strong
config. D	2	0.06	strong
config. E	1	0.06	strong

Table 3.57 | Reference values for the *interquartile range* feature.

3.4.12 Discretised intensity range 5Z3W

The *discretised intensity range*[1] is defined as:

$$F_{ih.range} = \max(\mathbf{X}_d) - \min(\mathbf{X}_d)$$

For *fixed bin number* discretisation, the *discretised intensity range* equals N_g by definition.

data	value	tol.	consensus
dig. phantom	5	—	very strong
config. A	35	0.4	strong
config. B	31	—	strong
config. C	53	0.6	strong
config. D	31	—	strong
config. E	31	—	strong

Table 3.58 | Reference values for the *range* feature.

3.4.13 Intensity histogram mean absolute deviation D2ZX

The *mean absolute deviation*[1] is a measure of dispersion from the mean of $\mathbf{X}_d$:

$$F_{ih.mad} = \frac{1}{N_v} \sum_{i=1}^{N_v} |X_{d,i} - \mu|$$

Here $\mu = F_{ih.mean}$.

data	value	tol.	consensus
dig. phantom	1.55	—	very strong
config. A	2.94	0.06	strong
config. B	2.67	0.03	strong
config. C	6.32	0.15	strong
config. D	3.15	0.05	strong
config. E	3.69	0.1	strong

Table 3.59 | Reference values for the *mean absolute deviation* feature.

3.4.14 Intensity histogram robust mean absolute deviation WRZB

Intensity histogram mean absolute deviation may be affected by outliers. To increase robustness, the set of discretised intensities under consideration can be restricted to those which are closer to the center of the distribution. Let

$$\mathbf{X}_{d,10-90} = \{x \in \mathbf{X}_d | P_{10}(\mathbf{X}_d) \leq x \leq P_{90}(\mathbf{X}_d)\}$$

In short, $\mathbf{X}_{d,10-90}$ is the set of $N_{v,10-90} \leq N_v$ voxels in $\mathbf{X}_d$ whose discretised intensities fall in the interval bounded by the 10th and 90th percentiles of $\mathbf{X}_d$. The robust mean absolute

deviation is then:

$$F_{ih.rmad} = \frac{1}{N_{v,10-90}} \sum_{k=1}^{N_{v,10-90}} \left| X_{d,10-90,k} - \overline{X}_{d,10-90} \right|$$

$\overline{X}_{d,10-90}$ denotes the sample mean of $\mathbf{X}_{d,10-90}$.

data	value	tol.	consensus
dig. phantom	1.11	—	very strong
config. A	1.18	0.04	strong
config. B	1.03	0.03	moderate
config. C	2.59	0.14	strong
config. D	1.33	0.06	strong
config. E	1.46	0.09	moderate

Table 3.60 | Reference values for the *robust mean absolute deviation* feature.

3.4.15 Intensity histogram median absolute deviation 4RNL

Histogram median absolute deviation is conceptually similar to *histogram mean absolute deviation*, but measures dispersion from the median instead of mean. Thus:

$$F_{ih.medad} = \frac{1}{N_v} \sum_{k=1}^{N_v} |X_{d,k} - M|$$

Here, median $M = F_{ih.median}$.

data	value	tol.	consensus
dig. phantom	1.15	—	very strong
config. A	2.58	0.05	strong
config. B	2.28	0.02	strong
config. C	4.75	0.12	strong
config. D	2.41	0.04	strong
config. E	2.89	0.07	strong

Table 3.61 | Reference values for the *median absolute deviation* feature.

3.4.16 Intensity histogram coefficient of variation CWYJ

The *coefficient of variation* measures the dispersion of the discretised intensity distribution. It is defined as:

$$F_{ih.cov} = \frac{\sigma}{\mu}$$

Here $\sigma = F_{ih.var}{}^{1/2}$ and $\mu = F_{ih.mean}$ are the standard deviation and mean of the discretised intensity distribution, respectively.

data	value	tol.	consensus
dig. phantom	0.812	–	very strong
config. A	0.227	0.004	strong
config. B	0.229	0.004	strong
config. C	0.234	0.005	strong
config. D	0.252	0.006	strong
config. E	0.254	0.006	strong

Table 3.62 | Reference values for the *coefficient of variation* feature.

3.4.17 Intensity histogram quartile coefficient of dispersion SLWD

The *quartile coefficient of dispersion* is a more robust alternative to the *intensity histogram coefficient of variance*. It is defined as:

$$F_{ih.qcod} = \frac{P_{75} - P_{25}}{P_{75} + P_{25}}$$

P_{25} and P_{75} are the 25th and 75th percentile of $\mathbf{X}_d$, respectively.

data	value	tol.	consensus
dig. phantom	0.6	–	very strong
config. A	0.0455	–	strong
config. B	0.05	0.0005	strong
config. C	0.0361	0.0027	strong
config. D	0.05	0.0021	strong
config. E	0.0213	0.0015	strong

Table 3.63 | Reference values for the *quartile coefficient of dispersion* feature.

3.4.18 Discretised intensity entropy TLU2

Entropy[1] is an information-theoretic concept that gives a metric for the information contained within $\mathbf{X}_d$. The particular metric used is Shannon entropy, which is defined as:

$$F_{ih.entropy} = -\sum_{i=1}^{N_g} p_i \log_2 p_i$$

Note that *entropy* can only be meaningfully defined for discretised intensities as it will tend to $-\log_2 N_v$ for continuous intensity distributions.

data	value	tol.	consensus
dig. phantom	1.27	—	very strong
config. A	3.36	0.03	very strong
config. B	3.16	0.01	strong
config. C	3.73	0.04	strong
config. D	2.94	0.01	strong
config. E	3.22	0.02	strong

Table 3.64 | Reference values for the *entropy* feature.

3.4.19 Discretised intensity uniformity

BJ5W

Uniformity[1] of $\mathbf{X}_d$ is defined as:

$$F_{ih.uniformity} = \sum_{i=1}^{N_g} p_i^2$$

For histograms where most intensities are contained in a single bin, *uniformity* approaches 1. The lower bound is $1/N_g$.

Note that this feature is sometimes referred to as *energy*.

data	value	tol.	consensus
dig. phantom	0.512	—	very strong
config. A	0.15	0.002	very strong
config. B	0.174	0.001	strong
config. C	0.14	0.003	strong
config. D	0.229	0.003	strong
config. E	0.184	0.001	strong

Table 3.65 | Reference values for the *uniformity* feature.

3.4.20 Maximum histogram gradient

12CE

The histogram gradient $\mathbf{H}'$ of intensity histogram $\mathbf{H}$ can be calculated as:

$$H_i' = \begin{cases} n_2 - n_1 & i = 1 \\ (n_{i+1} - n_{i-1})/2 & 1 < i < N_g \\ n_{N_g} - n_{N_g - 1} & i = N_g \end{cases}$$

Histogram $\mathbf{H}$ should be non-sparse, i.e. bins where $n_i = 0$ should not be omitted. Ostensibly, the histogram gradient can be calculated in different ways. The method above has the advantages of being easy to implement and leading to a gradient $\mathbf{H}'$ with same size as $\mathbf{H}$. This helps maintain a direct correspondence between the discretised intensities in $\mathbf{H}$ and the bins of $\mathbf{H}'$. The *maximum histogram gradient*[80] is:

$$F_{ih.max.grad} = \max(\mathbf{H}')$$

data	value	tol.	consensus
dig. phantom	8	—	very strong
config. A	1.1×10^4	100	strong
config. B	3.22×10^3	50	strong
config. C	4.75×10^3	30	strong
config. D	7.26×10^3	200	strong
config. E	6.01×10^3	130	strong

Table 3.66 | Reference values for the *maximum histogram gradient* feature.

3.4.21 Maximum histogram gradient intensity 8E60

The *maximum histogram gradient intensity*[80] $F_{ih.max.grad.gl}$ is the discretised intensity corresponding to the *maximum histogram gradient*, i.e. the value i in $\mathbf{H}$ for which $\mathbf{H}'$ is maximal.

data	value	tol.	consensus
dig. phantom	3	—	strong
config. A	21	—	strong
config. B	19	0.3	strong
config. C	41	—	strong
config. D	19	0.4	strong
config. E	23	0.2	moderate

Table 3.67 | Reference values for the *maximum histogram gradient intensity* feature.

3.4.22 Minimum histogram gradient VQB3

The *minimum histogram gradient*[80] is:

$$F_{ih.min.grad} = \min\left(\mathbf{H}'\right)$$

data	value	tol.	consensus
dig. phantom	−50	—	very strong
config. A	-1.01×10^4	100	strong
config. B	-3.02×10^3	50	strong
config. C	-4.68×10^3	50	strong
config. D	-6.67×10^3	230	strong
config. E	-6.11×10^3	180	strong

Table 3.68 | Reference values for the *minimum histogram gradient* feature.

3.4.23 Minimum histogram gradient intensity RHQZ

The *minimum histogram gradient intensity*[80] $F_{ih.min.grad.gl}$ is the discretised intensity corresponding to the *minimum histogram gradient*, i.e. the value i in $\mathbf{H}$ for which $\mathbf{H}'$ is minimal.

data	value	tol.	consensus
dig. phantom	1	–	strong
config. A	24	–	strong
config. B	22	0.3	strong
config. C	44	–	strong
config. D	22	0.4	strong
config. E	25	0.2	strong

Table 3.69 | Reference values for the *minimum histogram gradient intensity* feature.

3.5 Intensity-volume histogram features P88C

The (cumulative) intensity-volume histogram (IVH) of the set $\mathbf{X}_{gl}$ of voxel intensities in the ROI intensity mask describes the relationship between discretised intensity i and the fraction of the volume containing at least intensity i, ν [27].

Depending on the imaging modality, the calculation of IVH features requires discretising $\mathbf{X}_{gl}$ to generate a new voxel set $\mathbf{X}_{d,gl}$ with discretised intensities. Moreover, the total range $\mathbf{G}$ of discretised intensities and the discretisation interval w_d should be provided or determined. The total range $\mathbf{G}$ determines the range of discretised intensities to be included in the IVH, whereas the discretisation interval determines the intensity difference between adjacent discretised intensities in the IVH.

Recommendations for discretisation parameters differ depending on what type of data the image represents, and how it is represented. These recommendations are described below.

Discrete calibrated image intensities

Some imaging modalities by default generate voxels with calibrated, discrete intensities – for example CT. In this case, the discretised ROI voxel set $\mathbf{X}_{d,gl} = \mathbf{X}_{gl}$, i.e. no discretisation required. If a re-segmentation range is provided (see Section 2.5), the total range $\mathbf{G}$ is equal to the re-segmentation range. In the case of a half-open re-segmentation range, the upper limit of the range is $\max(\mathbf{X}_{gl})$. When no range is provided, $\mathbf{G} = [\min(\mathbf{X}_{gl}), \max(\mathbf{X}_{gl})]$. The discretisation interval is $w_d = 1$.

Continuous calibrated image intensities

Imaging with calibrated, continuous intensities such as PET requires discretisation to determine the IVH, while preserving the quantitative intensity information. The use of a *fixed bin size* discretisation method is thus recommended, see Section 2.7. This method requires a minimum intensity $X_{gl,min}$, a maximum intensity $X_{gl,max}$ and the bin width w_b. If a re-segmentation range is defined (see Section 2.5), $X_{gl,min}$ is set to the lower bound of the re-segmentation range and $X_{gl,max}$ to the upper bound; otherwise $X_{gl,min} = \min(\mathbf{X}_{gl})$ and $X_{gl,max} = \max(\mathbf{X}_{gl})$ (i.e. the minimum and maximum intensities in the imaging volume prior to discretisation). The bin width w_b is modality dependent, but should be small relative to the intensity range, e.g. 0.10 SUV for ^{18}F-FDG-PET.

Next, *fixed bin size* discretisation produces the voxel set $\mathbf{X}_d$ of bin numbers, which needs to be converted to bin centers in order to maintain a direct relationship with the original intensities. We thus replace bin numbers $\mathbf{X}_d$ with the intensity corresponding to the bin center:

$$\mathbf{X}_{d,gl} = X_{gl,min} + (\mathbf{X}_d - 0.5)\, w_b$$

The total range is then $\mathbf{G} = [X_{gl,min} + 0.5w_b, X_{gl,max} - 0.5w_b]$. In this case, the discretisation interval matches the bin width, i.e. $w_d = w_b$.

Arbitrary intensity units

Some imaging modalities, such as many MRI sequences, produce arbitrary intensities. In such cases, a *fixed bin number* discretisation method with $N_g = 1000$ bins is recommended, see Section 2.7. The discretisation bin width is $w_b = \left(X_{gl,max} - X_{gl,min}\right)/N_g$, with $X_{gl,max} = \max\left(\mathbf{X}_{gl}\right)$ and $X_{gl,min} = \min\left(\mathbf{X}_{gl}\right)$, as re-segmentation ranges generally cannot be provided for non-calibrated intensities. The *fixed bin number* discretisation produces

i	γ	ν
1	0.0	1.000
2	0.2	0.324
3	0.4	0.324
4	0.6	0.311
5	0.8	0.095
6	1.0	0.095

Table 3.70 | Example intensity-volume histogram evaluated at discrete intensities i of the digital phantom. The total range $\mathbf{G} = [1, 6]$, with discretisation interval $w = 1$. Thus γ is the intensity fraction and ν is the corresponding volume fraction that contains intensity i or greater.

the voxel set $\mathbf{X}_d \in \{1, 2, \dots, N_g\}$. Because of the lack of calibration, $\mathbf{X}_{d,gl} = \mathbf{X}_d$, and consequentially the discretisation interval is $w_d = 1$ and the total range is $\mathbf{G} = [1, N_g]$

Calculating the IV histogram

We use $\mathbf{X}_{d,gl}$ to calculate fractional volumes and fractional intensities.

As voxels for the same image stack generally all have the same dimensions, we may define fractional volume ν for discretised intensity i:

$$\nu_i = 1 - \frac{1}{N_v} \sum_{k=1}^{N_v} \left[X_{d,gl,k} < i\right]$$

Here $[\dots]$ is an Iverson bracket, yielding 1 if the condition is true and 0 otherwise. In essence, we count the voxels containing a discretised intensity smaller than i, divide by the total number of voxels, and then subtract this volume fraction to find ν_i.

The intensity fraction γ for discretised intensity i in the range $\mathbf{G}$ is calculated as:

$$\gamma_i = \frac{i - \min(\mathbf{G})}{\max(\mathbf{G}) - \min(\mathbf{G})}$$

Note that intensity fractions are also calculated for discretised intensities that are absent in $\mathbf{X}_{d,gl}$. For example intensities 2 and 5 are absent in the digital phantom (see Chapter 5), but are still evaluated to determine both the fractional volume and the intensity fraction. An example IVH for the digital phantom is shown in Table 3.70.

Aggregating features

We recommend calculating intensity-volume histogram features using the 3D volume (DHQ4). Computing features per slice and subsequently averaging (3IDG) is not recommended.

3.5.1 Volume at intensity fraction BC2M

The *volume at intensity fraction* V_x is the largest volume fraction ν that has an intensity fraction γ of at least $x\%$. This differs from conceptually similar dose-volume histograms used in radiotherapy planning, where V_{10} would indicate the volume fraction receiving at least 10 Gy planned dose. El Naqa et al.[27] defined both V_{10} and V_{90} as features. In the context of this work, these two features are defined as $F_{ivh.V10}$ and $F_{ivh.V90}$, respectively.

data	value	tol.	consensus
dig. phantom	0.324	—	very strong
config. A	0.978	0.001	strong
config. B	0.977	0.001	strong
config. C	0.998	0.001	moderate
config. D	0.972	0.003	strong
config. E	0.975	0.002	strong

Table 3.71 | Reference values for the *volume fraction at 10% intensity* feature.

data	value	tol.	consensus
dig. phantom	0.0946	—	very strong
config. A	6.98×10^{-5}	1.03×10^{-5}	strong
config. B	7.31×10^{-5}	1.03×10^{-5}	strong
config. C	0.000152	2×10^{-5}	strong
config. D	9×10^{-5}	0.000415	strong
config. E	0.000157	0.000248	strong

Table 3.72 | Reference values for the *volume fraction at 90% intensity* feature.

3.5.2 Intensity at volume fraction GBPN

The *intensity at volume fraction* I_x is the minimum discretised intensity i present in at most $x\%$ of the volume. El Naqa et al.[27] defined both I_{10} and I_{90} as features. In the context of this work, these two features are defined as $F_{ivh.I10}$ and $F_{ivh.I90}$, respectively.

data	value	tol.	consensus
dig. phantom	5	—	very strong
config. A	96	—	strong
config. B	92	—	strong
config. C	88.8	0.2	moderate
config. D	87	0.1	strong
config. E	770	5	moderate

Table 3.73 | Reference values for the *intensity at 10% volume* feature.

data	value	tol.	consensus
dig. phantom	2	—	very strong
config. A	−128	8	strong
config. B	−135	8	strong
config. C	−421	14	strong
config. D	−303	20	strong
config. E	399	17	moderate

Table 3.74 | Reference values for the *intensity at 90% volume* feature.

3.5.3 Volume fraction difference between intensity fractions DDTU

This feature is the difference between the volume fractions at two different intensity fractions, e.g. $V_{10} - V_{90}$ [27]. In the context of this work, this feature is defined as $F_{ivh.V10minusV90}$.

data	value	tol.	consensus
dig. phantom	0.23	—	very strong
config. A	0.978	0.001	strong
config. B	0.977	0.001	strong
config. C	0.997	0.001	strong
config. D	0.971	0.001	strong
config. E	0.974	0.001	strong

Table 3.75 | Reference values for the *volume fraction difference between 10% and 90% intensity* feature.

3.5.4 Intensity fraction difference between volume fractions CNV2

This feature is the difference between discretised intensities at two different fractional volumes, e.g. $I_{10} - I_{90}$ [27]. In the context of this work, this feature is defined as $F_{ivh.I10minusI90}$.

data	value	tol.	consensus
dig. phantom	3	—	very strong
config. A	224	8	strong
config. B	227	8	strong
config. C	510	14	strong
config. D	390	20	strong
config. E	371	13	moderate

Table 3.76 | Reference values for the *intensity difference between 10% and 90% volume* feature.

3.5.5 Area under the IVH curve 9CMM

Note: This feature currently has no reference values and should not be used.

The *area under the IVH curve* $F_{ivh.auc}$ was defined by van Velden et al. [82]. The *area under the IVH curve* can be approximated by calculating the Riemann sum using the trapezoidal rule. Note that if there is only one discretised intensity in the ROI, we define the *area under the IVH curve* $F_{ivh.auc} = 0$.

3.6 Grey level co-occurrence based features LFYI

In image analysis, texture is one of the defining sets of features. Texture features were originally designed to assess surface texture in 2D images. Texture analysis is however not restricted to 2D slices and can be extended to 3D objects. Image intensities are generally discretised before calculation of texture features, see Section 2.7.

The grey level co-occurrence matrix (GLCM) is a matrix that expresses how combinations of discretised intensities (grey levels) of neighbouring pixels, or voxels in a 3D volume, are distributed along one of the image directions. Generally, the neighbourhood for GLCM is a 26-connected neighbourhood in 3D and a 8-connected neighbourhood in 2D. Thus, in 3D there are 13 unique direction vectors within the neighbourhood for Chebyshev distance $\delta = 1$, i.e. $(0,0,1)$, $(0,1,0)$, $(1,0,0)$, $(0,1,1)$, $(0,1,-1)$, $(1,0,1)$, $(1,0,-1)$, $(1,1,0)$, $(1,-1,0)$, $(1,1,1)$, $(1,1,-1)$, $(1,-1,1)$ and $(1,-1,-1)$, whereas in 2D the direction vectors are $(1,0,0)$, $(1,1,0)$, $(0,1,0)$ and $(-1,1,0)$.

A GLCM is calculated for each direction vector, as follows. Let $\mathbf{M_m}$ be the $N_g \times N_g$ grey level co-occurrence matrix, with N_g the number of discretised grey levels present in the ROI intensity mask, and $\mathbf{m}$ the particular direction vector. Element (i,j) of the GLCM contains the frequency at which combinations of discretised grey levels i and j occur in neighbouring voxels along direction $\mathbf{m}_+ = \mathbf{m}$ and along direction $\mathbf{m}_- = -\mathbf{m}$. Then, $\mathbf{M_m} = \mathbf{M_{m_+}} + \mathbf{M_{m_-}} = \mathbf{M_{m_+}} + \mathbf{M}^T_{\mathbf{m}_+}$ [36]. As a consequence the GLCM matrix $\mathbf{M_m}$ is symmetric. An example of the calculation of a GLCM is shown in Table 3.77. Corresponding grey level co-occurrence matrices for each direction are shown in Table 3.78.

(a) Grey levels

1	2	2	3
1	2	3	3
4	2	4	1
4	1	2	3

(b) $\mathbf{M}_{\mathbf{m}_+=\rightarrow}$

	j			
i	0	3	0	0
	0	1	3	1
	0	0	1	0
	2	1	0	0

(c) $\mathbf{M}_{\mathbf{m}_-=\leftarrow}$

	j			
i	0	0	0	2
	3	1	0	1
	0	3	1	0
	0	1	0	0

Table 3.77 | Grey levels (a) and corresponding grey level co-occurrence matrices for the 0° (b) and 180° directions (c). In vector notation these directions are $\mathbf{m}_+ = (1,0)$ and $\mathbf{m}_- = (-1,0)$. To calculate the symmetrical co-occurrence matrix $\mathbf{M_m}$ both matrices are summed by element.

GLCM features rely on the probability distribution for the elements of the GLCM. Let us consider $\mathbf{M}_{\mathbf{m}=(1,0)}$ from the example, as shown in Table 3.79. We derive a probability distribution for grey level co-occurrences, $\mathbf{P_m}$, by normalising $\mathbf{M_m}$ by the sum of its elements. Each element p_{ij} of $\mathbf{P_m}$ is then the joint probability of grey levels i and j occurring in neighbouring voxels along direction $\mathbf{m}$. Then $p_{i.} = \sum_{j=1}^{N_g} p_{ij}$ is the row marginal probability, and $p_{.j} = \sum_{i=1}^{N_g} p_{ij}$ is the column marginal probability. As $\mathbf{P_m}$ is by definition symmetric, $p_{i.} = p_{.j}$. Furthermore, let us consider diagonal and cross-diagonal probabilities p_{i-j} and p_{i+j} [36,74]:

$$p_{i-j,k} = \sum_{i=1}^{N_g}\sum_{j=1}^{N_g} p_{ij}\ [k = |i-j|] \qquad k = 0,\dots,N_g-1$$

$$p_{i+j,k} = \sum_{i=1}^{N_g}\sum_{j=1}^{N_g} p_{ij}\ [k = i+j] \qquad k = 2,\dots,2N_g$$

Here, $[\dots]$ is an Iverson bracket, which equals 1 when the condition within the brackets is

(a) $\mathbf{M}_{\mathbf{m}=\rightarrow}$

	j			
i	0	3	0	2
	3	2	3	2
	0	3	2	0
	2	2	0	0

(b) $\mathbf{M}_{\mathbf{m}=\nearrow}$

	j			
i	0	2	0	1
	2	2	1	2
	0	1	2	1
	1	2	1	0

(c) $\mathbf{M}_{\mathbf{m}=\uparrow}$

	j			
i	2	1	2	1
	1	4	1	1
	2	1	2	1
	1	1	1	2

(d) $\mathbf{M}_{\mathbf{m}=\nwarrow}$

	j			
i	0	2	1	1
	2	2	2	1
	1	2	0	1
	1	1	1	0

Table 3.78 | Grey level co-occurrence matrices for the 0° (a), 45° (b), 90° (c) and 135° (d) directions. In vector notation these directions are $\mathbf{m} = (1,0)$, $\mathbf{m} = (1,1)$, $\mathbf{m} = (0,1)$ and $\mathbf{m} = (-1,1)$, respectively.

true and 0 otherwise. In effect we select only combinations of elements (i,j) for which the condition holds.

It should be noted that while a distance $\delta = 1$ is commonly used for GLCM, other distances are possible. However, this does not change the number of For example, for $\delta = 3$ (in 3D) the voxels at $(0,0,3)$, $(0,3,0)$, $(3,0,0)$, $(0,3,3)$, $(0,3,-3)$, $(3,0,3)$, $(3,0,-3)$, $(3,3,0)$, $(3,-3,0)$, $(3,3,3)$, $(3,3,-3)$, $(3,-3,3)$ and $(3,-3,-3)$ from the center voxel are considered.

Aggregating features

To improve rotational invariance, GLCM feature values are computed by aggregating information from the different underlying directional matrices[23]. Five methods can be used to aggregate GLCMs and arrive at a single feature value. A schematic example is shown in Figure 3.3. A feature may be aggregated as follows:

1. Features are computed from each 2D directional matrix and averaged over 2D directions and slices (`BTW3`).
2. Features are computed from a single matrix after merging 2D directional matrices per slice, and then averaged over slices (`SUJT`).
3. Features are computed from a single matrix after merging 2D directional matrices per direction, and then averaged over directions (`JJUI`).
4. The feature is computed from a single matrix after merging all 2D directional matrices (`ZW7Z`).
5. Features are computed from each 3D directional matrix and averaged over the 3D directions (`ITBB`).
6. The feature is computed from a single matrix after merging all 3D directional matrices (`IAZD`).

In methods 2,3,4 and 6, matrices are merged by summing the co-occurrence counts in each matrix element (i,j) over the different matrices. Probability distributions are subsequently calculated for the merged GLCM, which is then used to calculate GLCM features. Feature values may dependent strongly on the aggregation method.

		j			$\sum_j$
i	0	3	0	2	**5**
	3	2	3	2	**10**
	0	3	2	0	**5**
	2	2	0	0	**4**
$\sum_i$	**5**	**10**	**5**	**4**	**24**

(a) $\mathbf{M}_{\mathbf{m}=(1,0)}$ with margins

		j			$p_{i.}$
i	0.00	0.13	0.00	0.08	**0.21**
	0.13	0.08	0.13	0.08	**0.42**
	0.00	0.13	0.08	0.00	**0.21**
	0.08	0.08	0.00	0.00	**0.17**
$p_{.j}$	**0.21**	**0.42**	**0.21**	**0.17**	**1.00**

(b) $\mathbf{P}_{\mathbf{m}=(1,0)}$ with margins

$k=\lvert i-j\rvert$	0	1	2	3
p_{i-j}	0.17	0.50	0.17	0.17

(c) Diagonal probability for $\mathbf{P}_{\mathbf{m}=(1,0)}$

$k=i+j$	2	3	4	5	6	7	8
p_{i+j}	0.00	0.25	0.08	0.42	0.25	0.00	0.00

(d) Cross-diagonal probability for $\mathbf{P}_{\mathbf{m}=(1,0)}$

Table 3.79 | Grey level co-occurrence matrix for the 0° direction (a); its corresponding probability matrix $\mathbf{P}_{\mathbf{m}=(1,0)}$ with marginal probabilities $p_{i.}$ and $p_{.j}$(b); the diagonal probabilities p_{i-j} (c); and the cross-diagonal probabilities p_{i+j} (d). Discrepancies in panels b, c, and d are due to rounding errors caused by showing only two decimal places. Also, note that due to GLCM symmetry marginal probabilities $p_{i.}$ and $p_{.j}$ are the same in both row and column margins of panel b.

Distances and distance weighting

The default neighbourhood includes all voxels within Chebyshev distance 1. The corresponding direction vectors are multiplied by the desired distance δ. From a technical point-of-view, direction vectors may also be determined differently, using any distance norm. In this case, direction vectors are the vectors to the voxels at δ, or between δ and $\delta - 1$ for the Euclidean norm. Such usage is however rare and we caution against it due to potential reproducibility issues.

GLCMs may be weighted for distance by multiplying M with a weighting factor w. By default $w = 1$, but w may also be an inverse distance function to weight each GLCM, e.g. $w = \|\mathbf{m}\|^{-1}$ or $w = \exp(-\|\mathbf{m}\|^2)$ [81], with $\|\mathbf{m}\|$ the length of direction vector m. Whether distance weighting yields different feature values depends on several factors. When aggregating the feature values, matrices have to be merged first, otherwise weighting has no effect. Also, it has no effect if the default neighbourhood is used and the Chebyshev norm is using for weighting. Nor does weighting have an effect if either Manhattan or Chebyshev norms are used both for constructing a non-default neighbourhood and for weighting. Weighting may furthermore have no effect for distance $\delta = 1$, dependent on distance norms. Because of these exceptions, we recommend against using distance weighting for GLCM.

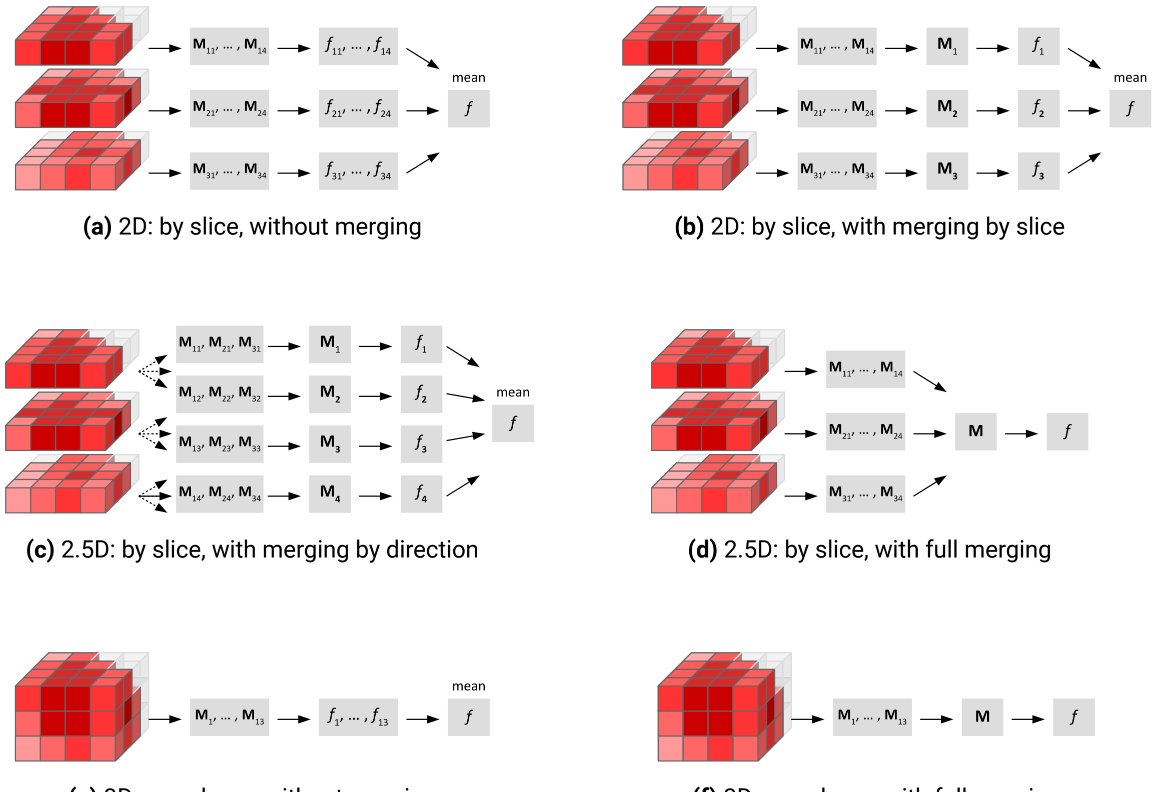

(a) 2D: by slice, without merging

(b) 2D: by slice, with merging by slice

(c) 2.5D: by slice, with merging by direction

(d) 2.5D: by slice, with full merging

(e) 3D: as volume, without merging

(f) 3D: as volume, with full merging

Figure 3.3 | Approaches to calculating grey level co-occurrence matrix-based features. $\mathrm{M}_{\Delta k}$ are texture matrices calculated for direction Δ in slice k (if applicable), and $f_{\Delta k}$ is the corresponding feature value. In (b-d) and (e) the matrices are merged prior to feature calculation.

3.6.1 Joint maximum GYBY

Joint maximum[35] is the probability corresponding to the most common grey level co-occurrence in the GLCM:

$$F_{cm.joint.max} = \max(p_{ij})$$

data	aggr. method	value	tol.	consensus
dig. phantom	2D, averaged	0.519	–	very strong
dig. phantom	2D, slice-merged	0.512	–	strong
dig. phantom	2.5D, direction-merged	0.489	–	strong
dig. phantom	2.5D, merged	0.492	–	strong
dig. phantom	3D, averaged	0.503	–	very strong
dig. phantom	3D, merged	0.509	–	very strong
config. A	2D, averaged	0.109	0.001	strong
config. A	2D, slice-merged	0.109	0.001	strong
config. A	2.5D, direction-merged	0.0943	0.0008	strong
config. A	2.5D, merged	0.0943	0.0008	strong
config. B	2D, averaged	0.156	0.002	strong
config. B	2D, slice-merged	0.156	0.002	strong
config. B	2.5D, direction-merged	0.126	0.002	strong

config. B	2.5D, merged	0.126	0.002	strong
config. C	3D, averaged	0.111	0.002	strong
config. C	3D, merged	0.111	0.002	very strong
config. D	3D, averaged	0.232	0.007	strong
config. D	3D, merged	0.232	0.007	strong
config. E	3D, averaged	0.153	0.003	moderate
config. E	3D, merged	0.153	0.003	strong

Table 3.80 | Reference values for the *joint maximum* feature.

3.6.2 Joint average 60VM

Joint average[74] is the grey level weighted sum of joint probabilities:

$$F_{cm.joint.avg} = \sum_{i=1}^{N_g} \sum_{j=1}^{N_g} i p_{ij}$$

data	aggr. method	value	tol.	consensus
dig. phantom	2D, averaged	2.14	—	very strong
dig. phantom	2D, slice-merged	2.14	—	strong
dig. phantom	2.5D, direction-merged	2.2	—	strong
dig. phantom	2.5D, merged	2.2	—	strong
dig. phantom	3D, averaged	2.14	—	very strong
dig. phantom	3D, merged	2.15	—	very strong
config. A	2D, averaged	20.6	0.1	strong
config. A	2D, slice-merged	20.6	0.1	strong
config. A	2.5D, direction-merged	21.3	0.1	strong
config. A	2.5D, merged	21.3	0.1	strong
config. B	2D, averaged	18.7	0.3	strong
config. B	2D, slice-merged	18.7	0.3	strong
config. B	2.5D, direction-merged	19.2	0.3	strong
config. B	2.5D, merged	19.2	0.3	strong
config. C	3D, averaged	39	0.2	strong
config. C	3D, merged	39	0.2	strong
config. D	3D, averaged	18.9	0.5	strong
config. D	3D, merged	18.9	0.5	strong
config. E	3D, averaged	22.1	0.3	strong
config. E	3D, merged	22.1	0.3	strong

Table 3.81 | Reference values for the *joint average* feature.

3.6.3 Joint variance

UR99

The *joint variance*[74], which is also called *sum of squares*[36], is defined as:

$$F_{cm.joint.var} = \sum_{i=1}^{N_g} \sum_{j=1}^{N_g} (i - \mu)^2 p_{ij}$$

Here μ is equal to the value of $F_{cm.joint.avg}$, which was defined above.

data	aggr. method	value	tol.	consensus
dig. phantom	2D, averaged	2.69	–	very strong
dig. phantom	2D, slice-merged	2.71	–	strong
dig. phantom	2.5D, direction-merged	3.22	–	strong
dig. phantom	2.5D, merged	3.24	–	strong
dig. phantom	3D, averaged	3.1	–	very strong
dig. phantom	3D, merged	3.13	–	very strong
config. A	2D, averaged	27	0.4	strong
config. A	2D, slice-merged	27	0.4	strong
config. A	2.5D, direction-merged	18.6	0.5	strong
config. A	2.5D, merged	18.6	0.5	strong
config. B	2D, averaged	21	0.3	strong
config. B	2D, slice-merged	21	0.3	strong
config. B	2.5D, direction-merged	14.2	0.1	strong
config. B	2.5D, merged	14.2	0.1	strong
config. C	3D, averaged	73.7	2	strong
config. C	3D, merged	73.8	2	very strong
config. D	3D, averaged	17.6	0.4	strong
config. D	3D, merged	17.6	0.4	strong
config. E	3D, averaged	24.4	0.9	moderate
config. E	3D, merged	24.4	0.9	strong

Table 3.82 | Reference values for the *joint variance* feature.

3.6.4 Joint entropy

TU9B

Joint entropy[36] is defined as:

$$F_{cm.joint.entr} = -\sum_{i=1}^{N_g} \sum_{j=1}^{N_g} p_{ij} \log_2 p_{ij}$$

data	aggr. method	value	tol.	consensus
dig. phantom	2D, averaged	2.05	–	very strong
dig. phantom	2D, slice-merged	2.24	–	strong
dig. phantom	2.5D, direction-merged	2.48	–	strong
dig. phantom	2.5D, merged	2.61	–	strong

dig. phantom	3D, averaged	2.4	—	very strong
dig. phantom	3D, merged	2.57	—	very strong
config. A	2D, averaged	5.82	0.04	strong
config. A	2D, slice-merged	5.9	0.04	strong
config. A	2.5D, direction-merged	5.78	0.04	strong
config. A	2.5D, merged	5.79	0.04	strong
config. B	2D, averaged	5.26	0.02	strong
config. B	2D, slice-merged	5.45	0.01	strong
config. B	2.5D, direction-merged	5.45	0.01	strong
config. B	2.5D, merged	5.46	0.01	strong
config. C	3D, averaged	6.39	0.06	strong
config. C	3D, merged	6.42	0.06	very strong
config. D	3D, averaged	4.95	0.03	strong
config. D	3D, merged	4.96	0.03	strong
config. E	3D, averaged	5.6	0.03	strong
config. E	3D, merged	5.61	0.03	strong

Table 3.83 | Reference values for the *joint entropy* feature.

3.6.5 Difference average TF7R

The *difference average*[74] for the diagonal probabilities is defined as:

$$F_{cm.diff.avg} = \sum_{k=0}^{N_g-1} k p_{i-j,k}$$

By definition *difference average* is equivalent to the *dissimilarity* feature[81].

data	aggr. method	value	tol.	consensus
dig. phantom	2D, averaged	1.42	—	very strong
dig. phantom	2D, slice-merged	1.4	—	strong
dig. phantom	2.5D, direction-merged	1.46	—	strong
dig. phantom	2.5D, merged	1.44	—	strong
dig. phantom	3D, averaged	1.43	—	very strong
dig. phantom	3D, merged	1.38	—	very strong
config. A	2D, averaged	1.58	0.03	strong
config. A	2D, slice-merged	1.57	0.03	strong
config. A	2.5D, direction-merged	1.35	0.03	strong
config. A	2.5D, merged	1.35	0.03	strong
config. B	2D, averaged	1.81	0.01	strong
config. B	2D, slice-merged	1.81	0.01	strong
config. B	2.5D, direction-merged	1.47	0.01	strong
config. B	2.5D, merged	1.47	0.01	strong
config. C	3D, averaged	2.17	0.05	strong
config. C	3D, merged	2.16	0.05	strong
config. D	3D, averaged	1.29	0.01	strong

config. D	3D, merged	1.29	0.01	strong
config. E	3D, averaged	1.7	0.01	strong
config. E	3D, merged	1.7	0.01	strong

Table 3.84 | Reference values for the *difference average* feature.

3.6.6 Difference variance D3YU

The *difference variance* for the diagonal probabilities[36] is defined as:

$$F_{cm.diff.var} = \sum_{k=0}^{N_g-1} (k-\mu)^2 p_{i-j,k}$$

Here μ is equal to the value of *difference average*.

data	aggr. method	value	tol.	consensus
dig. phantom	2D, averaged	2.9	–	very strong
dig. phantom	2D, slice-merged	3.06	–	strong
dig. phantom	2.5D, direction-merged	3.11	–	strong
dig. phantom	2.5D, merged	3.23	–	strong
dig. phantom	3D, averaged	3.06	–	very strong
dig. phantom	3D, merged	3.21	–	very strong
config. A	2D, averaged	4.94	0.19	strong
config. A	2D, slice-merged	4.96	0.19	strong
config. A	2.5D, direction-merged	4.12	0.2	strong
config. A	2.5D, merged	4.14	0.2	strong
config. B	2D, averaged	7.74	0.05	strong
config. B	2D, slice-merged	7.76	0.05	strong
config. B	2.5D, direction-merged	6.48	0.06	strong
config. B	2.5D, merged	6.48	0.06	strong
config. C	3D, averaged	14.4	0.5	strong
config. C	3D, merged	14.4	0.5	strong
config. D	3D, averaged	5.37	0.11	strong
config. D	3D, merged	5.38	0.11	strong
config. E	3D, averaged	8.22	0.06	strong
config. E	3D, merged	8.23	0.06	strong

Table 3.85 | Reference values for the *difference variance* feature.

3.6.7 Difference entropy NTRS

The *difference entropy* for the diagonal probabilities[36] is defined as:

$$F_{cm.diff.entr} = -\sum_{k=0}^{N_g-1} p_{i-j,k} \log_2 p_{i-j,k}$$

data	aggr. method	value	tol.	consensus
dig. phantom	2D, averaged	1.4	—	very strong
dig. phantom	2D, slice-merged	1.49	—	strong
dig. phantom	2.5D, direction-merged	1.61	—	strong
dig. phantom	2.5D, merged	1.67	—	strong
dig. phantom	3D, averaged	1.56	—	very strong
dig. phantom	3D, merged	1.64	—	very strong
config. A	2D, averaged	2.27	0.03	strong
config. A	2D, slice-merged	2.28	0.03	strong
config. A	2.5D, direction-merged	2.16	0.03	strong
config. A	2.5D, merged	2.16	0.03	strong
config. B	2D, averaged	2.35	0.01	strong
config. B	2D, slice-merged	2.38	0.01	strong
config. B	2.5D, direction-merged	2.24	0.01	moderate
config. B	2.5D, merged	2.24	0.01	strong
config. C	3D, averaged	2.64	0.03	strong
config. C	3D, merged	2.64	0.03	very strong
config. D	3D, averaged	2.13	0.01	strong
config. D	3D, merged	2.14	0.01	strong
config. E	3D, averaged	2.39	0.01	strong
config. E	3D, merged	2.4	0.01	strong

Table 3.86 | Reference values for the *difference entropy* feature.

3.6.8 Sum average ZGXS

The *sum average* for the cross-diagonal probabilities[36] is defined as:

$$F_{cm.sum.avg} = \sum_{k=2}^{2N_g} k\, p_{i+j,k}$$

By definition, $F_{cm.sum.avg} = 2F_{cm.joint.avg}$[81].

data	aggr. method	value	tol.	consensus
dig. phantom	2D, averaged	4.28	—	very strong
dig. phantom	2D, slice-merged	4.29	—	strong
dig. phantom	2.5D, direction-merged	4.41	—	strong
dig. phantom	2.5D, merged	4.41	—	strong
dig. phantom	3D, averaged	4.29	—	very strong
dig. phantom	3D, merged	4.3	—	very strong
config. A	2D, averaged	41.3	0.1	strong
config. A	2D, slice-merged	41.3	0.1	strong
config. A	2.5D, direction-merged	42.7	0.1	strong
config. A	2.5D, merged	42.7	0.1	strong
config. B	2D, averaged	37.4	0.5	strong

config. B	2D, slice-merged	37.4	0.5	strong
config. B	2.5D, direction-merged	38.5	0.6	strong
config. B	2.5D, merged	38.5	0.6	strong
config. C	3D, averaged	78	0.3	strong
config. C	3D, merged	78	0.3	strong
config. D	3D, averaged	37.7	0.8	strong
config. D	3D, merged	37.7	0.8	strong
config. E	3D, averaged	44.3	0.4	strong
config. E	3D, merged	44.3	0.4	strong

Table 3.87 | Reference values for the *sum average* feature.

3.6.9 Sum variance OEEB

The *sum variance* for the cross-diagonal probabilities[36] is defined as:

$$F_{cm.sum.var} = \sum_{k=2}^{2N_g} (k-\mu)^2 p_{i+j,k}$$

Here μ is equal to the value of *sum average*. *Sum variance* is mathematically identical to the *cluster tendency* feature[81].

data	aggr. method	value	tol.	consensus
dig. phantom	2D, averaged	5.47	–	very strong
dig. phantom	2D, slice-merged	5.66	–	strong
dig. phantom	2.5D, direction-merged	7.48	–	strong
dig. phantom	2.5D, merged	7.65	–	strong
dig. phantom	3D, averaged	7.07	–	very strong
dig. phantom	3D, merged	7.41	–	very strong
config. A	2D, averaged	100	1	strong
config. A	2D, slice-merged	100	1	strong
config. A	2.5D, direction-merged	68.5	1.3	strong
config. A	2.5D, merged	68.5	1.3	strong
config. B	2D, averaged	72.1	1	strong
config. B	2D, slice-merged	72.3	1	strong
config. B	2.5D, direction-merged	48.1	0.4	strong
config. B	2.5D, merged	48.1	0.4	strong
config. C	3D, averaged	276	8	strong
config. C	3D, merged	276	8	very strong
config. D	3D, averaged	63.4	1.3	strong
config. D	3D, merged	63.5	1.3	strong
config. E	3D, averaged	86.6	3.3	moderate
config. E	3D, merged	86.7	3.3	strong

Table 3.88 | Reference values for the *sum variance* feature.

3.6.10 Sum entropy P6QZ

The *sum entropy* for the cross-diagonal probabilities[36] is defined as:

$$F_{cm.sum.entr} = -\sum_{k=2}^{2N_g} p_{i+j,k} \log_2 p_{i+j,k}$$

data	aggr. method	value	tol.	consensus
dig. phantom	2D, averaged	1.6	–	very strong
dig. phantom	2D, slice-merged	1.79	–	strong
dig. phantom	2.5D, direction-merged	2.01	–	strong
dig. phantom	2.5D, merged	2.14	–	strong
dig. phantom	3D, averaged	1.92	–	very strong
dig. phantom	3D, merged	2.11	–	very strong
config. A	2D, averaged	4.19	0.03	strong
config. A	2D, slice-merged	4.21	0.03	strong
config. A	2.5D, direction-merged	4.17	0.03	strong
config. A	2.5D, merged	4.18	0.03	strong
config. B	2D, averaged	3.83	0.01	strong
config. B	2D, slice-merged	3.89	0.01	strong
config. B	2.5D, direction-merged	3.91	0.01	strong
config. B	2.5D, merged	3.91	0.01	strong
config. C	3D, averaged	4.56	0.04	strong
config. C	3D, merged	4.56	0.04	very strong
config. D	3D, averaged	3.68	0.02	strong
config. D	3D, merged	3.68	0.02	strong
config. E	3D, averaged	3.96	0.02	strong
config. E	3D, merged	3.97	0.02	strong

Table 3.89 | Reference values for the *sum entropy* feature.

3.6.11 Angular second moment 8ZQL

The *angular second moment*[36], which represents the energy of $\mathbf{P}_\Delta$, is defined as:

$$F_{cm.energy} = \sum_{i=1}^{N_g} \sum_{j=1}^{N_g} p_{ij}^2$$

This feature is also called *energy*[1,74] and *uniformity*[18].

data	aggr. method	value	tol.	consensus
dig. phantom	2D, averaged	0.368	–	very strong
dig. phantom	2D, slice-merged	0.352	–	strong
dig. phantom	2.5D, direction-merged	0.286	–	strong
dig. phantom	2.5D, merged	0.277	–	strong

dig. phantom	3D, averaged	0.303	—	very strong
dig. phantom	3D, merged	0.291	—	very strong
config. A	2D, averaged	0.045	0.0008	strong
config. A	2D, slice-merged	0.0446	0.0008	strong
config. A	2.5D, direction-merged	0.0429	0.0007	strong
config. A	2.5D, merged	0.0427	0.0007	strong
config. B	2D, averaged	0.0678	0.0006	strong
config. B	2D, slice-merged	0.0669	0.0006	strong
config. B	2.5D, direction-merged	0.0581	0.0006	strong
config. B	2.5D, merged	0.058	0.0006	strong
config. C	3D, averaged	0.045	0.001	strong
config. C	3D, merged	0.0447	0.001	very strong
config. D	3D, averaged	0.11	0.003	strong
config. D	3D, merged	0.109	0.003	strong
config. E	3D, averaged	0.0638	0.0009	strong
config. E	3D, merged	0.0635	0.0009	strong

Table 3.90 | Reference values for the *angular second moment* feature.

3.6.12 Contrast ACUI

Contrast assesses grey level variations[36]. Hence elements of $\mathbf{M}_\Delta$ that represent large grey level differences receive greater weight. *Contrast* is defined as[18]:

$$F_{cm.contrast} = \sum_{i=1}^{N_g} \sum_{j=1}^{N_g} (i-j)^2 p_{ij}$$

Note that the original definition by Haralick et al.[36] is seemingly more complex, but rearranging and simplifying terms leads to the above formulation of *contrast*.

data	aggr. method	value	tol.	consensus
dig. phantom	2D, averaged	5.28	—	very strong
dig. phantom	2D, slice-merged	5.19	—	strong
dig. phantom	2.5D, direction-merged	5.39	—	strong
dig. phantom	2.5D, merged	5.29	—	strong
dig. phantom	3D, averaged	5.32	—	very strong
dig. phantom	3D, merged	5.12	—	very strong
config. A	2D, averaged	7.85	0.26	strong
config. A	2D, slice-merged	7.82	0.26	strong
config. A	2.5D, direction-merged	5.96	0.27	strong
config. A	2.5D, merged	5.95	0.27	strong
config. B	2D, averaged	11.9	0.1	strong
config. B	2D, slice-merged	11.8	0.1	strong
config. B	2.5D, direction-merged	8.66	0.09	strong
config. B	2.5D, merged	8.65	0.09	strong
config. C	3D, averaged	19.2	0.7	strong

config. C	3D, merged	19.1	0.7	very strong
config. D	3D, averaged	7.07	0.13	strong
config. D	3D, merged	7.05	0.13	strong
config. E	3D, averaged	11.1	0.1	strong
config. E	3D, merged	11.1	0.1	strong

Table 3.91 | Reference values for the *contrast* feature.

3.6.13 Dissimilarity 8S9J

Dissimilarity[18] is conceptually similar to the *contrast* feature, and is defined as:

$$F_{cm.dissimilarity} = \sum_{i=1}^{N_g} \sum_{j=1}^{N_g} |i-j| p_{ij}$$

By definition *dissimilarity* is equivalent to the *difference average* feature[81].

data	aggr. method	value	tol.	consensus
dig. phantom	2D, averaged	1.42	—	very strong
dig. phantom	2D, slice-merged	1.4	—	strong
dig. phantom	2.5D, direction-merged	1.46	—	strong
dig. phantom	2.5D, merged	1.44	—	strong
dig. phantom	3D, averaged	1.43	—	very strong
dig. phantom	3D, merged	1.38	—	very strong
config. A	2D, averaged	1.58	0.03	strong
config. A	2D, slice-merged	1.57	0.03	strong
config. A	2.5D, direction-merged	1.35	0.03	strong
config. A	2.5D, merged	1.35	0.03	strong
config. B	2D, averaged	1.81	0.01	strong
config. B	2D, slice-merged	1.81	0.01	strong
config. B	2.5D, direction-merged	1.47	0.01	strong
config. B	2.5D, merged	1.47	0.01	strong
config. C	3D, averaged	2.17	0.05	strong
config. C	3D, merged	2.16	0.05	very strong
config. D	3D, averaged	1.29	0.01	strong
config. D	3D, merged	1.29	0.01	strong
config. E	3D, averaged	1.7	0.01	strong
config. E	3D, merged	1.7	0.01	strong

Table 3.92 | Reference values for the *dissimilarity* feature.

3.6.14 Inverse difference IB1Z

Inverse difference is a measure of homogeneity[18]. Grey level co-occurrences with a large difference in levels are weighed less, thus lowering the total feature value. The feature score

is maximal if all grey levels are the same. Inverse difference is defined as:

$$F_{cm.inv.diff} = \sum_{i=1}^{N_g} \sum_{j=1}^{N_g} \frac{p_{ij}}{1+|i-j|}$$

The equation above may also be expressed in terms of diagonal probabilities [81]:

$$F_{cm.inv.diff} = \sum_{k=0}^{N_g-1} \frac{p_{i-j,k}}{1+k}$$

data	aggr. method	value	tol.	consensus
dig. phantom	2D, averaged	0.678	—	very strong
dig. phantom	2D, slice-merged	0.683	—	strong
dig. phantom	2.5D, direction-merged	0.668	—	strong
dig. phantom	2.5D, merged	0.673	—	strong
dig. phantom	3D, averaged	0.677	—	very strong
dig. phantom	3D, merged	0.688	—	very strong
config. A	2D, averaged	0.581	0.003	strong
config. A	2D, slice-merged	0.581	0.003	strong
config. A	2.5D, direction-merged	0.605	0.003	strong
config. A	2.5D, merged	0.605	0.003	strong
config. B	2D, averaged	0.592	0.001	strong
config. B	2D, slice-merged	0.593	0.001	strong
config. B	2.5D, direction-merged	0.628	0.001	strong
config. B	2.5D, merged	0.628	0.001	strong
config. C	3D, averaged	0.582	0.004	strong
config. C	3D, merged	0.583	0.004	very strong
config. D	3D, averaged	0.682	0.003	strong
config. D	3D, merged	0.682	0.003	strong
config. E	3D, averaged	0.608	0.001	moderate
config. E	3D, merged	0.608	0.001	strong

Table 3.93 | Reference values for the *inverse difference* feature.

3.6.15 Normalised inverse difference NDRX

Clausi [18] suggested normalising *inverse difference* to improve classification ability. The normalised feature is then defined as:

$$F_{cm.inv.diff.norm} = \sum_{i=1}^{N_g} \sum_{j=1}^{N_g} \frac{p_{ij}}{1+|i-j|/N_g}$$

Note that in Clausi's definition, $|i-j|^2/N_g^2$ is used instead of $|i-j|/N_g$, which is likely an oversight, as this exactly matches the definition of the *normalised inverse difference moment* feature.

The equation may also be expressed in terms of diagonal probabilities[81]:

$$F_{cm.inv.diff.norm} = \sum_{k=0}^{N_g-1} \frac{p_{i-j,k}}{1+k/N_g}$$

data	aggr. method	value	tol.	consensus
dig. phantom	2D, averaged	0.851	–	very strong
dig. phantom	2D, slice-merged	0.854	–	strong
dig. phantom	2.5D, direction-merged	0.847	–	strong
dig. phantom	2.5D, merged	0.85	–	strong
dig. phantom	3D, averaged	0.851	–	very strong
dig. phantom	3D, merged	0.856	–	very strong
config. A	2D, averaged	0.961	0.001	strong
config. A	2D, slice-merged	0.961	0.001	strong
config. A	2.5D, direction-merged	0.966	0.001	strong
config. A	2.5D, merged	0.966	0.001	strong
config. B	2D, averaged	0.952	0.001	strong
config. B	2D, slice-merged	0.952	0.001	strong
config. B	2.5D, direction-merged	0.96	0.001	strong
config. B	2.5D, merged	0.96	0.001	strong
config. C	3D, averaged	0.966	0.001	strong
config. C	3D, merged	0.966	0.001	very strong
config. D	3D, averaged	0.965	0.001	strong
config. D	3D, merged	0.965	0.001	strong
config. E	3D, averaged	0.955	0.001	strong
config. E	3D, merged	0.955	0.001	strong

Table 3.94 | Reference values for the *normalised inverse difference* feature.

3.6.16 Inverse difference moment WF0Z

Inverse difference moment[36] is similar in concept to the *inverse difference* feature, but with lower weights for elements that are further from the diagonal:

$$F_{cm.inv.diff.mom} = \sum_{i=1}^{N_g} \sum_{j=1}^{N_g} \frac{p_{ij}}{1+(i-j)^2}$$

The equation above may also be expressed in terms of diagonal probabilities[81]:

$$F_{cm.inv.diff.mom} = \sum_{k=0}^{N_g-1} \frac{p_{i-j,k}}{1+k^2}$$

This feature is also called *homogeneity*[74].

data	aggr. method	value	tol.	consensus
dig. phantom	2D, averaged	0.619	—	very strong
dig. phantom	2D, slice-merged	0.625	—	strong
dig. phantom	2.5D, direction-merged	0.606	—	strong
dig. phantom	2.5D, merged	0.613	—	strong
dig. phantom	3D, averaged	0.618	—	very strong
dig. phantom	3D, merged	0.631	—	very strong
config. A	2D, averaged	0.544	0.003	strong
config. A	2D, slice-merged	0.544	0.003	strong
config. A	2.5D, direction-merged	0.573	0.003	strong
config. A	2.5D, merged	0.573	0.003	strong
config. B	2D, averaged	0.557	0.001	strong
config. B	2D, slice-merged	0.558	0.001	strong
config. B	2.5D, direction-merged	0.6	0.001	strong
config. B	2.5D, merged	0.6	0.001	strong
config. C	3D, averaged	0.547	0.004	strong
config. C	3D, merged	0.548	0.004	very strong
config. D	3D, averaged	0.656	0.003	strong
config. D	3D, merged	0.657	0.003	strong
config. E	3D, averaged	0.576	0.001	strong
config. E	3D, merged	0.577	0.001	strong

Table 3.95 | Reference values for the *inverse difference moment* feature.

3.6.17 Normalised inverse difference moment 1QCO

Clausi[18] suggested normalising *inverse difference moment* to improve classification performance. This leads to the following definition:

$$F_{cm.inv.diff.mom.norm} = \sum_{i=1}^{N_g} \sum_{j=1}^{N_g} \frac{p_{ij}}{1 + (i-j)^2 / N_g^2}$$

The equation above may also be expressed in terms of diagonal probabilities[81]:

$$F_{cm.inv.diff.mom.norm} = \sum_{k=0}^{N_g-1} \frac{p_{i-j,k}}{1 + \left(k/N_g\right)^2}$$

data	aggr. method	value	tol.	consensus
dig. phantom	2D, averaged	0.899	—	very strong
dig. phantom	2D, slice-merged	0.901	—	strong
dig. phantom	2.5D, direction-merged	0.897	—	strong
dig. phantom	2.5D, merged	0.899	—	strong
dig. phantom	3D, averaged	0.898	—	very strong
dig. phantom	3D, merged	0.902	—	very strong

config. A	2D, averaged	0.994	0.001	strong
config. A	2D, slice-merged	0.994	0.001	strong
config. A	2.5D, direction-merged	0.996	0.001	strong
config. A	2.5D, merged	0.996	0.001	strong
config. B	2D, averaged	0.99	0.001	strong
config. B	2D, slice-merged	0.99	0.001	strong
config. B	2.5D, direction-merged	0.992	0.001	strong
config. B	2.5D, merged	0.992	0.001	strong
config. C	3D, averaged	0.994	0.001	strong
config. C	3D, merged	0.994	0.001	very strong
config. D	3D, averaged	0.994	0.001	strong
config. D	3D, merged	0.994	0.001	strong
config. E	3D, averaged	0.99	0.001	strong
config. E	3D, merged	0.99	0.001	strong

Table 3.96 | Reference values for the *normalised inverse difference moment* feature.

3.6.18 Inverse variance E8JP

The *inverse variance*[1] feature is defined as:

$$F_{cm.inv.var} = 2\sum_{i=1}^{N_g}\sum_{j>i}^{N_g}\frac{p_{ij}}{(i-j)^2}$$

The equation above may also be expressed in terms of diagonal probabilities. Note that in this case, summation starts at $k = 1$ instead of $k = 0$[81]:

$$F_{cm.inv.var} = \sum_{k=1}^{N_g-1}\frac{p_{i-j,k}}{k^2}$$

data	aggr. method	value	tol.	consensus
dig. phantom	2D, averaged	0.0567	–	very strong
dig. phantom	2D, slice-merged	0.0553	–	strong
dig. phantom	2.5D, direction-merged	0.0597	–	strong
dig. phantom	2.5D, merged	0.0582	–	strong
dig. phantom	3D, averaged	0.0604	–	very strong
dig. phantom	3D, merged	0.0574	–	very strong
config. A	2D, averaged	0.441	0.001	strong
config. A	2D, slice-merged	0.441	0.001	strong
config. A	2.5D, direction-merged	0.461	0.002	strong
config. A	2.5D, merged	0.461	0.002	strong
config. B	2D, averaged	0.401	0.002	strong
config. B	2D, slice-merged	0.401	0.002	strong
config. B	2.5D, direction-merged	0.424	0.003	strong
config. B	2.5D, merged	0.424	0.003	strong
config. C	3D, averaged	0.39	0.003	strong

config. C	3D, merged	0.39	0.003	very strong
config. D	3D, averaged	0.341	0.005	strong
config. D	3D, merged	0.34	0.005	strong
config. E	3D, averaged	0.41	0.004	strong
config. E	3D, merged	0.41	0.004	strong

Table 3.97 | Reference values for the *inverse variance* feature.

3.6.19 Correlation NI2N

Correlation[36] is defined as:

$$F_{cm.corr} = \frac{1}{\sigma_{i.}\,\sigma_{.j}}\left(-\mu_{i.}\,\mu_{.j} + \sum_{i=1}^{N_g}\sum_{j=1}^{N_g} ij p_{ij}\right)$$

$\mu_{i.} = \sum_{i=1}^{N_g} i p_{i.}$ and $\sigma_{i.} = \left(\sum_{i=1}^{N_g}(i-\mu_{i.})^2 p_{i.}\right)^{1/2}$ are the mean and standard deviation of row marginal probability $p_{i.}$, respectively. Likewise, $\mu_{.j}$ and $\sigma_{.j}$ are the mean and standard deviation of the column marginal probability $p_{.j}$, respectively. The calculation of *correlation* can be simplified since $\mathbf{P}_\Delta$ is symmetrical:

$$F_{cm.corr} = \frac{1}{\sigma_{i.}^2}\left(-\mu_{i.}^2 + \sum_{i=1}^{N_g}\sum_{j=1}^{N_g} ij p_{ij}\right)$$

An equivalent formulation of *correlation* is:

$$F_{cm.corr} = \frac{1}{\sigma_{i.}\,\sigma_{.j}}\sum_{i=1}^{N_g}\sum_{j=1}^{N_g}\left(i-\mu_{i.}\right)\left(j-\mu_{.j}\right)p_{ij}$$

Again, simplifying due to matrix symmetry yields:

$$F_{cm.corr} = \frac{1}{\sigma_{i.}^2}\sum_{i=1}^{N_g}\sum_{j=1}^{N_g}\left(i-\mu_{i.}\right)\left(j-\mu_{i.}\right)p_{ij}$$

data	aggr. method	value	tol.	consensus
dig. phantom	2D, averaged	−0.0121	–	very strong
dig. phantom	2D, slice-merged	0.0173	–	strong
dig. phantom	2.5D, direction-merged	0.178	–	strong
dig. phantom	2.5D, merged	0.182	–	strong
dig. phantom	3D, averaged	0.157	–	very strong
dig. phantom	3D, merged	0.183	–	very strong
config. A	2D, averaged	0.778	0.002	strong
config. A	2D, slice-merged	0.78	0.002	strong
config. A	2.5D, direction-merged	0.839	0.003	strong
config. A	2.5D, merged	0.84	0.003	strong
config. B	2D, averaged	0.577	0.002	strong
config. B	2D, slice-merged	0.58	0.002	strong
config. B	2.5D, direction-merged	0.693	0.003	strong

config. B	2.5D, merged	0.695	0.003	strong
config. C	3D, averaged	0.869	0.001	strong
config. C	3D, merged	0.871	0.001	strong
config. D	3D, averaged	0.798	0.005	strong
config. D	3D, merged	0.8	0.005	strong
config. E	3D, averaged	0.771	0.006	moderate
config. E	3D, merged	0.773	0.006	strong

Table 3.98 | Reference values for the *correlation* feature.

3.6.20 Autocorrelation QWB0

Soh and Tsatsoulis [63] defined *autocorrelation* as:

$$F_{cm.auto.corr} = \sum_{i=1}^{N_g} \sum_{j=1}^{N_g} ij p_{ij}$$

data	aggr. method	value	tol.	consensus
dig. phantom	2D, averaged	5.09	—	very strong
dig. phantom	2D, slice-merged	5.14	—	strong
dig. phantom	2.5D, direction-merged	5.4	—	strong
dig. phantom	2.5D, merged	5.45	—	strong
dig. phantom	3D, averaged	5.06	—	very strong
dig. phantom	3D, merged	5.19	—	very strong
config. A	2D, averaged	455	2	strong
config. A	2D, slice-merged	455	2	strong
config. A	2.5D, direction-merged	471	2	strong
config. A	2.5D, merged	471	2	strong
config. B	2D, averaged	369	11	strong
config. B	2D, slice-merged	369	11	strong
config. B	2.5D, direction-merged	380	11	strong
config. B	2.5D, merged	380	11	strong
config. C	3D, averaged	1.58×10^3	10	strong
config. C	3D, merged	1.58×10^3	10	strong
config. D	3D, averaged	370	16	strong
config. D	3D, merged	370	16	strong
config. E	3D, averaged	509	8	strong
config. E	3D, merged	509	8	strong

Table 3.99 | Reference values for the *autocorrelation* feature.

3.6.21 Cluster tendency DG8W

Cluster tendency[1] is defined as:

$$F_{cm.clust.tend} = \sum_{i=1}^{N_g} \sum_{j=1}^{N_g} \left(i + j - \mu_{i.} - \mu_{.j}\right)^2 p_{ij}$$

Here $\mu_{i.} = \sum_{i=1}^{N_g} i p_{i.}$ and $\mu_{.j} = \sum_{j=1}^{N_g} j p_{.j}$. Because of the symmetric nature of $\mathbf{P}_\Delta$, the feature can also be formulated as:

$$F_{cm.clust.tend} = \sum_{i=1}^{N_g} \sum_{j=1}^{N_g} \left(i + j - 2\mu_{i.}\right)^2 p_{ij}$$

Cluster tendency is mathematically equal to the *sum variance* feature[81].

data	aggr. method	value	tol.	consensus
dig. phantom	2D, averaged	5.47	–	very strong
dig. phantom	2D, slice-merged	5.66	–	strong
dig. phantom	2.5D, direction-merged	7.48	–	strong
dig. phantom	2.5D, merged	7.65	–	strong
dig. phantom	3D, averaged	7.07	–	very strong
dig. phantom	3D, merged	7.41	–	very strong
config. A	2D, averaged	100	1	strong
config. A	2D, slice-merged	100	1	strong
config. A	2.5D, direction-merged	68.5	1.3	strong
config. A	2.5D, merged	68.5	1.3	strong
config. B	2D, averaged	72.1	1	strong
config. B	2D, slice-merged	72.3	1	strong
config. B	2.5D, direction-merged	48.1	0.4	strong
config. B	2.5D, merged	48.1	0.4	strong
config. C	3D, averaged	276	8	strong
config. C	3D, merged	276	8	very strong
config. D	3D, averaged	63.4	1.3	strong
config. D	3D, merged	63.5	1.3	strong
config. E	3D, averaged	86.6	3.3	moderate
config. E	3D, merged	86.7	3.3	strong

Table 3.100 | Reference values for the *cluster tendency* feature.

3.6.22 Cluster shade 7NFM

Cluster shade[74] is defined as:

$$F_{cm.clust.shade} = \sum_{i=1}^{N_g} \sum_{j=1}^{N_g} \left(i + j - \mu_{i.} - \mu_{.j}\right)^3 p_{ij}$$

As with *cluster tendency*, $\mu_{i.} = \sum_{i=1}^{N_g} i p_{i.}$ and $\mu_{.j} = \sum_{j=1}^{N_g} j p_{.j}$. Because of the symmetric nature of $\mathbf{P}_\Delta$, the feature can also be formulated as:

$$F_{cm.clust.shade} = \sum_{i=1}^{N_g} \sum_{j=1}^{N_g} \left(i + j - 2\mu_{i.}\right)^3 p_{ij}$$

data	aggr. method	value	tol.	consensus
dig. phantom	2D, averaged	7	–	very strong
dig. phantom	2D, slice-merged	6.98	–	strong
dig. phantom	2.5D, direction-merged	16.6	–	strong
dig. phantom	2.5D, merged	16.4	–	strong
dig. phantom	3D, averaged	16.6	–	very strong
dig. phantom	3D, merged	17.4	–	very strong
config. A	2D, averaged	-1.04×10^3	20	strong
config. A	2D, slice-merged	-1.05×10^3	20	strong
config. A	2.5D, direction-merged	-1.49×10^3	30	strong
config. A	2.5D, merged	-1.49×10^3	30	strong
config. B	2D, averaged	−668	17	strong
config. B	2D, slice-merged	−673	17	strong
config. B	2.5D, direction-merged	−905	19	strong
config. B	2.5D, merged	−906	19	strong
config. C	3D, averaged	-1.06×10^4	300	strong
config. C	3D, merged	-1.06×10^4	300	very strong
config. D	3D, averaged	-1.27×10^3	40	strong
config. D	3D, merged	-1.28×10^3	40	strong
config. E	3D, averaged	-2.07×10^3	70	moderate
config. E	3D, merged	-2.08×10^3	70	strong

Table 3.101 | Reference values for the *cluster shade* feature.

3.6.23 Cluster prominence AE86

Cluster prominence[74] is defined as:

$$F_{cm.clust.prom} = \sum_{i=1}^{N_g} \sum_{j=1}^{N_g} \left(i + j - \mu_{i.} - \mu_{.j}\right)^4 p_{ij}$$

As before, $\mu_{i.} = \sum_{i=1}^{N_g} i p_{i.}$ and $\mu_{.j} = \sum_{j=1}^{N_g} j p_{.j}$. Because of the symmetric nature of $\mathbf{P}_\Delta$, the feature can also be formulated as:

$$F_{cm.clust.prom} = \sum_{i=1}^{N_g} \sum_{j=1}^{N_g} \left(i + j - 2\mu_{i.}\right)^4 p_{ij}$$

data	aggr. method	value	tol.	consensus
dig. phantom	2D, averaged	79.1	–	very strong
dig. phantom	2D, slice-merged	80.4	–	strong
dig. phantom	2.5D, direction-merged	147	–	strong
dig. phantom	2.5D, merged	142	–	strong
dig. phantom	3D, averaged	145	–	very strong
dig. phantom	3D, merged	147	–	very strong
config. A	2D, averaged	5.27×10^4	500	strong
config. A	2D, slice-merged	5.28×10^4	500	strong
config. A	2.5D, direction-merged	4.76×10^4	700	strong
config. A	2.5D, merged	4.77×10^4	700	strong
config. B	2D, averaged	2.94×10^4	1.4×10^3	strong
config. B	2D, slice-merged	2.95×10^4	1.4×10^3	strong
config. B	2.5D, direction-merged	2.52×10^4	1×10^3	strong
config. B	2.5D, merged	2.53×10^4	1×10^3	strong
config. C	3D, averaged	5.69×10^5	1.1×10^4	strong
config. C	3D, merged	5.7×10^5	1.1×10^4	very strong
config. D	3D, averaged	3.57×10^4	1.4×10^3	strong
config. D	3D, merged	3.57×10^4	1.5×10^3	strong
config. E	3D, averaged	6.89×10^4	2.1×10^3	moderate
config. E	3D, merged	6.9×10^4	2.1×10^3	strong

Table 3.102 | Reference values for the *cluster prominence* feature.

3.6.24 Information correlation 1 R8DG

Information theoretic correlation is estimated using two different measures[36]. For symmetric $\mathbf{P}_\Delta$ the first measure is defined as:

$$F_{cm.info.corr.1} = \frac{HXY - HXY_1}{HX}$$

$HXY = -\sum_{i=1}^{N_g}\sum_{j=1}^{N_g} p_{ij}\log_2 p_{ij}$ is the entropy for the joint probability. $HX = -\sum_{i=1}^{N_g} p_{i.}\log_2 p_{i.}$ is the entropy for the row marginal probability, which due to symmetry is equal to the entropy of the column marginal probability. HXY_1 is a type of entropy that is defined as:

$$HXY_1 = -\sum_{i=1}^{N_g}\sum_{j=1}^{N_g} p_{ij}\log_2\left(p_{i.}p_{.j}\right)$$

data	aggr. method	value	tol.	consensus
dig. phantom	2D, averaged	−0.155	–	very strong
dig. phantom	2D, slice-merged	−0.0341	–	strong
dig. phantom	2.5D, direction-merged	−0.124	–	strong
dig. phantom	2.5D, merged	−0.0334	–	strong
dig. phantom	3D, averaged	−0.157	–	very strong

dig. phantom	3D, merged	−0.0288	−	very strong
config. A	2D, averaged	−0.236	0.001	strong
config. A	2D, slice-merged	−0.214	0.001	strong
config. A	2.5D, direction-merged	−0.231	0.001	strong
config. A	2.5D, merged	−0.228	0.001	strong
config. B	2D, averaged	−0.239	0.001	strong
config. B	2D, slice-merged	−0.181	0.001	strong
config. B	2.5D, direction-merged	−0.188	0.001	strong
config. B	2.5D, merged	−0.185	0.001	strong
config. C	3D, averaged	−0.236	0.001	strong
config. C	3D, merged	−0.228	0.001	strong
config. D	3D, averaged	−0.231	0.003	strong
config. D	3D, merged	−0.225	0.003	strong
config. E	3D, averaged	−0.181	0.003	moderate
config. E	3D, merged	−0.175	0.003	strong

Table 3.103 | Reference values for the *information correlation 1* feature.

3.6.25 Information correlation 2 JN9H

The *second measure of information theoretic correlation*[36] is estimated as follows for symmetric $\mathbf{P}_\Delta$:

$$F_{cm.info.corr.2} = \sqrt{1 - \exp\left(-2\left(HXY_2 - HXY\right)\right)}$$

As earlier, $HXY = -\sum_{i=1}^{N_g}\sum_{j=1}^{N_g} p_{ij}\log_2 p_{ij}$. HXY_2 is a type of entropy defined as:

$$HXY_2 = -\sum_{i=1}^{N_g}\sum_{j=1}^{N_g} p_{i.}p_{.j}\log_2\left(p_{i.}p_{.j}\right)$$

data	aggr. method	value	tol.	consensus
dig. phantom	2D, averaged	0.487	−	strong
dig. phantom	2D, slice-merged	0.263	−	strong
dig. phantom	2.5D, direction-merged	0.487	−	strong
dig. phantom	2.5D, merged	0.291	−	strong
dig. phantom	3D, averaged	0.52	−	very strong
dig. phantom	3D, merged	0.269	−	very strong
config. A	2D, averaged	0.863	0.003	strong
config. A	2D, slice-merged	0.851	0.002	strong
config. A	2.5D, direction-merged	0.879	0.001	strong
config. A	2.5D, merged	0.88	0.001	strong
config. B	2D, averaged	0.837	0.001	strong
config. B	2D, slice-merged	0.792	0.001	strong
config. B	2.5D, direction-merged	0.821	0.001	strong
config. B	2.5D, merged	0.819	0.001	strong

config. C	3D, averaged	0.9	0.001	strong
config. C	3D, merged	0.899	0.001	strong
config. D	3D, averaged	0.845	0.003	strong
config. D	3D, merged	0.846	0.003	strong
config. E	3D, averaged	0.813	0.004	moderate
config. E	3D, merged	0.813	0.004	strong

Table 3.104 | Reference values for the *information correlation 2* feature.

3.7 Grey level run length based features TP0I

The grey level run length matrix (GLRLM) was introduced by Galloway[30] to define various texture features. Like the grey level co-occurrence matrix, GLRLM also assesses the distribution of discretised grey levels in an image or in a stack of images. However, whereas GLCM assesses co-occurrence of grey levels within neighbouring pixels or voxels, GLRLM assesses run lengths. A run length is defined as the length of a consecutive sequence of pixels or voxels with the same grey level along direction $\mathbf{m}$, which was previously defined in Section 3.6. The GLRLM then contains the occurrences of runs with length j for a discretised grey level i.

A complete example for GLRLM construction from a 2D image is shown in Table 3.105. Let $\mathbf{M_m}$ be the $N_g \times N_r$ grey level run length matrix, where N_g is the number of discretised grey levels present in the ROI intensity mask and N_r the maximal possible run length along direction $\mathbf{m}$. Matrix element r_{ij} of the GLRLM is the occurrence of grev level i with run length j. Then, let N_v be the total number of voxels in the ROI intensity mask, and $N_s = \sum_{i=1}^{N_g}\sum_{j=1}^{N_r} r_{ij}$ the sum over all elements in $\mathbf{M_m}$. Marginal sums are also defined. Let $r_{i.}$ be the marginal sum of the runs over run lengths j for grey value i, that is $r_{i.} = \sum_{j=1}^{N_r} r_{ij}$. Similarly, the marginal sum of the runs over the grey values i for run length j is $r_{.j} = \sum_{i=1}^{N_g} r_{ij}$.

Aggregating features

To improve rotational invariance, GLRLM feature values are computed by aggregating information from the different underlying directional matrices[23]. Five methods can be used to aggregate GLRLMs and arrive at a single feature value. A schematic example was previously shown in Figure 3.3. A feature may be aggregated as follows:

1. Features are computed from each 2D directional matrix and averaged over 2D directions and slices (BTW3).
2. Features are computed from a single matrix after merging 2D directional matrices per slice, and then averaged over slices (SUJT).
3. Features are computed from a single matrix after merging 2D directional matrices per direction, and then averaged over directions (JJUI).
4. The feature is computed from a single matrix after merging all 2D directional matrices (ZW7Z).
5. Features are computed from each 3D directional matrix and averaged over the 3D directions (ITBB).
6. The feature is computed from a single matrix after merging all 3D directional matrices (IAZD).

In methods 2,3,4 and 6 matrices are merged by summing the run counts of each matrix element (i,j) over the different matrices. Note that when matrices are merged, N_v should likewise be summed to retain consistency. Feature values may dependent strongly on the aggregation method.

Distance weighting

GLRLMs may be weighted for distance by multiplying the run lengths with a weighting factor w. By default $w = 1$, but w may also be an inverse distance function, e.g. $w = \|\mathbf{m}\|^{-1}$ or

$w = \exp(-\|\mathbf{m}\|^2)$ [81], with $\|\mathbf{m}\|$ the length of direction vector m. Whether distance weighting yields different feature values depends on several factors. When aggregating the feature values, matrices have to be merged first, otherwise weighting has no effect. It also has no effect if the Chebyshev norm is used for weighting. Distance weighting is non-standard use, and we caution against it due to potential reproducibility issues.

1	2	2	3
1	2	3	3
4	2	4	1
4	1	2	3

(a) Grey levels

		Run length j			
		1	2	3	4
i	1	4	0	0	0
	2	3	1	0	0
	3	2	1	0	0
	4	3	0	0	0

(b) $\mathbf{M}_{\mathbf{m}=\rightarrow}$

		Run length j			
		1	2	3	4
i	1	4	0	0	0
	2	3	1	0	0
	3	2	1	0	0
	4	3	0	0	0

(c) $\mathbf{M}_{\mathbf{m}=\nearrow}$

		Run length j			
		1	2	3	4
i	1	2	1	0	0
	2	2	0	1	0
	3	2	1	0	0
	4	1	1	0	0

(d) $\mathbf{M}_{\mathbf{m}=\uparrow}$

		Run length j			
		1	2	3	4
i	1	4	0	0	0
	2	3	1	0	0
	3	4	0	0	0
	4	3	0	0	0

(e) $\mathbf{M}_{\mathbf{m}=\nwarrow}$

Table 3.105 | Grey level run length matrices for the 0° (a), 45° (b), 90° (c) and 135° (d) directions. In vector notation these directions are $\mathbf{m} = (1,0)$, $\mathbf{m} = (1,1)$, $\mathbf{m} = (0,1)$ and $\mathbf{m} = (-1,1)$, respectively.

3.7.1 Short runs emphasis 22OV

This feature emphasises short run lengths [30]. It is defined as:

$$F_{rlm.sre} = \frac{1}{N_s}\sum_{j=1}^{N_r}\frac{r_{.j}}{j^2}$$

data	aggr. method	value	tol.	consensus
dig. phantom	2D, averaged	0.641	—	very strong
dig. phantom	2D, slice-merged	0.661	—	strong
dig. phantom	2.5D, direction-merged	0.665	—	strong

dig. phantom	2.5D, merged	0.68	—	strong
dig. phantom	3D, averaged	0.705	—	very strong
dig. phantom	3D, merged	0.729	—	very strong
config. A	2D, averaged	0.785	0.003	strong
config. A	2D, slice-merged	0.786	0.003	strong
config. A	2.5D, direction-merged	0.768	0.003	strong
config. A	2.5D, merged	0.769	0.003	strong
config. B	2D, averaged	0.781	0.001	strong
config. B	2D, slice-merged	0.782	0.001	strong
config. B	2.5D, direction-merged	0.759	0.001	strong
config. B	2.5D, merged	0.759	0.001	strong
config. C	3D, averaged	0.786	0.003	strong
config. C	3D, merged	0.787	0.003	strong
config. D	3D, averaged	0.734	0.001	strong
config. D	3D, merged	0.736	0.001	strong
config. E	3D, averaged	0.776	0.001	moderate
config. E	3D, merged	0.777	0.001	strong

Table 3.106 | Reference values for the *short runs emphasis* feature.

3.7.2 Long runs emphasis W4KF

This feature emphasises long run lengths[30]. It is defined as:

$$F_{rlm.lre} = \frac{1}{N_s}\sum_{j=1}^{N_r} j^2 r_{.j}$$

data	aggr. method	value	tol.	consensus
dig. phantom	2D, averaged	3.78	—	very strong
dig. phantom	2D, slice-merged	3.51	—	strong
dig. phantom	2.5D, direction-merged	3.46	—	strong
dig. phantom	2.5D, merged	3.27	—	strong
dig. phantom	3D, averaged	3.06	—	very strong
dig. phantom	3D, merged	2.76	—	very strong
config. A	2D, averaged	2.91	0.03	strong
config. A	2D, slice-merged	2.89	0.03	strong
config. A	2.5D, direction-merged	3.09	0.03	strong
config. A	2.5D, merged	3.08	0.03	strong
config. B	2D, averaged	3.52	0.04	strong
config. B	2D, slice-merged	3.5	0.04	strong
config. B	2.5D, direction-merged	3.82	0.05	strong
config. B	2.5D, merged	3.81	0.05	strong
config. C	3D, averaged	3.31	0.04	strong
config. C	3D, merged	3.28	0.04	strong
config. D	3D, averaged	6.66	0.18	strong

config. D	3D, merged	6.56	0.18	strong
config. E	3D, averaged	3.55	0.07	strong
config. E	3D, merged	3.52	0.07	strong

Table 3.107 | Reference values for the *long runs emphasis* feature.

3.7.3 Low grey level run emphasis V3SW

This feature is a grey level analogue to *short runs emphasis*[15]. Instead of short run lengths, low grey levels are emphasised. The feature is defined as:

$$F_{rlm.lgre} = \frac{1}{N_s} \sum_{i=1}^{N_g} \frac{r_{i.}}{i^2}$$

data	aggr. method	value	tol.	consensus
dig. phantom	2D, averaged	0.604	–	very strong
dig. phantom	2D, slice-merged	0.609	–	strong
dig. phantom	2.5D, direction-merged	0.58	–	strong
dig. phantom	2.5D, merged	0.585	–	strong
dig. phantom	3D, averaged	0.603	–	very strong
dig. phantom	3D, merged	0.607	–	very strong
config. A	2D, averaged	0.0264	0.0003	strong
config. A	2D, slice-merged	0.0264	0.0003	strong
config. A	2.5D, direction-merged	0.0148	0.0004	strong
config. A	2.5D, merged	0.0147	0.0004	strong
config. B	2D, averaged	0.0331	0.0006	strong
config. B	2D, slice-merged	0.033	0.0006	strong
config. B	2.5D, direction-merged	0.0194	0.0006	strong
config. B	2.5D, merged	0.0194	0.0006	strong
config. C	3D, averaged	0.00155	5×10^{-5}	strong
config. C	3D, merged	0.00155	5×10^{-5}	strong
config. D	3D, averaged	0.0257	0.0012	strong
config. D	3D, merged	0.0257	0.0012	strong
config. E	3D, averaged	0.0204	0.0008	moderate
config. E	3D, merged	0.0204	0.0008	strong

Table 3.108 | Reference values for the *low grey level run emphasis* feature.

3.7.4 High grey level run emphasis G3QZ

The *high grey level run emphasis* feature is a grey level analogue to *long runs emphasis*[15]. The feature emphasises high grey levels, and is defined as:

$$F_{rlm.hgre} = \frac{1}{N_s} \sum_{i=1}^{N_g} i^2 r_{i.}$$

data	aggr. method	value	tol.	consensus
dig. phantom	2D, averaged	9.82	—	very strong
dig. phantom	2D, slice-merged	9.74	—	strong
dig. phantom	2.5D, direction-merged	10.3	—	strong
dig. phantom	2.5D, merged	10.2	—	strong
dig. phantom	3D, averaged	9.7	—	very strong
dig. phantom	3D, merged	9.64	—	very strong
config. A	2D, averaged	428	3	strong
config. A	2D, slice-merged	428	3	strong
config. A	2.5D, direction-merged	449	3	strong
config. A	2.5D, merged	449	3	strong
config. B	2D, averaged	342	11	strong
config. B	2D, slice-merged	342	11	strong
config. B	2.5D, direction-merged	356	11	strong
config. B	2.5D, merged	356	11	strong
config. C	3D, averaged	1.47×10^3	10	strong
config. C	3D, merged	1.47×10^3	10	strong
config. D	3D, averaged	326	17	strong
config. D	3D, merged	326	17	strong
config. E	3D, averaged	471	9	strong
config. E	3D, merged	471	9	strong

Table 3.109 | Reference values for the *high grey level run emphasis* feature.

3.7.5 Short run low grey level emphasis HTZT

This feature emphasises runs in the upper left quadrant of the GLRLM, where short run lengths and low grey levels are located[22]. It is defined as:

$$F_{rlm.srlge} = \frac{1}{N_s} \sum_{i=1}^{N_g} \sum_{j=1}^{N_r} \frac{r_{ij}}{i^2 j^2}$$

data	aggr. method	value	tol.	consensus
dig. phantom	2D, averaged	0.294	—	very strong
dig. phantom	2D, slice-merged	0.311	—	strong
dig. phantom	2.5D, direction-merged	0.296	—	strong
dig. phantom	2.5D, merged	0.312	—	strong
dig. phantom	3D, averaged	0.352	—	very strong
dig. phantom	3D, merged	0.372	—	very strong
config. A	2D, averaged	0.0243	0.0003	strong
config. A	2D, slice-merged	0.0243	0.0003	strong
config. A	2.5D, direction-merged	0.0135	0.0004	strong
config. A	2.5D, merged	0.0135	0.0004	strong
config. B	2D, averaged	0.0314	0.0006	strong

config. B	2D, slice-merged	0.0313	0.0006	strong
config. B	2.5D, direction-merged	0.0181	0.0006	strong
config. B	2.5D, merged	0.0181	0.0006	strong
config. C	3D, averaged	0.00136	5×10^{-5}	strong
config. C	3D, merged	0.00136	5×10^{-5}	strong
config. D	3D, averaged	0.0232	0.001	strong
config. D	3D, merged	0.0232	0.001	strong
config. E	3D, averaged	0.0187	0.0007	moderate
config. E	3D, merged	0.0186	0.0007	strong

Table 3.110 | Reference values for the *short run low grey level emphasis* feature.

3.7.6 Short run high grey level emphasis GD3A

This feature emphasises runs in the lower left quadrant of the GLRLM, where short run lengths and high grey levels are located[22]. The feature is defined as:

$$F_{rlm.srhge} = \frac{1}{N_s} \sum_{i=1}^{N_g} \sum_{j=1}^{N_r} \frac{i^2 r_{ij}}{j^2}$$

data	aggr. method	value	tol.	consensus
dig. phantom	2D, averaged	8.57	–	very strong
dig. phantom	2D, slice-merged	8.67	–	strong
dig. phantom	2.5D, direction-merged	9.03	–	strong
dig. phantom	2.5D, merged	9.05	–	strong
dig. phantom	3D, averaged	8.54	–	very strong
dig. phantom	3D, merged	8.67	–	very strong
config. A	2D, averaged	320	1	strong
config. A	2D, slice-merged	320	1	strong
config. A	2.5D, direction-merged	332	1	strong
config. A	2.5D, merged	333	1	strong
config. B	2D, averaged	251	8	strong
config. B	2D, slice-merged	252	8	strong
config. B	2.5D, direction-merged	257	9	strong
config. B	2.5D, merged	258	9	strong
config. C	3D, averaged	1.1×10^3	10	strong
config. C	3D, merged	1.1×10^3	10	strong
config. D	3D, averaged	219	13	strong
config. D	3D, merged	219	13	strong
config. E	3D, averaged	346	7	strong
config. E	3D, merged	347	7	strong

Table 3.111 | Reference values for the *short run high grey level emphasis* feature.

3.7.7 Long run low grey level emphasis IVPO

This feature emphasises runs in the upper right quadrant of the GLRLM, where long run lengths and low grey levels are located [22]. The feature is defined as:

$$F_{rlm.lrlge} = \frac{1}{N_s} \sum_{i=1}^{N_g} \sum_{j=1}^{N_r} \frac{j^2 r_{ij}}{i^2}$$

data	aggr. method	value	tol.	consensus
dig. phantom	2D, averaged	3.14	—	very strong
dig. phantom	2D, slice-merged	2.92	—	strong
dig. phantom	2.5D, direction-merged	2.79	—	strong
dig. phantom	2.5D, merged	2.63	—	strong
dig. phantom	3D, averaged	2.39	—	very strong
dig. phantom	3D, merged	2.16	—	very strong
config. A	2D, averaged	0.0386	0.0003	strong
config. A	2D, slice-merged	0.0385	0.0003	strong
config. A	2.5D, direction-merged	0.0229	0.0004	strong
config. A	2.5D, merged	0.0228	0.0004	strong
config. B	2D, averaged	0.0443	0.0008	strong
config. B	2D, slice-merged	0.0442	0.0008	strong
config. B	2.5D, direction-merged	0.0293	0.0009	strong
config. B	2.5D, merged	0.0292	0.0009	strong
config. C	3D, averaged	0.00317	4×10^{-5}	strong
config. C	3D, merged	0.00314	4×10^{-5}	strong
config. D	3D, averaged	0.0484	0.0031	strong
config. D	3D, merged	0.0478	0.0031	strong
config. E	3D, averaged	0.0313	0.0016	moderate
config. E	3D, merged	0.0311	0.0016	strong

Table 3.112 | Reference values for the *long run low grey level emphasis* feature.

3.7.8 Long run high grey level emphasis 3KUM

This feature emphasises runs in the lower right quadrant of the GLRLM, where long run lengths and high grey levels are located [22]. The feature is defined as:

$$F_{rlm.lrhge} = \frac{1}{N_s} \sum_{i=1}^{N_g} \sum_{j=1}^{N_r} i^2 j^2 r_{ij}$$

data	aggr. method	value	tol.	consensus
dig. phantom	2D, averaged	17.4	—	very strong
dig. phantom	2D, slice-merged	16.1	—	strong
dig. phantom	2.5D, direction-merged	17.9	—	strong

dig. phantom	2.5D, merged	17	–	strong
dig. phantom	3D, averaged	17.6	–	very strong
dig. phantom	3D, merged	15.6	–	very strong
config. A	2D, averaged	1.41×10^3	20	strong
config. A	2D, slice-merged	1.4×10^3	20	strong
config. A	2.5D, direction-merged	1.5×10^3	20	strong
config. A	2.5D, merged	1.5×10^3	20	strong
config. B	2D, averaged	1.39×10^3	30	strong
config. B	2D, slice-merged	1.38×10^3	30	strong
config. B	2.5D, direction-merged	1.5×10^3	30	strong
config. B	2.5D, merged	1.5×10^3	30	strong
config. C	3D, averaged	5.59×10^3	80	strong
config. C	3D, merged	5.53×10^3	80	strong
config. D	3D, averaged	2.67×10^3	30	strong
config. D	3D, merged	2.63×10^3	30	strong
config. E	3D, averaged	1.9×10^3	20	moderate
config. E	3D, merged	1.89×10^3	20	strong

Table 3.113 | Reference values for the *long run high grey level emphasis* feature.

3.7.9 Grey level non-uniformity R5YN

This feature assesses the distribution of runs over the grey values[30]. The feature value is low when runs are equally distributed along grey levels. The feature is defined as:

$$F_{rlm.glnu} = \frac{1}{N_s} \sum_{i=1}^{N_g} r_{i.}^2$$

data	aggr. method	value	tol.	consensus
dig. phantom	2D, averaged	5.2	–	very strong
dig. phantom	2D, slice-merged	20.5	–	strong
dig. phantom	2.5D, direction-merged	19.5	–	strong
dig. phantom	2.5D, merged	77.1	–	strong
dig. phantom	3D, averaged	21.8	–	very strong
dig. phantom	3D, merged	281	–	very strong
config. A	2D, averaged	432	1	strong
config. A	2D, slice-merged	1.73×10^3	10	strong
config. A	2.5D, direction-merged	9.85×10^3	10	strong
config. A	2.5D, merged	3.94×10^4	100	strong
config. B	2D, averaged	107	1	strong
config. B	2D, slice-merged	427	1	strong
config. B	2.5D, direction-merged	2.4×10^3	10	strong
config. B	2.5D, merged	9.6×10^3	20	strong
config. C	3D, averaged	3.18×10^3	10	strong
config. C	3D, merged	4.13×10^4	100	strong

config. D	3D, averaged	3.29×10^3	10	strong
config. D	3D, merged	4.28×10^4	200	strong
config. E	3D, averaged	4×10^3	10	moderate
config. E	3D, merged	5.19×10^4	200	strong

Table 3.114 | Reference values for the *grey level non-uniformity* feature.

3.7.10 Normalised grey level non-uniformity OVBL

This is a normalised version of the *grey level non-uniformity* feature. It is defined as:

$$F_{rlm.glnu.norm} = \frac{1}{N_s^2} \sum_{i=1}^{N_g} r_{i.}^2$$

data	aggr. method	value	tol.	consensus
dig. phantom	2D, averaged	0.46	–	very strong
dig. phantom	2D, slice-merged	0.456	–	strong
dig. phantom	2.5D, direction-merged	0.413	–	strong
dig. phantom	2.5D, merged	0.412	–	strong
dig. phantom	3D, averaged	0.43	–	very strong
dig. phantom	3D, merged	0.43	–	very strong
config. A	2D, averaged	0.128	0.003	strong
config. A	2D, slice-merged	0.128	0.003	strong
config. A	2.5D, direction-merged	0.126	0.003	strong
config. A	2.5D, merged	0.126	0.003	strong
config. B	2D, averaged	0.145	0.001	strong
config. B	2D, slice-merged	0.145	0.001	strong
config. B	2.5D, direction-merged	0.137	0.001	strong
config. B	2.5D, merged	0.137	0.001	strong
config. C	3D, averaged	0.102	0.003	strong
config. C	3D, merged	0.102	0.003	very strong
config. D	3D, averaged	0.133	0.002	strong
config. D	3D, merged	0.134	0.002	strong
config. E	3D, averaged	0.135	0.003	strong
config. E	3D, merged	0.135	0.003	strong

Table 3.115 | Reference values for the *normalised grey level non-uniformity* feature.

3.7.11 Run length non-uniformity W92Y

This features assesses the distribution of runs over the run lengths[30]. The feature value is low when runs are equally distributed along run lengths. It is defined as:

$$F_{rlm.rlnu} = \frac{1}{N_s} \sum_{j=1}^{N_r} r_{.j}^2$$

data	aggr. method	value	tol.	consensus
dig. phantom	2D, averaged	6.12	—	very strong
dig. phantom	2D, slice-merged	21.6	—	strong
dig. phantom	2.5D, direction-merged	22.3	—	strong
dig. phantom	2.5D, merged	83.2	—	strong
dig. phantom	3D, averaged	26.9	—	very strong
dig. phantom	3D, merged	328	—	very strong
config. A	2D, averaged	1.65×10^3	10	strong
config. A	2D, slice-merged	6.6×10^3	30	strong
config. A	2.5D, direction-merged	4.27×10^4	200	strong
config. A	2.5D, merged	1.71×10^5	1×10^3	strong
config. B	2D, averaged	365	3	strong
config. B	2D, slice-merged	1.46×10^3	10	strong
config. B	2.5D, direction-merged	9.38×10^3	70	strong
config. B	2.5D, merged	3.75×10^4	300	strong
config. C	3D, averaged	1.8×10^4	500	strong
config. C	3D, merged	2.34×10^5	6×10^3	strong
config. D	3D, averaged	1.24×10^4	200	strong
config. D	3D, merged	1.6×10^5	3×10^3	strong
config. E	3D, averaged	1.66×10^4	300	strong
config. E	3D, merged	2.15×10^5	4×10^3	strong

Table 3.116 | Reference values for the *run length non-uniformity* feature.

3.7.12 Normalised run length non-uniformity IC23

This is normalised version of the *run length non-uniformity* feature. It is defined as:

$$F_{rlm.rlnu.norm} = \frac{1}{N_s^2} \sum_{j=1}^{N_r} r_{.j}^2$$

data	aggr. method	value	tol.	consensus
dig. phantom	2D, averaged	0.492	—	very strong
dig. phantom	2D, slice-merged	0.441	—	strong
dig. phantom	2.5D, direction-merged	0.461	—	strong
dig. phantom	2.5D, merged	0.445	—	strong
dig. phantom	3D, averaged	0.513	—	very strong
dig. phantom	3D, merged	0.501	—	very strong
config. A	2D, averaged	0.579	0.003	strong
config. A	2D, slice-merged	0.579	0.003	strong
config. A	2.5D, direction-merged	0.548	0.003	strong
config. A	2.5D, merged	0.548	0.003	strong
config. B	2D, averaged	0.578	0.001	strong
config. B	2D, slice-merged	0.578	0.001	strong

config. B	2.5D, direction-merged	0.533	0.001	strong
config. B	2.5D, merged	0.534	0.001	strong
config. C	3D, averaged	0.574	0.004	strong
config. C	3D, merged	0.575	0.004	strong
config. D	3D, averaged	0.5	0.001	strong
config. D	3D, merged	0.501	0.001	strong
config. E	3D, averaged	0.559	0.001	moderate
config. E	3D, merged	0.56	0.001	strong

Table 3.117 | Reference values for the *normalised run length non-uniformity* feature.

3.7.13 Run percentage 9ZK5

This feature measures the fraction of the number of realised runs and the maximum number of potential runs[30]. Strongly linear or highly uniform ROI volumes produce a low *run percentage*. It is defined as:

$$F_{rlm.r.perc} = \frac{N_s}{N_v}$$

As noted before, when this feature is calculated using a merged GLRLM, N_v should be the sum of the number of voxels of the underlying matrices to allow proper normalisation.

data	aggr. method	value	tol.	consensus
dig. phantom	2D, averaged	0.627	—	very strong
dig. phantom	2D, slice-merged	0.627	—	strong
dig. phantom	2.5D, direction-merged	0.632	—	strong
dig. phantom	2.5D, merged	0.632	—	strong
dig. phantom	3D, averaged	0.68	—	very strong
dig. phantom	3D, merged	0.68	—	very strong
config. A	2D, averaged	0.704	0.003	strong
config. A	2D, slice-merged	0.704	0.003	strong
config. A	2.5D, direction-merged	0.68	0.003	strong
config. A	2.5D, merged	0.68	0.003	strong
config. B	2D, averaged	0.681	0.002	strong
config. B	2D, slice-merged	0.681	0.002	strong
config. B	2.5D, direction-merged	0.642	0.002	strong
config. B	2.5D, merged	0.642	0.002	strong
config. C	3D, averaged	0.679	0.003	strong
config. C	3D, merged	0.679	0.003	strong
config. D	3D, averaged	0.554	0.005	strong
config. D	3D, merged	0.554	0.005	strong
config. E	3D, averaged	0.664	0.003	moderate
config. E	3D, merged	0.664	0.003	strong

Table 3.118 | Reference values for the *run percentage* feature.

3.7.14 Grey level variance 8CE5

This feature estimates the variance in runs over the grey levels. Let $p_{ij} = r_{ij}/N_s$ be the joint probability estimate for finding discretised grey level i with run length j. *Grey level variance* is then defined as:

$$F_{rlm.gl.var} = \sum_{i=1}^{N_g}\sum_{j=1}^{N_r}(i-\mu)^2 p_{ij}$$

Here, $\mu = \sum_{i=1}^{N_g}\sum_{j=1}^{N_r} i\, p_{ij}$.

data	aggr. method	value	tol.	consensus
dig. phantom	2D, averaged	3.35	–	very strong
dig. phantom	2D, slice-merged	3.37	–	strong
dig. phantom	2.5D, direction-merged	3.58	–	strong
dig. phantom	2.5D, merged	3.59	–	strong
dig. phantom	3D, averaged	3.46	–	very strong
dig. phantom	3D, merged	3.48	–	very strong
config. A	2D, averaged	33.7	0.6	strong
config. A	2D, slice-merged	33.7	0.6	strong
config. A	2.5D, direction-merged	29.1	0.6	strong
config. A	2.5D, merged	29.1	0.6	strong
config. B	2D, averaged	28.3	0.3	strong
config. B	2D, slice-merged	28.3	0.3	strong
config. B	2.5D, direction-merged	25.7	0.2	strong
config. B	2.5D, merged	25.7	0.2	strong
config. C	3D, averaged	101	3	strong
config. C	3D, merged	101	3	very strong
config. D	3D, averaged	31.5	0.4	strong
config. D	3D, merged	31.4	0.4	strong
config. E	3D, averaged	39.8	0.9	moderate
config. E	3D, merged	39.7	0.9	strong

Table 3.119 | Reference values for the *grey level variance* feature.

3.7.15 Run length variance SXLW

This feature estimates the variance in runs over the run lengths. As before let $p_{ij} = r_{ij}/N_s$. The feature is defined as:

$$F_{rlm.rl.var} = \sum_{i=1}^{N_g}\sum_{j=1}^{N_r}(j-\mu)^2 p_{ij}$$

Mean run length is defined as $\mu = \sum_{i=1}^{N_g}\sum_{j=1}^{N_r} j\, p_{ij}$.

data	aggr. method	value	tol.	consensus
dig. phantom	2D, averaged	0.761	—	very strong
dig. phantom	2D, slice-merged	0.778	—	strong
dig. phantom	2.5D, direction-merged	0.758	—	strong
dig. phantom	2.5D, merged	0.767	—	strong
dig. phantom	3D, averaged	0.574	—	very strong
dig. phantom	3D, merged	0.598	—	very strong
config. A	2D, averaged	0.828	0.008	strong
config. A	2D, slice-merged	0.826	0.008	strong
config. A	2.5D, direction-merged	0.916	0.011	strong
config. A	2.5D, merged	0.914	0.011	strong
config. B	2D, averaged	1.22	0.03	strong
config. B	2D, slice-merged	1.21	0.03	strong
config. B	2.5D, direction-merged	1.39	0.03	strong
config. B	2.5D, merged	1.39	0.03	strong
config. C	3D, averaged	1.12	0.02	strong
config. C	3D, merged	1.11	0.02	strong
config. D	3D, averaged	3.35	0.14	strong
config. D	3D, merged	3.29	0.13	strong
config. E	3D, averaged	1.26	0.05	strong
config. E	3D, merged	1.25	0.05	strong

Table 3.120 | Reference values for the *run length variance* feature.

3.7.16 Run entropy HJ90

Run entropy was investigated by Albregtsen et al.[3]. Again, let $p_{ij} = r_{ij}/N_s$. The entropy is then defined as:

$$F_{rlm.rl.entr} = -\sum_{i=1}^{N_g}\sum_{j=1}^{N_r} p_{ij} \log_2 p_{ij}$$

data	aggr. method	value	tol.	consensus
dig. phantom	2D, averaged	2.17	—	very strong
dig. phantom	2D, slice-merged	2.57	—	strong
dig. phantom	2.5D, direction-merged	2.52	—	strong
dig. phantom	2.5D, merged	2.76	—	strong
dig. phantom	3D, averaged	2.43	—	very strong
dig. phantom	3D, merged	2.62	—	very strong
config. A	2D, averaged	4.73	0.02	strong
config. A	2D, slice-merged	4.76	0.02	strong
config. A	2.5D, direction-merged	4.87	0.01	strong
config. A	2.5D, merged	4.87	0.01	strong
config. B	2D, averaged	4.53	0.02	strong

config. B	2D, slice-merged	4.58	0.01	strong
config. B	2.5D, direction-merged	4.84	0.01	strong
config. B	2.5D, merged	4.84	0.01	strong
config. C	3D, averaged	5.35	0.03	strong
config. C	3D, merged	5.35	0.03	very strong
config. D	3D, averaged	5.08	0.02	strong
config. D	3D, merged	5.08	0.02	strong
config. E	3D, averaged	4.87	0.03	strong
config. E	3D, merged	4.87	0.03	strong

Table 3.121 | Reference values for the *run entropy* feature.

3.8 Grey level size zone based features 9SAK

The grey level size zone matrix (GLSZM) counts the number of groups (or zones) of linked voxels [71]. Voxels are linked if the neighbouring voxel has an identical discretised grey level. Whether a voxel classifies as a neighbour depends on its connectedness. In a 3D approach to texture analysis we consider 26-connectedness, which indicates that a center voxel is linked to all of the 26 neighbouring voxels with the same grey level. In the 2 dimensional approach, 8-connectedness is used. A potential issue for the 2D approach is that voxels which may otherwise be considered to belong to the same zone by linking across slices, are now two or more separate zones within the slice plane. Whether this issue negatively affects predictive performance of GLSZM-based features or their reproducibility has not been determined.

Let M be the $N_g \times N_z$ grey level size zone matrix, where N_g is the number of discretised grey levels present in the ROI intensity mask and N_z the maximum zone size of any group of linked voxels. Element s_{ij} of M is then the number of zones with discretised grey level i and size j. Furthermore, let N_v be the number of voxels in the intensity mask and $N_s = \sum_{i=1}^{N_g}\sum_{j=1}^{N_z} s_{ij}$ be the total number of zones. Marginal sums can likewise be defined. Let $s_{i.} = \sum_{j=1}^{N_z} s_{ij}$ be the number of zones with discretised grey level i, regardless of size. Likewise, let $s_{.j} = \sum_{i=1}^{N_g} s_{ij}$ be the number of zones with size j, regardless of grey level. A two dimensional example is shown in Table 3.122.

Aggregating features

Three methods can be used to aggregate GLSZMs and arrive at a single feature value. A schematic example is shown in Figure 3.4. A feature may be aggregated as follows:

1. Features are computed from 2D matrices and averaged over slices (8QNN).
2. The feature is computed from a single matrix after merging all 2D matrices (62GR).
3. The feature is computed from a 3D matrix (KOBO).

Method 2 involves merging GLSZMs by summing the number of zones s_{ij} over the GLSZM for the different slices. Note that when matrices are merged, N_v should likewise be summed to retain consistency. Feature values may dependent strongly on the aggregation method.

Distances

The default neighbourhood for GLSZM is constructed using Chebyshev distance $\delta = 1$. Manhattan or Euclidean norms may also be used to construct a neighbourhood, and both lead to a 6-connected (3D) and 4-connected (2D) neighbourhoods. Larger distances are also technically possible, but will occasionally cause separate zones with the same intensity to be considered as belonging to the same zone. Using different neighbourhoods for determining voxel linkage is non-standard use, and we caution against it due to potential reproducibility issues.

Note on feature references

GLSZM feature definitions are based on the definitions of GLRLM features [71]. Hence, references may be found in the section on GLRLM (3.7).

1	2	2	3
1	2	3	3
4	2	4	1
4	1	2	3

(a) Grey levels

		Zone size j				
		1	2	3	4	5
i	1	2	1	0	0	0
	2	0	0	0	0	1
	3	1	0	1	0	0
	4	1	1	0	0	0

(b) Grey level size zone matrix

Table 3.122 | Original image with grey levels (a); and corresponding grey level size zone matrix (GLSZM) under 8-connectedness (b). Element $s(i,j)$ of the GLSZM indicates the number of times a zone of j linked pixels and grey level i occurs within the image.

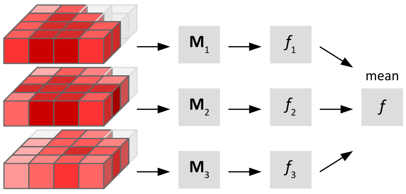

(a) 2D: by slice, without merging

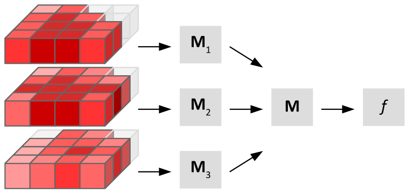

(b) 2.5D: by slice, with merging

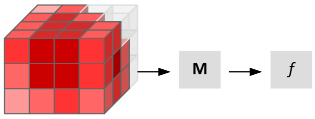

(c) 3D: as volume

Figure 3.4 | Approaches to calculating grey level size zone matrix-based features. M_k are texture matrices calculated for slice k (if applicable), and f_k is the corresponding feature value. In (b) the matrices from the different slices are merged prior to feature calculation.

3.8.1 Small zone emphasis 5QRC

This feature emphasises small zones. It is defined as:

$$F_{szm.sze} = \frac{1}{N_s} \sum_{j=1}^{N_z} \frac{s_{.j}}{j^2}$$

data	aggr. method	value	tol.	consensus
dig. phantom	2D	0.363	–	strong
dig. phantom	2.5D	0.368	–	strong
dig. phantom	3D	0.255	–	very strong
config. A	2D	0.688	0.003	strong
config. A	2.5D	0.68	0.003	strong
config. B	2D	0.745	0.003	strong
config. B	2.5D	0.741	0.003	strong
config. C	3D	0.695	0.001	strong
config. D	3D	0.637	0.005	strong
config. E	3D	0.676	0.003	strong

Table 3.123 | Reference values for the *small zone emphasis* feature.

3.8.2 Large zone emphasis 48P8

This feature emphasises large zones. It is defined as:

$$F_{szm.lze} = \frac{1}{N_s} \sum_{j=1}^{N_z} j^2 s_{.j}$$

data	aggr. method	value	tol.	consensus
dig. phantom	2D	43.9	–	strong
dig. phantom	2.5D	34.2	–	strong
dig. phantom	3D	550	–	very strong
config. A	2D	625	9	strong
config. A	2.5D	675	8	strong
config. B	2D	439	8	strong
config. B	2.5D	444	8	strong
config. C	3D	3.89×10^4	900	strong
config. D	3D	9.91×10^4	2.8×10^3	strong
config. E	3D	5.86×10^4	800	strong

Table 3.124 | Reference values for the *large zone emphasis* feature.

3.8.3 Low grey level zone emphasis XMSY

This feature is a grey level analogue to *small zone emphasis*. Instead of small zone sizes, low grey levels are emphasised. The feature is defined as:

$$F_{szm.lgze} = \frac{1}{N_s}\sum_{i=1}^{N_g}\frac{s_{i.}}{i^2}$$

data	aggr. method	value	tol.	consensus
dig. phantom	2D	0.371	—	strong
dig. phantom	2.5D	0.368	—	strong
dig. phantom	3D	0.253	—	very strong
config. A	2D	0.0368	0.0005	strong
config. A	2.5D	0.0291	0.0005	strong
config. B	2D	0.0475	0.001	strong
config. B	2.5D	0.0387	0.001	strong
config. C	3D	0.00235	6×10^{-5}	strong
config. D	3D	0.0409	0.0005	strong
config. E	3D	0.034	0.0004	strong

Table 3.125 | Reference values for the *low grey level emphasis* feature.

3.8.4 High grey level zone emphasis 5GN9

The *high grey level zone emphasis* feature is a grey level analogue to *large zone emphasis*. The feature emphasises high grey levels, and is defined as:

$$F_{szm.hgze} = \frac{1}{N_s}\sum_{i=1}^{N_g} i^2 s_{i.}$$

data	aggr. method	value	tol.	consensus
dig. phantom	2D	16.4	—	strong
dig. phantom	2.5D	16.2	—	strong
dig. phantom	3D	15.6	—	very strong
config. A	2D	363	3	strong
config. A	2.5D	370	3	strong
config. B	2D	284	11	strong
config. B	2.5D	284	11	strong
config. C	3D	971	7	strong
config. D	3D	188	10	strong
config. E	3D	286	6	strong

Table 3.126 | Reference values for the *high grey level emphasis* feature.

3.8.5 Small zone low grey level emphasis 5RAI

This feature emphasises zone counts within the upper left quadrant of the GLSZM, where small zone sizes and low grey levels are located. It is defined as:

$$F_{szm.szlge} = \frac{1}{N_s} \sum_{i=1}^{N_g} \sum_{j=1}^{N_z} \frac{s_{ij}}{i^2 j^2}$$

data	aggr. method	value	tol.	consensus
dig. phantom	2D	0.0259	—	strong
dig. phantom	2.5D	0.0295	—	strong
dig. phantom	3D	0.0256	—	very strong
config. A	2D	0.0298	0.0005	strong
config. A	2.5D	0.0237	0.0005	strong
config. B	2D	0.0415	0.0008	strong
config. B	2.5D	0.0335	0.0009	strong
config. C	3D	0.0016	4×10^{-5}	strong
config. D	3D	0.0248	0.0004	strong
config. E	3D	0.0224	0.0004	strong

Table 3.127 | Reference values for the *small zone low grey level emphasis* feature.

3.8.6 Small zone high grey level emphasis HW1V

This feature emphasises zone counts in the lower left quadrant of the GLSZM, where small zone sizes and high grey levels are located. The feature is defined as:

$$F_{szm.szhge} = \frac{1}{N_s} \sum_{i=1}^{N_g} \sum_{j=1}^{N_z} \frac{i^2 s_{ij}}{j^2}$$

data	aggr. method	value	tol.	consensus
dig. phantom	2D	10.3	—	strong
dig. phantom	2.5D	9.87	—	strong
dig. phantom	3D	2.76	—	very strong
config. A	2D	226	1	strong
config. A	2.5D	229	1	strong
config. B	2D	190	7	strong
config. B	2.5D	190	7	strong
config. C	3D	657	4	strong
config. D	3D	117	7	strong
config. E	3D	186	4	strong

Table 3.128 | Reference values for the *small zone high grey level emphasis* feature.

3.8.7 Large zone low grey level emphasis YH51

This feature emphasises zone counts in the upper right quadrant of the GLSZM, where large zone sizes and low grey levels are located. The feature is defined as:

$$F_{szm.lzlge} = \frac{1}{N_s} \sum_{i=1}^{N_g} \sum_{j=1}^{N_z} \frac{j^2 s_{ij}}{i^2}$$

data	aggr. method	value	tol.	consensus
dig. phantom	2D	40.4	—	strong
dig. phantom	2.5D	30.6	—	strong
dig. phantom	3D	503	—	very strong
config. A	2D	1.35	0.03	strong
config. A	2.5D	1.44	0.02	strong
config. B	2D	1.15	0.04	strong
config. B	2.5D	1.16	0.04	strong
config. C	3D	21.6	0.5	strong
config. D	3D	241	14	strong
config. E	3D	105	4	strong

Table 3.129 | Reference values for the *large zone low grey level emphasis* feature.

3.8.8 Large zone high grey level emphasis J17V

This feature emphasises zone counts in the lower right quadrant of the GLSZM, where large zone sizes and high grey levels are located. The feature is defined as:

$$F_{szm.lzhge} = \frac{1}{N_s} \sum_{i=1}^{N_g} \sum_{j=1}^{N_z} i^2 j^2 s_{ij}$$

data	aggr. method	value	tol.	consensus
dig. phantom	2D	113	—	strong
dig. phantom	2.5D	107	—	strong
dig. phantom	3D	1.49×10^3	—	very strong
config. A	2D	3.16×10^5	5×10^3	strong
config. A	2.5D	3.38×10^5	5×10^3	strong
config. B	2D	1.81×10^5	3×10^3	strong
config. B	2.5D	1.81×10^5	3×10^3	strong
config. C	3D	7.07×10^7	1.5×10^6	strong
config. D	3D	4.14×10^7	3×10^5	strong
config. E	3D	3.36×10^7	3×10^5	strong

Table 3.130 | Reference values for the *large zone high grey level emphasis* feature.

3.8.9 Grey level non-uniformity JNSA

This feature assesses the distribution of zone counts over the grey values. The feature value is low when zone counts are equally distributed along grey levels. The feature is defined as:

$$F_{szm.glnu} = \frac{1}{N_s} \sum_{i=1}^{N_g} s_{i.}^2$$

data	aggr. method	value	tol.	consensus
dig. phantom	2D	1.41	–	strong
dig. phantom	2.5D	5.44	–	strong
dig. phantom	3D	1.4	–	very strong
config. A	2D	82.2	0.1	strong
config. A	2.5D	1.8×10^3	10	strong
config. B	2D	20.5	0.1	strong
config. B	2.5D	437	3	strong
config. C	3D	195	6	strong
config. D	3D	212	6	very strong
config. E	3D	231	6	strong

Table 3.131 | Reference values for the *grey level non-uniformity* feature.

3.8.10 Normalised grey level non-uniformity Y1RO

This is a normalised version of the *grey level non-uniformity* feature. It is defined as:

$$F_{szm.glnu.norm} = \frac{1}{N_s^2} \sum_{i=1}^{N_g} s_{i.}^2$$

data	aggr. method	value	tol.	consensus
dig. phantom	2D	0.323	–	strong
dig. phantom	2.5D	0.302	–	strong
dig. phantom	3D	0.28	–	very strong
config. A	2D	0.0728	0.0014	strong
config. A	2.5D	0.0622	0.0007	strong
config. B	2D	0.0789	0.001	strong
config. B	2.5D	0.0613	0.0005	strong
config. C	3D	0.0286	0.0003	strong
config. D	3D	0.0491	0.0008	strong
config. E	3D	0.0414	0.0003	strong

Table 3.132 | Reference values for the *normalised grey level non-uniformity* feature.

3.8.11 Zone size non-uniformity 4JP3

This features assesses the distribution of zone counts over the different zone sizes. *Zone size non-uniformity* is low when zone counts are equally distributed along zone sizes. It is defined as:

$$F_{szm.zsnu} = \frac{1}{N_s}\sum_{j=1}^{N_z} s_{.j}^2$$

data	aggr. method	value	tol.	consensus
dig. phantom	2D	1.49	–	strong
dig. phantom	2.5D	3.44	–	strong
dig. phantom	3D	1	–	very strong
config. A	2D	479	4	strong
config. A	2.5D	1.24×10^4	100	strong
config. B	2D	140	3	strong
config. B	2.5D	3.63×10^3	70	strong
config. C	3D	3.04×10^3	100	strong
config. D	3D	1.63×10^3	10	strong
config. E	3D	2.37×10^3	40	strong

Table 3.133 | Reference values for the *zone size non-uniformity* feature.

3.8.12 Normalised zone size non-uniformity VB3A

This is a normalised version of *zone size non-uniformity*. It is defined as:

$$F_{szm.zsnu.norm} = \frac{1}{N_s^2}\sum_{i=1}^{N_z} s_{.j}^2$$

data	aggr. method	value	tol.	consensus
dig. phantom	2D	0.333	–	strong
dig. phantom	2.5D	0.191	–	strong
dig. phantom	3D	0.2	–	very strong
config. A	2D	0.44	0.004	strong
config. A	2.5D	0.427	0.004	strong
config. B	2D	0.521	0.004	strong
config. B	2.5D	0.509	0.004	strong
config. C	3D	0.447	0.001	strong
config. D	3D	0.377	0.006	strong
config. E	3D	0.424	0.004	strong

Table 3.134 | Reference values for the *normalised zone size non-uniformity* feature.

3.8.13 Zone percentage P30P

This feature measures the fraction of the number of realised zones and the maximum number of potential zones. Highly uniform ROIs produce a low *zone percentage*. It is defined as:

$$F_{szm.z.perc} = \frac{N_s}{N_v}$$

data	aggr. method	value	tol.	consensus
dig. phantom	2D	0.24	—	strong
dig. phantom	2.5D	0.243	—	strong
dig. phantom	3D	0.0676	—	very strong
config. A	2D	0.3	0.003	strong
config. A	2.5D	0.253	0.004	strong
config. B	2D	0.324	0.001	strong
config. B	2.5D	0.26	0.002	strong
config. C	3D	0.148	0.003	very strong
config. D	3D	0.0972	0.0007	strong
config. E	3D	0.126	0.001	strong

Table 3.135 | Reference values for the *zone percentage* feature.

3.8.14 Grey level variance BYLV

This feature estimates the variance in zone counts over the grey levels. Let $p_{ij} = s_{ij}/N_s$ be the joint probability estimate for finding zones with discretised grey level i and size j. The feature is then defined as:

$$F_{szm.gl.var} = \sum_{i=1}^{N_g} \sum_{j=1}^{N_z} (i - \mu)^2 p_{ij}$$

Here, $\mu = \sum_{i=1}^{N_g} \sum_{j=1}^{N_z} i\, p_{ij}$.

data	aggr. method	value	tol.	consensus
dig. phantom	2D	3.97	—	strong
dig. phantom	2.5D	3.92	—	strong
dig. phantom	3D	2.64	—	very strong
config. A	2D	42.7	0.7	strong
config. A	2.5D	47.9	0.4	strong
config. B	2D	36.1	0.3	strong
config. B	2.5D	41	0.7	strong
config. C	3D	106	1	strong
config. D	3D	32.7	1.6	strong
config. E	3D	50.8	0.9	strong

Table 3.136 | Reference values for the *grey level variance* feature.

3.8.15 Zone size variance 3NSA

This feature estimates the variance in zone counts over the different zone sizes. As before let $p_{ij} = s_{ij}/N_s$. The feature is defined as:

$$F_{szm.zs.var} = \sum_{i=1}^{N_g}\sum_{j=1}^{N_z}(j-\mu)^2 p_{ij}$$

Mean zone size is defined as $\mu = \sum_{i=1}^{N_g}\sum_{j=1}^{N_z} j\, p_{ij}$.

data	aggr. method	value	tol.	consensus
dig. phantom	2D	21	–	strong
dig. phantom	2.5D	17.3	–	strong
dig. phantom	3D	331	–	very strong
config. A	2D	609	9	strong
config. A	2.5D	660	8	strong
config. B	2D	423	8	strong
config. B	2.5D	429	8	strong
config. C	3D	3.89×10^4	900	strong
config. D	3D	9.9×10^4	2.8×10^3	strong
config. E	3D	5.85×10^4	800	strong

Table 3.137 | Reference values for the *zone size variance* feature.

3.8.16 Zone size entropy GU8N

Let $p_{ij} = s_{ij}/N_s$. *Zone size entropy* is then defined as:

$$F_{szm.zs.entr} = -\sum_{i=1}^{N_g}\sum_{j=1}^{N_z} p_{ij}\log_2 p_{ij}$$

data	aggr. method	value	tol.	consensus
dig. phantom	2D	1.93	–	strong
dig. phantom	2.5D	3.08	–	strong
dig. phantom	3D	2.32	–	very strong
config. A	2D	5.92	0.02	strong
config. A	2.5D	6.39	0.01	strong
config. B	2D	5.29	0.01	strong
config. B	2.5D	5.98	0.02	strong
config. C	3D	7	0.01	strong
config. D	3D	6.52	0.01	strong
config. E	3D	6.57	0.01	strong

Table 3.138 | Reference values for the *zone size entropy* feature.

3.9 Grey level distance zone based features VMDZ

The grey level distance zone matrix (GLDZM) counts the number of groups (or zones) of linked voxels which share a specific discretised grey level value and possess the same distance to ROI edge [71]. The GLDZM thus captures the relation between location and grey level. Two maps are required to calculate the GLDZM. The first is a grey level zone map, which is identical to the one created for the grey level size zone matrix (GLSZM), see Section 3.8. The second is a distance map, which will be described in detail later.

As with GSLZM, neighbouring voxels are linked if they share the same grey level value. Whether a voxel classifies as a neighbour depends on its connectedness. We consider 26-connectedness for a 3D approach and 8-connectedness in the 2D approach.

The distance to the ROI edge is defined according to 6 and 4-connectedness for 3D and 2D, respectively. Because of the connectedness definition used, the distance of a voxel to the outer border is equal to the minimum number edges of neighbouring voxels that need to be crossed to reach the ROI edge. The distance for a linked group of voxels with the same grey value is equal to the minimum distance for the respective voxels in the distance map.

Our definition deviates from the original by Thibault et al. [71]. The original was defined in a rectangular 2D image, whereas ROIs are rarely rectangular cuboids. Approximating distance using Chamfer maps is then no longer a fast and easy solution. Determining distance iteratively in 6 or 4-connectedness is a relatively efficient solution, implemented as follows:

1. The ROI mask is morphologically eroded using the appropriate (6 or 4-connected) structure element.
2. All eroded ROI voxels are updated in the distance map by adding 1.
3. The above steps are performed iteratively until the ROI mask is empty.

A second difference with the original definition is that the lowest possible distance is 1 instead of 0 for voxels directly on the ROI edge. This prevents division by 0 for some features.

Let $\mathbf{M}$ be the $N_g \times N_d$ grey level size zone matrix, where N_g is the number of discretised grey levels present in the ROI intensity mask and N_d the largest distance of any zone. Element $d_{ij} = d(i,j)$ of $\mathbf{M}$ is then number of zones with discretised grey level i and distance j. Furthermore, let N_v be the number of voxels and $N_s = \sum_{i=1}^{N_g}\sum_{j=1}^{N_d} d_{ij}$ be the total zone count. Marginal sums can likewise be defined. Let $d_{i.} = \sum_{j=1}^{N_d} d_{ij}$ be the number of zones with discretised grey level i, regardless of distance. Likewise, let $d_{.j} = \sum_{i=1}^{N_g} d_{ij}$ be the number of zones with distance j, regardless of grey level. A two dimensional example is shown in Table 3.139.

Morphological and intensity masks.

The GLDZM is special in that it uses both ROI masks. The distance map is determined using the morphological ROI mask, whereas the intensity mask is used for determining the zones, as with the GLSZM.

Aggregating features

Three methods can be used to aggregate GLDZMs and arrive at a single feature value. A schematic example was previously shown in Figure 3.4. A feature may be aggregated as follows:

1	2	2	3
1	2	3	3
4	2	4	1
4	1	2	3

(a) Grey levels

1	1	1	1
1	2	2	1
1	2	2	1
1	1	1	1

(b) Distance map

		j = 1	j = 2
i	1	3	0
	2	2	0
	3	2	0
	4	1	1

(c) Grey level distance zone matrix

Table 3.139 | Original image with grey levels (a); corresponding distance map for distance to border (b); and corresponding grey level distance zone matrix (GLDZM) under 4-connectedness (c). Element $d(i,j)$ of the GLDZM indicates the number of times a zone with grey level i and a minimum distance to border j occurs within the image.

1. Features are computed from 2D matrices and averaged over slices (8QNN).
2. The feature is computed from a single matrix after merging all 2D matrices (62GR).
3. The feature is computed from a 3D matrix (KOBO).

Method 2 involves merging GLDZMs by summing the number of zones d_{ij} over the GLDZM for the different slices. Note that when matrices are merged, N_v should likewise be summed to retain consistency. Feature values may dependent strongly on the aggregation method.

Distances

In addition to the use of different distance norms to determine voxel linkage, as described in section 3.8, different distance norms may be used to determine distance of zones to the boundary. The default is to use the Manhattan norm which allows for a computationally efficient implementation, as described above. A similar implementation is possible using the Chebyshev norm, as it merely changes connectedness of the structure element. Implementations using an Euclidean distance norm are less efficient as this demands searching for the nearest non-ROI voxel for each of the N_v voxels in the ROI. An added issue is that Euclidean norms may lead to a wide range of different distances j that require rounding before constructing the grey level distance zone matrix M. Using different distance norms is non-standard use, and we caution against it due to potential reproducibility issues.

Note on feature references

GLDZM feature definitions are based on the definitions of GLRLM features[71]. Hence, references may be found in the section on GLRLM (3.7).

3.9.1 Small distance emphasis 0GBI

This feature emphasises small distances. It is defined as:

$$F_{dzm.sde} = \frac{1}{N_s}\sum_{j=1}^{N_d}\frac{d_{.j}}{j^2}$$

data	aggr. method	value	tol.	consensus
dig. phantom	2D	0.946	–	strong
dig. phantom	2.5D	0.917	–	moderate
dig. phantom	3D	1	–	very strong
config. A	2D	0.192	0.006	strong
config. A	2.5D	0.168	0.005	strong
config. B	2D	0.36	0.005	strong
config. B	2.5D	0.329	0.004	strong
config. C	3D	0.531	0.006	strong
config. D	3D	0.579	0.004	strong
config. E	3D	0.527	0.004	moderate

Table 3.140 | Reference values for the *small distance emphasis* feature.

3.9.2 Large distance emphasis MB4I

This feature emphasises large distances. It is defined as:

$$F_{dzm.lde} = \frac{1}{N_s}\sum_{j=1}^{N_d} j^2 d_{.j}$$

data	aggr. method	value	tol.	consensus
dig. phantom	2D	1.21	–	strong
dig. phantom	2.5D	1.33	–	moderate
dig. phantom	3D	1	–	very strong
config. A	2D	161	1	moderate
config. A	2.5D	178	1	moderate
config. B	2D	31.6	0.2	moderate
config. B	2.5D	34.3	0.2	moderate
config. C	3D	11	0.3	strong
config. D	3D	10.3	0.1	strong
config. E	3D	12.6	0.1	moderate

Table 3.141 | Reference values for the *large distance emphasis* feature.

3.9.3 Low grey level zone emphasis S1RA

This feature is a grey level analogue to *small distance emphasis*. Instead of small zone distances, low grey levels are emphasised. The feature is defined as:

$$F_{dzm.lgze} = \frac{1}{N_s}\sum_{i=1}^{N_g}\frac{d_{i.}}{i^2}$$

data	aggr. method	value	tol.	consensus
dig. phantom	2D	0.371	—	strong
dig. phantom	2.5D	0.368	—	moderate
dig. phantom	3D	0.253	—	very strong
config. A	2D	0.0368	0.0005	strong
config. A	2.5D	0.0291	0.0005	strong
config. B	2D	0.0475	0.001	strong
config. B	2.5D	0.0387	0.001	strong
config. C	3D	0.00235	6×10^{-5}	strong
config. D	3D	0.0409	0.0005	strong
config. E	3D	0.034	0.0004	moderate

Table 3.142 | Reference values for the *low grey level emphasis* feature.

3.9.4 High grey level zone emphasis K26C

The *high grey level zone emphasis* feature is a grey level analogue to *large distance emphasis*. The feature emphasises high grey levels, and is defined as:

$$F_{dzm.hgze} = \frac{1}{N_s}\sum_{i=1}^{N_g} i^2 d_{i.}$$

data	aggr. method	value	tol.	consensus
dig. phantom	2D	16.4	—	strong
dig. phantom	2.5D	16.2	—	moderate
dig. phantom	3D	15.6	—	very strong
config. A	2D	363	3	strong
config. A	2.5D	370	3	strong
config. B	2D	284	11	strong
config. B	2.5D	284	11	strong
config. C	3D	971	7	strong
config. D	3D	188	10	strong
config. E	3D	286	6	strong

Table 3.143 | Reference values for the *high grey level emphasis* feature.

3.9.5 Small distance low grey level emphasis RUVG

This feature emphasises runs in the upper left quadrant of the GLDZM, where small zone distances and low grey levels are located. It is defined as:

$$F_{dzm.sdlge} = \frac{1}{N_s} \sum_{i=1}^{N_g} \sum_{j=1}^{N_d} \frac{d_{ij}}{i^2 j^2}$$

data	aggr. method	value	tol.	consensus
dig. phantom	2D	0.367	–	strong
dig. phantom	2.5D	0.362	–	moderate
dig. phantom	3D	0.253	–	very strong
config. A	2D	0.00913	0.00023	strong
config. A	2.5D	0.00788	0.00022	strong
config. B	2D	0.0192	0.0005	strong
config. B	2.5D	0.0168	0.0005	strong
config. C	3D	0.00149	4×10^{-5}	strong
config. D	3D	0.0302	0.0006	strong
config. E	3D	0.0228	0.0003	moderate

Table 3.144 | Reference values for the *small distance low grey level emphasis* feature.

3.9.6 Small distance high grey level emphasis DKNJ

This feature emphasises runs in the lower left quadrant of the GLDZM, where small zone distances and high grey levels are located. *Small distance high grey level emphasis* is defined as:

$$F_{dzm.sdhge} = \frac{1}{N_s} \sum_{i=1}^{N_g} \sum_{j=1}^{N_d} \frac{i^2 d_{ij}}{j^2}$$

data	aggr. method	value	tol.	consensus
dig. phantom	2D	15.2	–	strong
dig. phantom	2.5D	14.3	–	moderate
dig. phantom	3D	15.6	–	very strong
config. A	2D	60.1	3.3	strong
config. A	2.5D	49.5	2.8	strong
config. B	2D	95.7	5.5	strong
config. B	2.5D	81.4	4.6	strong
config. C	3D	476	11	strong
config. D	3D	99.3	5.1	strong
config. E	3D	136	4	moderate

Table 3.145 | Reference values for the *small distance high grey level emphasis* feature.

3.9.7 Large distance low grey level emphasis A7WM

This feature emphasises runs in the upper right quadrant of the GLDZM, where large zone distances and low grey levels are located. The feature is defined as:

$$F_{dzm.ldlge} = \frac{1}{N_s}\sum_{i=1}^{N_g}\sum_{j=1}^{N_d}\frac{j^2 d_{ij}}{i^2}$$

data	aggr. method	value	tol.	consensus
dig. phantom	2D	0.386	—	strong
dig. phantom	2.5D	0.391	—	moderate
dig. phantom	3D	0.253	—	very strong
config. A	2D	2.96	0.02	moderate
config. A	2.5D	2.31	0.01	moderate
config. B	2D	0.934	0.018	moderate
config. B	2.5D	0.748	0.017	moderate
config. C	3D	0.0154	0.0005	strong
config. D	3D	0.183	0.004	strong
config. E	3D	0.179	0.004	moderate

Table 3.146 | Reference values for the *large distance low grey level emphasis* feature.

3.9.8 Large distance high grey level emphasis KLTH

This feature emphasises runs in the lower right quadrant of the GLDZM, where large zone distances and high grey levels are located. The *large distance high grey level emphasis* feature is defined as:

$$F_{dzm.ldhge} = \frac{1}{N_s}\sum_{i=1}^{N_g}\sum_{j=1}^{N_d} i^2 j^2 d_{ij}$$

data	aggr. method	value	tol.	consensus
dig. phantom	2D	21.3	—	strong
dig. phantom	2.5D	23.7	—	moderate
dig. phantom	3D	15.6	—	very strong
config. A	2D	7.01×10^4	100	moderate
config. A	2.5D	7.95×10^4	100	moderate
config. B	2D	1.06×10^4	300	strong
config. B	2.5D	1.16×10^4	400	strong
config. C	3D	1.34×10^4	200	strong
config. D	3D	2.62×10^3	110	strong
config. E	3D	4.85×10^3	60	moderate

Table 3.147 | Reference values for the *large distance high grey level emphasis* feature.

3.9.9 Grey level non-uniformity VFT7

This feature measures the distribution of zone counts over the grey values. *Grey level non-uniformity* is low when zone counts are equally distributed along grey levels. The feature is defined as:

$$F_{dzm.glnu} = \frac{1}{N_s}\sum_{i=1}^{N_g} d_{i.}^2$$

data	aggr. method	value	tol.	consensus
dig. phantom	2D	1.41	—	strong
dig. phantom	2.5D	5.44	—	moderate
dig. phantom	3D	1.4	—	very strong
config. A	2D	82.2	0.1	strong
config. A	2.5D	1.8×10^3	10	strong
config. B	2D	20.5	0.1	strong
config. B	2.5D	437	3	strong
config. C	3D	195	6	strong
config. D	3D	212	6	strong
config. E	3D	231	6	moderate

Table 3.148 | Reference values for the *grey level non-uniformity* feature.

3.9.10 Normalised grey level non-uniformity 7HP3

This is a normalised version of the *grey level non-uniformity* feature. It is defined as:

$$F_{dzm.glnu.norm} = \frac{1}{N_s^2}\sum_{i=1}^{N_g} d_{i.}^2$$

data	aggr. method	value	tol.	consensus
dig. phantom	2D	0.323	—	strong
dig. phantom	2.5D	0.302	—	moderate
dig. phantom	3D	0.28	—	very strong
config. A	2D	0.0728	0.0014	strong
config. A	2.5D	0.0622	0.0007	strong
config. B	2D	0.0789	0.001	strong
config. B	2.5D	0.0613	0.0005	strong
config. C	3D	0.0286	0.0003	strong
config. D	3D	0.0491	0.0008	strong
config. E	3D	0.0414	0.0003	moderate

Table 3.149 | Reference values for the *normalised grey level non-uniformity* feature.

3.9.11 Zone distance non-uniformity V294

Zone distance non-uniformity measures the distribution of zone counts over the different zone distances. *Zone distance non-uniformity* is low when zone counts are equally distributed along zone distances. It is defined as:

$$F_{dzm.zdnu} = \frac{1}{N_s} \sum_{j=1}^{N_d} d_{.j}^2$$

data	aggr. method	value	tol.	consensus
dig. phantom	2D	3.79	—	strong
dig. phantom	2.5D	14.4	—	moderate
dig. phantom	3D	5	—	very strong
config. A	2D	64	0.4	moderate
config. A	2.5D	1.57×10^3	10	strong
config. B	2D	39.8	0.3	moderate
config. B	2.5D	963	6	moderate
config. C	3D	1.87×10^3	40	strong
config. D	3D	1.37×10^3	20	strong
config. E	3D	1.5×10^3	30	moderate

Table 3.150 | Reference values for the *zone distance non-uniformity* feature.

3.9.12 Normalised zone distance non-uniformity IATH

This is a normalised version of the *zone distance non-uniformity* feature. It is defined as:

$$F_{dzm.zdnu.norm} = \frac{1}{N_s^2} \sum_{i=1}^{N_d} d_{.j}^2$$

data	aggr. method	value	tol.	consensus
dig. phantom	2D	0.898	—	strong
dig. phantom	2.5D	0.802	—	moderate
dig. phantom	3D	1	—	very strong
config. A	2D	0.0716	0.0022	strong
config. A	2.5D	0.0543	0.0014	strong
config. B	2D	0.174	0.003	strong
config. B	2.5D	0.135	0.001	strong
config. C	3D	0.274	0.005	strong
config. D	3D	0.317	0.004	strong
config. E	3D	0.269	0.003	moderate

Table 3.151 | Reference values for the *normalised zone distance non-uniformity* feature.

3.9.13 Zone percentage VIWW

This feature measures the fraction of the number of realised zones and the maximum number of potential zones. Highly uniform ROIs produce a low *zone percentage*. It is defined as:

$$F_{dzm.z.perc} = \frac{N_s}{N_v}$$

data	aggr. method	value	tol.	consensus
dig. phantom	2D	0.24	—	strong
dig. phantom	2.5D	0.243	—	moderate
dig. phantom	3D	0.0676	—	very strong
config. A	2D	0.3	0.003	strong
config. A	2.5D	0.253	0.004	moderate
config. B	2D	0.324	0.001	strong
config. B	2.5D	0.26	0.002	moderate
config. C	3D	0.148	0.003	strong
config. D	3D	0.0972	0.0007	strong
config. E	3D	0.126	0.001	moderate

Table 3.152 | Reference values for the *zone percentage* feature.

3.9.14 Grey level variance QK93

This feature estimates the variance in zone counts over the grey levels. Let $p_{ij} = d_{ij}/N_s$ be the joint probability estimate for finding zones with discretised grey level i at distance j. The feature is then defined as:

$$F_{dzm.gl.var} = \sum_{i=1}^{N_g} \sum_{j=1}^{N_d} (i - \mu)^2 p_{ij}$$

Here, $\mu = \sum_{i=1}^{N_g} \sum_{j=1}^{N_d} i\, p_{ij}$.

data	aggr. method	value	tol.	consensus
dig. phantom	2D	3.97	—	strong
dig. phantom	2.5D	3.92	—	moderate
dig. phantom	3D	2.64	—	very strong
config. A	2D	42.7	0.7	moderate
config. A	2.5D	47.9	0.4	strong
config. B	2D	36.1	0.3	moderate
config. B	2.5D	41	0.7	strong
config. C	3D	106	1	strong
config. D	3D	32.7	1.6	strong
config. E	3D	50.8	0.9	strong

Table 3.153 | Reference values for the *grey level variance* feature.

3.9.15 Zone distance variance 7WT1

This feature estimates the variance in zone counts for the different zone distances. As before let $p_{ij} = d_{ij}/N_s$. The feature is defined as:

$$F_{dzm.zd.var} = \sum_{i=1}^{N_g}\sum_{j=1}^{N_d}(j-\mu)^2 p_{ij}$$

Mean zone size is defined as $\mu = \sum_{i=1}^{N_g}\sum_{j=1}^{N_d} j\,p_{ij}$.

data	aggr. method	value	tol.	consensus
dig. phantom	2D	0.051	—	strong
dig. phantom	2.5D	0.0988	—	moderate
dig. phantom	3D	0	—	very strong
config. A	2D	69.4	0.1	moderate
config. A	2.5D	78.9	0.1	moderate
config. B	2D	13.5	0.1	moderate
config. B	2.5D	15	0.1	moderate
config. C	3D	4.6	0.06	strong
config. D	3D	4.61	0.04	strong
config. E	3D	5.56	0.05	strong

Table 3.154 | Reference values for the *zone distance variance* feature.

3.9.16 Zone distance entropy GBDU

Again, let $p_{ij} = d_{ij}/N_s$. Zone distance entropy is then defined as:

$$F_{dzm.zd.entr} = -\sum_{i=1}^{N_g}\sum_{j=1}^{N_d} p_{ij}\log_2 p_{ij}$$

data	aggr. method	value	tol.	consensus
dig. phantom	2D	1.73	—	strong
dig. phantom	2.5D	2	—	moderate
dig. phantom	3D	1.92	—	very strong
config. A	2D	8	0.04	strong
config. A	2.5D	8.87	0.03	strong
config. B	2D	6.47	0.03	strong
config. B	2.5D	7.58	0.01	moderate
config. C	3D	7.56	0.03	strong
config. D	3D	6.61	0.03	strong
config. E	3D	7.06	0.01	moderate

Table 3.155 | Reference values for the *zone distance entropy* feature.

3.10 Neighbourhood grey tone difference based features IPET

Amadasun and King [5] introduced an alternative to the grey level co-occurrence matrix. The neighbourhood grey tone difference matrix (NGTDM) contains the sum of grey level differences of pixels/voxels with discretised grey level i and the average discretised grey level of neighbouring pixels/voxels within a Chebyshev distance δ. For 3D volumes, we can extend the original definition by Amadasun and King. Let $X_{d,k}$ be the discretised grey level of a voxel at position $\mathbf{k} = (k_x, k_y, k_z)$. Then the average grey level within a neighbourhood centred at (k_x, k_y, k_z), but excluding (k_x, k_y, k_z) itself is:

$$\overline{X}_k = \frac{1}{W} \sum_{m_z=-\delta}^{\delta} \sum_{m_y=-\delta}^{\delta} \sum_{m_x=-\delta}^{\delta} X_d(k_x+m_x, k_y+m_y, k_z+m_z)$$

$$(m_x, m_y, m_z) \neq (0,0,0)$$

$W = (2\delta + 1)^3 - 1$ is the size of the 3D neighbourhood. For 2D $W = (2\delta + 1)^2 - 1$, and averages are not calculated between different slices. Neighbourhood grey tone difference s_i for discretised grey level i is then:

$$s_i = \sum_{k}^{N_v} |i - \overline{X}_k| \left[X_d(\mathbf{k}) = i \text{ and } k \text{ has a valid neighbourhood}\right]$$

Here, $[\ldots]$ is an Iverson bracket, which is 1 if the conditions that the grey level $X_{d,k}$ of voxel k is equal to i and the voxel has a valid neighbourhood are both true; it is 0 otherwise. N_v is the number of voxels in the ROI intensity mask.

A 2D example is shown in Table 3.156. A distance of $\delta = 1$ is used in this example, leading to 8 neighbouring pixels. Entry $s_1 = 0$ because there are no valid pixels with grey level 1. Two pixels have grey level 2. The average value of their neighbours are 19/8 and 21/8. Thus $s_2 = |2 - 19/8| + |2 - 21/8| = 1$. Similarly $s_3 = |3 - 19/8| = 0.625$ and $s_4 = |4 - 17/8| = 1.825$.

We deviate from the original definition by Amadasun and King [5] as we do not demand that valid neighbourhoods are completely inside the ROI. In an irregular ROI mask, valid neighbourhoods may simply not exist for a distance δ. Instead, we consider a valid neighbourhood to exist if there is at least one neighbouring voxel included in the ROI mask. The average grey level for voxel k within a valid neighbourhood is then:

$$\overline{X}_k = \frac{1}{W_k} \sum_{m_z=-\delta}^{\delta} \sum_{m_y=-\delta}^{\delta} \sum_{m_x=-\delta}^{\delta} X_d(\mathbf{k}+\mathbf{m}) \left[\mathbf{m} \neq \mathbf{0} \text{ and } \mathbf{k}+\mathbf{m} \text{ in ROI}\right]$$

The neighbourhood size W_k for this voxel is equal to the number of voxels in the neighbourhood that are part of the ROI mask:

$$W_k = \sum_{m_z=-\delta}^{\delta} \sum_{m_y=-\delta}^{\delta} \sum_{m_x=-\delta}^{\delta} \left[\mathbf{m} \neq \mathbf{0} \text{ and } \mathbf{k}+\mathbf{m} \text{ in ROI}\right]$$

Under our definition, neighbourhood grey tone difference s_i for discretised grey level i can be directly expressed using neighbourhood size W_k of voxel k:

$$s_i = \sum_{k}^{N_v} |i - \overline{X}_k| \left[X_d(\mathbf{k}) = i \text{ and } W_k \neq 0\right]$$

Consequentially, n_i is the total number of voxels with grey level i which have a non-zero

1	2	2	3
1	2	3	3
4	2	4	1
4	1	2	3

(a) Grey levels

		n_i	p_i	s_i
i	1	0	0.00	0.000
	2	2	0.50	1.000
	3	1	0.25	0.625
	4	1	0.25	1.875

(b) Neighbourhood grey tone difference matrix

Table 3.156 | Original image with grey levels (a) and corresponding neighbourhood grey tone difference matrix (NGTDM) (b). The $N_{v,c}$ pixels with valid neighbours at distance 1 are located within the rectangle in (a). The grey level voxel count n_i, the grey level probability $p_i = n_i/N_{v,c}$, and the neighbourhood grey level difference s_i for pixels with grey level i are included in the NGTDM. Note that our actual definition deviates from the original definition of Amadasun and King [5], which is used here. In our definition complete neighbourhood are no longer required. In our definition the NGTDM would be calculated on the entire pixel area, and not solely on those pixels within the rectangle of panel (a).

neighbourhood size.

Many NGTDM-based features depend on the N_g grey level probabilities $p_i = n_i/N_{v,c}$, where N_g is the number of discretised grey levels in the ROI intensity mask and $N_{v,c} = \sum n_i$ is total number of voxels that have at least one neighbour. If all voxels have at least one neighbour $N_{v,c} = N_v$. Furthermore, let $N_{g,p} \leq N_g$ be the number of discretised grey levels with $p_i > 0$. In the above example, $N_g = 4$ and $N_{g,p} = 3$.

Aggregating features

Three methods can be used to aggregate NGTDMs and arrive at a single feature value. A schematic example was previously shown in Figure 3.4. A feature may be aggregated as follows:

1. Features are computed from 2D matrices and averaged over slices (8QNN).
2. The feature is computed from a single matrix after merging all 2D matrices (62GR).
3. The feature is computed from a 3D matrix (KOBO).

Method 2 involves merging NGTDMs by summing the neighbourhood grey tone difference s_i and the number of voxels with a valid neighbourhood n_i and grey level i for NGTDMs of the different slices. Note that when NGTDMs are merged, $N_{v,c}$ and p_i should be updated based on the merged NGTDM. Feature values may dependent strongly on the aggregation method.

Distances and distance weighting

The default neighbourhood is defined using the Chebyshev norm. Manhattan or Euclidean norms may be used as well. This requires a more general definition for the average grey level $\overline{X}_k$:

$$\overline{X}_k = \frac{1}{W_k} \sum_{m_z=-\delta}^{\delta} \sum_{m_y=-\delta}^{\delta} \sum_{m_x=-\delta}^{\delta} X_d(\mathbf{k}+\mathbf{m})\,[\|\mathbf{m}\| \leq \delta \text{ and } \mathbf{m} \neq \mathbf{0} \text{ and } \mathbf{k}+\mathbf{m} \text{ in ROI}]$$

The neighbourhood size W_k is:

$$W_k = \sum_{m_z=-\delta}^{\delta} \sum_{m_y=-\delta}^{\delta} \sum_{m_x=-\delta}^{\delta} [\|\mathbf{m}\| \leq \delta \text{ and } \mathbf{m} \neq \mathbf{0} \text{ and } \mathbf{k}+\mathbf{m} \text{ in ROI}]$$

As before, $[\dots]$ is an Iverson bracket.

Distance weighting for NGTDM is relatively straightforward. Let w be a weight dependent on $\mathbf{m}$, e.g. $w = \|\mathbf{m}\|^{-1}$ or $w = \exp(-\|\mathbf{m}\|^2)$. The average grey level is then:

$$\overline{X}_k = \frac{1}{W_k} \sum_{m_z=-\delta}^{\delta} \sum_{m_y=-\delta}^{\delta} \sum_{m_x=-\delta}^{\delta} w(\mathbf{m}) X_d(\mathbf{k}+\mathbf{m}) [\|\mathbf{m}\| \leq \delta \text{ and } \mathbf{m} \neq \mathbf{0} \text{ and } \mathbf{k}+\mathbf{m} \text{ in ROI}]$$

The neighbourhood size W_k becomes a general weight:

$$W_k = \sum_{m_z=-\delta}^{\delta} \sum_{m_y=-\delta}^{\delta} \sum_{m_x=-\delta}^{\delta} w(\mathbf{m}) [\|\mathbf{m}\| \leq \delta \text{ and } \mathbf{m} \neq \mathbf{0} \text{ and } \mathbf{k}+\mathbf{m} \text{ in ROI}]$$

Employing different distance norms and distance weighting is considered non-standard use, and we caution against them due to potential reproducibility issues.

3.10.1 Coarseness QCDE

Grey level differences in coarse textures are generally small due to large-scale patterns. Summing differences gives an indication of the level of the spatial rate of change in intensity[5]. *Coarseness* is defined as:

$$F_{ngt.coarseness} = \frac{1}{\sum_{i=1}^{N_g} p_i\, s_i}$$

Because $\sum_{i=1}^{N_g} p_i\, s_i$ potentially evaluates to 0, the maximum *coarseness* value is set to an arbitrary number of 10^6. Amadasun and King originally circumvented this issue by adding a unspecified small number ϵ to the denominator, but an explicit, though arbitrary, maximum value should allow for more consistency.

data	aggr. method	value	tol.	consensus
dig. phantom	2D	0.121	–	strong
dig. phantom	2.5D	0.0285	–	strong
dig. phantom	3D	0.0296	–	very strong
config. A	2D	0.00629	0.00046	strong
config. A	2.5D	9.06×10^{-5}	3.3×10^{-6}	strong
config. B	2D	0.0168	0.0005	strong
config. B	2.5D	0.000314	4×10^{-6}	strong
config. C	3D	0.000216	4×10^{-6}	strong
config. D	3D	0.000208	4×10^{-6}	strong
config. E	3D	0.000188	4×10^{-6}	strong

Table 3.157 | Reference values for the *coarseness* feature.

3.10.2 Contrast 65HE

Contrast depends on the dynamic range of the grey levels as well as the spatial frequency of intensity changes[5]. Thus, *contrast* is defined as:

$$F_{ngt.contrast} = \left(\frac{1}{N_{g,p}\left(N_{g,p}-1\right)} \sum_{i_1=1}^{N_g} \sum_{i_2=1}^{N_g} p_{i_1} p_{i_2} \left(i_1 - i_2\right)^2 \right) \left(\frac{1}{N_{v,c}} \sum_{i=1}^{N_g} s_i \right)$$

Grey level probabilities p_{i_1} and p_{i_2} are copies of p_i with different iterators, i.e. $p_{i_1} = p_{i_2}$ for $i_1 = i_2$. The first term considers the grey level dynamic range, whereas the second term is a measure for intensity changes within the volume. If $N_{g,p} = 1$, $F_{ngt.contrast} = 0$.

data	aggr. method	value	tol.	consensus
dig. phantom	2D	0.925	–	strong
dig. phantom	2.5D	0.601	–	strong
dig. phantom	3D	0.584	–	very strong
config. A	2D	0.107	0.002	strong
config. A	2.5D	0.0345	0.0009	strong
config. B	2D	0.181	0.001	strong
config. B	2.5D	0.0506	0.0005	strong
config. C	3D	0.0873	0.0019	strong
config. D	3D	0.046	0.0005	strong
config. E	3D	0.0752	0.0019	moderate

Table 3.158 | Reference values for the *contrast* feature.

3.10.3 Busyness NQ30

Textures with large changes in grey levels between neighbouring voxels are said to be busy[5]. *Busyness* was defined as:

$$F_{ngt.busyness} = \frac{\sum_{i=1}^{N_g} p_i\, s_i}{\sum_{i_1=1}^{N_g} \sum_{i_2=1}^{N_g} i_1 p_{i_1} - i_2 p_{i_2}}, \qquad p_{i_1} \neq 0 \text{ and } p_{i_2} \neq 0$$

As before, $p_{i_1} = p_{i_2}$ for $i_1 = i_2$. The original definition was erroneously formulated as the denominator will always evaluate to 0. Therefore we use a slightly different definition[38]:

$$F_{ngt.busyness} = \frac{\sum_{i=1}^{N_g} p_i\, s_i}{\sum_{i_1=1}^{N_g} \sum_{i_2=1}^{N_g} \left| i_1 p_{i_1} - i_2 p_{i_2} \right|}, \qquad p_{i_1} \neq 0 \text{ and } p_{i_2} \neq 0$$

If $N_{g,p} = 1$, $F_{ngt.busyness} = 0$.

data	aggr. method	value	tol.	consensus
dig. phantom	2D	2.99	–	strong
dig. phantom	2.5D	6.8	–	strong
dig. phantom	3D	6.54	–	very strong

config. A	2D	0.489	0.001	strong
config. A	2.5D	8.84	0.01	strong
config. B	2D	0.2	0.005	strong
config. B	2.5D	3.45	0.07	strong
config. C	3D	1.39	0.01	very strong
config. D	3D	5.14	0.14	strong
config. E	3D	4.65	0.1	strong

Table 3.159 | Reference values for the *busyness* feature.

3.10.4 Complexity HDEZ

Complex textures are non-uniform and rapid changes in grey levels are common[5]. Texture *complexity* is defined as:

$$F_{ntg.complexity} = \frac{1}{N_{v,c}} \sum_{i_1=1}^{N_g} \sum_{i_2=1}^{N_g} |i_1 - i_2| \frac{p_{i_1} s_{i_1} + p_{i_2} s_{i_2}}{p_{i_1} + p_{i_2}}, \qquad p_{i_1} \neq 0 \text{ and } p_{i_2} \neq 0$$

As before, $p_{i_1} = p_{i_2}$ for $i_1 = i_2$, and likewise $s_{i_1} = s_{i_2}$ for $i_1 = i_2$.

data	aggr. method	value	tol.	consensus
dig. phantom	2D	10.4	–	strong
dig. phantom	2.5D	14.1	–	strong
dig. phantom	3D	13.5	–	very strong
config. A	2D	438	9	strong
config. A	2.5D	580	19	strong
config. B	2D	391	7	strong
config. B	2.5D	496	5	strong
config. C	3D	1.81×10^3	60	very strong
config. D	3D	400	5	strong
config. E	3D	574	1	moderate

Table 3.160 | Reference values for the *complexity* feature.

3.10.5 Strength

1X9X

Amadasun and King[5] defined texture *strength* as:

$$F_{ngt.strength} = \frac{\sum_{i_1=1}^{N_g} \sum_{i_2=1}^{N_g} \left(p_{i_1} + p_{i_2}\right) \left(i_1 - i_2\right)^2}{\sum_{i=1}^{N_g} s_i}, \qquad p_{i_1} \neq 0 \text{ and } p_{i_2} \neq 0$$

As before, $p_{i_1} = p_{i_2}$ for $i_1 = i_2$. If $\sum_{i=1}^{N_g} s_i = 0$, $F_{ngt.strength} = 0$.

data	aggr. method	value	tol.	consensus
dig. phantom	2D	2.88	–	strong
dig. phantom	2.5D	0.741	–	strong
dig. phantom	3D	0.763	–	very strong
config. A	2D	3.33	0.08	strong
config. A	2.5D	0.0904	0.0027	strong
config. B	2D	6.02	0.23	strong
config. B	2.5D	0.199	0.009	strong
config. C	3D	0.651	0.015	strong
config. D	3D	0.162	0.008	strong
config. E	3D	0.167	0.006	strong

Table 3.161 | Reference values for the *strength* feature.

3.11 Neighbouring grey level dependence based features REK0

Sun and Wee [69] defined the neighbouring grey level dependence matrix (NGLDM) as an alternative to the grey level co-occurrence matrix. The NGLDM aims to capture the coarseness of the overall texture and is rotationally invariant.

NGLDM also involves the concept of a neighbourhood around a central voxel. All voxels within Chebyshev distance δ are considered to belong to the neighbourhood of the center voxel. The discretised grey levels of the center voxel k at position $\mathbf{k}$ and a neighbouring voxel m at $\mathbf{k}+\mathbf{m}$ are said to be dependent if $|X_d(\mathbf{k}) - X_d(\mathbf{k}+\mathbf{m})| \leq \alpha$, with α being a non-negative integer coarseness parameter. The number of grey level dependent voxels j within the neighbourhood is then counted as:

$$j_k = 1 + \sum_{m_z=-\delta}^{\delta} \sum_{m_y=-\delta}^{\delta} \sum_{m_x=-\delta}^{\delta} [|X_d(\mathbf{k}) - X_d(\mathbf{k}+\mathbf{m})| \leq \alpha \text{ and } \mathbf{m} \neq \mathbf{0}]$$

Here, $[\dots]$ is an Iverson bracket, which is 1 if the aforementioned condition is fulfilled, and 0 otherwise. Note that the minimum dependence $j_k = 1$ and not $j_k = 0$. This is done because some feature definitions require a minimum dependence of 1 or are undefined otherwise. One may therefore also simplify the expression for j_k by including the center voxel:

$$j_k = \sum_{m_z=-\delta}^{\delta} \sum_{m_y=-\delta}^{\delta} \sum_{m_x=-\delta}^{\delta} [|X_d(\mathbf{k}) - X_d(\mathbf{k}+\mathbf{m})| \leq \alpha]$$

Dependence j_k is iteratively determined for each voxel k in the ROI intensity mask. $\mathbf{M}$ is then the $N_g \times N_n$ neighbouring grey level dependence matrix, where N_g is the number of discretised grey levels present in the ROI intensity mask and $N_n = \max(j_k)$ the maximum grey level dependence count found. Element s_{ij} of $\mathbf{M}$ is then the number of neighbourhoods with a center voxel with discretised grey level i and a neighbouring voxel dependence j. Furthermore, let N_v be the number of voxels in the ROI intensity mask, and $N_s = \sum_{i=1}^{N_g}\sum_{j=1}^{N_n} s_{ij}$ the number of neighbourhoods. Marginal sums can likewise be defined. Let $s_{i.} = \sum_{j=1}^{N_n}$ be the number of neighbourhoods with discretised grey level i, and let $s_{j.} = \sum_{i=1}^{N_g} s_{ij}$ be the number of neighbourhoods with dependence j, regardless of grey level. A two dimensional example is shown in Table 3.162.

The definition we actually use deviates from the original by Sun and Wee [69]. Because regions of interest are rarely cuboid, omission of neighbourhoods which contain voxels outside the ROI mask may lead to inconsistent results, especially for larger distance δ. Hence the neighbourhoods of all voxels in the within the ROI intensity mask are considered, and consequently $N_v = N_s$. Neighbourhood voxels located outside the ROI do not add to dependence j:

$$j_k = \sum_{m_z=-\delta}^{\delta} \sum_{m_y=-\delta}^{\delta} \sum_{m_x=-\delta}^{\delta} [|X_d(\mathbf{k}) - X_d(\mathbf{k}+\mathbf{m})| \leq \alpha \text{ and } \mathbf{k}+\mathbf{m} \text{ in ROI}]$$

Note that while $\alpha = 0$ is a typical choice for the coarseness parameter, different α are possible. Likewise, a typical choice for neighbourhood radius δ is Chebyshev distance $\delta = 1$ but larger values are possible as well.

1	2	2	3
1	2	3	3
4	2	4	1
4	1	2	3

(a) Grey levels

		dependence k			
		0	1	2	3
i	1	0	0	0	0
	2	0	0	1	1
	3	0	0	1	0
	4	1	0	0	0

(b) Neighbouring grey level dependence matrix

Table 3.162 | Original image with grey levels and pixels with a complete neighbourhood within the square (a); corresponding neighbouring grey level dependence matrix for distance $d = \sqrt{2}$ and coarseness parameter $a = 0$ (b). Element $s(i,j)$ of the NGLDM indicates the number of neighbourhoods with a center pixel with grey level i and neighbouring grey level dependence k within the image. Note that in our definition a complete neighbourhood is no longer required. Thus every voxel is considered as a center voxel with a neighbourhood, instead of being constrained to the voxels within the square in panel (a).

Aggregating features

Three methods can be used to aggregate NGLDMs and arrive at a single feature value. A schematic example was previously shown in Figure 3.4. A feature may be aggregated as follows:

1. Features are computed from 2D matrices and averaged over slices (8QNN).
2. The feature is computed from a single matrix after merging all 2D matrices (62GR).
3. The feature is computed from a 3D matrix (KOBO).

Method 2 involves merging NGLDMs by summing the dependence count s_{ij} by element over the NGLDM of the different slices. Note that when NGLDMs are merged, N_v and N_s should likewise be summed to retain consistency. Feature values may dependent strongly on the aggregation method.

Distances and distance weighting

Default neighbourhoods are constructed using the Chebyshev norm, but other norms can be used as well. For this purpose it is useful to generalise the dependence count equation to:

$$j_k = \sum_{m_z=-\delta}^{\delta} \sum_{m_y=-\delta}^{\delta} \sum_{m_x=-\delta}^{\delta} [\|\mathbf{m}\| \leq \delta \text{ and } |X_d(\mathbf{k}) - X_d(\mathbf{k}+\mathbf{m})| \leq \alpha \text{ and } \mathbf{k}+\mathbf{m} \text{ in ROI}]$$

with $\mathbf{m}$ the vector between voxels k and m and $\|\mathbf{m}\|$ its length according to the particular norm.

In addition, dependence may be weighted by distance. Let w be a weight dependent on $\mathbf{m}$, e.g. $w = \|\mathbf{m}\|^{-1}$ or $w = \exp(-\|\mathbf{m}\|^2)$. The dependence of voxel k is then:

$$j_k = \sum_{m_z=-\delta}^{\delta} \sum_{m_y=-\delta}^{\delta} \sum_{m_x=-\delta}^{\delta} w(\mathbf{m})[\|\mathbf{m}\| \leq \delta \text{ and } |X_d(\mathbf{k}) - X_d(\mathbf{k}+\mathbf{m})| \leq \alpha \text{ and } \mathbf{k}+\mathbf{m} \text{ in ROI}]$$

Employing different distance norms and distance weighting is considered non-standard use, and we caution against them due to potential reproducibility issues.

Note on feature references

The NGLDM is structured similarly to the GLRLM, GLSZM and GLDZM. NGLDM feature definitions are therefore based on the definitions of GLRLM features, and references may be found in Section 3.7, except for the features originally defined by Sun and Wee [69].

3.11.1 Low dependence emphasis SODN

This feature emphasises low neighbouring grey level dependence counts. Sun and Wee [69] refer to this feature as *small number emphasis*. It is defined as:

$$F_{ngl.lde} = \frac{1}{N_s}\sum_{j=1}^{N_n}\frac{s_{.j}}{j^2}$$

data	aggr. method	value	tol.	consensus
dig. phantom	2D	0.158	–	strong
dig. phantom	2.5D	0.159	–	strong
dig. phantom	3D	0.045	–	very strong
config. A	2D	0.281	0.003	strong
config. A	2.5D	0.243	0.004	strong
config. B	2D	0.31	0.001	strong
config. B	2.5D	0.254	0.002	strong
config. C	3D	0.137	0.003	very strong
config. D	3D	0.0912	0.0007	strong
config. E	3D	0.118	0.001	strong

Table 3.163 | Reference values for the *low dependence emphasis* feature.

3.11.2 High dependence emphasis IMOQ

This feature emphasises high neighbouring grey level dependence counts. Sun and Wee [69] refer to this feature as *large number emphasis*. It is defined as:

$$F_{ngl.hde} = \frac{1}{N_s}\sum_{j=1}^{N_n} j^2 s_{.j}$$

data	aggr. method	value	tol.	consensus
dig. phantom	2D	19.2	–	strong
dig. phantom	2.5D	18.8	–	strong
dig. phantom	3D	109	–	very strong
config. A	2D	14.8	0.1	strong
config. A	2.5D	16.1	0.2	strong

config. B	2D	17.3	0.2	strong
config. B	2.5D	19.6	0.2	strong
config. C	3D	126	2	strong
config. D	3D	223	5	strong
config. E	3D	134	3	strong

Table 3.164 | Reference values for the *high dependence emphasis* feature.

3.11.3 Low grey level count emphasis TL9H

This feature is a grey level analogue to *low dependence emphasis*. Instead of low neighbouring grey level dependence counts, low grey levels are emphasised. The feature is defined as:

$$F_{ngl.lgce} = \frac{1}{N_s} \sum_{i=1}^{N_g} \frac{s_{i.}}{i^2}$$

data	aggr. method	value	tol.	consensus
dig. phantom	2D	0.702	—	strong
dig. phantom	2.5D	0.693	—	strong
dig. phantom	3D	0.693	—	very strong
config. A	2D	0.0233	0.0003	strong
config. A	2.5D	0.0115	0.0003	strong
config. B	2D	0.0286	0.0004	strong
config. B	2.5D	0.0139	0.0005	strong
config. C	3D	0.0013	4×10^{-5}	strong
config. D	3D	0.0168	0.0009	strong
config. E	3D	0.0154	0.0007	strong

Table 3.165 | Reference values for the *low grey level count emphasis* feature.

3.11.4 High grey level count emphasis OAE7

The *high grey level count emphasis* feature is a grey level analogue to *high dependence emphasis*. The feature emphasises high grey levels, and is defined as:

$$F_{ngl.hgce} = \frac{1}{N_s} \sum_{i=1}^{N_g} i^2 s_{i.}$$

data	aggr. method	value	tol.	consensus
dig. phantom	2D	7.49	—	strong
dig. phantom	2.5D	7.66	—	strong
dig. phantom	3D	7.66	—	very strong
config. A	2D	446	2	strong

config. A	2.5D	466	2	strong
config. B	2D	359	10	strong
config. B	2.5D	375	11	strong
config. C	3D	1.57×10^{3}	10	strong
config. D	3D	364	16	strong
config. E	3D	502	8	strong

Table 3.166 | Reference values for the *high grey level count emphasis* feature.

3.11.5 Low dependence low grey level emphasis EQ3F

This feature emphasises neighbouring grey level dependence counts in the upper left quadrant of the NGLDM, where low dependence counts and low grey levels are located. It is defined as:

$$F_{ngl.ldlge} = \frac{1}{N_s} \sum_{i=1}^{N_g} \sum_{j=1}^{N_n} \frac{s_{ij}}{i^2 j^2}$$

data	aggr. method	value	tol.	consensus
dig. phantom	2D	0.0473	–	strong
dig. phantom	2.5D	0.0477	–	strong
dig. phantom	3D	0.00963	–	very strong
config. A	2D	0.0137	0.0002	strong
config. A	2.5D	0.00664	0.0002	strong
config. B	2D	0.0203	0.0003	strong
config. B	2.5D	0.00929	0.00026	strong
config. C	3D	0.000306	1.2×10^{-5}	strong
config. D	3D	0.00357	4×10^{-5}	strong
config. E	3D	0.00388	4×10^{-5}	strong

Table 3.167 | Reference values for the *low dependence low grey level emphasis* feature.

3.11.6 Low dependence high grey level emphasis JA6D

This feature emphasises neighbouring grey level dependence counts in the lower left quadrant of the NGLDM, where low dependence counts and high grey levels are located. The feature is defined as:

$$F_{ngl.ldhge} = \frac{1}{N_s} \sum_{i=1}^{N_g} \sum_{j=1}^{N_n} \frac{i^2 s_{ij}}{j^2}$$

data	aggr. method	value	tol.	consensus
dig. phantom	2D	3.06	—	strong
dig. phantom	2.5D	3.07	—	strong
dig. phantom	3D	0.736	—	very strong
config. A	2D	94.2	0.4	strong
config. A	2.5D	91.9	0.5	strong
config. B	2D	78.9	2.2	strong
config. B	2.5D	73.4	2.1	strong
config. C	3D	141	2	strong
config. D	3D	18.9	1.1	strong
config. E	3D	36.7	0.5	strong

Table 3.168 | Reference values for the *low dependence high grey level emphasis* feature.

3.11.7 High dependence low grey level emphasis NBZI

This feature emphasises neighbouring grey level dependence counts in the upper right quadrant of the NGLDM, where high dependence counts and low grey levels are located. The feature is defined as:

$$F_{ngl.hdlge} = \frac{1}{N_s} \sum_{i=1}^{N_g} \sum_{j=1}^{N_n} \frac{j^2 s_{ij}}{i^2}$$

data	aggr. method	value	tol.	consensus
dig. phantom	2D	17.6	—	strong
dig. phantom	2.5D	17.2	—	strong
dig. phantom	3D	102	—	very strong
config. A	2D	0.116	0.001	strong
config. A	2.5D	0.0674	0.0004	strong
config. B	2D	0.108	0.003	strong
config. B	2.5D	0.077	0.0019	strong
config. C	3D	0.0828	0.0003	strong
config. D	3D	0.798	0.072	strong
config. E	3D	0.457	0.031	strong

Table 3.169 | Reference values for the *high dependence low grey level emphasis* feature.

3.11.8 High dependence high grey level emphasis 9QMG

The *high dependence high grey level emphasis* feature emphasises neighbouring grey level dependence counts in the lower right quadrant of the NGLDM, where high dependence counts and high grey levels are located. The feature is defined as:

$$F_{ngl.hdhge} = \frac{1}{N_s}\sum_{i=1}^{N_g}\sum_{j=1}^{N_n} i^2 j^2 s_{ij}$$

data	aggr. method	value	tol.	consensus
dig. phantom	2D	49.5	–	strong
dig. phantom	2.5D	50.8	–	strong
dig. phantom	3D	235	–	very strong
config. A	2D	7.54×10^3	60	strong
config. A	2.5D	8.1×10^3	60	strong
config. B	2D	7.21×10^3	130	strong
config. B	2.5D	7.97×10^3	150	strong
config. C	3D	2.27×10^5	3×10^3	strong
config. D	3D	9.28×10^4	1.3×10^3	strong
config. E	3D	7.6×10^4	600	strong

Table 3.170 | Reference values for the *high dependence high grey level emphasis* feature.

3.11.9 Grey level non-uniformity FP8K

Grey level non-uniformity assesses the distribution of neighbouring grey level dependence counts over the grey values. The feature value is low when dependence counts are equally distributed along grey levels. The feature is defined as:

$$F_{ngl.glnu} = \frac{1}{N_s}\sum_{i=1}^{N_g} s_{i.}^2$$

data	aggr. method	value	tol.	consensus
dig. phantom	2D	10.2	–	strong
dig. phantom	2.5D	37.9	–	strong
dig. phantom	3D	37.9	–	very strong
config. A	2D	757	1	strong
config. A	2.5D	1.72×10^4	100	strong
config. B	2D	216	3	strong
config. B	2.5D	4.76×10^3	50	strong
config. C	3D	6.42×10^3	10	strong
config. D	3D	1.02×10^4	300	strong
config. E	3D	8.17×10^3	130	strong

Table 3.171 | Reference values for the *grey level non-uniformity* feature.

3.11.10 Normalised grey level non-uniformity 5SPA

This is a normalised version of the *grey level non-uniformity* feature. It is defined as:

$$F_{ngl.glnu.norm} = \frac{1}{N_s^2} \sum_{i=1}^{N_g} s_{i.}^2$$

The *normalised grey level non-uniformity* computed from a single 3D NGLDM matrix is equivalent to the *intensity histogram uniformity* feature[81].

data	aggr. method	value	tol.	consensus
dig. phantom	2D	0.562	–	strong
dig. phantom	2.5D	0.512	–	strong
dig. phantom	3D	0.512	–	very strong
config. A	2D	0.151	0.003	strong
config. A	2.5D	0.15	0.002	strong
config. B	2D	0.184	0.001	strong
config. B	2.5D	0.174	0.001	strong
config. C	3D	0.14	0.003	very strong
config. D	3D	0.229	0.003	strong
config. E	3D	0.184	0.001	strong

Table 3.172 | Reference values for the *normalised grey level non-uniformity* feature.

3.11.11 Dependence count non-uniformity Z87G

This features assesses the distribution of neighbouring grey level dependence counts over the different dependence counts. The feature value is low when dependence counts are equally distributed. Sun and Wee[69] refer to this feature as *number non-uniformity*. It is defined as:

$$F_{ngl.dcnu} = \frac{1}{N_s} \sum_{j=1}^{N_n} s_{.j}^2$$

data	aggr. method	value	tol.	consensus
dig. phantom	2D	3.96	–	strong
dig. phantom	2.5D	12.4	–	strong
dig. phantom	3D	4.86	–	very strong
config. A	2D	709	2	strong
config. A	2.5D	1.75×10^4	100	strong
config. B	2D	157	1	strong
config. B	2.5D	3.71×10^3	30	strong
config. C	3D	2.45×10^3	60	strong
config. D	3D	1.84×10^3	30	strong
config. E	3D	2.25×10^3	30	strong

Table 3.173 | Reference values for the *dependence count non-uniformity* feature.

3.11.12 Normalised dependence count non-uniformity 0KJI

This is a normalised version of the *dependence count non-uniformity* feature. It is defined as:

$$F_{ngl.dcnu.norm} = \frac{1}{N_s^2} \sum_{i=1}^{N_n} s_{.j}^2$$

data	aggr. method	value	tol.	consensus
dig. phantom	2D	0.212	–	strong
dig. phantom	2.5D	0.167	–	strong
dig. phantom	3D	0.0657	–	very strong
config. A	2D	0.175	0.001	strong
config. A	2.5D	0.153	0.001	strong
config. B	2D	0.179	0.001	strong
config. B	2.5D	0.136	0.001	strong
config. C	3D	0.0532	0.0005	strong
config. D	3D	0.0413	0.0003	strong
config. E	3D	0.0505	0.0003	strong

Table 3.174 | Reference values for the *normalised dependence count non-uniformity* feature.

3.11.13 Dependence count percentage 6XV8

This feature measures the fraction of the number of realised neighbourhoods and the maximum number of potential neighbourhoods. *Dependence count percentage* may be completely omitted as it evaluates to 1 when complete neighbourhoods are not required, as is the case under our definition. It is defined as:

$$F_{ngl.dc.perc} = \frac{N_s}{N_v}$$

data	aggr. method	value	tol.	consensus
dig. phantom	2D	1	–	strong
dig. phantom	2.5D	1	–	moderate
dig. phantom	3D	1	–	strong
config. A	2D	1	–	moderate
config. A	2.5D	1	–	strong
config. B	2D	1	–	moderate
config. B	2.5D	1	–	moderate
config. C	3D	1	–	strong
config. D	3D	1	–	strong
config. E	3D	1	–	moderate

Table 3.175 | Reference values for the *dependence count percentage* feature.

3.11.14 Grey level variance 1PFV

This feature estimates the variance in dependence counts over the grey levels. Let $p_{ij} = s_{ij}/N_s$ be the joint probability estimate for finding discretised grey level i with dependence j. The feature is then defined as:

$$F_{ngl.gl.var} = \sum_{i=1}^{N_g} \sum_{j=1}^{N_n} (i - \mu)^2 p_{ij}$$

Here, $\mu = \sum_{i=1}^{N_g} \sum_{j=1}^{N_n} i\, p_{ij}$.

data	aggr. method	value	tol.	consensus
dig. phantom	2D	2.7	–	strong
dig. phantom	2.5D	3.05	–	strong
dig. phantom	3D	3.05	–	very strong
config. A	2D	31.1	0.5	strong
config. A	2.5D	22.8	0.6	strong
config. B	2D	25.3	0.4	strong
config. B	2.5D	18.7	0.2	strong
config. C	3D	81.1	2.1	very strong
config. D	3D	21.7	0.4	strong
config. E	3D	30.4	0.8	strong

Table 3.176 | Reference values for the *grey level variance* feature.

3.11.15 Dependence count variance DNX2

This feature estimates the variance in dependence counts over the different possible dependence counts. As before let $p_{ij} = s_{ij}/N_s$. The feature is defined as:

$$F_{ngl.dc.var} = \sum_{i=1}^{N_g} \sum_{j=1}^{N_n} (j - \mu)^2 p_{ij}$$

Mean dependence count is defined as $\mu = \sum_{i=1}^{N_g} \sum_{j=1}^{N_n} j\, p_{ij}$.

data	aggr. method	value	tol.	consensus
dig. phantom	2D	2.73	–	strong
dig. phantom	2.5D	3.27	–	strong
dig. phantom	3D	22.1	–	very strong
config. A	2D	3.12	0.02	strong
config. A	2.5D	3.37	0.01	strong
config. B	2D	4.02	0.05	strong
config. B	2.5D	4.63	0.06	strong
config. C	3D	39.2	0.1	strong

config. D	3D	63.9	1.3	strong
config. E	3D	39.4	1	strong

Table 3.177 | Reference values for the *dependence count variance* feature.

3.11.16 Dependence count entropy FCBV

This feature is referred to as *entropy* by Sun and Wee [69]. Let $p_{ij} = s_{ij}/N_s$. *Dependence count entropy* is then defined as:

$$F_{ngl.dc.entr} = -\sum_{i=1}^{N_g}\sum_{j=1}^{N_n} p_{ij}\log_2 p_{ij}$$

This definition remedies an error in the definition of Sun and Wee [69], where the term within the logarithm is dependence count s_{ij} instead of count probability p_{ij}.

data	aggr. method	value	tol.	consensus
dig. phantom	2D	2.71	–	strong
dig. phantom	2.5D	3.36	–	strong
dig. phantom	3D	4.4	–	very strong
config. A	2D	5.76	0.02	strong
config. A	2.5D	5.93	0.02	strong
config. B	2D	5.38	0.01	strong
config. B	2.5D	5.78	0.01	strong
config. C	3D	7.54	0.03	very strong
config. D	3D	6.98	0.01	strong
config. E	3D	7.06	0.02	strong

Table 3.178 | Reference values for the *dependence count entropy* feature.

3.11.17 Dependence count energy CAS9

This feature is called *second moment* by Sun and Wee [69]. Let $p_{ij} = s_{ij}/N_s$. Then *dependence count energy* is defined as:

$$F_{ngl.dc.energy} = \sum_{i=1}^{N_g}\sum_{j=1}^{N_n} p_{ij}^2$$

This definition also remedies an error in the original definition, where squared dependence count s_{ij}^2 is divided by N_s only, thus leaving a major volume dependency. In the definition given here, s_{ij}^2 is normalised by N_s^2 through the use of count probability p_{ij}.

data	aggr. method	value	tol.	consensus
dig. phantom	2D	0.17	–	strong
dig. phantom	2.5D	0.122	–	strong
dig. phantom	3D	0.0533	–	very strong

config. A	2D	0.0268	0.0004	strong
config. A	2.5D	0.0245	0.0003	strong
config. B	2D	0.0321	0.0002	strong
config. B	2.5D	0.0253	0.0001	moderate
config. C	3D	0.00789	0.00011	strong
config. D	3D	0.0113	0.0002	strong
config. E	3D	0.0106	0.0001	strong

Table 3.179 | Reference values for the *dependence count energy* feature.

Chapter 4

Radiomics reporting guidelines and nomenclature

Reliable and complete reporting is necessary to ensure reproducibility and validation of results. To help provide a complete report on image processing and image biomarker extraction, we present the guidelines below, as well as a nomenclature system to uniquely features.

4.1 Reporting guidelines

These guidelines are partially based on the work of Lambin et al. [41], Sanduleanu et al. [57], Sollini et al. [64], Traverso et al. [73]. Additionally, guidelines are derived from the image processing and feature calculation steps described within this document. An earlier version was reported elsewhere [79].

**topic**		**item**	**description**
**Patient**			
Region of interest[1]		1	Describe the region of interest that is being imaged.
Patient preparation		2a	Describe specific instructions given to patients prior to image acquisition, e.g. fasting prior to imaging.
		2b	Describe administration of drugs to the patient prior to image acquisition, e.g. muscle relaxants.
		2c	Describe the use of specific equipment for patient comfort during scanning, e.g. ear plugs.
Radioactive tracer	PET, SPECT	3a	Describe which radioactive tracer was administered to the patient, e.g. 18F-FDG.
	PET, SPECT	3b	Describe the administration method.
	PET, SPECT	3c	Describe the injected activity of the radioactive tracer at administration.

continued on next page

[1]Also referred to as volume of interest.

topic		item	description
	PET, SPECT	3d	Describe the uptake time prior to image acquisition.
	PET, SPECT	3e	Describe how competing substance levels were controlled.[2]
Contrast agent		4a	Describe which contrast agent was administered to the patient.
		4b	Describe the administration method.
		4c	Describe the injected quantity of contrast agent.
		4d	Describe the uptake time prior to image acquisition.
		4e	Describe how competing substance levels were controlled.
Comorbidities		5	Describe if the patients have comorbidities that affect imaging.[3]
**Acquisition**[4]			
Acquisition protocol		6	Describe whether a standard imaging protocol was used, and where its description may be found.
Scanner type		7	Describe the scanner type(s) and vendor(s) used in the study.
Imaging modality		8	Clearly state the imaging modality that was used in the study, e.g. CT, MRI.
Static/dynamic scans		9a	State if the scans were static or dynamic.
	Dynamic scans	9b	Describe the acquisition time per time frame.
	Dynamic scans	9c	Describe any temporal modelling technique that was used.
Scanner calibration		10	Describe how and when the scanner was calibrated.
Patient instructions		11	Describe specific instructions given to the patient during acquisition, e.g. breath holding.
Anatomical motion correction		12	Describe the method used to minimise the effect of anatomical motion.
Scan duration		13	Describe the duration of the complete scan or the time per bed position.
Tube voltage	CT	14	Describe the peak kilo voltage output of the X-ray source.
Tube current	CT	15	Describe the tube current in mA.
Time-of-flight	PET	16	State if scanner time-of-flight capabilities are used during acquisition.
RF coil	MRI	17	Describe what kind RF coil used for acquisition, incl. vendor.

continued on next page

[2]An example is glucose present in the blood which competes with the uptake of 18F-FDG tracer in tumour tissue. To reduce competition with the tracer, patients are usually asked to fast for several hours and a blood glucose measurement may be conducted prior to tracer administration.

[3]An example of a comorbidity that may affect image quality in 18F-FDG PET scans are type I and type II diabetes melitus, as well as kidney failure.

[4]Many acquisition parameters may be extracted from DICOM header meta-data, or calculated from them.

topic		item	description
Scanning sequence	MRI	18a	Describe which scanning sequence was acquired.
	MRI	18b	Describe which sequence variant was acquired.
	MRI	18c	Describe which scan options apply to the current sequence, e.g. flow compensation, cardiac gating.
Repetition time	MRI	19	Describe the time in ms between subsequent pulse sequences.
Echo time	MRI	20	Describe the echo time in ms.
Echo train length	MRI	21	Describe the number of lines in k-space that are acquired per excitation pulse.
Inversion time	MRI	22	Describe the time in ms between the middle of the inverting RF pulse to the middle of the excitation pulse.
Flip angle	MRI	23	Describe the flip angle produced by the RF pulses.
Acquisition type	MRI	24	Describe the acquisition type of the MRI scan, e.g. 3D.
k-space traversal	MRI	25	Describe the acquisition trajectory of the k-space.
Number of averages/ excitations	MRI	26	Describe the number of times each point in k-space is sampled.
Magnetic field strength	MRI	27	Describe the nominal strength of the MR magnetic field.
**Reconstruction**[5]			
In-plane resolution		28	Describe the distance between pixels, or alternatively the field of view and matrix size.
Image slice thickness		29	Describe the slice thickness.
Image slice spacing		30	Describe the distance between image slices.[6]
Convolution kernel	CT	31a	Describe the convolution kernel used to reconstruct the image.
	CT	31b	Describe settings pertaining to iterative reconstruction algorithms.
Exposure	CT	31c	Describe the exposure (in mAs) in slices containing the region of interest.
Reconstruction method	PET	32a	Describe which reconstruction method was used, e.g. 3D OSEM.
	PET	32b	Describe the number of iterations for iterative reconstruction.
	PET	32c	Describe the number of subsets for iterative reconstruction.
Point spread function modelling	PET	33	Describe if and how point-spread function modelling was performed.
Image corrections	PET	34a	Describe if and how attenuation correction was performed.

continued on next page

[5]Many reconstruction parameters may be extracted from DICOM header meta-data.
[6]Spacing between image slicing is commonly, but not necessarily, the same as the slice thickness,.

topic		item	description
	PET	34b	Describe if and how other forms of correction were performed, e.g. scatter correction, randoms correction, dead time correction etc.
Reconstruction method	MRI	35a	Describe the reconstruction method used to reconstruct the image from the k-space information.
	MRI	35b	Describe any artifact suppression methods used during reconstruction to suppress artifacts due to undersampling of k-space.
Diffusion-weighted imaging	DWI-MRI	36	Describe the b-values used for diffusion-weighting.
**Image registration**			
Registration method		37	Describe the method used to register multi-modality imaging.
**Image processing - data conversion**			
SUV normalisation	PET	38	Describe which standardised uptake value (SUV) normalisation method is used.
ADC computation	DWI-MRI	39	Describe how apparent diffusion coefficient (ADC) values were calculated.
Other data conversions		40	Describe any other conversions that are performed to generate e.g. perfusion maps.
**Image processing - post-acquisition processing**			
Anti-aliasing		41	Describe the method used to deal with anti-aliasing when down-sampling during interpolation.
Noise suppression		42	Describe methods used to suppress image noise.
Post-reconstruction smoothing filter	PET	43	Describe the width of the Gaussian filter (FWHM) to spatially smooth intensities.
Skull stripping	MRI (brain)	44	Describe method used to perform skull stripping.
Non-uniformity correction[7]	MRI	45	Describe the method and settings used to perform non-uniformity correction.
Intensity normalisation		46	Describe the method and settings used to normalise intensity distributions within a patient or patient cohort.
Other post-acquisition processing methods		47	Describe any other methods that were used to process the image and are not mentioned separately in this list.
**Segmentation**			
Segmentation method		48a	Describe how regions of interest were segmented, e.g. manually.
		48b	Describe the number of experts, their expertise and consensus strategies for manual delineation.

continued on next page

[7]Also known as bias-field correction.

topic		item	description
		48c	Describe methods and settings used for semi-automatic and fully automatic segmentation.
		48d	Describe which image was used to define segmentation in case of multi-modality imaging.
Conversion to mask		49	Describe the method used to convert polygonal or mesh-based segmentations to a voxel-based mask.
**Image processing - image interpolation**			
Interpolation method		50a	Describe which interpolation algorithm was used to interpolate the image.
		50b	Describe how the position of the interpolation grid was defined, e.g. align by center.
		50c	Describe how the dimensions of the interpolation grid were defined, e.g. rounded to nearest integer.
		50d	Describe how extrapolation beyond the original image was handled.
Voxel dimensions		51	Describe the size of the interpolated voxels.
Intensity rounding	CT	52	Describe how fractional Hounsfield Units are rounded to integer values after interpolation.
**Image processing - ROI interpolation**			
Interpolation method		53	Describe which interpolation algorithm was used to interpolate the region of interest mask.
Partially masked voxels		54	Describe how partially masked voxels after interpolation are handled.
**Image processing - re-segmentation**			
Re-segmentation methods		55	Describe which methods and settings are used to re-segment the ROI intensity mask.
**Image processing - discretisation**			
Discretisation method[8]		56a	Describe the method used to discretise image intensities.
		56b	Describe the number of bins (FBN) or the bin size (FBS) used for discretisation.
		56c	Describe the lowest intensity in the first bin for FBS discretisation.[9]
**Image processing - image transformation**			
Image filter[10]		57	Describe the methods and settings used to filter images, e.g. Laplacian-of-Gaussian.
**Image biomarker computation**			

continued on next page

[8]Discretisation may be performed separately to create intensity-volume histograms. If this is indeed the case, this should be described as well.
[9]This is typically set by range re-segmentation.
[10]The IBSI has not introduced image transformation into the standardised image processing scheme, and is in the process of benchmarking various common filters. This section may therefore be expanded in the future.

topic	item	description
Biomarker set	58	Describe which set of image biomarkers is computed and refer to their definitions or provide these.
IBSI compliance	59	State if the software used to extract the set of image biomarkers is able to reproduce the IBSI feature reference values.[11]
Robustness	60	Describe how robustness of the image biomarkers was assessed, e.g. test-retest analysis.
Software availability	61	Describe which software and version was used to compute image biomarkers.
**Image biomarker computation - texture parameters**		
Texture matrix aggregation	62	Define how texture-matrix based biomarkers were computed from underlying texture matrices.
Distance weighting	63	Define how CM, RLM, NGTDM and NGLDM weight distances, e.g. no weighting.
CM symmetry	64	Define whether symmetric or asymmetric co-occurrence matrices were computed.
CM distance	65	Define the (Chebyshev) distance at which co-occurrence of intensities is determined, e.g. 1.
SZM linkage distance	66	Define the distance and distance norm for which voxels with the same intensity are considered to belong to the same zone for the purpose of constructing an SZM, e.g. Chebyshev distance of 1.
DZM linkage distance	67	Define the distance and distance norm for which voxels with the same intensity are considered to belong to the same zone for the purpose of constructing a DZM, e.g. Chebyshev distance of 1.
DZM zone distance norm	68	Define the distance norm for determining the distance of zones to the border of the ROI, e.g. Manhattan distance.
NGTDM distance	69	Define the neighbourhood distance and distance norm for the NGTDM, e.g. Chebyshev distance of 1.
NGLDM distance	70	Define the neighbourhood distance and distance norm for the NGLDM, e.g. Chebyshev distance of 1.
NGLDM coarseness	71	Define the coarseness parameter for the NGLDM, e.g. 0.
**Machine learning and radiomics analysis**		
Diagnostic and prognostic modelling	72	See the TRIPOD guidelines for reporting on diagnostic and prognostic modelling.

continued on next page

[11]A software is compliant if and only if it is able to reproduce image biomarker reference values for the digital phantom and for one or more image processing configurations using the radiomics CT phantom. Reviewers may demand that you provide the IBSI compliance spreadsheet for your software.

topic	item	description
Comparison with known factors	73	Describe where performance of radiomics models is compared with known (clinical) factors.
Multicollinearity	74	Describe where the multicollinearity between image biomarkers in the signature is assessed.
Model availability	75	Describe where radiomics models with the necessary pre-processing information may be found.
Data availability	76	Describe where imaging data and relevant meta-data used in the study may be found.

Table 4.1 | Guidelines for reporting on radiomic studies. Not all items may be applicable.

4.2 Feature nomenclature

Image features may be extracted using a variety of different settings, and may even share the same name. A feature nomenclature is thus required. Let us take the example of differentiating the following features: *i*) intensity histogram-based entropy, discretised using a *fixed bin size* algorithm with 25 HU bins, extracted from a CT image; and *ii*) grey level run length matrix entropy, discretised using a *fixed bin number* algorithm with 32 bins, extracted from a PET image. To refer to both as *entropy* would be ambiguous, whereas to add a full textual description would be cumbersome. In the nomenclature proposed below, the features would be called $entropy_{IH,\ CT,\ FBS:25HU}$ and $entropy_{RLM,\ PET,\ FBN:32}$, respectively.

Features are thus indicated by a feature name and a subscript. As the nomenclature is designed to both concise and complete, only details for which ambiguity may exist are to be explicitly incorporated in the subscript. The subscript of a feature name may contain the following items to address ambiguous naming:

1. An abbreviation of the feature family (required).
2. The aggregation method of a feature (optional).
3. A descriptor describing the modality the feature is based on, the specific channel (for microscopy images), the specific imaging data (in the case of repeat imaging or delta-features) sets, conversions (such as SUV and SUL), and/or the specific ROI. For example, one could write *PET:SUV* to separate it from *CT* and *PET:SUL* features (optional).
4. Spatial filters and settings (optional).
5. The interpolation algorithm and uniform interpolation grid spacing (optional).
6. The re-segmentation range and outlier filtering (optional).
7. The discretisation method and relevant discretisation parameters, i.e. number of bins or bin size (optional).
8. Feature specific parameters, such as distance for some texture features (optional).

Optional descriptors are only added to the subscript if there are multiple possibilities. For example, if only CT data is used, adding the modality to the subscript is not required. Nonetheless, such details must be reported as well (see section 4.1).

The sections below have tables with permanent IBSI identifiers for concepts that were defined within this document.

4.2.1 Abbreviating feature families

The following is a list of the feature families in this document and their suggested abbreviations:

**feature family**	**abbreviation**	
morphology	MORPH	HCUG
local intensity	LI	9ST6
intensity-based statistics	IS, STAT	UHIW
intensity histogram	IH	ZVCW
intensity-volume histogram	IVH	P88C
grey level co-occurrence matrix	GLCM, CM	LFYI
grey level run length matrix	GLRLM, RLM	TP0I
grey level size zone matrix	GLSZM, SZM	9SAK
grey level distance zone matrix	GLDZM, DZM	VMDZ
neighbourhood grey tone difference matrix	NGTDM	IPET
neighbouring grey level dependence matrix	NGLDM	REK0

4.2.2 Abbreviating feature aggregation

The following is a list of feature families and the possible aggregation methods:

**morphology, LI**		
–	features are 3D by definition	DHQ4
**IS, IH, IVH**		
2D	averaged over slices (rare)	3IDG
–, 3D	calculated over the volume (default)	DHQ4
**GLCM, GLRLM**		
2D:avg	averaged over slices and directions	BTW3
2D:mrg, 2D:smrg	merged directions per slice and averaged	SUJT
2.5D:avg, 2.5D:dmrg	merged per direction and averaged	JJUI
2.5D:mrg, 2.5D:vmrg	merged over all slices	ZW7Z
3D:avg	averaged over 3D directions	ITBB
3D:mrg	merged 3D directions	IAZD
**GLSZM, GLDZM, NGTDM, NGLDM**		
2D	averaged over slices	8QNN
2.5D	merged over all slices	62GR
3D	calculated from single 3D matrix	KOBO

In the list above, '–' signifies an empty entry which does not need to be added to the subscript. The following examples highlight the nomenclature used above:

- joint maximum$_{\text{CM, 2D:avg}}$: GLCM-based *joint maximum* feature, calculated by averaging the feature for every in-slice GLCM.

- short runs emphasis$_{\text{RLM, 3D:mrg}}$: RLM-based *short runs emphasis* feature, calculated from an RLM that was aggregated by merging the RLM of each 3D direction.
- mean$_{\text{IS}}$: intensity statistical *mean* feature, calculated over the 3D ROI volume.
- grey level variance$_{\text{SZM, 2D}}$: SZM-based *grey level variance* feature, calculated by averaging the feature value from the SZM in each slice over all the slices.

4.2.3 Abbreviating interpolation

The following is a list of interpolation methods and the suggested notation. Note that # is the interpolation spacing, including units, and *dim* is 2D for interpolation with the slice plane and 3D for volumetric interpolation.

**interpolation method**	**notation**
none	INT:–
nearest neighbour interpolation	NNB:*dim*:#
linear interpolation	LIN:*dim*:#
cubic convolution interpolation	CCI:*dim*:#
cubic spline interpolation	CSI:*dim*:#, SI3:*dim*:#

The dimension attribute and interpolation spacing may be omitted if this is clear from the context. The following examples highlight the nomenclature introduced above:

- mean$_{\text{IS, LIN:2D:2mm}}$: intensity statistical *mean* feature, calculated after *bilinear* interpolation with the slice planes to uniform voxel sizes of 2mm.
- mean$_{\text{IH, NNB:3D:1mm}}$: intensity histogram *mean* feature, calculated after *trilinear* interpolation to uniform voxel sizes of 1mm.
- joint maximum$_{\text{CM, 2D:mrg, CSI:2D:2mm}}$: GLCM-based *joint maximum* feature, calculated by first merging all GLCM within a slice to single GLCM, calculating the feature and then averaging the feature values over the slices. GLCMs were determined in the image interpolated within the slice plane to 2 × 2mm voxels using *cubic spline* interpolation.

4.2.4 Describing re-segmentation

Re-segmentation can be noted as follows:

**re-segmentation method**	**notation**	
none	RS:–	
range	RS:[#,#]	USB3
outlier filtering	RS:#σ	7ACA

In the table above # signify numbers. A re-segmentation range can be half-open, i.e. RS:[#,∞). Re-segmentation methods may be combined, i.e. both range and outlier filtering methods may be used. This is noted as RS:[#,#]+#σ or RS:#σ+[#,#]. The following are examples of the application of the above notation:

- mean$_{\text{IS, CT, RS:[-200,150]}}$: intensity statistical *mean* feature, based on an ROI in a CT image that was re-segmented within a [-200,150] HU range.

- $\text{mean}_{\text{IS, PET:SUV, RS:}[3,\infty)}$: intensity statistical *mean* feature, based on an ROI in a PET image with SUV values, that was re-segmented to contain only SUV of 3 and above.
- $\text{mean}_{\text{IS, MRI:T1, RS:}3\sigma}$: intensity statistical *mean* feature, based on an ROI in a T1-weighted MR image where the ROI was re-segmented by removing voxels with an intensity outside a $\mu \pm 3\sigma$ range.

4.2.5 Abbreviating discretisation

The following is a list of discretisation methods and the suggested notation. Note that # is the value of the relevant discretisation parameter, e.g. number of bins or bin size, including units.

discretisation method	notation	
none	DIS:–	
fixed bin size	FBS:#	Q3RU
fixed bin number	FBN:#	K15C
histogram equalisation	EQ:#	
Lloyd-Max, minimum mean squared	LM:#, MMS:#	

In the table above, # signify numbers such as the number of bins or their width. Histogram equalisation of the ROI intensities can be performed before the "none", "fixed bin size", "fixed bin number" or "Lloyd-Max, minimum mean squared" algorithms defined above, with # specifying the number of bins in the histogram to be equalised. The following are examples of the application of the above notation:

- $\text{mean}_{\text{IH,PET:SUV,RS}[0,\infty]\text{,FBS:0.2}}$: intensity histogram *mean* feature, based on an ROI in a SUV-PET image, with bin-width of 0.2 SUV, and binning from 0.0 SUV.
- $\text{grey level variance}_{\text{SZM,MR:T1,RS:}3\sigma\text{,FBN:64}}$: size zone matrix-based *grey level variance* feature, based on an ROI in a T1-weighted MR image, with 3σ re-segmentation and subsequent binning into 64 bins.

4.2.6 Abbreviating feature-specific parameters

Some features and feature families require additional parameters, which may be varied. These are the following:

**grey level co-occurrence matrix**		
co-occurrence matrix symmetry		
–, SYM	symmetrical co-occurrence matrices	
ASYM	asymmetrical co-occurrence matrices (not recommended)	
distance		
δ:#, δ-∞:#	Chebyshev (ℓ_∞) norm with distance # (default)	PVMT
δ-2:#	Euclidean (ℓ_2) norm with distance #	G9EV
δ-1:#	Manhattan (ℓ_1) norm with distance #	LIFZ
distance weighting		

continued on next page

–, w:1	no weighting (default)	
w:f	weighting with function f	
**grey level run length matrix**		
distance weighting		
–, w:1	no weighting (default)	
w:f	weighting with function f	
**grey level size zone matrix**		
linkage distance		
δ:#, δ-∞:#	Chebyshev (ℓ_∞) norm with distance (default) #	PVMT
δ-2:#	Euclidean (ℓ_2) norm with distance #	G9EV
δ-1:#	Manhattan (ℓ_1) norm with distance #	LIFZ
**grey level distance zone matrix**		
linkage distance		
δ:#, δ-∞:#	Chebyshev (ℓ_∞) norm with distance (default) #	PVMT
δ-2:#	Euclidean (ℓ_2) norm with distance #	G9EV
δ-1:#	Manhattan (ℓ_1) norm with distance #	LIFZ
zone distance norm		
l-∞:#	Chebyshev (ℓ_∞) norm	PVMT
l-2:#	Euclidean (ℓ_2) norm	G9EV
–, l-1:#	Manhattan (ℓ_1) norm (default)	LIFZ
**neighbourhood grey tone difference matrix**		
distance		
δ:#, δ-∞:#	Chebyshev (ℓ_∞) norm with distance # (default)	PVMT
δ-2:#	Euclidean (ℓ_2) norm with distance #	G9EV
δ-1:#	Manhattan (ℓ_1) norm with distance #	LIFZ
distance weighting		
–, w:1	no weighting (default)	
w:f	weighting with function f	
**neighbouring grey level dependence matrix**		
dependence coarseness		
α:#	dependence coarseness parameter with value #	
distance		
δ:#, δ-∞:#	Chebyshev (ℓ_∞) norm with distance # (default)	PVMT
δ-2:#	Euclidean (ℓ_2) norm with distance #	G9EV
δ-1:#	Manhattan (ℓ_1) norm with distance #	LIFZ
distance weighting		
–, w:1	no weighting (default)	
w:f	weighting with function f	

In the above table, # represents numbers.

Chapter 5

Reference data sets

Reference values for features were obtained using a digital image phantom and the CT image of a lung cancer patient, which are described below. The same data sets can be used to verify radiomics software implementations. The data sets themselves may be found here: https://github.com/theibsi/data_sets.

5.1 Digital phantom

A small digital phantom was developed to derive image features manually and compare these values with values obtained from radiomics software implementations. The phantom is shown in figure 5.1. The phantom has the following characteristics:

- The phantom consists of 5 × 4 × 4 (x, y, z) voxels.
- A slice consists of the voxels in (x, y) plane for a particular slice at position z. Slices are therefore stacked in the z direction.
- Voxels are 2.0 × 2.0 × 2.0 mm in size.
- Not all voxels are included in the region of interest. Several excluded voxels are located on the outside of the ROI, and one internal voxel was excluded as well. Voxels excluded from the ROI are shown in blue in figure 5.1.
- Some intensities are not present in the phantom. Notably, grey levels 2 and 5 are absent. 1 is the lowest grey level present in the ROI, and 6 the highest.

5.1.1 Computing image features

The digital phantom was designed to not require image processing prior to calculating the features. Thus, feature calculation is done directly on the phantom itself. The following should be taken into account for calculating image features:

- Discretisation is not required. All features are to be calculated using the phantom as it is. Alternatively, one could use a *fixed bin size* discretisation of 1 or *fixed bin number* discretisation of 6 bins, which does not alter the contents of the phantom.
- Grey level co-occurrence matrices are symmetrical and calculated for (Chebyshev) distance $\delta = 1$.

- Neighbouring grey level dependence and neighbourhood grey tone difference matrices are likewise calculated for (Chebyshev) distance $\delta = 1$. Additionally, the neighbouring grey level dependence coarseness parameter has the value $\alpha = 0$.
- Because discretisation is lacking, most intensity-based statistical features will match their intensity histogram-based analogues in value.
- The ROI morphological and intensity masks are identical for the digital phantom, due to lack of re-segmentation.

5.2 Lung cancer CT image

A small data set of CT images from four non-small-cell lung carcinoma patients was made publicly available to serve as radiomics phantoms (DOI:10.17195/candat.2016.08.1). We use the image for the first patient (PAT1) to obtain feature reference values for different configurations of the image processing scheme, as detailed below.

The CT image set is stored as a stack of slices in DICOM format. The image slices can be identified by the DCM_IMG prefix. The gross tumour volume (GTV) was delineated and is used as the region of interest (ROI). Contour information is stored as an RT structure set in the DICOM file starting with DCM_RS. For broader use, both the DICOM set and segmentation mask have been converted to the NIfTI format. When using the data in NIfTI format, both image stacks should be converted to (at least) 32-bit floating point and rounded to the nearest integer before further processing.

We defined five image processing configurations to test different image processing methods, see Table 5.1. While most settings are self-explanatory, there are several aspects that require some attention. Configurations are divided in 2D and 3D approaches. For the 2D configurations (A, B), image interpolation is conducted within the slice, and likewise texture features are extracted from the in-slice plane, and not volumetrically (3D). For the 3D configurations (C-E) interpolation is conducted in three dimensions, and features are likewise extracted volumetrically. Discretisation is moreover required for texture, intensity histogram and intensity-volume histogram features, and both *fixed bin number* and *fixed bin size* algorithms are tested.

5.2.1 Notes on interpolation

Interpolation has a major influence on feature values. Different implementations of the same interpolation method may ostensibly provide the same functionality, but may use different interpolation grids. It is therefore recommended to read the documentation of the particular implementation to assess if the implementation allows or implements the following:

- The spatial origin of the original (input) grid in world coordinates matches the DICOM origin by definition.
- The size of the interpolation grid is determined by rounding the fractional grid size towards infinity, i.e. a ceiling operation. This prevents the interpolation grid from disappearing for very small images, but is otherwise an arbitrary choice.

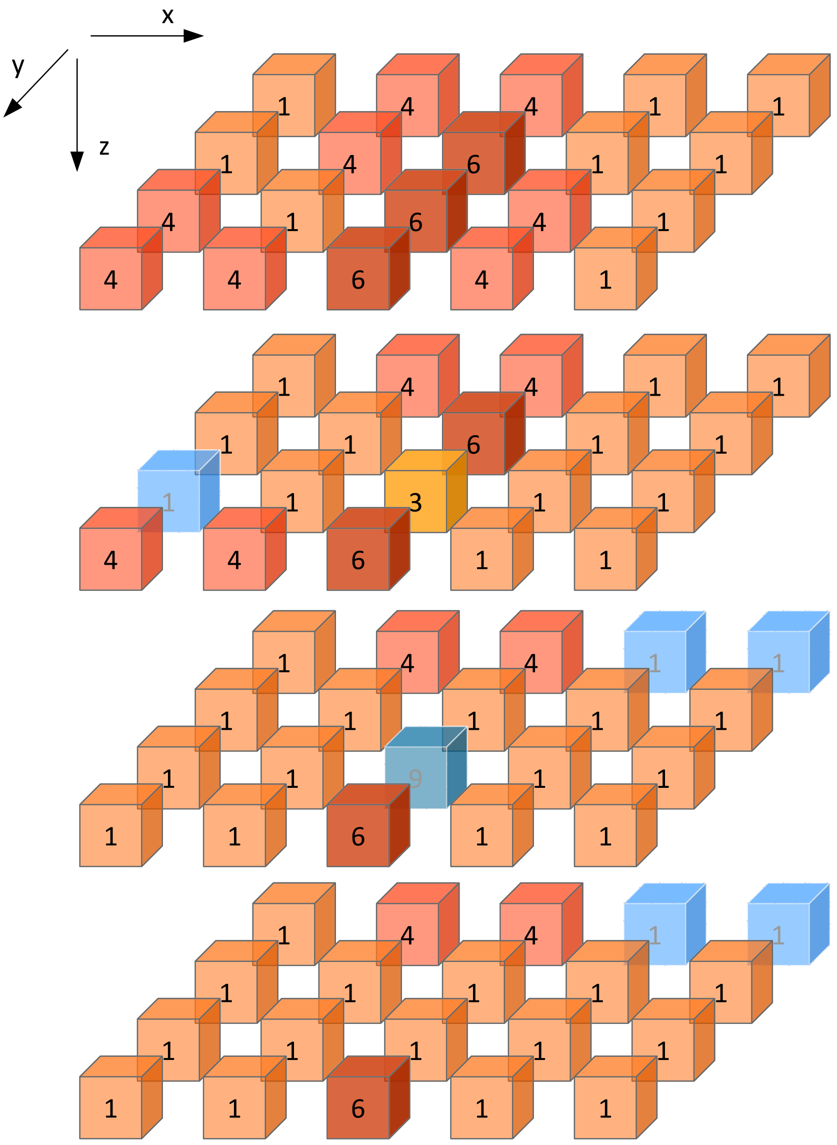

Figure 5.1 | Exploded view of the test volume. The number in each voxel corresponds with its grey level. Blue voxels are excluded from the region of interest. The coordinate system is so that x increases from left to right, y increases from back to front and z increases from top to bottom, as is indicated by the axis definition in the top-left.

- The centers of the interpolation and original image grids should be aligned, i.e. the interpolation grid is centered on the center of the original image grid. This prevents spacing inconsistencies in the interpolation grid and avoids potential issues with grid orientation.
- The extent of the interpolation grid is, by definition, always equal or larger than that of the original grid. This means that intensities at the grid boundary are extrapolated. To facilitate this process, the image should be sufficiently padded with voxels that take on the nearest boundary intensity.
- The floating point representation of the image and the ROI masks affects interpolation precision, and consequentially feature values. Image and ROI masks should at least be represented at full precision (32-bit) to avoid rounding errors. One example is the unintended exclusion of voxels from the interpolated ROI mask, which occurs when interpolation yields 0.4999...instead of 0.5. When images and ROI masks are converted to full precision from lower precision (e.g. 16-bit), values may require rounding if the original data were integer values, such as Hounsfield Units or the ROI mask labels.

More details are provided in Section 2.4.

5.2.2 Diagnostic features

Identifying issues with an implementation of the image processing sequence may be challenging. Multiple steps follow one another and differences propagate. Hence we define a small number of diagnostic features that describe how the image and ROI masks change with each image processing step. These diagnostic features also have reference values that may be found in IBSI compliance check spreadsheet.

Initial image stack. The following features may be used to describe the initial image stack (i.e. after loading image data for processing):

- *Image dimensions.* This describes the image dimensions in voxels along the different image axes.
- *Voxel dimensions.* This describes the voxel dimensions in mm. The dimension along the z-axis is equal to the distance between the origin voxels of two adjacent slices, and is generally equal to the slice thickness.
- *Mean intensity.* This is the average intensity within the entire image.
- *Minimum intensity.* This is the lowest intensity within the entire image.
- *Maximum intensity.* This is the highest intensity within the entire image.

Interpolated image stack. The above features may also be used to describe the image stack after image interpolation.

Initial region of interest. The following descriptors are used to describe the region of interest (ROI) directly after segmentation of the image:

- *ROI intensity mask dimensions.* This describes the dimensions, in voxels, of the ROI intensity mask.

- *ROI intensity mask bounding box dimensions.* This describes the dimensions, in voxels, of the bounding box of the ROI intensity mask.
- *ROI morphological mask bounding box dimensions.* This describes the dimensions, in voxels, of the bounding box of the ROI morphological mask.
- *Number of voxels in the ROI intensity mask.* This describes the number of voxels included in the ROI intensity mask.
- *Number of voxels in the ROI morphological mask.* This describes the number of voxels included in the ROI intensity mask.
- *Mean ROI intensity.* This is the mean intensity of image voxels within the ROI intensity mask.
- *Minimum ROI intensity.* This is the lowest intensity of image voxels within the ROI intensity mask.
- *Maximum ROI intensity.* This is the highest intensity of image voxels within the ROI intensity mask.

Interpolated region of interest. The same features can be used to describe the ROI after interpolation of the ROI mask.

Re-segmented region of interest. Again, the same features as above can be used to describe the ROI after re-segmentation.

5.2.3 Computing image features

Unlike the digital phantom, the lung cancer CT image does require additional image processing, which is done according to the processing configurations described in Table 5.1. The following should be taken into account when calculating image features:

- Grey level co-occurrence matrices are symmetrical and calculated for (Chebyshev) distance $\delta = 1$.
- Neighbouring grey level dependence and neighbourhood grey tone difference matrices are likewise calculated for (Chebyshev) distance $\delta = 1$. Additionally, the neighbouring grey level dependence coarseness parameter $\alpha = 0$.
- Intensity-based statistical features and their intensity histogram-based analogues will differ in value due to discretisation, in contrast to the same features for the digital phantom.
- Due to re-segmentation, the ROI morphological and intensity masks are not identical.
- Calculation of IVH feature: since by default CT contains calibrated and discrete intensities, no separate discretisation prior to the calculation of intensity-volume histogram features is required. This is the case for configurations A, B and D (i.e. 'calibrated intensity units – discrete case'). However, for configurations C and E, we re-discretise the ROI intensities prior to calculation of intensity-volume histogram features to allow for testing of of these methods. Configuration C simulates the 'calibrated intensity units – continuous case', while configuration E simulates the 'arbitrary intensity units'

case where the re-segmentation range is not used. For details, please consult section 3.5.

Parameter	Config. A	Config. B	Config. C	Config. D	Config. E
sample identifier	PAT1	PAT1	PAT1	PAT1	PAT1
ROI name	GTV-1	GTV-1	GTV-1	GTV-1	GTV-1
slice-wise or single volume (3D)	2D	2D	3D	3D	3D
interpolation	no	yes	yes	yes	yes
resampled voxel spacing (mm)		2×2 (axial)	$2 \times 2 \times 2$	$2 \times 2 \times 2$	$2 \times 2 \times 2$
interpolation method		bilinear	trilinear	trilinear	tricubic spline
intensity rounding		nearest integer	nearest integer	nearest integer	nearest integer
ROI interpolation method		bilinear	trilinear	trilinear	trilinear
ROI partial mask volume		0.5	0.5	0.5	0.5
re-segmentation					
range (HU)	$[-500, 400]$	$[-500, 400]$	$[-1000, 400]$	no	$[-1000, 400]$
outlier filtering	no	no	no	3σ	3σ
discretisation					
texture and IH	FBS: 25 HU	FBN: 32 bins	FBS: 25 HU	FBN: 32 bins	FBN: 32 bins
IVH	no	no	FBS: 2.5 HU	no	FBN: 1000 bins
texture parameters					
GLCM, NGTDM, NGLDM distance	1	1	1	1	1
GLSZM, GLDZM linkage distance	1	1	1	1	1
NGLDM coarseness	0.0	0.0	0.0	0.0	0.0

Table 5.1 | Different configurations for image processing. For details, refer to the corresponding sections in chapter 2. ROI: region of interest; HU: Hounsfield Unit; IH: intensity histogram; FBS: fixed bin size; FBN: fixed bin number; IVH: intensity-volume histogram; GLCM: grey level co-occurrence matrix; NGTDM: neighborhood grey tone difference matrix; NGLDM: neighbouring grey level dependence matrix; GLSZM: grey level size zone matrix; GLDZM: grey level distance zone matrix.

Appendix A

Digital phantom texture matrices

This section contains the texture matrices extracted from the digital phantom for reference purposes.

A.1 Grey level co-occurrence matrix (2D)

i	j	n
1.0	1.0	10
1.0	4.0	4
4.0	1.0	4
4.0	4.0	6
4.0	6.0	1
6.0	4.0	1
6.0	6.0	4

(a) x: (0,1,0)
slice: 1 of 4

i	j	n
1.0	1.0	16
1.0	4.0	2
3.0	6.0	2
4.0	1.0	2
4.0	6.0	1
6.0	3.0	2
6.0	4.0	1

(b) x: (0,1,0)
slice: 2 of 4

i	j	n
1.0	1.0	18
1.0	4.0	2
4.0	1.0	2

(c) x: (0,1,0)
slice: 3 of 4

i	j	n
1.0	1.0	20
1.0	4.0	2
1.0	6.0	1
4.0	1.0	2
6.0	1.0	1

(d) x: (0,1,0)
slice: 4 of 4

i	j	n
1.0	1.0	2
1.0	4.0	4
1.0	6.0	3
4.0	1.0	4
4.0	4.0	4
4.0	6.0	2
6.0	1.0	3
6.0	4.0	2

(e) x: (1,-1,0)
slice: 1 of 4

i	j	n
1.0	1.0	6
1.0	3.0	1
1.0	4.0	3
1.0	6.0	3
3.0	1.0	1
3.0	4.0	1
4.0	1.0	3
4.0	3.0	1
6.0	1.0	3

(f) x: (1,-1,0)
slice: 2 of 4

i	j	n
1.0	1.0	10
1.0	4.0	2
1.0	6.0	1
4.0	1.0	2
6.0	1.0	1

(g) x: (1,-1,0)
slice: 3 of 4

i	j	n
1.0	1.0	14
1.0	4.0	2
1.0	6.0	1
4.0	1.0	2
6.0	1.0	1

(h) x: (1,-1,0)
slice: 4 of 4

i	j	n
1.0	1.0	4
1.0	4.0	6
1.0	6.0	2
4.0	1.0	6
4.0	4.0	4
4.0	6.0	4
6.0	1.0	2
6.0	4.0	4

(i) d: (1,0,0)
slice: 1 of 4

i	j	n
1.0	1.0	10
1.0	3.0	2
1.0	4.0	2
1.0	6.0	3
3.0	1.0	2
4.0	1.0	2
4.0	4.0	4
4.0	6.0	1
6.0	1.0	3
6.0	4.0	1

(j) d: (1,0,0)
slice: 2 of 4

i	j	n
1.0	1.0	16
1.0	4.0	1
1.0	6.0	2
4.0	1.0	1
4.0	4.0	2
6.0	1.0	2

(k) d: (1,0,0)
slice: 3 of 4

i	j	n
1.0	1.0	20
1.0	4.0	1
1.0	6.0	2
4.0	1.0	1
4.0	4.0	2
6.0	1.0	2

(l) d: (1,0,0)
slice: 4 of 4

i	j	n
1.0	1.0	6
1.0	4.0	3
1.0	6.0	1
4.0	1.0	3
4.0	4.0	2
4.0	6.0	4
6.0	1.0	1
6.0	4.0	4

(m) d: (1,1,0)
slice: 1 of 4

i	j	n
1.0	1.0	10
1.0	3.0	2
1.0	4.0	1
1.0	6.0	2
3.0	1.0	2
4.0	1.0	1
4.0	6.0	1
6.0	1.0	2
6.0	4.0	1

(n) d: (1,1,0)
slice: 2 of 4

i	j	n
1.0	1.0	12
1.0	4.0	2
1.0	6.0	1
4.0	1.0	2
6.0	1.0	1

(o) d: (1,1,0)
slice: 3 of 4

i	j	n
1.0	1.0	16
1.0	4.0	2
1.0	6.0	1
4.0	1.0	2
6.0	1.0	1

(p) d: (1,1,0)
slice: 4 of 4

Table A.1 | Grey-level co-occurrence matrices extracted from the xy plane (2D) of the digital phantom using Chebyshev distance 1. **x** indicates the direction in (x, y, z) coordinates.

A.2 Grey level co-occurrence matrix (2D, merged)

i	j	n
1.0	1.0	22
1.0	4.0	17
1.0	6.0	6
4.0	1.0	17
4.0	4.0	16
4.0	6.0	11
6.0	1.0	6
6.0	4.0	11
6.0	6.0	4

(a) slice: 1 of 4

i	j	n
1.0	1.0	42
1.0	3.0	5
1.0	4.0	8
1.0	6.0	8
3.0	1.0	5
3.0	4.0	1
3.0	6.0	2
4.0	1.0	8
4.0	3.0	1
4.0	4.0	4
4.0	6.0	3
6.0	1.0	8
6.0	3.0	2
6.0	4.0	3

(b) slice: 2 of 4

i	j	n
1.0	1.0	56
1.0	4.0	7
1.0	6.0	4
4.0	1.0	7
4.0	4.0	2
6.0	1.0	4

(c) slice: 3 of 4

i	j	n
1.0	1.0	70
1.0	4.0	7
1.0	6.0	5
4.0	1.0	7
4.0	4.0	2
6.0	1.0	5

(d) slice: 4 of 4

Table A.2 | Merged grey-level co-occurrence matrices extracted from the xy plane (2D) of the digital phantom using Chebyshev distance 1.

A.3 Grey level co-occurrence matrix (3D)

i	j	n
1.0	1.0	66
1.0	4.0	5
1.0	6.0	1
3.0	6.0	1
4.0	1.0	5
4.0	4.0	16
6.0	1.0	1
6.0	3.0	1
6.0	6.0	8

(a) x: (0,0,1)

i	j	n
1.0	1.0	42
1.0	3.0	1
1.0	4.0	9
1.0	6.0	1
3.0	1.0	1
3.0	6.0	1
4.0	1.0	9
4.0	4.0	2
4.0	6.0	2
6.0	1.0	1
6.0	3.0	1
6.0	4.0	2
6.0	6.0	2

(b) x: (0,1,-1)

i	j	n
1.0	1.0	64
1.0	4.0	10
1.0	6.0	1
3.0	6.0	2
4.0	1.0	10
4.0	4.0	6
4.0	6.0	2
6.0	1.0	1
6.0	3.0	2
6.0	4.0	2
6.0	6.0	4

(c) x: (0,1,0)

i	j	n
1.0	1.0	52
1.0	4.0	8
3.0	6.0	2
4.0	1.0	8
4.0	4.0	2
4.0	6.0	1
6.0	3.0	2
6.0	4.0	1
6.0	6.0	2

(d) x: (0,1,1)

i	j	n
1.0	1.0	30
1.0	3.0	2
1.0	4.0	7
1.0	6.0	5
3.0	1.0	2
4.0	1.0	7
4.0	6.0	2
6.0	1.0	5
6.0	4.0	2

(e) x: (1,-1,-1)

i	j	n
1.0	1.0	32
1.0	3.0	1
1.0	4.0	11
1.0	6.0	8
3.0	1.0	1
3.0	4.0	1
4.0	1.0	11
4.0	3.0	1
4.0	4.0	4
4.0	6.0	2
6.0	1.0	8
6.0	4.0	2

(f) x: (1,-1,0)

i	j	n
1.0	1.0	20
1.0	3.0	1
1.0	4.0	10
1.0	6.0	6
3.0	1.0	1
3.0	4.0	1
4.0	1.0	10
4.0	3.0	1
4.0	4.0	2
6.0	1.0	6

(g) x: (1,-1,1)

i	j	n
1.0	1.0	38
1.0	3.0	1
1.0	4.0	7
1.0	6.0	8
3.0	1.0	1
3.0	4.0	1
4.0	1.0	7
4.0	3.0	1
4.0	4.0	8
4.0	6.0	2
6.0	1.0	8
6.0	4.0	2

(h) x: (1,0,-1)

i	j	n
1.0	1.0	50
1.0	3.0	2
1.0	4.0	10
1.0	6.0	9
3.0	1.0	2
4.0	1.0	10
4.0	4.0	12
4.0	6.0	5
6.0	1.0	9
6.0	4.0	5

(i) x: (1,0,0)

i	j	n
1.0	1.0	34
1.0	3.0	2
1.0	4.0	8
1.0	6.0	7
3.0	1.0	2
4.0	1.0	8
4.0	4.0	8
4.0	6.0	3
6.0	1.0	7
6.0	4.0	3

(j) x: (1,0,1)

i	j	n
1.0	1.0	32
1.0	3.0	1
1.0	4.0	6
1.0	6.0	4
3.0	1.0	1
3.0	4.0	1
4.0	1.0	6
4.0	3.0	1
4.0	6.0	3
6.0	1.0	4
6.0	4.0	3

(k) x: (1,1,-1)

i	j	n
1.0	1.0	44
1.0	3.0	2
1.0	4.0	8
1.0	6.0	5
3.0	1.0	2
4.0	1.0	8
4.0	4.0	2
4.0	6.0	5
6.0	1.0	5
6.0	4.0	5

(l) x: (1,1,0)

i	j	n
1.0	1.0	32
1.0	3.0	1
1.0	4.0	6
1.0	6.0	6
3.0	1.0	1
3.0	4.0	1
4.0	1.0	6
4.0	3.0	1
4.0	4.0	2
4.0	6.0	1
6.0	1.0	6
6.0	4.0	1

(m) x: (1,1,1)

Table A.3 | Grey-level co-occurrence matrices extracted volumetrically (3D) from the digital phantom using Chebyshev distance 1. **x** indicates the direction in (x, y, z) coordinates.

A.4 Grey level co-occurrence matrix (3D, merged)

i	j	n
1.0	1.0	536
1.0	3.0	14
1.0	4.0	105
1.0	6.0	61
3.0	1.0	14
3.0	4.0	5
3.0	6.0	6
4.0	1.0	105
4.0	3.0	5
4.0	4.0	64
4.0	6.0	28
6.0	1.0	61
6.0	3.0	6
6.0	4.0	28
6.0	6.0	16

Table A.4 | Merged grey-level co-occurrence matrix extracted volumetrically (3D) from the digital phantom using Chebyshev distance 1.

A.5 Grey level run length matrix (2D)

i	r	n
1.0	1.0	1.0
1.0	2.0	2.0
1.0	4.0	1.0
4.0	1.0	2.0
4.0	2.0	3.0
6.0	3.0	1.0

(a) x: (0,1,0)
slice: 1 of 4

i	r	n
1.0	2.0	2.0
1.0	4.0	2.0
3.0	1.0	1.0
4.0	1.0	4.0
6.0	1.0	2.0

(b) x: (0,1,0)
slice: 2 of 4

i	r	n
1.0	1.0	1.0
1.0	3.0	3.0
1.0	4.0	1.0
4.0	1.0	2.0
6.0	1.0	1.0

(c) x: (0,1,0)
slice: 3 of 4

i	r	n
1.0	2.0	1.0
1.0	3.0	3.0
1.0	4.0	1.0
4.0	1.0	2.0
6.0	1.0	1.0

(d) x: (0,1,0)
slice: 4 of 4

i	r	n
1.0	1.0	7.0
1.0	2.0	1.0
4.0	1.0	5.0
4.0	3.0	1.0
6.0	1.0	3.0

(e) x: (1,-1,0)
slice: 1 of 4

i	r	n
1.0	1.0	6.0
1.0	2.0	3.0
3.0	1.0	1.0
4.0	1.0	4.0
6.0	1.0	2.0

(f) x: (1,-1,0)
slice: 2 of 4

i	r	n
1.0	1.0	5.0
1.0	2.0	3.0
1.0	3.0	1.0
4.0	1.0	2.0
6.0	1.0	1.0

(g) x: (1,-1,0)
slice: 3 of 4

i	r	n
1.0	1.0	3.0
1.0	2.0	3.0
1.0	3.0	2.0
4.0	1.0	2.0
6.0	1.0	1.0

(h) x: (1,-1,0)
slice: 4 of 4

i	r	n
1.0	1.0	5.0
1.0	2.0	2.0
4.0	1.0	4.0
4.0	2.0	2.0
6.0	1.0	3.0

(i) x: (1,0,0)
slice: 1 of 4

i	r	n
1.0	1.0	2.0
1.0	2.0	5.0
3.0	1.0	1.0
4.0	2.0	2.0
6.0	1.0	2.0

(j) x: (1,0,0)
slice: 2 of 4

i	r	n
1.0	1.0	1.0
1.0	2.0	4.0
1.0	5.0	1.0
4.0	2.0	1.0
6.0	1.0	1.0

(k) x: (1,0,0)
slice: 3 of 4

i	r	n
1.0	1.0	1.0
1.0	2.0	2.0
1.0	5.0	2.0
4.0	2.0	1.0
6.0	1.0	1.0

(l) x: (1,0,0)
slice: 4 of 4

i	r	n
1.0	1.0	3.0
1.0	2.0	3.0
4.0	1.0	6.0
4.0	2.0	1.0
6.0	1.0	3.0

(m) x: (1,1,0)
slice: 1 of 4

i	r	n
1.0	1.0	2.0
1.0	2.0	5.0
3.0	1.0	1.0
4.0	1.0	4.0
6.0	1.0	2.0

(n) x: (1,1,0)
slice: 2 of 4

i	r	n
1.0	1.0	3.0
1.0	2.0	4.0
1.0	3.0	1.0
4.0	1.0	2.0
6.0	1.0	1.0

(o) x: (1,1,0)
slice: 3 of 4

i	r	n
1.0	1.0	2.0
1.0	2.0	3.0
1.0	3.0	1.0
1.0	4.0	1.0
4.0	1.0	2.0
6.0	1.0	1.0

(p) x: (1,1,0)
slice: 4 of 4

Table A.5 | Grey-level run length matrices extracted from the xy plane (2D) of the digital phantom. **x** indicates the direction in (x, y, z) coordinates.

A.6 Grey level run length matrix (2D, merged)

i	r	n
1.0	1.0	16.0
1.0	2.0	8.0
1.0	4.0	1.0
4.0	1.0	17.0
4.0	2.0	6.0
4.0	3.0	1.0
6.0	1.0	9.0
6.0	3.0	1.0

(a) slice: 1 of 4

i	r	n
1.0	1.0	10.0
1.0	2.0	15.0
1.0	4.0	2.0
3.0	1.0	4.0
4.0	1.0	12.0
4.0	2.0	2.0
6.0	1.0	8.0

(b) slice: 2 of 4

i	r	n
1.0	1.0	10.0
1.0	2.0	11.0
1.0	3.0	5.0
1.0	4.0	1.0
1.0	5.0	1.0
4.0	1.0	6.0
4.0	2.0	1.0
6.0	1.0	4.0

(c) slice: 3 of 4

i	r	n
1.0	1.0	6.0
1.0	2.0	9.0
1.0	3.0	6.0
1.0	4.0	2.0
1.0	5.0	2.0
4.0	1.0	6.0
4.0	2.0	1.0
6.0	1.0	4.0

(d) slice: 4 of 4

Table A.6 | Merged grey-level run length matrices extracted from the xy plane (2D) of the digital phantom.

A.7 Grey level run length matrix (3D)

i	r	n
1.0	1.0	1.0
1.0	2.0	6.0
1.0	3.0	3.0
1.0	4.0	7.0
3.0	1.0	1.0
4.0	1.0	4.0
4.0	2.0	2.0
4.0	4.0	2.0
6.0	1.0	1.0
6.0	2.0	1.0
6.0	4.0	1.0

(a) x: (0,0,1)

i	r	n
1.0	1.0	11.0
1.0	2.0	15.0
1.0	3.0	3.0
3.0	1.0	1.0
4.0	1.0	14.0
4.0	2.0	1.0
6.0	1.0	5.0
6.0	2.0	1.0

(b) x: (0,1,-1)

i	r	n
1.0	1.0	2.0
1.0	2.0	5.0
1.0	3.0	6.0
1.0	4.0	5.0
3.0	1.0	1.0
4.0	1.0	10.0
4.0	2.0	3.0
6.0	1.0	4.0
6.0	3.0	1.0

(c) x: (0,1,0)

i	r	n
1.0	1.0	10.0
1.0	2.0	5.0
1.0	3.0	6.0
1.0	4.0	3.0
3.0	1.0	1.0
4.0	1.0	14.0
4.0	2.0	1.0
6.0	1.0	5.0
6.0	2.0	1.0

(d) x: (0,1,1)

i	r	n
1.0	1.0	22.0
1.0	2.0	11.0
1.0	3.0	2.0
3.0	1.0	1.0
4.0	1.0	16.0
6.0	1.0	7.0

(e) x: (1,-1,-1)

i	r	n
1.0	1.0	21.0
1.0	2.0	10.0
1.0	3.0	3.0
3.0	1.0	1.0
4.0	1.0	13.0
4.0	3.0	1.0
6.0	1.0	7.0

(f) x: (1,-1,0)

i	r	n
1.0	1.0	30.0
1.0	2.0	10.0
3.0	1.0	1.0
4.0	1.0	14.0
4.0	2.0	1.0
6.0	1.0	7.0

(g) x: (1,-1,1)

i	r	n
1.0	1.0	16.0
1.0	2.0	12.0
1.0	3.0	2.0
1.0	4.0	1.0
3.0	1.0	1.0
4.0	1.0	8.0
4.0	2.0	4.0
6.0	1.0	7.0

(h) x: (1,0,-1)

i	r	n
1.0	1.0	9.0
1.0	2.0	13.0
1.0	5.0	3.0
3.0	1.0	1.0
4.0	1.0	4.0
4.0	2.0	6.0
6.0	1.0	7.0

(i) x: (1,0,0)

i	r	n
1.0	1.0	19.0
1.0	2.0	12.0
1.0	3.0	1.0
1.0	4.0	1.0
3.0	1.0	1.0
4.0	1.0	8.0
4.0	2.0	4.0
6.0	1.0	7.0

(j) x: (1,0,1)

i	r	n
1.0	1.0	20.0
1.0	2.0	12.0
1.0	3.0	2.0
3.0	1.0	1.0
4.0	1.0	16.0
6.0	1.0	7.0

(k) x: (1,1,-1)

i	r	n
1.0	1.0	10.0
1.0	2.0	15.0
1.0	3.0	2.0
1.0	4.0	1.0
3.0	1.0	1.0
4.0	1.0	14.0
4.0	2.0	1.0
6.0	1.0	7.0

(l) x: (1,1,0)

i	r	n
1.0	1.0	19.0
1.0	2.0	14.0
1.0	3.0	1.0
3.0	1.0	1.0
4.0	1.0	14.0
4.0	2.0	1.0
6.0	1.0	7.0

(m) x: (1,1,1)

Table A.7 | Grey-level run length matrices extracted volumetrically (3D) from the digital phantom. **x** indicates the direction in (x, y, z) coordinates.

A.8 Grey level run length matrix (3D, merged)

i	r	n
1.0	1.0	190.0
1.0	2.0	140.0
1.0	3.0	31.0
1.0	4.0	18.0
1.0	5.0	3.0
3.0	1.0	13.0
4.0	1.0	149.0
4.0	2.0	24.0
4.0	3.0	1.0
4.0	4.0	2.0
6.0	1.0	78.0
6.0	2.0	3.0
6.0	3.0	1.0
6.0	4.0	1.0

Table A.8 | Merged grey-level run length matrix extracted volumetrically (3D) from the digital phantom.

A.9 Grey level size zone matrix (2D)

i	s	n
1.0	3	1
1.0	6	1
4.0	2	1
4.0	6	1
6.0	3	1

(a) slice: 1 of 4

i	s	n
1.0	4	1
1.0	8	1
3.0	1	1
4.0	2	2
6.0	1	2

(b) slice: 2 of 4

i	s	n
1.0	14	1
4.0	2	1
6.0	1	1

(c) slice: 3 of 4

i	s	n
1.0	15	1
4.0	2	1
6.0	1	1

(d) slice: 4 of 4

Table A.9 | Grey level size zone matrices extracted from the xy plane (2D) of the digital phantom.

A.10 Grey level size zone matrix (3D)

i	s	n
1.0	50	1
3.0	1	1
4.0	2	1
4.0	14	1
6.0	7	1

Table A.10 | Grey level size zone matrix extracted volumetrically (3D) from the digital phantom.

A.11 Grey level distance zone matrix (2D)

i	d	n
1.0	1.0	2
4.0	1.0	2
6.0	1.0	1

(a) slice: 1 of 4

i	d	n
1.0	1.0	2
3.0	2.0	1
4.0	1.0	2
6.0	1.0	1
6.0	2.0	1

(b) slice: 2 of 4

i	d	n
1.0	1.0	1
4.0	1.0	1
6.0	1.0	1

(c) slice: 3 of 4

i	d	n
1.0	1.0	1
4.0	1.0	1
6.0	1.0	1

(d) slice: 4 of 4

Table A.11 | Grey level distance zone matrices extracted from the xy plane (2D) of the digital phantom.

A.12 Grey level distance zone matrix (3D)

i	d	n
1.0	1.0	1
3.0	1.0	1
4.0	1.0	2
6.0	1.0	1

Table A.12 | Grey level distance zone matrix extracted volumetrically (3D) from the digital phantom.

A.13 Neighbourhood grey tone difference matrix (2D)

i	s	n
1.0	14.575	9
4.0	5.775	8
6.0	7.325	3

(a) slice: 1 of 4

i	s	n
1.0	11.928571	12
3.0	0.375000	1
4.0	4.800000	4
6.0	8.000000	2

(b) slice: 2 of 4

i	s	n
1.0	7.985714	14
4.0	4.650000	2
6.0	5.000000	1

(c) slice: 3 of 4

i	s	n
1.0	7.582143	15
4.0	4.650000	2
6.0	5.000000	1

(d) slice: 4 of 4

Table A.13 | Neighbourhood grey tone difference matrices extracted from the xy plane (2D) of the digital phantom using Chebyshev distance 1.

A.14 Neighbourhood grey tone difference matrix (3D)

i	s	n
1.0	39.946954	50
3.0	0.200000	1
4.0	20.825401	16
6.0	24.127005	7

Table A.14 | Neighbourhood grey tone difference matrix extracted volumetrically (3D) from the digital phantom using Chebyshev distance 1.

A.15 Neighbouring grey level dependence matrix (2D)

i	j	s
1.0	2.0	3
1.0	3.0	1
1.0	4.0	3
1.0	5.0	2
4.0	2.0	2
4.0	3.0	4
4.0	4.0	2
6.0	2.0	2
6.0	3.0	1

(a) slice: 1 of 4

i	j	s
1.0	3.0	2
1.0	4.0	6
1.0	6.0	4
3.0	1.0	1
4.0	2.0	4
6.0	1.0	2

(b) slice: 2 of 4

i	j	s
1.0	3.0	1
1.0	4.0	5
1.0	5.0	3
1.0	6.0	3
1.0	7.0	2
4.0	2.0	2
6.0	1.0	1

(c) slice: 3 of 4

i	j	s
1.0	3.0	1
1.0	4.0	3
1.0	5.0	3
1.0	6.0	4
1.0	7.0	1
1.0	8.0	3
4.0	2.0	2
6.0	1.0	1

(d) slice: 4 of 4

Table A.15 | Neighbouring grey level dependence matrices extracted from the xy plane (2D) of the digital phantom using Chebyshev distance 1 and coarseness 0.

A.16 Neighbouring grey level dependence matrix (3D)

i	j	s
1.0	5.0	2
1.0	6.0	2
1.0	7.0	1
1.0	8.0	6
1.0	9.0	4
1.0	10.0	6
1.0	11.0	5
1.0	12.0	5
1.0	13.0	3
1.0	14.0	2
1.0	15.0	5
1.0	16.0	3
1.0	17.0	3
1.0	18.0	2
1.0	21.0	1
3.0	1.0	1
4.0	2.0	2
4.0	4.0	2
4.0	5.0	6
4.0	6.0	4
4.0	7.0	2
6.0	2.0	1
6.0	3.0	4
6.0	4.0	1
6.0	5.0	1

Table A.16 | Neighbouring grey level dependence matrix extracted volumetrically (3D) from the digital phantom using Chebyshev distance 1 and coarseness 0.

Bibliography

[1] H. J. W. L. Aerts, E. Rios-Velazquez, R. T. H. Leijenaar, C. Parmar, P. Grossmann, S. Cavalho, J. Bussink, R. Monshouwer, B. Haibe-Kains, D. Rietveld, F. J. P. Hoebers, M. M. Rietbergen, C. R. Leemans, A. Dekker, J. Quackenbush, R. J. Gillies, and P. Lambin. Decoding tumour phenotype by noninvasive imaging using a quantitative radiomics approach. *Nature communications*, 5:4006, 2014.

[2] S. D. Ahipaşaoğlu. Fast algorithms for the minimum volume estimator. *Journal of Global Optimization*, 62(2):351–370, 2015.

[3] F. Albregtsen, B. Nielsen, and H. Danielsen. Adaptive gray level run length features from class distance matrices. In *Proceedings 15th International Conference on Pattern Recognition. ICPR-2000*, volume 3, pages 738–741. IEEE Comput. Soc, 2000.

[4] B. A. Altazi, G. G. Zhang, D. C. Fernandez, M. E. Montejo, D. Hunt, J. Werner, M. C. Biagioli, and E. G. Moros. Reproducibility of F18-FDG PET radiomic features for different cervical tumor segmentation methods, gray-level discretization, and reconstruction algorithms. *Journal of applied clinical medical physics*, 18(6):32–48, 2017.

[5] M. Amadasun and R. King. Textural features corresponding to textural properties. *IEEE Transactions on Systems, Man and Cybernetics*, 19(5):1264–1273, 1989.

[6] I. Apostolova, I. G. Steffen, F. Wedel, A. Lougovski, S. Marnitz, T. Derlin, H. Amthauer, R. Buchert, F. Hofheinz, and W. Brenner. Asphericity of pretherapeutic tumour FDG uptake provides independent prognostic value in head-and-neck cancer. *European radiology*, 24(9):2077–87, 2014.

[7] J. Atkinson A.J., W. Colburn, V. DeGruttola, D. DeMets, G. Downing, D. Hoth, J. Oates, C. Peck, R. Schooley, B. Spilker, J. Woodcock, and S. Zeger. Biomarkers and surrogate endpoints: Preferred definitions and conceptual framework. *Clinical Pharmacology and Therapeutics*, 69(3):89–95, 2001.

[8] C. Bailly, C. Bodet-Milin, S. Couespel, H. Necib, F. Kraeber-Bodéré, C. Ansquer, and T. Carlier. Revisiting the Robustness of PET-Based Textural Features in the Context of Multi-Centric Trials. *PloS one*, 11(7):e0159984, 2016.

[9] M. A. Balafar, A. R. Ramli, M. I. Saripan, and S. Mashohor. Review of brain MRI image segmentation methods. *Artificial Intelligence Review*, 33(3):261–274, 2010.

[10] G. Barequet and S. Har-Peled. Efficiently Approximating the Minimum-Volume Bounding Box of a Point Set in Three Dimensions. *Journal of Algorithms*, 38(1):91–109, 2001.

[11] R. Boellaard, R. Delgado-Bolton, W. J. G. Oyen, F. Giammarile, K. Tatsch, W. Eschner, F. J. Verzijlbergen, S. F. Barrington, L. C. Pike, W. A. Weber, S. G. Stroobants, D. Delbeke, K. J. Donohoe, S. Holbrook, M. M. Graham, G. Testanera, O. S. Hoekstra, J. M. Zijlstra, E. P. Visser, C. J. Hoekstra, J. Pruim, A. T. Willemsen, B. Arends, J. Kotzerke, A. Bockisch,

T. Beyer, A. Chiti, and B. J. Krause. FDG PET/CT: EANM procedure guidelines for tumour imaging: version 2.0. *European journal of nuclear medicine and molecular imaging*, 42(2): 328–54, 2015.

[12] N. Boussion, C. C. Le Rest, M. Hatt, and D. Visvikis. Incorporation of wavelet-based denoising in iterative deconvolution for partial volume correction in whole-body PET imaging. *European journal of nuclear medicine and molecular imaging*, 36(7):1064–75, 2009.

[13] J. C. Caicedo, S. Cooper, F. Heigwer, S. Warchal, P. Qiu, C. Molnar, A. S. Vasilevich, J. D. Barry, H. S. Bansal, O. Kraus, M. Wawer, L. Paavolainen, M. D. Herrmann, M. Rohban, J. Hung, H. Hennig, J. Concannon, I. Smith, P. A. Clemons, S. Singh, P. Rees, P. Horvath, R. G. Linington, and A. E. Carpenter. Data-analysis strategies for image-based cell profiling. *Nature Methods*, 14(9):849–863, 2017.

[14] C. Chan and S. Tan. Determination of the minimum bounding box of an arbitrary solid: an iterative approach. *Computers and Structures*, 79(15):1433–1449, 2001.

[15] A. Chu, C. M. Sehgal, and J. F. Greenleaf. Use of gray value distribution of run lengths for texture analysis. *Pattern Recognition Letters*, 11(6):415–419, 1990.

[16] K. Clark, B. Vendt, K. Smith, J. Freymann, J. Kirby, P. Koppel, S. Moore, S. Phillips, D. Maffitt, M. Pringle, L. Tarbox, and F. Prior. The Cancer Imaging Archive (TCIA): maintaining and operating a public information repository. *Journal of digital imaging*, 26(6):1045–57, 2013.

[17] L. P. Clarke, R. J. Nordstrom, H. Zhang, P. Tandon, Y. Zhang, G. Redmond, K. Farahani, G. Kelloff, L. Henderson, L. Shankar, J. Deye, J. Capala, and P. Jacobs. The Quantitative Imaging Network: NCI's Historical Perspective and Planned Goals. *Translational oncology*, 7(1):1–4, 2014.

[18] D. A. Clausi. An analysis of co-occurrence texture statistics as a function of grey level quantization. *Canadian Journal of Remote Sensing*, 28(1):45–62, 2002.

[19] G. Collewet, M. Strzelecki, and F. Mariette. Influence of MRI acquisition protocols and image intensity normalization methods on texture classification. *Magnetic resonance imaging*, 22(1):81–91, 2004.

[20] E. C. Da Silva, A. C. Silva, A. C. De Paiva, and R. A. Nunes. Diagnosis of lung nodule using Moran's index and Geary's coefficient in computerized tomography images. *Pattern Analysis and Applications*, 11(1):89–99, 2008.

[21] M. R. T. Dale, P. Dixon, M.-J. Fortin, P. Legendre, D. E. Myers, and M. S. Rosenberg. Conceptual and mathematical relationships among methods for spatial analysis. *Ecography*, 25(5):558–577, 2002.

[22] B. V. Dasarathy and E. B. Holder. Image characterizations based on joint gray level-run length distributions. *Pattern Recognition Letters*, 12(8):497–502, 1991.

[23] A. Depeursinge and J. Fageot. Biomedical Texture Operators and Aggregation Functions. In A. Depeursinge, J. Fageot, and O. Al-Kadi, editors, *Biomedical texture analysis*, chapter 3, pages 63–101. Academic Press, London, UK, 1st edition, 2017.

[24] A. Depeursinge, A. Foncubierta-Rodriguez, D. Van De Ville, and H. Müller. Three-dimensional solid texture analysis in biomedical imaging: review and opportunities. *Medical image analysis*, 18(1):176–96, 2014.

[25] M.-C. Desseroit, F. Tixier, W. A. Weber, B. A. Siegel, C. Cheze Le Rest, D. Visvikis, and M. Hatt. Reliability of PET/CT Shape and Heterogeneity Features in Functional and Morphologic Components of Non-Small Cell Lung Cancer Tumors: A Repeatability Analysis in a Prospective Multicenter Cohort. *Journal of nuclear medicine*, 58(3):406–411, 2017.

[26] I. El Naqa. *Image Processing and Analysis of PET and Hybrid PET Imaging*, pages 285–301. Springer International Publishing, Cham, 2017.

[27] I. El Naqa, P. W. Grigsby, A. Apte, E. Kidd, E. Donnelly, D. Khullar, S. Chaudhari, D. Yang, M. Schmitt, R. Laforest, W. L. Thorstad, and J. O. Deasy. Exploring feature-based approaches in PET images for predicting cancer treatment outcomes. *Pattern recognition*, 42(6):1162–1171, 2009.

[28] European Society of Radiology (ESR). ESR statement on the stepwise development of imaging biomarkers. *Insights into imaging*, 4(2):147–52, 2013.

[29] V. Frings, F. H. P. van Velden, L. M. Velasquez, W. Hayes, P. M. van de Ven, O. S. Hoekstra, and R. Boellaard. Repeatability of metabolically active tumor volume measurements with FDG PET/CT in advanced gastrointestinal malignancies: a multicenter study. *Radiology*, 273(2):539–48, 2014.

[30] M. M. Galloway. Texture analysis using gray level run lengths. *Computer Graphics and Image Processing*, 4(2):172–179, 1975.

[31] R. C. Geary. The Contiguity Ratio and Statistical Mapping. *The Incorporated Statistician*, 5(3):115–145, 1954.

[32] L. Gjesteby, B. De Man, Y. Jin, H. Paganetti, J. Verburg, D. Giantsoudi, and G. Wang. Metal Artifact Reduction in CT: Where Are We After Four Decades? *IEEE Access*, 4:5826–5849, 2016.

[33] H. Gudbjartsson and S. Patz. The Rician distribution of noisy MRI data. *Magnetic resonance in medicine*, 34(6):910–4, 1995.

[34] E. L. Hall, R. P. Kruger, J. Samuel, D. Dwyer, R. W. McLaren, D. L. Hall, and G. Lodwick. A Survey of Preprocessing and Feature Extraction Techniques for Radiographic Images. *IEEE Transactions on Computers*, C-20(9):1032–1044, 1971.

[35] R. M. Haralick. Statistical and structural approaches to texture. *Proceedings of the IEEE*, 67(5):786–804, 1979.

[36] R. M. Haralick, K. Shanmugam, and I. Dinstein. Textural Features for Image Classification. *IEEE Transactions on Systems, Man, and Cybernetics*, 3(6):610–621, 1973.

[37] M. Hatt, M. Majdoub, M. Vallières, F. Tixier, C. C. Le Rest, D. Groheux, E. Hindié, A. Martineau, O. Pradier, R. Hustinx, R. Perdrisot, R. Guillevin, I. El Naqa, and D. Visvikis. 18F-FDG PET uptake characterization through texture analysis: investigating the complementary nature of heterogeneity and functional tumor volume in a multi-cancer site patient cohort. *Journal of nuclear medicine*, 56(1):38–44, 2015.

[38] M. Hatt, F. Tixier, L. Pierce, P. E. Kinahan, C. C. Le Rest, and D. Visvikis. Characterization of PET/CT images using texture analysis: the past, the present... any future? *European journal of nuclear medicine and molecular imaging*, 44(1):151–165, 2017.

[39] R. M. Heiberger and B. Holland. *Statistical Analysis and Data Display*. Springer Texts in Statistics. Springer New York, New York, NY, 2015.

[40] L. G. Khachiyan. Rounding of Polytopes in the Real Number Model of Computation. *Mathematics of Operations Research*, 21(2):307–320, 1996.

[41] P. Lambin, R. T. H. Leijenaar, T. M. Deist, J. Peerlings, E. E. C. de Jong, J. van Timmeren, S. Sanduleanu, R. T. H. M. Larue, A. J. G. Even, A. Jochems, Y. van Wijk, H. Woodruff, J. van Soest, T. Lustberg, E. Roelofs, W. J. C. van Elmpt, A. L. A. J. Dekker, F. M. Mottaghy, J. E. Wildberger, and S. Walsh. Radiomics: the bridge between medical imaging and personalized medicine. *Nature reviews. Clinical oncology*, 14(12):749–762, 2017.

[42] R. T. H. M. Larue, J. E. van Timmeren, E. E. C. de Jong, G. Feliciani, R. T. H. Leijenaar, W. M. J. Schreurs, M. N. Sosef, F. H. P. J. Raat, F. H. R. van der Zande, M. Das, W. J. C. van Elmpt, and P. Lambin. Influence of gray level discretization on radiomic feature stability for different CT scanners, tube currents and slice thicknesses: a comprehensive phantom study. *Acta oncologica*, pages 1–10, 2017.

[43] A. Le Pogam, H. Hanzouli, M. Hatt, C. Cheze Le Rest, and D. Visvikis. Denoising of PET images by combining wavelets and curvelets for improved preservation of resolution and quantitation. *Medical image analysis*, 17(8):877–91, 2013.

[44] R. T. H. Leijenaar, G. Nalbantov, S. Carvalho, W. J. C. van Elmpt, E. G. C. Troost, R. Boellaard, H. J. W. L. Aerts, R. J. Gillies, and P. Lambin. The effect of SUV discretization in quantitative FDG-PET Radiomics: the need for standardized methodology in tumor texture analysis. *Scientific reports*, 5(August):11075, 2015.

[45] T. Lewiner, H. Lopes, A. W. Vieira, and G. Tavares. Efficient Implementation of Marching Cubes' Cases with Topological Guarantees. *Journal of Graphics Tools*, 8(2):1–15, 2003.

[46] E. J. Limkin, S. Reuzé, A. Carré, R. Sun, A. Schernberg, A. Alexis, E. Deutsch, C. Ferté, and C. Robert. The complexity of tumor shape, spiculatedness, correlates with tumor radiomic shape features. *Sci. Rep.*, 9(1):4329, 2019.

[47] S. P. Lloyd. Least Squares Quantization in PCM. *IEEE Transactions on Information Theory*, 28(2):129–137, 1982.

[48] W. E. Lorensen and H. E. Cline. Marching cubes: A high resolution 3D surface construction algorithm. *ACM SIGGRAPH Computer Graphics*, 21(4):163–169, 1987.

[49] D. Mackin, X. Fave, L. Zhang, J. Yang, A. K. Jones, C. S. Ng, and L. Court. Harmonizing the pixel size in retrospective computed tomography radiomics studies. *PLOS ONE*, 12 (9):e0178524, 2017.

[50] J. Max. Quantizing for minimum distortion. *IEEE Transactions on Information Theory*, 6 (1):7–12, 1960.

[51] M. A. Mazurowski, N. M. Czarnek, L. M. Collins, K. B. Peters, and K. Clark. Predicting outcomes in glioblastoma patients using computerized analysis of tumor shape: preliminary data. In G. D. Tourassi and S. G. Armato, editors, *SPIE Medical Imaging*, volume 9785, page 97852T, 2016.

[52] P. A. P. Moran. Notes on continuous stochastic phenomena. *Biometrika*, 37:17–23, 1950.

[53] J. L. Mulshine, D. S. Gierada, S. G. Armato, R. S. Avila, D. F. Yankelevitz, E. A. Kazerooni, M. F. McNitt-Gray, A. J. Buckler, and D. C. Sullivan. Role of the Quantitative Imaging Biomarker Alliance in optimizing CT for the evaluation of lung cancer screen-detected nodules. *Journal of the American College of Radiology*, 12(4):390–5, 2015.

[54] R. J. Nordstrom. The quantitative imaging network in precision medicine. *Tomography*, 2(4):239, 2016.

[55] J. P. B. O'Connor, E. O. Aboagye, J. E. Adams, H. J. W. L. Aerts, S. F. Barrington, A. J. Beer, R. Boellaard, S. E. Bohndiek, M. Brady, G. Brown, D. L. Buckley, T. L. Chenevert, L. P. Clarke, S. Collette, G. J. Cook, N. M. DeSouza, J. C. Dickson, C. Dive, J. L. Evelhoch, C. Faivre-Finn, F. A. Gallagher, F. J. Gilbert, R. J. Gillies, V. Goh, J. R. Griffiths, A. M. Groves, S. Halligan, A. L. Harris, D. J. Hawkes, O. S. Hoekstra, E. P. Huang, B. F. Hutton, E. F. Jackson, G. C. Jayson, A. Jones, D.-M. Koh, D. Lacombe, P. Lambin, N. Lassau, M. O. Leach, T.-Y. Lee, E. L. Leen, J. S. Lewis, Y. Liu, M. F. Lythgoe, P. Manoharan, R. J. Maxwell, K. A. Miles, B. Morgan, S. Morris, T. Ng, A. R. Padhani, G. J. M. Parker, M. Partridge, A. P. Pathak, A. C. Peet, S. Punwani, A. R. Reynolds, S. P. Robinson, L. K. Shankar, R. A. Sharma, D. Soloviev, S. Stroobants, D. C. Sullivan, S. A. Taylor, P. S. Tofts, G. M. Tozer, M. van Herk, S. Walker-Samuel, J. Wason, K. J. Williams, P. Workman, T. E. Yankeelov, K. M. Brindle, L. M. McShane, A. Jackson, and J. C. Waterton. Imaging biomarker roadmap for cancer studies. *Nature Reviews Clinical Oncology*, 14(3):169–186, 2017.

[56] J. O'Rourke. Finding minimal enclosing boxes. *International Journal of Computer and Information Sciences*, 14(3):183–199, 1985.

[57] S. Sanduleanu, H. C. Woodruff, E. E. C. de Jong, J. E. van Timmeren, A. Jochems, L. Dubois, and P. Lambin. Tracking tumor biology with radiomics: A systematic review utilizing a radiomics quality score. *Radiother. Oncol.*, 127(3):349–360, 2018.

[58] S. Schirra. How Reliable Are Practical Point-in-Polygon Strategies? In *Algorithms - ESA 2008*, pages 744–755. Springer Berlin Heidelberg, Berlin, Heidelberg, 2008.

[59] M. Shafiq-Ul-Hassan, G. G. Zhang, K. Latifi, G. Ullah, D. C. Hunt, Y. Balagurunathan, M. A. Abdalah, M. B. Schabath, D. G. Goldgof, D. Mackin, L. E. Court, R. J. Gillies, and E. G. Moros. Intrinsic dependencies of CT radiomic features on voxel size and number of gray levels. *Medical physics*, 44(3):1050–1062, 2017.

[60] I. Shiri, A. Rahmim, P. Ghaffarian, P. Geramifar, H. Abdollahi, and A. Bitarafan-Rajabi. The impact of image reconstruction settings on 18F-FDG PET radiomic features: multi-scanner phantom and patient studies. *European Radiology*, 27(11):4498–4509, 2017.

[61] J. G. Sled, A. P. Zijdenbos, and A. C. Evans. A nonparametric method for automatic correction of intensity nonuniformity in MRI data. *IEEE transactions on medical imaging*, 17(1):87–97, 1998.

[62] K. Smith, Y. Li, F. Piccinini, G. Csucs, C. Balazs, A. Bevilacqua, and P. Horvath. CIDRE: An illumination-correction method for optical microscopy. *Nature Methods*, 12(5):404–406, 2015.

[63] L.-K. Soh and C. Tsatsoulis. Texture analysis of sar sea ice imagery using gray level co-occurrence matrices. *IEEE Transactions on Geoscience and Remote Sensing*, 37(2):780–795, 1999.

[64] M. Sollini, L. Cozzi, L. Antunovic, A. Chiti, and M. Kirienko. PET Radiomics in NSCLC: state of the art and a proposal for harmonization of methodology. *Scientific reports*, 7(1): 358, 2017.

[65] C. Solomon and T. Breckon. Features. In *Fundamentals of Digital Image Processing*, chapter 9, pages 235–262. John Wiley & Sons, Ltd, Chichester, UK, 2011.

[66] M. Soret, S. L. Bacharach, and I. Buvat. Partial-volume effect in PET tumor imaging. *Journal of nuclear medicine*, 48(6):932–45, 2007.

[67] P. Stelldinger, L. J. Latecki, and M. Siqueira. Topological equivalence between a 3D object and the reconstruction of its digital image. *IEEE transactions on pattern analysis and machine intelligence*, 29(1):126–40, 2007.

[68] D. C. Sullivan, N. A. Obuchowski, L. G. Kessler, D. L. Raunig, C. Gatsonis, E. P. Huang, M. Kondratovich, L. M. McShane, A. P. Reeves, D. P. Barboriak, A. R. Guimaraes, R. L. Wahl, and RSNA-QIBA Metrology Working Group. Metrology Standards for Quantitative Imaging Biomarkers. *Radiology*, 277(3):813–25, 2015.

[69] C. Sun and W. G. Wee. Neighboring gray level dependence matrix for texture classification. *Computer Vision, Graphics, and Image Processing*, 23(3):341–352, 1983.

[70] P. Thévenaz, T. Blu, and M. Unser. Image interpolation and resampling. In *Handbook of medical imaging*, pages 393–420. Academic Press, Inc., 2000.

[71] G. Thibault, J. Angulo, and F. Meyer. Advanced statistical matrices for texture characterization: application to cell classification. *IEEE transactions on bio-medical engineering*, 61(3):630–7, 2014.

[72] M. J. Todd and E. A. Yıldırım. On Khachiyan's algorithm for the computation of minimum-volume enclosing ellipsoids. *Discrete Applied Mathematics*, 155(13):1731–1744, 2007.

[73] A. Traverso, L. Wee, A. Dekker, and R. Gillies. Repeatability and reproducibility of radiomic features: A systematic review. *Int. J. Radiat. Oncol. Biol. Phys.*, 102(4):1143–1158, 2018.

[74] M. Unser. Sum and difference histograms for texture classification. *IEEE transactions on pattern analysis and machine intelligence*, 8(1):118–125, 1986.

[75] M. Vaidya, K. M. Creach, J. Frye, F. Dehdashti, J. D. Bradley, and I. El Naqa. Combined PET/CT image characteristics for radiotherapy tumor response in lung cancer. *Radiotherapy and oncology*, 102(2):239–45, 2012.

[76] M. Vallières, C. R. Freeman, S. R. Skamene, and I. El Naqa. A radiomics model from joint FDG-PET and MRI texture features for the prediction of lung metastases in soft-tissue sarcomas of the extremities. *Physics in medicine and biology*, 60(14):5471–96, 2015.

[77] M. Vallières, C. R. Freeman, S. R. Skamene, and I. El Naqa. Data from: A radiomics model from joint FDG-PET and MRI texture features for the prediction of lung metastases in soft-tissue sarcomas of the extremities, 2015.

[78] M. Vallières, E. Kay-Rivest, L. J. Perrin, X. Liem, C. Furstoss, H. J. W. L. Aerts, N. Khaouam, P. F. Nguyen-Tan, C.-S. Wang, K. Sultanem, J. Seuntjens, and I. El Naqa. Radiomics strategies for risk assessment of tumour failure in head-and-neck cancer. *Scientific reports*, 7:10117, 2017.

[79] M. Vallieres, A. Zwanenburg, B. Badic, C. Cheze-Le Rest, D. Visvikis, and M. Hatt. Responsible radiomics research for faster clinical translation, 2017.

[80] L. V. van Dijk, C. L. Brouwer, A. van der Schaaf, J. G. Burgerhof, R. J. Beukinga, J. A. Langendijk, N. M. Sijtsema, and R. J. Steenbakkers. CT image biomarkers to improve patient-specific prediction of radiation-induced xerostomia and sticky saliva. *Radiotherapy and Oncology*, 122(2):185–191, 2017.

[81] J. J. van Griethuysen, A. Fedorov, C. Parmar, A. Hosny, N. Aucoin, V. Narayan, R. G. Beets-Tan, J.-C. Fillion-Robin, S. Pieper, and H. J. Aerts. Computational radiomics system to decode the radiographic phenotype. *Cancer research*, 77(21):e104–e107, 2017.

[82] F. H. P. van Velden, P. Cheebsumon, M. Yaqub, E. F. Smit, O. S. Hoekstra, A. A. Lammertsma, and R. Boellaard. Evaluation of a cumulative SUV-volume histogram method for parameterizing heterogeneous intratumoural FDG uptake in non-small cell lung cancer PET studies. *European journal of nuclear medicine and molecular imaging*, 38(9): 1636–47, 2011.

[83] F. H. P. van Velden, G. M. Kramer, V. Frings, I. A. Nissen, E. R. Mulder, A. J. de Langen, O. S. Hoekstra, E. F. Smit, and R. Boellaard. Repeatability of Radiomic Features in Non-Small-Cell Lung Cancer [(18)F]FDG-PET/CT Studies: Impact of Reconstruction and Delineation. *Molecular imaging and biology*, 18(5):788–95, 2016.

[84] U. Vovk, F. Pernus, and B. Likar. A review of methods for correction of intensity inhomogeneity in MRI. *IEEE transactions on medical imaging*, 26(3):405–21, 2007.

[85] R. L. Wahl, H. Jacene, Y. Kasamon, and M. A. Lodge. From RECIST to PERCIST: Evolving Considerations for PET response criteria in solid tumors. *Journal of nuclear medicine*, 50 Suppl 1(5):122S–50S, 2009.

[86] J. C. Waterton and L. Pylkkanen. Qualification of imaging biomarkers for oncology drug development. *European journal of cancer*, 48(4):409–15, 2012.

[87] J. Yan, J. L. Chu-Shern, H. Y. Loi, L. K. Khor, A. K. Sinha, S. T. Quek, I. W. K. Tham, and D. Townsend. Impact of Image Reconstruction Settings on Texture Features in 18F-FDG PET. *Journal of nuclear medicine*, 56(11):1667–73, 2015.

[88] S. S. F. Yip and H. J. W. L. Aerts. Applications and limitations of radiomics. *Physics in medicine and biology*, 61(13):R150–66, 2016.

[89] C. Zhang and T. Chen. Efficient feature extraction for 2D/3D objects in mesh representation. In *Proceedings 2001 International Conference on Image Processing*, volume 2, pages 935–938. IEEE, 2001.

[90] A. Zwanenburg, S. Leger, L. Agolli, K. Pilz, E. G. C. Troost, C. Richter, and S. Löck. Assessing robustness of radiomic features by image perturbation. *Sci. Rep.*, 9(1):614, 2019.

This supplementary file describes the standardization of image segmentation and feature extraction.
